# A Genome-Wide Identification Analysis of Small Regulatory RNAs in *Mycobacterium tuberculosis* by RNA-Seq and Conservation Analysis

**DOI:** 10.1371/journal.pone.0032723

**Published:** 2012-03-28

**Authors:** Danilo Pellin, Paolo Miotto, Alessandro Ambrosi, Daniela Maria Cirillo, Clelia Di Serio

**Affiliations:** 1 University Centre for Statistics in the Biomedical Sciences, Università Vita-Salute San Raffaele, Milan, Italy; 2 Emerging Bacterial Pathogens Unit, San Raffaele Scientific Institute, Milan, Italy; National Institute for Infectious Diseases (L. Spallanzani), Italy

## Abstract

We propose a new method for smallRNAs (sRNAs) identification. First we build an effective target genome (ETG) by means of a strand-specific procedure. Then we propose a new bioinformatic pipeline based mainly on the combination of two types of information: the first provides an expression map based on RNA-seq data (Reads Map) and the second applies principles of comparative genomics leading to a Conservation Map. By superimposing these two maps, a robust method for the search of sRNAs is obtained. We apply this methodology to investigate sRNAs in *Mycobacterium tuberculosis H37Rv*. This bioinformatic procedure leads to a total list of 1948 candidate sRNAs. The size of the candidate list is strictly related to the aim of the study and to the technology used during the verification process. We provide performance measures of the algorithm in identifying annotated sRNAs reported in three recent published studies.

## Introduction

Modulation of gene expression through RNA regulators in bacteria is mediated by a heterogeneous group of molecules that act by various mechanisms. One of the most extensively studied class of regulators is represented by small non-coding RNAs (sRNAs) [1], [2], [3]. The growing list of discoveries in the field of sRNAs is the best indication of why it is indispensable for those involved in the field of genetics to gain knowledge about this critical regulatory concept. The typical size of these functional RNA molecules varies from 30 to 350 nucleotides. sRNAs can modulate transcription, translation, mRNA stability, and DNA maintenance or silencing. Thus, bacteria can regulate transcript expression through *cis*-acting sRNAs, acting in antisense manner and controlling the expression of mRNAs encoded on the opposite DNA strand, or *trans*-acting sRNAs. This alters the expression of multiple target mRNAs [4], [5].

sRNAs are involved predominantly in stress response pathways and pathogenesis, but they are also found to regulate metabolism, transport, quorum sensing, and other important physiological paths [1], [3], [5], [6]. RNA regulators may have several advantages over protein regulators and transcriptional factors in terms of cost, speed of response, complexity and specificity of the regulatory outcome [3], [5]. Bacterial capacity to survive in hostile environments has driven the evolution of specific mechanisms for monitoring, which adjust their gene expression and physiology accordingly to the hostile environment. This is crucial for pathogenic bacteria in constant interaction with the host during infection. The majority of sRNAs were discovered in *Escherichia coli*, and a smaller subset was characterized in other bacteria, including pathogenic species such as *Salmonella enterica* serovar Typhimurium, *Vibrio cholerae*, and *Mycobacterium leprae* [7], [8]. On *Mycobacterium tuberculosis* H37Rv (MTB) [9], the causative agent of tuberculosis, only few sRNAs have been identified and experimentally validated, and an experimental genome-wide approach was never adopted [10], [11]. Tuberculosis remains one of the leading causes of morbidity and mortality from infectious disease worldwide, and understanding the pathophysiology of MTB is imperative for developing new drugs and vaccines.

Most of the 140 bacterial sRNAs discovered in the past six years were identified by systematic screens using computational methods or experimental-based approaches, including microarray and shotgun cloning [12], [13]. A smaller number were discovered by direct labelling or by functional genetic screens.

Computational approaches predicting sRNAs commonly rely on comparative genomics analysis focused on intergenic genomic regions (IGRs); some examples are methods such as QRNA [14] and Intergenic Sequence Inspector [15]. However, these methods present some limitations being species-specific and discarding regions on the antisense strand of protein-encoding genes. Recently, further bioinformatic approaches for identification of sRNA molecules in bacteria have used different combinations of comparative genomics, GC content profiling, sequence alignment of IGRs with known sRNAs, together with the search for appropriate consensus sequences for transcriptional initiation and termination sites [12], [16], [17], [18]. However, the aforementioned approaches may be ineffective when studying mycobacteria due to different factors, such as their genomic composition, the difficulty in defining accurate transcriptional signals, and the lack of verified sRNAs.

Among experimental-based methods two approaches led to very promising results. The first [19], [20] is based on the search for sRNA in *Escherichia coli* by the analysis of low-molecular-weight RNA molecules isolated from cultures. According to this approach, nine putative sRNAs have been recently identified [11] in MTB. A second approach provides important suggestions to improve the accuracy of annotation of many genes dealing with strand-specific variation of RNA-seq [21], called single-strand RNA-seq, ssRNA-seq [22]; this approach also applies to the identification of novel sRNAs.

However, these methods suffer from some limitations. The main one is the strong dependency on both the amount of sRNAs present in the sample as well as experimental conditions. For this reason this methodology identifies only sRNAs that are highly expressed during the selected experimental conditions.

We propose a new bioinformatic approach for sRNAs identification based on both ssRNA-seq data and comparative genomics. We provide a genome-wide identification of sRNAs in MTB.

In section 2 we illustrate the statistical and bioinformatic fundamentals leading to our identification method, which consisted of the construction of both the expression map and the conservation map.

Section 3 shows the results of the proposed method applied to data from sRNA-seq experiment conducted specifically on *Mycobacterium tuberculosis* H37Rv.

## Methods

**Figure 1:**
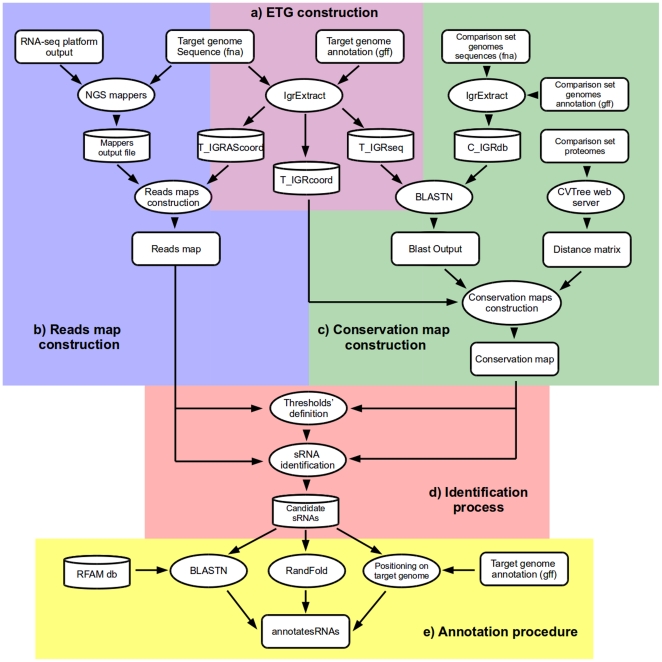
Outline of the bioinformatic pipeline. (a) Construction of the effective target genome (ETG) in terms both of sequences and coordinates. (b) Construction of the two strand specific reads maps. (c) Construction of two strand specific conservation maps. (d) Combination of reads and conservation map to allow for the identification of putative sRNA encoding regions. (e) annotations of putative sRNA to assess their reliability.

We introduce a method (summarized in Figure 1) which relies mainly on the combination of two genomic features: the first extracts information from RNA-seq data (Reads Map) and the second is based on IGR conservation analysis (Conservation Map). The reliability of sRNA candidates has been assessed by screening their genomic characteristics, such as secondary structure stability, similar to the already annotated sRNAs and others that will be discussed in further sections.

We next describe in detail the preliminary step, sRNAs screening maps methods, threshold criteria, sRNAs candidate definition and discuss the reliability of our approach.

### 2.1 Construction of the Effective Target Genome (ETG)

The first step of the analysis is the generation of the Effective Target Genome (ETG). Since our target region (IGR), all regions annotated as coding for proteins (CDS) or as coding for functional RNA molecules (tRNA, rRNA) are extracted and discarded. This procedure is performed by means of custom BioPerl [23] script named IGRExtract, which combines information about bacterial genome sequences (.fna) and annotation files (.gff) obtained from the NCBI FTP site and returns:

1. two strand-specific databases containing genomic coordinates (start, end position and strand) of regions which are not used as template for transcription (IGR+AS) named Target InterGenic AntiSense Region Coordinates -T_IGRAScoord-. The sum of these two databases corresponds to the Effective Target Genome (ETG).
2. one database containing genomic coordinates of IGR regions named Target InterGenic Region Coordinates -T_IGRcoord-. It represents a sub-sample of the ETG.
3. the third database reports DNA sequences corresponding to the IGR regions (Target InterGenic Region Sequences -T_IGRseq-).

### 2.2 Reads maps Construction

**Figure 2:**
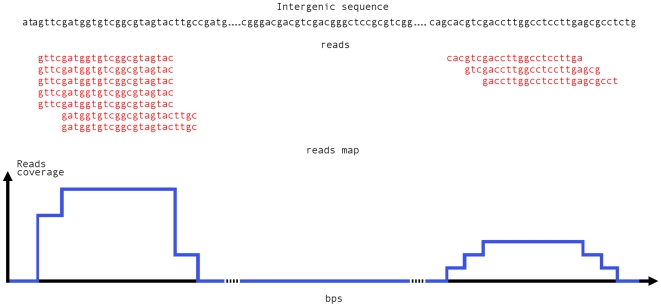
Reads maps construction. Reads map (blue curve) is obtained by assembling together all reads (sequences in red) mapping uniquely and completely within the same IGR or AS region (sequence in black). The BioPerl procedure implemented merges NGS mappers output and T_IGRAScoord files.

We next introduce a novel genome-wide approach to exploit RNA-seq technology for the identification of putative sRNAs encoded by transcriptional templates located in not annotated regions. The input needed in this step corresponds to the output of standard next generation sequencing mappers such as SOAPv1 [24], Bowtie [25], ELAND or BWA [26] and the previously described (see section 2.1) T_IGRAScoord database. A filtering procedure has been implemented in BioPerl to extract from these an alignment program output, those reads that uniquely map to any of the genomic intervals contained in each of the T_IGRAScoord files separately. Only reads completely mapping within the IGRAS are included. Two strand-specific reads maps are obtained. From these maps a coverage value is computed at a single base resolution for all screened bases of the Target InterGenic AntiSense genome. We define a *reads coverage value R_i_* of a specific genomic position *i* as the count of reads overlapping that position (Figure 2) given by:
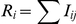
where *I_ij_* is the indicator function equal to 1 if the position *i* is between the start position and the end position of the generic reads *r_i_* and *i* can assume all values included in the regions contained in T_IGRAScoord database.

### 2.3 Conservation map construction

The following step is based on IGRs conservation analysis. The main advantage of this conservation- based method is also its main limitation. For example, the more an IGR is conserved among other species the more likely it can be considered as a functional unit for the genome. However, it is not possible to identify organism-specific coding elements. A first fundamental issue in conservation analysis is the choice of the comparative set of organisms. The number of genomes considered for the comparison as well as their relative evolutionary distance represents a crucial feature together with computational time required for the analysis. For instance, a large set, which includes distant genomes, leads to a computationally intensive procedure without significantly improving the identification power. On the other hand, a restricted set of genomes closer to the selected target produces a high conservation index diffused across all IGRs, thus leading to a reduction in terms of discriminatory power. This approach starts from the creation of single-base resolution conservation map. Unlike the reads map, the conservation map is not calculated in AS regions since these show a very high conservation degree. It is clear that the conservation map is symmetrical with respect to strand, since strands are defined starting from IGRs, which are complementary. First we extract information on genome sequence (.fna) and annotation files (.gff) from the NCBI FTP website for each bacterium included in the conservation set. Running the script (IGRExtract script defined in section 2.1) over the genomes set, a comparison database of IGRseq is created (C_IGRseq). Each IGR in T_IGRseq file was compared using BLASTN 2.0 [27] with the whole C_IGRdb (E-value threshold: 1e-2). By processing the obtained matches output file, a conservation map is subsequently created. The *sequence conservation value* (*C_i_*) of a specific genomic position corresponds to the weighted count of different genomes containing that position with at least one alignment satisfying quality criteria. Details on the definition of the sequence conservation value are reported in Text S1. The distance between genomes included in the analysis is calculated accordingly with the method proposed in the recent literature [28].

### 2.4 Thresholds definition

sRNAs identification procedure is based on the definition of reads and conservation thresholds which are established on empirical distribution functions determined by means of statistical criteria and according to data characteristics (Text S2). Factors such as mRNA degradation, background signal and genome complexity influence the thresholds identification. On the other hand, thresholds can affect candidate features like length, start-end positions, type, and secondary structure estimation.

A first general consideration for threshold definition is that each observation within a given base is the result of two sources of random values: background noise [29] and sRNA expression. We assume that these two sources of randomness are independent and apply the Empirical Distribution Function (EDF) to define thresholds. A detailed description of the statistical approach is provided in Text S2.

In particular, ExprT1 and ConsT1 thresholds correspond to 95^th^ percentile of reads abundance and conservation distributions respectively, while ExprT2 and ConsT2 thresholds correspond to the 90th percentile. Setting the threshold on a combination of background noise and sRNA candidates signal represents a conservative approach since it shifts the discriminating cut-off to a larger value and hence to a higher percentiles producing an higher significant effective level. From the comparative genomics step we consider the percentiles of the conservation distributions as *ad hoc* cut-offs to identify the more likely promising regions.

### 2.5 sRNA candidate definition

For each region stored in the strand-specific databases T_IGRAScoord files, reads and conservation maps are superimposed to identify putative regions encoding for sRNA candidates. As mentioned above, *conservation map* support is a subset of reads map support. In AS Regions the value of *C_i_* is set equal to 0. In particular we defined a Genomic Region (GR) as an sRNA candidate if all of the following conditions are satisfied:

- genomic region made up by consecutive nucleotides
- genomic region length ≥30 nt and ≤550 nt

In addition, at least one of the following conditions must be tested and satisfied on GR base, *GR_i_* (for thresholds definition see section 2.4):

- all *GR_i_* has reads abundance *R_i_* value≥ExprT1 threshold
- all *GR_i_* has a conservation value *C_i_*≥ConsT1 threshold
- all *GR_i_* interval has an abundance reads *R_i_* value≥ExprT2 threshold and a conservation value *C_i_*≥ConsT2 threshold.

**Figure 3:**
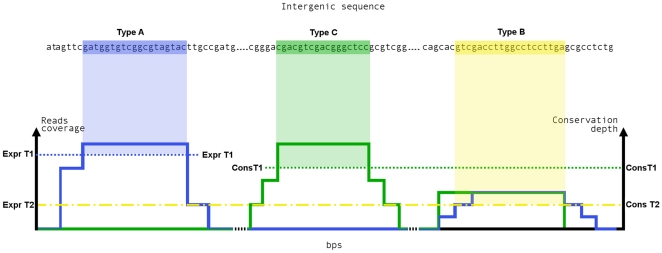
SRNA identification process. For each IGR (sequence in black), reads (blue curve) and conservation (green curve) maps are superimposed. First Type A candidates (highlighted in blue) are identified and extracted by testing length constrains (conditions I and II) and reads coverage above ExprT1 (dotted blue line). On the remaining portions of IGRs, Type B candidates (highlight in yellow) are identified and extracted by testing length constrains (conditions I and II) and contemporaneously both reads coverage above ExprT2 and conservation depth above ConsT2 (dot and dashed yellow lines). Finally, Type C candidate (highlighted in green) are identified in the remaining IGRs on the basis of high sequence conservation (above ConsT1 threshold reported as dotted green line).

Different combinations of three of the previous testing conditions lead to three possible candidate types (type A, type B, type C) (Figure 3). The selection of the candidate type is crucially affected by the order of testing conditions. In other words finding type A candidates should be the first step which influences step 2 for the search of type B and step 3 for identifying C candidates.

Step 1: identification of regions satisfying conditions I, II and III (type A candidates);
Step 2: identification of regions satisfying conditions I, II and V (type B candidates);
Step 3: identification of regions satisfying conditions I, II and IV (type C candidates).

The application of these steps will enable the identification of “highly expressed” candidates, “mildly expressed” candidates that show conservation features, and “lowly expressed” candidates conserved among the phylogenetic genus. Accordingly to this hierarchical approach, we should obtain “bona fide” candidates identified on evidence criteria such as expression means (Type A), expression plus conservation (Type B), and conservation alone (Type C). We would also expect that the most promising candidates are included in the category Type A, whereas Type B and Type C could include candidates with an higher number of false positives.

By fulfilling expression-based conditions a first experimental validation is provided. Thus we start data processing by scanning all regions specified in T_IGRAScoord, for type A candidates which are then removed from the ETG to select regions satisfying the expression constraints. On the remaining ETG, type B candidates are identified and removed. Last, residual ETG is analysed to search for type C candidates. This procedure clearly implies that sRNAs candidates lying on AS Regions (with *C_i_* = 0) can only be type A candidates. In IGRs all types of candidates are identifiable. We remark that type C candidates based on conservation only, could also lie on opposite strands (with the same coordinates). Reads map can be used to select the more likely strand. Therefore, results are highly dependent on the order of the scanning criteria applied, as mentioned above. Note that type A candidates are the most promising ones.

Within the analysis of spatial distribution of reads derived from NGS technology along the genome a well-known problem is the presence of un-mappable regions in the investigated genome which represents a crucial bias factor [30], [31]. For example, a possible misleading scenario may occur in the presence of a non-mappable genomic interval inside a true candidate. This type of *blind* portion of the genome corresponds to not uniquely mappable reads sequences. These reads are filtered out in the reads map construction step, showing a decrease in the expression signal. Whenever these “blind regions” have a considerably larger length, the reads map signal goes below ExprT1 threshold, splitting a single candidate into multiple, shorter ones. This leads to an unreliable predictions dataset (in terms of both total amount of sRNA as well as start and end coordinates). Unreliability refers also to secondary structure stability, target predictions and other sequence dependent information. To overcome this genome mappability problem basically related to NGS technology, we implemented an additional merging procedure focused on type A candidates. To be more specific, the procedure binds together two consecutive type A candidates located on the same strand if, and only if, their distance is less than a given threshold and the total length satisfies the above mentioned length constraints.

We last remark that in the merging procedure of type A candidates, the threshold must assume a small value according with reads length, mappability profile for the investigated genome, minimum length of identifiable sRNA and typical length of the promoter region.

### 2.6 Assessing prediction reliability

In order to assess and to minimize the risk of identifying false positive sRNAs from the list of candidates, many different characteristics of each putative sRNA are analyzed. Parts of these are commonly used in *in silico* identification algorithms whereas others are new. More specifically, identified sRNAs are annotated for

- maps derived information:
  mean reads coverage value across the candidate
  mean conservation across the candidate
- position within the genome:
  candidate type (*trans*-encoded, coding region *cis*-encoded, 5′/3′ *cis*-encoded)
  distance from closest upstream and downstream annotated gene
- secondary structure stability (Minimum Free Energy associated p-value):
  Minimum Free Energy (MFE) calculated by means of Randfold algorithm [32]
- homology with previously verified sRNA annotated in RFAM database version 10.0 [33].
  primary structure conservation using BLASTN comparison (E-value<1e-2).
  secondary structure conservation using Infernal algorithm [34] (E-value<1e-2).

Specifically, we define as *trans*-encoded sRNAs those candidates located between stop and start codons of CDSs. We do not fix any minimum distance from flanking encoding regions to avoid possible bias. *Cis*-encoded antisense sRNA (in the opposite direction of a transcription unit) are divided into two main groups depending on the relative location of the associated gene. Coding regions *cis*-encoded are located antisense to the coding region and 5′/3′ *cis*-encoded are located antisense to the start or end region of a coding region.

All these criteria may lead to different constraints depending on the aim of the experiment. For instance, the definition of precise constraints on average reads abundance or homology with sRNA stored in the RFAM database, enable us to select a subset of very promising sRNA encoding regions. Generally, the criteria and consequently the dimension of the validation subset depend on verification technology and can vary from 1000–2000 candidates for massive custom-designed microarray-based verification to 1–10 for northern blot approach. Indeed, if we were interested in a particular genomic region, for example *cis*-encoded sRNA of a specific target gene, a position- and type-specific filtering could be performed.

## Results

Our algorithm was tested on data from sRNA-seq experiments conducted specifically to identify and provide novel insights on the regulatory network mediated by novel sRNAs in *Mycobacterium tuberculosis* H37Rv. *M. tuberculosis* H37Rv genome (NCBI RefSeq Accession NC_000962) is ∼4.4 million bps long and hosts 4047 genes (protein coding+t/rRNA). Due to different factors, such as the high GC Content (61.65%) typical of Mycobacterium species, the lack of a well conserved rho-independent terminator sequence and the absence of verified sRNA in related species, most computational approaches for sRNA analysis do not perform well on the MTB genome.

### 3.1 MTB Effective Target Genome

After removing the genomic regions used as the template and transcribed in RNA by means of IGRExtract script we obtained an ETG of a length equal to about half of the whole genome. To be more specific, regions in T_IGRAScoord retrieved from the positive strand are 1669, corresponding to 2443765 nucleotides (nt) and those retrieved from the negative one are 1677 corresponding 2362067 nt (*M. tuberculosis* H37Rv complete genome: 4,411,532 nt). The total number of regions stored in T_IGRcoord and corresponding sequences in T_IGRseq is 3218.

### 3.2 sRNA-seq data

For direct sequencing of sRNAs, *M. tuberculosis* H37Rv was grown in Middlebrook 7H9 medium, supplemented with 10% OADC (Oleic Acid, Albumin, Dextrose, Catalase). Exponential growth phase culture was harvested at OD_600_ between 0.5 and 0.8 and washed twice in PBS. The sRNA fraction was extracted using the mirVana miRNA Isolation kit (Ambion) according to the manufacturer's instructions. Both sRNA and total RNA (depleted of sRNAs) fractions were collected and checked by Bioanalyzer analysis (Agilent Biotechnologies). The sRNA fraction was then sequenced through the Illumina high-throughput sequencing approach at GATC Biotech AG (Konstanz, Germany). According to the protocol used for cDNA library preparation, RNA samples were poly(A)-tailed using poly(A) polymerase followed by ligation of a RNA adapter to the 5′-phosphate of the sRNAs. First-strand cDNA synthesis was then performed using an oligo(dT)-adapter primer and the M-MLV reverse transcriptase. The resulting cDNAs were PCR-amplified using a high fidelity DNA polymerase.

Illumina sequencing produces 36-base reads; these have been computationally processed to remove the poly-A tail. Resulting sequences show an average length of 20 nt. These were then mapped to the whole genome using SOAPv1 tool (seed size parameters: 6; maximum gap size allowed: 1).

After running SOAP on 9711909 reads from the Illumina platform we combined SOAPv1 output with T_IGRcoor databases. The total dataset was split as follow:

3548742 reads mapping to previously annotated CDS (27%)
1507477 reads repeatedly mapped or not mapped (11%)
8204432 reads mapping uniquely to any interval contained in T_IGRAScoor databases (4042926 on strand plus+4161506 on strand minus) (62%)

### 3.3 Genomes comparison set and conservation analysis

As a comparison set, 21 genomes were selected for conservation analysis, corresponding to all annotated organisms of the same genus of MTB. Conservation distances between the comparison set and the target genome were computed by means of the CVTree web server [28], accounting for standard parameter setting. From the matrix of distances (Table S1) and cluster analysis (Figure S1) one can easily see that, as expected, all genomes belonging to *Mycobacterium tubercolosis* complex are very close (minimum distance equal to 0.0066 in *Mycobacterium tuberculosis* H37Ra and maximum distance equal to 0.0365 in *Mycobacterium tuberculosis* CDC1551). The genomes of the remaining bacteria have distances from MTB between 0.399 (*Mycobacterium marinum* M) and 0.46 (*Mycobacterium abscessus*). The complete list of weights *w_j_* is reported in Table 1.

**Table 1:** Complete list of weights *w_j_* for conservation map calculation.

Genome	Ref seq Accession	*w_j_*
***Mycobacterium tuberculosis*** ** H37Ra**	**NC_009525**	**0.00661**
***Mycobacterium tuberculosis*** ** F11**	**NC_009565**	**0.01299**
***Mycobacterium tuberculosis*** ** KZN 1435**	**NC_012943**	**0.01555**
***Mycobacterium bovis*** ** AF2122/97**	**NC_002495**	**0.01948**
***Mycobacterium bovis*** ** BCG str. Tokyo 172**	**NC_012207**	**0.02284**
***Mycobacterium bovis*** ** strain Pasteur 1173P2**	**NC_008769**	**0.02404**
***Mycobacterium tuberculosis*** ** CDC1551**	**NC_002755**	**0.03652**
*Mycobacterium marinum* M	NC_010612	0.39919
*Mycobacterium avium subsp. paratuberculosis* K-10	NC_002944	0.40764
*Mycobacterium ulcerans* Agy99	NC_005916	0.41261
*Mycobacterium avium*	NC_008595	0.41444
*Mycobacterium leprae* TN	NC_002677	0.41792
*Mycobacterium leprae*	NC_011896	0.41802
*Mycobacterium sp.* MCS	NC_008146	0.44789
*Mycobacterium sp.* KMS	NC_008705	0.44793
*Mycobacterium sp.* JLS	NC_009077	0.44918
*Mycobacterium sp.* Spyr1	NC_014814	0.45108
*Mycobacterium gilvum* PYR-GCK	NC_009338	0.45114
*Mycobacterium vanbaalenii* PYR-1	NC_008726	0.45129
*Mycobacterium smegmatis* str. MC2 155	NC_008596	0.45333
*Mycobacterium abscessus* ATCC 19977	NC_010397	0.46
	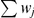	**6.22**

For each genome of the comparison set the corresponding Ref Seq accession number and the evolutionary distance from MTB genome are reported. The sum of *w_j_* is equal to 6.22 that correspond to the upper limit of conservation value *C_i_*.

The final IGR database contains 148140 sequences. The BLASTN output lists 133591 hits satisfying the quality criteria of e-value<1e-2.

### 3.4 Thresholds definition

Empirical distribution of percentiles (Table 2) of reads abundance leads us to set ExprT1 (95% percentile) to 50 and ExprT2 (90% percentile) to 22. Conservation thresholds ConsT1 and ConsT2 are fixed to 1.77 and 0.95, respectively. The threshold distance between two candidates in the merging procedure is fixed to 30 bps. This value derives from considerations about promoter region length in bacteria (35 bps) and reads average length (20 bps).

**Table 2:** Summary of reads coverage and conservation depth empirical distributions.

	Reads coverage	Conservation depth
*Min*	0	*0*
*25.00%*	0	*0.1*
*50.00%*	1	*0.14*
*75.00%*	6	*0.14*
*90.00%*	22	*0.95*
*95.00%*	50	*1.77*
*99.00%*	260	*3.97*
*Max*	79033	*6.22*

### 3.5 Candidates sRNA encoding region

In total, 1948 sRNA candidates were identified (891 strand+; 1057 strand −). Subdivisions in terms of candidate type and relative position to other genes in the genome are summarized in Table 3; details are available in Table S2. sRNA candidates in regions which are *cis*-encoded and 5′/3′ *cis*-encoded have at least one hit within an AS Region. Therefore, these can be only type A candidates. Type C candidates are reported in Table 3 and are symmetric and unique (data in parenthesis are divided into the two typologies).

**Table 3:** Candidate classification based on candidate definition provided in 2.4.

	Coding region *cis*-encoded	*Trans*-encoded	5′/3′ *cis*-encoded
Strand +			
type A	329	190	82
type B	0	61	0
type C	0	229(190+39)	0
Strand −			
type A	432	217	128
type B	0	53	0
type C	0	227 (186+41)	0
	761	977	210
1948			

SRNA candidate length range from 30 to 543 nt with an average length equal to 67 (median value equal to 46). Among these candidates, 237 show a secondary structure MFE p-value<5e-2, calculated by means of the Randfold algorithm using simple a mononucleotide shuffling method (Table S2).

Type A candidates show a mean reads abundance value varying between 59 and 24770, with an average value of 342. Within this group there are 120 (27%) candidates localized in IGR (220+212), which presents an average conservation value which is higher than 90% of the distribution. These candidates (65 are over 95-th percentile) are particularly promising since they are selected both on expression information and high conservation degree. Moreover, some of these (36 out of 120) present a p-value associated to MFE score<5e-2.

### 3.6 Candidate Selection Strategy

In the literature, existing computational approaches for identifying bacterial sRNAs demonstrate an increasing level of success, being based on both computational and experimental evidence [18]. Our approach has the advantage of accounting for expression and conservation aspects within a sequential approach, by testing before the deep sequencing data and then the conservation comparison.

The algorithm that we propose here, once the statistical threshold is set on the available data, leads to a total amount of ∼2000 sRNAs in MTB. Two main features are fundamental. First, considering that totally 14 over 27 (B11 and F6 sRNAs in [10] correspond to Mcr14 and Mpr19 in [11]) previously annotated sRNAs are identified by means of both computational and experimental approaches, we are confident on a high level identification performance since predictions obtained on MTB are strongly consistent with annotated and verified sRNAs.

Second, due to the large number of candidates sRNAs identified, criteria are needed to enable selection of the most promising candidates. We consider as a filter for the putative *trans*-encoded sRNAs a minimum distance from upstream and downstream genes (50 bps) and for a minimum free energy (MFE) p-value ≤5e-2. Thus, we obtain a shorter list of 59 candidates, where 4 out of 5 *trans*-encoded sRNAs reported in [10] (C8 pvalue = 0.08) and Mcr3 annotated in [11] are included. By ranking this short-list for mean reads abundance, we can extract the most promising *trans*-encoded sRNAs.

A further possible filtering strategy can be designed on available sRNA target prediction algorithms, such as IntaRNA [35], sRNATarget [36], TargetRNA [37], where possible interaction with genes involved in particular pathways such as virulence control can be investigated. Regarding putative *cis*-encoded sRNA, the target mRNA is fixed to the antisense gene. We preserve only those interactions with a score above a given threshold, depending upon the algorithm.

## Discussion

Elucidating the mechanisms of tuberculosis pathogenesis is a key-point in achieving disease control. Although MTB is one of most prominent bacterial pathogens, the presence of sRNAs has only recently been investigated as a post-transcriptional regulatory mechanism. Due to particular characteristics of the mycobacterial genome, a “pure” computational approach is not reliable. This work presents a genome-wide screening for sRNA identification in MTB that combines experimental and computational approaches for the first time. Further characterization of sRNAs in MTB will open a broad range of opportunities in the field.

In this paper we propose a new bioinformatic pipeline for identification of sRNAs in *Mycobacterium tubercolosis* A robust method for the search of sRNAs is obtained by sequentially testing information from an expression map and a conservation map. This approach can be conceptualized by superimposing the two maps as shown in Figure 3. The procedure leads to new sRNAs candidates for MTB.

In Table 4 we compare our sRNAs candidate regions with those previously verified in the literature [10] for coding sRNAs.

**Table 4:** Comparison with Arnvig, et al. [10] annotated sRNA.

Arnvig, KB., *et al.*, 2009 annotated sRNA			Candidate identified by our method						
sRNA	Start	End	Id	Type	Start	End	meanExpr	meanCons	mfePvalue
Trans-encoded sRNA									
B11	4099478	4099386	candidate_1603	A	4099477	4099384	2564.23	4.12	0
B55	704187	704247	candidate_84	A	704187	704246	3713.08	0.13	0
C8	4168281	416815441682124168224	candidate_1621	A	4168281	4168193	970.13	3.68	0.08
F6	293604	293641293661293705	candidate_29	A	293604	293662	634.93	1.88	0
G2	19151901915028	19149621914977	candidate_1269	A	1915164	1915013	548.41	0.14	0.01
Cis-encoded sRNA									
ASdes	918264918350918365	918432918412918458	candidate_121	A	918327	918360	256.03	0	0.47

More specifically, nine sRNA candidates are so classified: five are trans-encoded (B11, B55, C8, F6 and G2) and four are cis-encoded (antisense -AS- to *desA1*, *pks12*, *Rv 1726* and *Rv 1890c*). For trans-encoded sRNAs we highlight the successful performance of the algorithm, especially for those with one distinct processed 5′ end (B11, B55). Decreasing accuracy in start and end coordinates identification is observed for all the *trans*- and *cis*-encoded sRNAs showing multiple 5′ ends (C8, F6, G2 and ASdes) as expected. No candidates result in regions around the remaining 3 antisense sRNAs (AS*pks12*, AS*Rv 1726* and AS*Rv 1890c*). This is probably due to a very low amount or absence of transcript in RNA-seq experiment conditions, corresponding to a very small amount of reads mapping in those genomic intervals. This is likely to occur especially for ASpks, that has only been observed under certain stress conditions [10].

To reinforce the robustness of our methodology we performed a second comparison with results reported in [12] where the SIPHT algorithm was applied to the MTB genome. SIPHT is a “pure computational” tool that utilizes workflow management and distributed computing to effectively conduct kingdom-wide predictions and annotations of intergenic sRNA-encoding genes. This algorithm considers a few sequence features such as the presence of putative Rho-independent terminators and intergenic sequence conservation in the identification step, and several other features in the annotation of sRNAs encoding loci step. Using SIPHT, a total number of 102 candidates were identified in MTB (http://newbio.cs.wisc.edu/sRNA/). We remark that the searching region investigated by SIPHT algorithm, is different from our ETG. In Livny's paper [12] a genomic region is considered as intergenic if none of the two strands is used as a template for coding, that corresponds to our IGR. Therefore, to perform a correct cross-check with our results, we have to consider only candidates defined as trans-encoded (977 candidates). 27 genomic loci are defined as possible coding for sRNAs according to both methods (Table S3).

Most putative sRNA encoding regions identified with a corresponding SIPHT result are type A candidates (type A:16, type B:8,type C:14). We reveal a significant difference in length and in start-end coordinates identification between datasets with in some cases more than one candidates corresponding to one SIPHT putative sRNA. Since there is no experimental-based (Northern Blot, tiling array) validation on Livny predictions [12], performance measures to compare the two algorithms cannot be provided. Despite that, since thresholds are defined in a highly conservative manner and the possible problems previously described can occur also at the start/end positions of candidates, an underestimation of sRNAs length may occur.

In Di Chiara et al. [11] several sRNA are identified in *Mycobacterium bovis* BCG by means of Northern Blot. The identification procedure was derived by in silico predictions based on the SIPHT (Mpr) algorithm, along with an experimental approach based on cloning (Mcr). In total, 37 candidates were obtained and validated on *Mycobacterium bovis BCG* (19 Mcr and 18 Mpr). Twenty (11 Mcr and 9 Mpr) out of 37 were present also in MTB. To compare these candidates with ours, we extracted sequences of all 37 candidates using the same start and end position predicted and available in [11]. The dataset so obtained was compared by means of BLASTN algorithm with our MTB candidate sequences, after setting a cut-off on e-value<0.1 (Table 5).

**Table 5:** Comparison with DiChiara, et al. [11] sRNAs annotated in *Mycobacterium bovis* BCG.

SRNAs verified in *Mycobacterium bovis* BCG in DiChiara et al [11]				Candidate identified by our method						
Id	Start	End	Verify by Northern in MTB	Id	Type	Start	End	meanExpr	meanCons	e-value
Mcr3,Mpr7	1498201	1498256	+	candidate_190	A	1471657	1471737	24422.29	3	1.00E-024
Mcr4	2137103	2137148	−	candidate_1314	A	2136173	2136126	10706	0.22	1.00E-021
Mcr6	4141762	4141802	+	candidate_1621	A	4168281	4168193	970.13	3.68	9.00E-019
Mcr8	4073966	4073908	+	candidate_1935	C	4100859	4100792	13.72	3.06	3.00E-029
Mcr9,Mpr14	3317517	3317634	−	candidate_1502	A	3363153	3363023	215.58	2.04	3.00E-064
Mcr11	1439808	1439904	+	candidate_1693	B	1413139	1413102	330.15	0.96	1.00E-016
Mcr14	321693	321658	+	candidate_1676	B	293659	293603	388.51	1.96	6.00E-013
Mpr1	2813325	2813408	−	candidate_801	C	2849576	2849542	0.83	2.58	8.00E-015
Mpr3	935527	935628	−	candidate_710	C	905089	905185	19.96	4.8	3.00E-042
Mpr4	4073793	4074096	+	candidate_561	A	4100684	4100816	167.03	2.37	1.00E-072
Mpr5	1205934	1205651	+	candidate_1142	A	1292095	1291823	272.63	0	0.09
Mpr8	1857323	1857431	−	candidate_1822	C	1852175	1852137	3.1	2.59	4.00E-017
Mpr10	2300720	2300817	−	candidate_330	A	2522164	2522218	190.9	2.01	1.00E-007
Mpr15	3506470	3506359	+	candidate_846	C	3551163	3551221	0	3.19	5.00E-026
Mpr18	4066488	4066544	+	candidate_1155	A	1321893	1321807	147.74	0	0.02
Mpr19	4072633	4072493	+	candidate_877	C	4099384	4099497	3.26	3.93	3.00E-046
Mpr21	818357	818428	−	candidate_1881	C	3215656	3215591	1.53	3.12	2.00E-008

With regards to sRNAs based on cloning (Mcr), 5 candidates validated in MTB were very close to our candidates. Moreover, 2 Mcr in MTB which are not validated in [11] show homologous Type A candidates in our prediction dataset (Mcr4 and Mcr9).

As regarding Mpr candidates, 5 out of 9 validated in MTB also have a homologue in our database. 7 sRNAs, which Di Chiara et al. have identified in *bovis* BCG but not in MTB, have a high degree of homology with our candidates.

Homology with a custom library of annotated sRNAs stored in RFAM database (excluding human, viral, and microRNA sequences) was investigated from both primary and secondary structure levels. Regarding primary structure conservation, each candidate sequence was searched against the custom library using BLASTN, with an E-value threshold of 1e-2. The total number of matches was 757, tracing back to 27 different candidates and 23 different RFAM families (Table S4). The more significant were candidate_852 (e-value 3E-048) that showed a high homology with SAM-IV riboswitches (RF00634), candidate_1740 (e-value 1E-046) very similar to RF00059 TPP riboswitches family, candidate_766 and candidate_754 (e-value 2e-36 7e-29) similar to RF00380 YkoK leader family.

To verify conservation in terms of secondary structure, we compared our candidate sequences with RNA secondary structure profile by means of the INFERNAL software package [34] with an E-value threshold of 1e-2 (Table S5). As in BLASTN comparison, the more significant homologies are between candidate_852 (e-value 1.23e-22) An interesting homology is discovered between 6C RNA family and candidate_877 (e-value 1.35e-14).

It is commonly known that sRNAs play an important functional role in the physiology and virulence of many bacterial species. A critical question is to determine how sRNAs contribute to bacterial adaptation mechanisms [10], [12]. Our results will enable us to develop conditional mutants to elucidate the regulation of the stress response in *M. tuberculosis* and to better understand the role of specific metabolic pathways in the mechanism of adaptation to the human immune system.

The proposed approach represents a new perspective to the extent that it relies on the use on combing expression data from RNA-seq to address the identification of new molecules on a genome-wide scale with comparative genomics information. The algorithm here proposed merges information deriving from expression data and conservation analysis, and via a data-driven threshold definition method leads to a total number of ∼2000 sRNAs in MTB. Considering that in total 14 out of 27 (B11 and F6 sRNAs in [10] correspond to Mcr14 and Mpr19 in [11]) different sRNAs previously annotated are identified by means of both computational and experimental approaches, we are confident on a high-level identification performance since predictions obtained on MTB are strongly consistent with annotated and verified sRNAs.

## Supporting Information

Figure S1 **Heatmap of distances between genomes in comparison set and reference genome.**
(TIFF)

Table S1 **Matrix of distances between genomes.** Table contains all distances between the 22 genomes calculated by CVTree web server.
(DOC)

Table S2 **Complete list of identified sRNAs.** Table contains information regarding the whole list of candidate sRNAs identified.
(DOC)

Table S3 **Comparison with Livny J, et al., 2007** [**12**] **putative sRNA encoding loci.**
(DOC)

Table S4 **BLASTN output of comparison between candidates sequences and Rfam V.10 database.**
(DOC)

Table S5 **Infernal output of comparison between secondary structure candidates sequences and secondary structure of ncRNA in Rfam V.10 database.**
(DOC)

Text S1 **Conservation map construction.**
(DOC)

Text S2 **Thresholds definition.**
(DOC)
