# Supplementary figures and images for: A Genome-Wide Identification Analysis of Small Regulatory RNAs in Mycobacterium tuberculosis by RNA-Seq and Conservation Analysis
Source: PLoS One. 2012 Mar 28;7(3):e32723. doi: 10.1371/journal.pone.0032723 (PMC3314655; doi:10.1371/journal.pone.0032723)

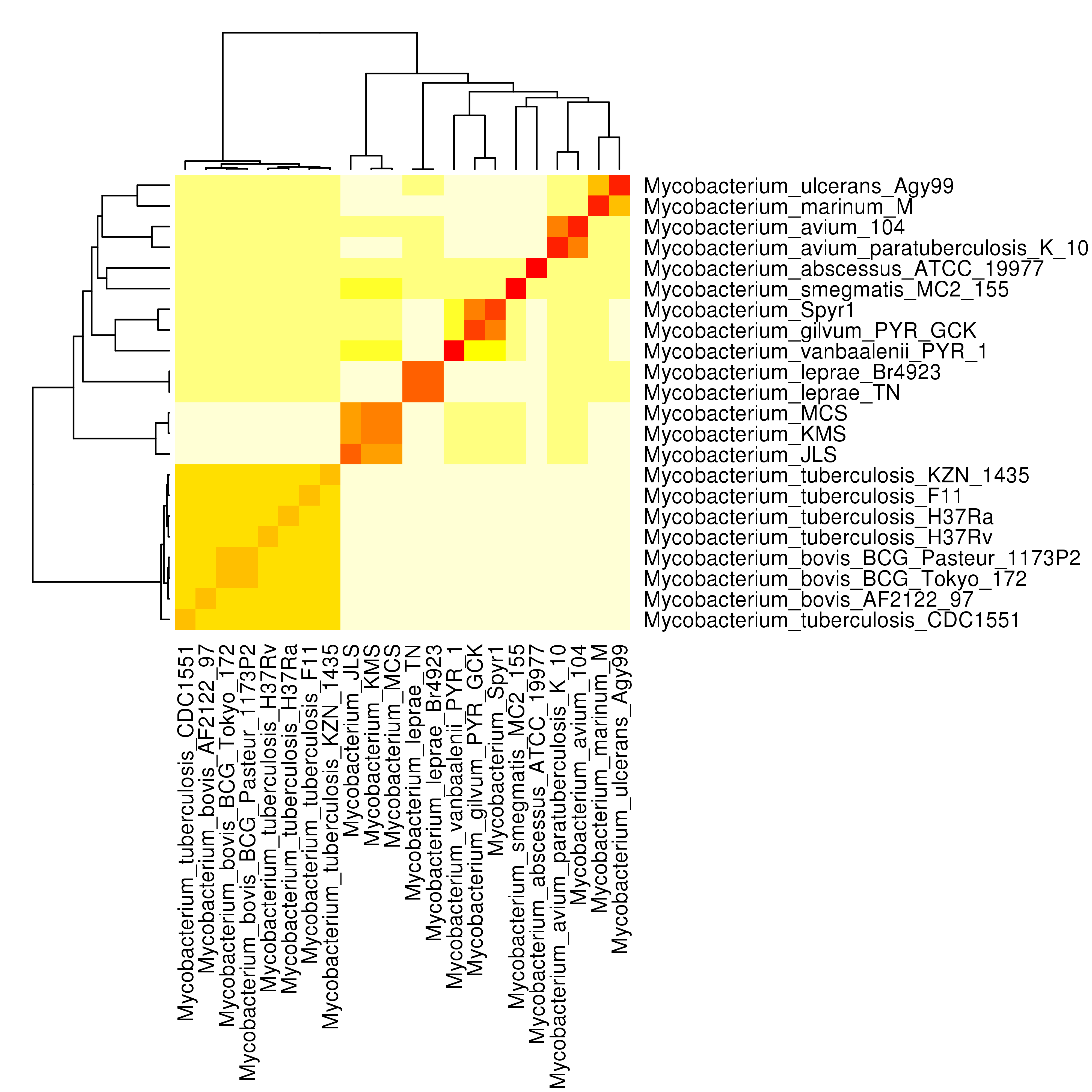

Supplement: Figure S1 — Heatmap of distances between genomes in comparison set and reference genome. (TIFF) [file pone.0032723.s001.tif]
