# Supplementary material for: A Genome-Wide Identification Analysis of Small Regulatory RNAs in Mycobacterium tuberculosis by RNA-Seq and Conservation Analysis
Source: PLoS One. 2012 Mar 28;7(3):e32723. doi: 10.1371/journal.pone.0032723 (PMC3314655; doi:10.1371/journal.pone.0032723)
Supplement: Table S1 — Matrix of distances between genomes. Table contains all distances between the 22 genomes calculated by CVTree web server. (DOC) [file pone.0032723.s002.doc]

|  | G1 | G2 | G3 | G4 | G5 | G6 | G7 | G8 | G9 | G10 | G11 | G12 | G13 | G14 | G15 | G16 | G17 | G18 | **G19** | G20 | G21 | G22 |
| --- | --- | --- | --- | --- | --- | --- | --- | --- | --- | --- | --- | --- | --- | --- | --- | --- | --- | --- | --- | --- | --- | --- |
| G1 | 0 | 0.08 | 0.09 | 0.4 | 0.46 | 0.44 | 0.44 | 0.45 | 0.45 | 0.45 | 0.4 | 0.46 | 0.46 | 0.45 | 0.42 | 0.45 | 0.45 | 0.45 | **0.45** | 0.45 | 0.46 | 0.41 |
| G2 | 0.08 | 0 | 0.01 | 0.4 | 0.45 | 0.44 | 0.44 | 0.45 | 0.45 | 0.45 | 0.4 | 0.46 | 0.46 | 0.45 | 0.42 | 0.45 | 0.45 | 0.45 | **0.45** | 0.45 | 0.46 | 0.4 |
| G3 | 0.09 | 0.01 | 0 | 0.4 | 0.45 | 0.44 | 0.44 | 0.45 | 0.45 | 0.45 | 0.4 | 0.46 | 0.46 | 0.45 | 0.42 | 0.45 | 0.45 | 0.45 | **0.45** | 0.45 | 0.46 | 0.4 |
| G4 | 0.4 | 0.4 | 0.4 | 0 | 0.46 | 0.45 | 0.44 | 0.45 | 0.45 | 0.45 | 0.08 | 0.46 | 0.46 | 0.45 | 0.43 | 0.45 | 0.45 | 0.45 | **0.45** | 0.45 | 0.46 | 0.35 |
| G5 | 0.46 | 0.45 | 0.45 | 0.46 | 0 | 0.46 | 0.46 | 0.46 | 0.46 | 0.46 | 0.46 | 0.47 | 0.47 | 0.46 | 0.46 | 0.46 | 0.46 | 0.46 | **0.46** | 0.46 | 0.47 | 0.46 |
| G6 | 0.44 | 0.44 | 0.44 | 0.45 | 0.46 | 0 | 0.13 | 0.41 | 0.41 | 0.41 | 0.45 | 0.44 | 0.44 | 0.42 | 0.45 | 0.42 | 0.41 | 0.41 | **0.41** | 0.41 | 0.43 | 0.45 |
| G7 | 0.44 | 0.44 | 0.44 | 0.44 | 0.46 | 0.13 | 0 | 0.41 | 0.41 | 0.41 | 0.44 | 0.43 | 0.43 | 0.41 | 0.45 | 0.41 | 0.41 | 0.41 | **0.41** | 0.41 | 0.42 | 0.44 |
| G8 | 0.45 | 0.45 | 0.45 | 0.45 | 0.46 | 0.41 | 0.41 | 0 | 0.01 | 0.01 | 0.45 | 0.42 | 0.42 | 0.4 | 0.45 | 0.04 | 0.03 | 0.02 | **0.02** | 0.03 | 0.41 | 0.45 |
| G9 | 0.45 | 0.45 | 0.45 | 0.45 | 0.46 | 0.41 | 0.41 | 0.01 | 0 | 0 | 0.45 | 0.42 | 0.42 | 0.4 | 0.45 | 0.05 | 0.03 | 0.03 | **0.02** | 0.03 | 0.41 | 0.45 |
| G10 | 0.45 | 0.45 | 0.45 | 0.45 | 0.46 | 0.41 | 0.41 | 0.01 | 0 | 0 | 0.45 | 0.42 | 0.42 | 0.4 | 0.45 | 0.05 | 0.03 | 0.02 | **0.02** | 0.03 | 0.41 | 0.45 |
| G11 | 0.4 | 0.4 | 0.4 | 0.08 | 0.46 | 0.45 | 0.44 | 0.45 | 0.45 | 0.45 | 0 | 0.46 | 0.46 | 0.45 | 0.43 | 0.45 | 0.45 | 0.45 | **0.45** | 0.45 | 0.46 | 0.35 |
| G12 | 0.46 | 0.46 | 0.46 | 0.46 | 0.47 | 0.44 | 0.43 | 0.42 | 0.42 | 0.42 | 0.46 | 0 | 0 | 0.44 | 0.46 | 0.42 | 0.42 | 0.42 | **0.42** | 0.42 | 0.44 | 0.46 |
| G13 | 0.46 | 0.46 | 0.46 | 0.46 | 0.47 | 0.44 | 0.43 | 0.42 | 0.42 | 0.42 | 0.46 | 0 | 0 | 0.44 | 0.46 | 0.42 | 0.42 | 0.42 | **0.42** | 0.42 | 0.44 | 0.46 |
| G14 | 0.45 | 0.45 | 0.45 | 0.45 | 0.46 | 0.42 | 0.41 | 0.4 | 0.4 | 0.4 | 0.45 | 0.44 | 0.44 | 0 | 0.45 | 0.4 | 0.4 | 0.4 | **0.4** | 0.4 | 0.23 | 0.45 |
| G15 | 0.42 | 0.42 | 0.42 | 0.43 | 0.46 | 0.45 | 0.45 | 0.45 | 0.45 | 0.45 | 0.43 | 0.46 | 0.46 | 0.45 | 0 | 0.45 | 0.45 | 0.45 | **0.45** | 0.45 | 0.46 | 0.42 |
| G16 | 0.45 | 0.45 | 0.45 | 0.45 | 0.46 | 0.42 | 0.41 | 0.04 | 0.05 | 0.05 | 0.45 | 0.42 | 0.42 | 0.4 | 0.45 | 0 | 0.03 | 0.03 | **0.04** | 0.03 | 0.41 | 0.45 |
| G17 | 0.45 | 0.45 | 0.45 | 0.45 | 0.46 | 0.41 | 0.41 | 0.03 | 0.03 | 0.03 | 0.45 | 0.42 | 0.42 | 0.4 | 0.45 | 0.03 | 0 | 0.01 | **0.01** | 0.01 | 0.41 | 0.45 |
| G18 | 0.45 | 0.45 | 0.45 | 0.45 | 0.46 | 0.41 | 0.41 | 0.02 | 0.03 | 0.02 | 0.45 | 0.42 | 0.42 | 0.4 | 0.45 | 0.03 | 0.01 | 0 | **0.01** | 0.02 | 0.41 | 0.45 |
| **G19** | **0.45** | **0.45** | **0.45** | **0.45** | **0.46** | **0.41** | **0.41** | **0.02** | **0.02** | **0.02** | **0.45** | **0.42** | **0.42** | **0.4** | **0.45** | **0.04** | **0.01** | **0.01** | **0** | **0.02** | **0.41** | **0.45** |
| G20 | 0.45 | 0.45 | 0.45 | 0.45 | 0.46 | 0.41 | 0.41 | 0.03 | 0.03 | 0.03 | 0.45 | 0.42 | 0.42 | 0.4 | 0.45 | 0.03 | 0.01 | 0.02 | **0.02** | 0 | 0.41 | 0.45 |
| G21 | 0.46 | 0.46 | 0.46 | 0.46 | 0.47 | 0.43 | 0.42 | 0.41 | 0.41 | 0.41 | 0.46 | 0.44 | 0.44 | 0.23 | 0.46 | 0.41 | 0.41 | 0.41 | **0.41** | 0.41 | 0 | 0.46 |
| G22 | 0.41 | 0.4 | 0.4 | 0.35 | 0.46 | 0.45 | 0.44 | 0.45 | 0.45 | 0.45 | 0.35 | 0.46 | 0.46 | 0.45 | 0.42 | 0.45 | 0.45 | 0.45 | **0.45** | 0.45 | 0.46 | 0 |

G1=Mycobacterium sp. JLS

G2=Mycobacterium sp. KMS

G3=Mycobacteriumsp. MCS

G4=Mycobacterium sp. Spyr1

G5=Mycobacterium abscessus ATCC 19977

G6=Mycobacterium avium

G7=Mycobacterium avium subsp. paratuberculosis K-10

G8=Mycobacterium bovis AF2122/97

G9=M.bovis strain Pasteur 1173P2

G10=Mycobacterium bovis BCG str. Tokyo 172

G11=Mycobacterium gilvum PYR-GCK

G12=Mycobacterium leprae

G13=Mycobacterium leprae TN

G14=Mycobacterium marinum M

G15=Mycobacterium smegmatis str. MC2 155

G16=Mycobacterium tuberculosis CDC1551

G17=Mycobacterium tuberculosis F11

G18=Mycobacterium tuberculosis H37Ra

G19=Mycobacterium tuberculosis H37Rv

G20=Mycobacterium tuberculosis KZN 1435

G21=Mycobacterium ulcerans Agy99

G22=Mycobacterium vanbaalenii PYR-1
