# Supplementary material for: A Genome-Wide Identification Analysis of Small Regulatory RNAs in Mycobacterium tuberculosis by RNA-Seq and Conservation Analysis
Source: PLoS One. 2012 Mar 28;7(3):e32723. doi: 10.1371/journal.pone.0032723 (PMC3314655; doi:10.1371/journal.pone.0032723)
Supplement: Table S2 — Complete list of identified sRNAs. Table contains information regarding the whole list of candidate sRNAs identified. (DOC) [file pone.0032723.s003.doc]

| **id** | **strand** | **type** | **length** | **meanExpr** | **meanCons** | **posRel** | **mfe** | **mfePvalue** |
| --- | --- | --- | --- | --- | --- | --- | --- | --- |
| candidate_1 | + | A | 48 | 202.68 | 0.01 | 5'/3'-UTR | -58.2 | 0.112 |
| candidate_2 | + | A | 111 | 171.99 | 0 | AntiSense | -48.1 | 0.516 |
| candidate_3 | + | A | 115 | 520.35 | 0 | AntiSense | -55.3 | 0.28 |
| candidate_4 | + | A | 407 | 443.58 | 0 | AntiSense | -152.17 | 0.903 |
| candidate_5 | + | A | 493 | 1091.69 | 0 | AntiSense | -224.4 | 0.265 |
| candidate_6 | + | A | 88 | 414.12 | 0.44 | 5'/3'-UTR | -21 | 0.701 |
| candidate_7 | + | A | 38 | 76.21 | 0 | AntiSense | -9.5 | 0.504 |
| candidate_8 | + | A | 79 | 260.65 | 0.03 | 5'/3'-UTR | -22 | 0.2 |
| candidate_9 | + | A | 120 | 195.9 | 0 | AntiSense | -36.7 | 0.993 |
| candidate_10 | + | A | 30 | 117.35 | 0 | AntiSense | -7.3 | 0.62 |
| candidate_11 | + | A | 33 | 726.82 | 0.11 | NonCoding | 0 | 1 |
| candidate_12 | + | A | 70 | 182.92 | 0 | AntiSense | -7.34 | 0.997 |
| candidate_13 | + | A | 132 | 308.86 | 0.01 | 5'/3'-UTR | -46.6 | 0.21 |
| candidate_14 | + | A | 76 | 468.36 | 0.05 | NonCoding | -19.7 | 0.376 |
| candidate_15 | + | A | 37 | 93.89 | 0.13 | NonCoding | -9.1 | 0.203 |
| candidate_16 | + | A | 30 | 271.58 | 0.14 | NonCoding | -3.4 | 0.606 |
| candidate_17 | + | A | 41 | 104.5 | 0 | AntiSense | -6.4 | 0.787 |
| candidate_18 | + | A | 100 | 551.73 | 0 | AntiSense | -38.7 | 0.105 |
| candidate_19 | + | A | 61 | 271.97 | 1.78 | NonCoding | -22.1 | 0.108 |
| candidate_20 | + | A | 50 | 181.37 | 0 | AntiSense | -10.1 | 0.858 |
| candidate_21 | + | A | 160 | 286.02 | 0 | AntiSense | -51.1 | 0.854 |
| candidate_22 | + | A | 52 | 95.87 | 0.12 | NonCoding | -18.6 | 0.12 |
| candidate_23 | + | A | 57 | 165.26 | 0.14 | NonCoding | -11.3 | 0.237 |
| candidate_24 | + | A | 141 | 662.08 | 0.07 | 5'/3'-UTR | -71.5 | 0.017 |
| candidate_25 | + | A | 49 | 237.42 | 0 | AntiSense | -5.4 | 1 |
| candidate_26 | + | A | 70 | 154.73 | 0 | AntiSense | -22.1 | 0.518 |
| candidate_27 | + | A | 259 | 1124.7 | 0.26 | 5'/3'-UTR | -88.8 | 0.825 |
| candidate_28 | + | A | 33 | 144.79 | 0 | AntiSense | -8.4 | 0.123 |
| candidate_29 | + | A | 58 | 616.32 | 1.91 | NonCoding | -32.7 | 0.001 |
| candidate_30 | + | A | 181 | 626.63 | 0 | AntiSense | -74.6 | 0.5 |
| candidate_31 | + | A | 37 | 64.42 | 0 | AntiSense | -7.1 | 0.823 |
| candidate_32 | + | A | 30 | 67.23 | 0 | AntiSense | -7.3 | 0.376 |
| candidate_33 | + | A | 134 | 547.81 | 0 | AntiSense | -38.7 | 0.885 |
| candidate_34 | + | A | 189 | 476.87 | 0 | AntiSense | -72.8 | 0.268 |
| candidate_35 | + | A | 71 | 240.86 | 0 | AntiSense | -27.6 | 0.084\ |
| candidate_36 | + | A | 44 | 219.53 | 0 | AntiSense | -13.3 | 0.105 |
| candidate_37 | + | A | 83 | 154.46 | 0 | AntiSense | -25.7 | 0.299 |
| candidate_38 | + | A | 106 | 212.69 | 0 | AntiSense | -30.3 | 0.693 |
| candidate_39 | + | A | 67 | 219.81 | 0.02 | 5'/3'-UTR | -17.7 | 0.499 |
| candidate_40 | + | A | 34 | 131.74 | 0 | AntiSense | -7.5 | 0.343 |
| candidate_41 | + | A | 49 | 757.24 | 0.1 | 5'/3'-UTR | -9.4 | 0.602 |
| candidate_42 | + | A | 43 | 414.98 | 0.09 | NonCoding | -7.4 | 0.944 |
| candidate_43 | + | A | 131 | 670.05 | 0.27 | NonCoding | -40.4 | 0.365 |
| candidate_44 | + | A | 32 | 224.94 | 0.5 | NonCoding | 0 | 1 |
| candidate_45 | + | A | 51 | 100.48 | 0 | AntiSense | -12.8 | 0.883 |
| candidate_46 | + | A | 41 | 132.69 | 0 | AntiSense | -6.4 | 0.699 |
| candidate_47 | + | A | 36 | 129.68 | 0 | AntiSense | -11.1 | 0.388 |
| candidate_48 | + | A | 30 | 113 | 0 | AntiSense | -2.9 | 0.951 |
| candidate_49 | + | A | 122 | 542.27 | 0.56 | NonCoding | -45.6 | 0.003 |
| candidate_50 | + | A | 50 | 133.57 | 0.06 | 5'/3'-UTR | -9 | 0.944 |
| candidate_51 | + | A | 31 | 133.5 | 0 | AntiSense | -6.6 | 0.246 |
| candidate_52 | + | A | 37 | 249.08 | 0 | AntiSense | -5.3 | 0.65 |
| candidate_53 | + | A | 31 | 71.38 | 0 | AntiSense | -3.9 | 0.823 |
| candidate_54 | + | A | 54 | 381.27 | 0 | AntiSense | -17.7 | 0.217 |
| candidate_55 | + | A | 50 | 212.86 | 0 | AntiSense | -15.6 | 0.337 |
| candidate_56 | + | A | 31 | 320.44 | 0 | AntiSense | -0.81 | 0.926 |
| candidate_57 | + | A | 35 | 164.97 | 0 | AntiSense | -8.2 | 0.819 |
| candidate_58 | + | A | 40 | 105.54 | 0 | AntiSense | -6.3 | 0.626 |
| candidate_59 | + | A | 50 | 331.35 | 0 | AntiSense | -5.7 | 0.704 |
| candidate_60 | + | A | 70 | 140.63 | 0.07 | NonCoding | -15 | 0.131 |
| candidate_61 | + | A | 39 | 168.03 | 0 | AntiSense | -7.1 | 0.748 |
| candidate_62 | + | A | 49 | 110.54 | 0 | AntiSense | -13.7 | 0.943 |
| candidate_63 | + | A | 129 | 183.8 | 0 | AntiSense | -44 | 0.874 |
| candidate_64 | + | A | 53 | 294.13 | 0 | AntiSense | -7.2 | 0.79 |
| candidate_65 | + | A | 36 | 112.19 | 0 | AntiSense | -1.7 | 0.969 |
| candidate_66 | + | A | 39 | 287.95 | 0 | AntiSense | -6.1 | 0.877 |
| candidate_67 | + | A | 135 | 1688.89 | 0 | 5'/3'-UTR | -52.1 | 0.177 |
| candidate_68 | + | A | 89 | 182.92 | 0 | AntiSense | -30.1 | 0.359 |
| candidate_69 | + | A | 88 | 121.92 | 0 | AntiSense | -36.8 | 0.323 |
| candidate_70 | + | A | 46 | 222.7 | 0 | AntiSense | -11.6 | 0.258 |
| candidate_71 | + | A | 38 | 119.95 | 0 | AntiSense | -5.8 | 0.959 |
| candidate_72 | + | A | 36 | 714.89 | 0.14 | NonCoding | -2.4 | 0.866 |
| candidate_73 | + | A | 38 | 71.87 | 0.12 | NonCoding | -11.4 | 0.459 |
| candidate_74 | + | A | 40 | 491.68 | 0.12 | NonCoding | -2.1 | 0.5 |
| candidate_75 | + | A | 142 | 917.8 | 1.36 | NonCoding | -57.5 | 0.123 |
| candidate_76 | + | A | 98 | 139.24 | 0.52 | NonCoding | -33.1 | 0.668 |
| candidate_77 | + | A | 53 | 375.31 | 0 | AntiSense | -7.2 | 0.971 |
| candidate_78 | + | A | 32 | 231.79 | 0 | AntiSense | -10.2 | 0.028 |
| candidate_79 | + | A | 84 | 177.48 | 0.14 | NonCoding | -29.9 | 0.126 |
| candidate_80 | + | A | 31 | 201.28 | 0.14 | NonCoding | -5.4 | 0.982 |
| candidate_81 | + | A | 36 | 254.16 | 0.09 | NonCoding | -8.3 | 0.028 |
| candidate_82 | + | A | 164 | 196.21 | 0 | AntiSense | -64.21 | 0.305 |
| candidate_83 | + | A | 158 | 309.28 | 0 | AntiSense | -66.4 | 0.317 |
| candidate_84 | + | A | 59 | 3648.93 | 0.13 | NonCoding | -38 | 0.001 |
| candidate_85 | + | A | 142 | 367.85 | 0 | AntiSense | -51.9 | 0.471 |
| candidate_86 | + | A | 31 | 102.38 | 0 | AntiSense | -6.1 | 0.859 |
| candidate_87 | + | A | 36 | 2368.03 | 0.12 | NonCoding | -2.9 | 0.445 |
| candidate_88 | + | A | 55 | 80.91 | 0.69 | NonCoding | -14.5 | 0.605 |
| candidate_89 | + | A | 32 | 547.76 | 0.13 | NonCoding | -2.3 | 0.803 |
| candidate_90 | + | A | 34 | 118.8 | 0 | AntiSense | -5.5 | 0.911 |
| candidate_91 | + | A | 51 | 457.33 | 0 | AntiSense | -19 | 0.039 |
| candidate_92 | + | A | 36 | 116.95 | 0 | AntiSense | -2.8 | 0.797 |
| candidate_93 | + | A | 53 | 152.87 | 0 | AntiSense | -10.3 | 0.782 |
| candidate_94 | + | A | 35 | 117.31 | 0.07 | NonCoding | -2.3 | 0.645 |
| candidate_95 | + | A | 35 | 284.72 | 1.57 | NonCoding | -23.2 | 0.001 |
| candidate_96 | + | A | 65 | 181.5 | 4.42 | NonCoding | -12.4 | 0.649 |
| candidate_97 | + | A | 43 | 241.18 | 0.38 | NonCoding | -9.7 | 0.446 |
| candidate_98 | + | A | 62 | 165.11 | 1.69 | NonCoding | -43.2 | 0.001 |
| candidate_99 | + | A | 37 | 951.24 | 0.57 | NonCoding | -6.4 | 0.797 |
| candidate_100 | + | A | 43 | 130.41 | 0.13 | NonCoding | -1.1 | 0.966 |
| candidate_101 | + | A | 46 | 164.83 | 1.18 | NonCoding | -4.9 | 0.84 |
| candidate_102 | + | A | 132 | 954.93 | 0.31 | NonCoding | -43.4 | 0.518 |
| candidate_103 | + | A | 33 | 157.35 | 0.14 | NonCoding | -28.1 | 0.001 |
| candidate_104 | + | A | 34 | 231.49 | 0.29 | NonCoding | -3.6 | 0.542 |
| candidate_105 | + | A | 30 | 1645.26 | 0.66 | NonCoding | 0 | 1 |
| candidate_106 | + | A | 33 | 92.79 | 1.42 | NonCoding | -26.4 | 0.001 |
| candidate_107 | + | A | 34 | 74.23 | 0 | AntiSense | -7.5 | 0.709 |
| candidate_108 | + | A | 36 | 148.49 | 0 | AntiSense | -1.2 | 0.742 |
| candidate_109 | + | A | 40 | 161.41 | 0 | AntiSense | -6.4 | 0.926 |
| candidate_110 | + | A | 46 | 170.7 | 0.03 | 5'/3'-UTR | -11.5 | 0.381 |
| candidate_111 | + | A | 32 | 695.91 | 0 | AntiSense | -10 | 0.419 |
| candidate_112 | + | A | 60 | 511.77 | 0.65 | NonCoding | -9.6 | 0.397 |
| candidate_113 | + | A | 32 | 99.97 | 0 | AntiSense | -5.4 | 0.396 |
| candidate_114 | + | A | 44 | 291.49 | 0 | AntiSense | -14.9 | 0.3 |
| candidate_115 | + | A | 36 | 152.86 | 0 | AntiSense | -3.5 | 0.358 |
| candidate_116 | + | A | 50 | 123 | 0 | AntiSense | -11.4 | 0.885 |
| candidate_117 | + | A | 41 | 252.95 | 0 | AntiSense | -10.2 | 0.344 |
| candidate_118 | + | A | 37 | 772.84 | 0.14 | NonCoding | -8.7 | 0.14 |
| candidate_119 | + | A | 216 | 657.71 | 2.36 | NonCoding | -92.6 | 0.277 |
| candidate_120 | + | A | 40 | 156.29 | 0 | AntiSense | -11 | 0.171 |
| candidate_121 | + | A | 33 | 250.06 | 0 | AntiSense | -6.9 | 0.488 |
| candidate_122 | + | A | 44 | 432.38 | 0.5 | NonCoding | -7 | 0.727 |
| candidate_123 | + | A | 87 | 445.27 | 0 | AntiSense | -24.06 | 0.819 |
| candidate_124 | + | A | 125 | 237.63 | 0.13 | NonCoding | -54.36 | 0.012 |
| candidate_125 | + | A | 66 | 180.12 | 0 | AntiSense | -18.5 | 0.717 |
| candidate_126 | + | A | 38 | 158.62 | 2.77 | NonCoding | -29.1 | 0.001 |
| candidate_127 | + | A | 31 | 474.78 | 0 | AntiSense | -1.9 | 0.687 |
| candidate_128 | + | A | 65 | 265.44 | 0.62 | 5'/3'-UTR | -14.5 | 0.767 |
| candidate_129 | + | A | 110 | 358.3 | 0.14 | NonCoding | -30.9 | 0.601 |
| candidate_130 | + | A | 32 | 288.03 | 0.13 | NonCoding | -6.1 | 0.746 |
| candidate_131 | + | A | 47 | 146.56 | 0.04 | 5'/3'-UTR | -11 | 0.429 |
| candidate_132 | + | A | 48 | 309.69 | 0 | AntiSense | -12.8 | 0.862 |
| candidate_133 | + | A | 44 | 93 | 0 | AntiSense | -6 | 0.784 |
| candidate_134 | + | A | 35 | 80.56 | 0 | AntiSense | -10.3 | 0.17 |
| candidate_135 | + | A | 293 | 1264.79 | 0 | AntiSense | -133.01 | 0.03 |
| candidate_136 | + | A | 33 | 150.09 | 0.03 | 5'/3'-UTR | -6.8 | 0.758 |
| candidate_137 | + | A | 149 | 803.31 | 0.76 | 5'/3'-UTR | -47.5 | 0.609 |
| candidate_138 | + | A | 72 | 515.92 | 0 | AntiSense | -10.6 | 0.671 |
| candidate_139 | + | A | 47 | 840.9 | 2.28 | NonCoding | -5.1 | 0.883 |
| candidate_140 | + | A | 120 | 761.75 | 0.51 | NonCoding | -38.8 | 0.327 |
| candidate_141 | + | A | 32 | 136.21 | 1.58 | NonCoding | -17.5 | 0.001 |
| candidate_142 | + | A | 39 | 405.75 | 0.13 | NonCoding | -7.2 | 0.678 |
| candidate_143 | + | A | 44 | 118.31 | 0 | AntiSense | -13 | 0.319 |
| candidate_144 | + | A | 35 | 5996 | 0 | AntiSense | -4.4 | 0.73 |
| candidate_145 | + | A | 39 | 327.8 | 0 | 5'/3'-UTR | -1.7 | 0.931 |
| candidate_146 | + | A | 32 | 110.91 | 0 | AntiSense | -1.7 | 0.822 |
| candidate_147 | + | A | 102 | 197.32 | 0.87 | NonCoding | -30.4 | 0.82 |
| candidate_148 | + | A | 82 | 324.92 | 0.23 | NonCoding | -44.63 | 0.001 |
| candidate_149 | + | A | 50 | 4366.27 | 1.33 | NonCoding | -10.6 | 0.744 |
| candidate_150 | + | A | 32 | 199.03 | 2 | NonCoding | -22.2 | 0.001 |
| candidate_151 | + | A | 31 | 87.75 | 0 | AntiSense | -2.2 | 0.644 |
| candidate_152 | + | A | 33 | 107.29 | 0 | AntiSense | -11.1 | 0.169 |
| candidate_153 | + | A | 176 | 119.95 | 0 | 5'/3'-UTR | -69.6 | 0.737 |
| candidate_154 | + | A | 42 | 89.28 | 0 | AntiSense | -9.9 | 0.95 |
| candidate_155 | + | A | 60 | 129.79 | 0.05 | 5'/3'-UTR | -12.5 | 0.984 |
| candidate_156 | + | A | 30 | 143.65 | 3.52 | NonCoding | -7 | 0.28 |
| candidate_157 | + | A | 44 | 138.8 | 0.09 | NonCoding | -14.3 | 0.024 |
| candidate_158 | + | A | 53 | 129.94 | 0.1 | 5'/3'-UTR | -16.6 | 0.09 |
| candidate_159 | + | A | 77 | 224.97 | 0.18 | NonCoding | -31.7 | 0.034 |
| candidate_160 | + | A | 32 | 186.45 | 0.18 | NonCoding | -6.8 | 0.651 |
| candidate_161 | + | A | 75 | 138.17 | 0.14 | NonCoding | -22.6 | 0.893 |
| candidate_162 | + | A | 39 | 77.58 | 0 | AntiSense | -14.7 | 0.299 |
| candidate_163 | + | A | 57 | 894.05 | 0.14 | NonCoding | -11.7 | 0.825 |
| candidate_164 | + | A | 30 | 791.58 | 2.79 | NonCoding | -4.8 | 0.587 |
| candidate_165 | + | A | 40 | 816.22 | 0.14 | NonCoding | -14.2 | 0.413 |
| candidate_166 | + | A | 37 | 165.34 | 0 | AntiSense | -11.2 | 0.261 |
| candidate_167 | + | A | 81 | 1006.95 | 0.11 | NonCoding | -21.9 | 0.908 |
| candidate_168 | + | A | 127 | 660.12 | 0 | AntiSense | -48.3 | 0.478 |
| candidate_169 | + | A | 59 | 1025.9 | 0 | AntiSense | -16.7 | 0.178 |
| candidate_170 | + | A | 33 | 97.18 | 0 | AntiSense | -6.4 | 0.573 |
| candidate_171 | + | A | 128 | 2457.25 | 0 | AntiSense | -40.5 | 0.758 |
| candidate_172 | + | A | 30 | 180.29 | 0 | AntiSense | -8.6 | 0.374 |
| candidate_173 | + | A | 313 | 577.02 | 0 | AntiSense | -121.3 | 0.39 |
| candidate_174 | + | A | 56 | 258.89 | 0.14 | NonCoding | -16.8 | 0.393 |
| candidate_175 | + | A | 37 | 244.84 | 0.14 | NonCoding | -8.1 | 0.643 |
| candidate_176 | + | A | 40 | 454.17 | 0.14 | NonCoding | -10.8 | 0.688 |
| candidate_177 | + | A | 40 | 171.46 | 0 | AntiSense | -11.1 | 0.263 |
| candidate_178 | + | A | 72 | 203.23 | 0 | AntiSense | -20.1 | 0.789 |
| candidate_179 | + | A | 121 | 146.84 | 0.01 | 5'/3'-UTR | -40.7 | 0.086 |
| candidate_180 | + | A | 192 | 565.71 | 0 | AntiSense | -78.6 | 0.85 |
| candidate_181 | + | A | 91 | 159.9 | 0 | AntiSense | -33.6 | 0.728 |
| candidate_182 | + | A | 44 | 510.31 | 0 | AntiSense | -13.3 | 0.591 |
| candidate_183 | + | A | 33 | 237.38 | 0 | AntiSense | -15.4 | 0.106 |
| candidate_184 | + | A | 37 | 230.87 | 0 | AntiSense | -12.8 | 0.175 |
| candidate_185 | + | A | 36 | 197.65 | 1.25 | NonCoding | -6.9 | 0.643 |
| candidate_186 | + | A | 44 | 170.87 | 0 | AntiSense | -15.7 | 0.525 |
| candidate_187 | + | A | 41 | 119.26 | 0 | AntiSense | -6.7 | 0.863 |
| candidate_188 | + | A | 31 | 219.97 | 0 | AntiSense | -15.9 | 0.018 |
| candidate_189 | + | A | 53 | 217.13 | 2.2 | NonCoding | -13.8 | 0.123 |
| candidate_190 | + | A | 80 | 23731.14 | 3.03 | NonCoding | -25.8 | 0.038 |
| candidate_191 | + | A | 63 | 574.02 | 0.14 | NonCoding | -15.7 | 0.064 |
| candidate_192 | + | A | 187 | 1461.84 | 2.95 | NonCoding | -79.7 | 0.001 |
| candidate_193 | + | A | 59 | 454.95 | 0.37 | NonCoding | -10.5 | 0.111 |
| candidate_194 | + | A | 31 | 171.5 | 0 | AntiSense | -5.4 | 0.47 |
| candidate_195 | + | A | 147 | 1195.24 | 0 | 5'/3'-UTR | -63 | 0.003 |
| candidate_196 | + | A | 37 | 599.87 | 1.7 | NonCoding | -27.5 | 0.001 |
| candidate_197 | + | A | 45 | 345.22 | 0 | AntiSense | -7.7 | 0.622 |
| candidate_198 | + | A | 76 | 139.52 | 0 | AntiSense | -21.6 | 0.678 |
| candidate_199 | + | A | 35 | 131.78 | 0 | AntiSense | -4.5 | 0.797 |
| candidate_200 | + | A | 30 | 107.23 | 0 | AntiSense | -4.2 | 0.975 |
| candidate_201 | + | A | 30 | 105.97 | 0 | AntiSense | -1.6 | 0.981 |
| candidate_202 | + | A | 112 | 398.12 | 0.51 | NonCoding | -22.3 | 0.997 |
| candidate_203 | + | A | 34 | 80.29 | 0.02 | 5'/3'-UTR | -7.9 | 0.473 |
| candidate_204 | + | A | 43 | 484.43 | 0.14 | NonCoding | -9.4 | 0.32 |
| candidate_205 | + | A | 64 | 919.22 | 0.14 | NonCoding | -12.2 | 0.953 |
| candidate_206 | + | A | 56 | 272.32 | 0.1 | NonCoding | -8.1 | 0.946 |
| candidate_207 | + | A | 34 | 688.91 | 1.67 | NonCoding | -27.8 | 0.001 |
| candidate_208 | + | A | 34 | 242.89 | 0 | AntiSense | -6.7 | 0.491 |
| candidate_209 | + | A | 40 | 71.07 | 0 | AntiSense | -14.2 | 0.133 |
| candidate_210 | + | A | 36 | 519.54 | 0 | AntiSense | -7.6 | 0.274 |
| candidate_211 | + | A | 45 | 98.22 | 0 | AntiSense | -13.6 | 0.537 |
| candidate_212 | + | A | 45 | 119.17 | 0 | AntiSense | -11.4 | 0.503 |
| candidate_213 | + | A | 31 | 208.06 | 0 | AntiSense | -4.9 | 0.643 |
| candidate_214 | + | A | 44 | 164.22 | 0 | AntiSense | -11.9 | 0.553 |
| candidate_215 | + | A | 62 | 318.33 | 0 | AntiSense | -20.7 | 0.464 |
| candidate_216 | + | A | 31 | 377.63 | 0.13 | NonCoding | -7.7 | 0.602 |
| candidate_217 | + | A | 39 | 150.48 | 0 | AntiSense | -9.2 | 0.714 |
| candidate_218 | + | A | 35 | 74.11 | 0.27 | NonCoding | -1.2 | 0.837 |
| candidate_219 | + | A | 42 | 146.07 | 0 | AntiSense | -10.3 | 0.457 |
| candidate_220 | + | A | 31 | 106.91 | 0 | AntiSense | -4.9 | 0.818 |
| candidate_221 | + | A | 118 | 202.88 | 0 | AntiSense | -38.2 | 0.647 |
| candidate_222 | + | A | 32 | 197.09 | 0 | AntiSense | -5.4 | 0.718 |
| candidate_223 | + | A | 42 | 146.28 | 0 | AntiSense | -9.6 | 0.811 |
| candidate_224 | + | A | 54 | 107.47 | 0.25 | NonCoding | -17.2 | 0.131 |
| candidate_225 | + | A | 126 | 192.41 | 0.33 | NonCoding | -45.7 | 0.023 |
| candidate_226 | + | A | 49 | 540.22 | 0 | AntiSense | -6.8 | 0.302 |
| candidate_227 | + | A | 59 | 86.18 | 0.06 | 5'/3'-UTR | -9.6 | 0.491 |
| candidate_228 | + | A | 32 | 476.09 | 0 | AntiSense | -21.8 | 0.008 |
| candidate_229 | + | A | 124 | 371.33 | 0.13 | NonCoding | -50.4 | 0.418 |
| candidate_230 | + | A | 65 | 761.42 | 0.03 | 5'/3'-UTR | -14.9 | 0.332 |
| candidate_231 | + | A | 51 | 174.81 | 0 | AntiSense | -9.24 | 0.655 |
| candidate_232 | + | A | 92 | 167.72 | 0.09 | 5'/3'-UTR | -27.8 | 0.278 |
| candidate_233 | + | A | 57 | 230.31 | 0 | AntiSense | -6.8 | 0.99 |
| candidate_234 | + | A | 35 | 167.39 | 1.27 | NonCoding | -22.5 | 0.001 |
| candidate_235 | + | A | 49 | 3771.96 | 2.55 | NonCoding | -4.9 | 0.301 |
| candidate_236 | + | A | 40 | 217.95 | 1.19 | NonCoding | -14.5 | 0.097 |
| candidate_237 | + | A | 31 | 764.59 | 0 | AntiSense | -15.1 | 0.008 |
| candidate_238 | + | A | 49 | 246.72 | 0.13 | NonCoding | -7.4 | 0.255 |
| candidate_239 | + | A | 35 | 259 | 0.83 | NonCoding | -5.8 | 0.297 |
| candidate_240 | + | A | 42 | 359.47 | 0.14 | NonCoding | -4.5 | 0.827 |
| candidate_241 | + | A | 102 | 146.69 | 0 | AntiSense | -46.1 | 0.172 |
| candidate_242 | + | A | 30 | 128.42 | 0 | AntiSense | -2.3 | 0.677 |
| candidate_243 | + | A | 180 | 382.83 | 0.14 | NonCoding | -75.16 | 0.176 |
| candidate_244 | + | A | 161 | 8388.07 | 0.07 | 5'/3'-UTR | -80.5 | 0.001 |
| candidate_245 | + | A | 30 | 71.42 | 0 | AntiSense | -1.3 | 0.999 |
| candidate_246 | + | A | 67 | 88.26 | 0.14 | NonCoding | -21.1 | 0.16 |
| candidate_247 | + | A | 120 | 111.88 | 0 | AntiSense | -42.2 | 0.648 |
| candidate_248 | + | A | 42 | 171 | 0.07 | NonCoding | -8 | 0.563 |
| candidate_249 | + | A | 111 | 634.06 | 0 | 5'/3'-UTR | -42.2 | 0.055 |
| candidate_250 | + | A | 40 | 154.63 | 0 | AntiSense | -8.9 | 0.749 |
| candidate_251 | + | A | 40 | 171.49 | 0 | AntiSense | -9.1 | 0.474 |
| candidate_252 | + | A | 45 | 201.76 | 0.11 | NonCoding | -11.3 | 0.859 |
| candidate_253 | + | A | 44 | 164.18 | 0.08 | 5'/3'-UTR | -11.1 | 0.574 |
| candidate_254 | + | A | 155 | 573.37 | 0 | AntiSense | -59.3 | 0.924 |
| candidate_255 | + | A | 41 | 177.81 | 0.14 | NonCoding | -9.2 | 0.638 |
| candidate_256 | + | A | 32 | 88.85 | 0.14 | NonCoding | -1.7 | 0.879 |
| candidate_257 | + | A | 51 | 1278.6 | 0.82 | NonCoding | -9.1 | 0.368 |
| candidate_258 | + | A | 86 | 536.48 | 1.16 | NonCoding | -38.8 | 0.002 |
| candidate_259 | + | A | 42 | 202.95 | 0.07 | 5'/3'-UTR | -3.2 | 0.894 |
| candidate_260 | + | A | 36 | 117.19 | 0.13 | NonCoding | -5.9 | 0.515 |
| candidate_261 | + | A | 62 | 278.92 | 0 | AntiSense | -19.6 | 0.52 |
| candidate_262 | + | A | 122 | 273.93 | 0.1 | 5'/3'-UTR | -38.69 | 0.599 |
| candidate_263 | + | A | 277 | 299.03 | 0.06 | 5'/3'-UTR | -119.9 | 0.109 |
| candidate_264 | + | A | 41 | 87.86 | 0.57 | NonCoding | -14.9 | 0.015 |
| candidate_265 | + | A | 37 | 167.47 | 0.6 | NonCoding | -7.8 | 0.879 |
| candidate_266 | + | A | 96 | 714.52 | 0.14 | NonCoding | -34.9 | 0.397 |
| candidate_267 | + | A | 95 | 261.64 | 0 | AntiSense | -30.3 | 0.688 |
| candidate_268 | + | A | 97 | 116.96 | 3.06 | NonCoding | -54.9 | 0.012 |
| candidate_269 | + | A | 35 | 89.92 | 0 | AntiSense | -8.3 | 0.357 |
| candidate_270 | + | A | 45 | 348.93 | 0 | AntiSense | -9.4 | 0.793 |
| candidate_271 | + | A | 30 | 100.39 | 0 | AntiSense | -5 | 0.834 |
| candidate_272 | + | A | 54 | 772.38 | 0 | AntiSense | -18.5 | 0.43 |
| candidate_273 | + | A | 130 | 1787.83 | 0.27 | NonCoding | -43.1 | 0.972 |
| candidate_274 | + | A | 31 | 97.13 | 0 | 5'/3'-UTR | -3.5 | 0.458 |
| candidate_275 | + | A | 32 | 126.27 | 0.14 | NonCoding | -4.9 | 0.216 |
| candidate_276 | + | A | 56 | 191.68 | 0 | AntiSense | -18.8 | 0.278 |
| candidate_277 | + | A | 73 | 992.39 | 0 | AntiSense | -27.2 | 0.227 |
| candidate_278 | + | A | 58 | 271.03 | 0 | AntiSense | -14.84 | 0.542 |
| candidate_279 | + | A | 40 | 234.39 | 0.11 | NonCoding | -7.46 | 0.221 |
| candidate_280 | + | A | 275 | 343 | 0 | AntiSense | -112.4 | 0.199 |
| candidate_281 | + | A | 64 | 166.72 | 0.28 | 5'/3'-UTR | -14.1 | 0.81 |
| candidate_282 | + | A | 54 | 79.71 | 0.08 | NonCoding | -16.1 | 0.154 |
| candidate_283 | + | A | 32 | 220.58 | 0.11 | NonCoding | -5.4 | 0.687 |
| candidate_284 | + | A | 30 | 287.19 | 0 | AntiSense | -3.6 | 0.758 |
| candidate_285 | + | A | 32 | 168.58 | 0.06 | NonCoding | -9.9 | 0.356 |
| candidate_286 | + | A | 40 | 237.27 | 0.14 | NonCoding | -6.62 | 0.725 |
| candidate_287 | + | A | 50 | 277.18 | 0 | AntiSense | -14.7 | 0.278 |
| candidate_288 | + | A | 31 | 1341.72 | 0 | AntiSense | -5.7 | 0.497 |
| candidate_289 | + | A | 74 | 886.36 | 0 | AntiSense | -15.4 | 0.331 |
| candidate_290 | + | A | 47 | 120.31 | 0 | AntiSense | -8.8 | 0.576 |
| candidate_291 | + | A | 48 | 268.14 | 0 | AntiSense | -9.49 | 0.59 |
| candidate_292 | + | A | 50 | 680.43 | 0.01 | 5'/3'-UTR | -3.2 | 0.431 |
| candidate_293 | + | A | 94 | 887.43 | 0 | 5'/3'-UTR | -25.7 | 0.158 |
| candidate_294 | + | A | 91 | 2390.95 | 0.12 | NonCoding | -38.7 | 0.006 |
| candidate_295 | + | A | 125 | 292.31 | 0 | AntiSense | -41.1 | 0.852 |
| candidate_296 | + | A | 195 | 555.28 | 0 | AntiSense | -77.1 | 0.288 |
| candidate_297 | + | A | 164 | 494.04 | 0 | AntiSense | -60.5 | 0.829 |
| candidate_298 | + | A | 81 | 424.77 | 0.14 | NonCoding | -22.1 | 0.926 |
| candidate_299 | + | A | 128 | 544.01 | 0.08 | NonCoding | -47.4 | 0.492 |
| candidate_300 | + | A | 161 | 425.88 | 0.1 | 5'/3'-UTR | -51.3 | 0.341 |
| candidate_301 | + | A | 49 | 402.58 | 0 | AntiSense | -12.2 | 0.785 |
| candidate_302 | + | A | 36 | 160.65 | 0 | AntiSense | -8.8 | 0.448 |
| candidate_303 | + | A | 40 | 226.93 | 0 | AntiSense | -11.1 | 0.338 |
| candidate_304 | + | A | 68 | 193.58 | 0 | AntiSense | -14.5 | 0.997 |
| candidate_305 | + | A | 40 | 175.07 | 0.14 | NonCoding | -8.9 | 0.902 |
| candidate_306 | + | A | 41 | 162.29 | 0 | AntiSense | -7.6 | 0.757 |
| candidate_307 | + | A | 39 | 103.5 | 0 | AntiSense | -12.5 | 0.375 |
| candidate_308 | + | A | 48 | 134.31 | 0 | AntiSense | -7.1 | 0.676 |
| candidate_309 | + | A | 39 | 180.13 | 0 | AntiSense | -8.4 | 0.527 |
| candidate_310 | + | A | 62 | 434.9 | 0 | AntiSense | -9 | 0.844 |
| candidate_311 | + | A | 43 | 103.34 | 0 | AntiSense | -5.1 | 0.957 |
| candidate_312 | + | A | 45 | 110.22 | 0 | AntiSense | -9.1 | 0.345 |
| candidate_313 | + | A | 89 | 416.26 | 3.39 | NonCoding | -19.8 | 0.458 |
| candidate_314 | + | A | 38 | 110.33 | 0.07 | 5'/3'-UTR | -4 | 0.897 |
| candidate_315 | + | A | 81 | 360.33 | 0 | AntiSense | -24 | 0.662 |
| candidate_316 | + | A | 54 | 81.24 | 3.08 | NonCoding | -11.4 | 0.752 |
| candidate_317 | + | A | 84 | 123.08 | 0 | AntiSense | -22.8 | 0.742 |
| candidate_318 | + | A | 56 | 115.74 | 1.08 | NonCoding | -15.4 | 0.443 |
| candidate_319 | + | A | 76 | 474.86 | 0.04 | 5'/3'-UTR | -13.8 | 0.665 |
| candidate_320 | + | A | 44 | 145.11 | 0 | AntiSense | -10.52 | 0.334 |
| candidate_321 | + | A | 32 | 164.55 | 0 | AntiSense | -6.2 | 0.519 |
| candidate_322 | + | A | 41 | 117.5 | 0.29 | NonCoding | -17.4 | 0.214 |
| candidate_323 | + | A | 76 | 120.58 | 0 | AntiSense | -25.1 | 0.256 |
| candidate_324 | + | A | 36 | 816.81 | 0.14 | NonCoding | -7 | 0.49 |
| candidate_325 | + | A | 30 | 94.84 | 1.82 | NonCoding | -3.8 | 0.775 |
| candidate_326 | + | A | 55 | 426.21 | 0 | AntiSense | -15.9 | 0.442 |
| candidate_327 | + | A | 40 | 73.95 | 0 | AntiSense | -6.6 | 0.803 |
| candidate_328 | + | A | 76 | 2606.95 | 0.89 | NonCoding | -9.77 | 0.982 |
| candidate_329 | + | A | 31 | 235.44 | 0.13 | NonCoding | -5.2 | 0.572 |
| candidate_330 | + | A | 54 | 190.91 | 2.01 | NonCoding | -24.4 | 0.047 |
| candidate_331 | + | A | 71 | 136.1 | 0 | AntiSense | -21 | 0.642 |
| candidate_332 | + | A | 30 | 520 | 0 | AntiSense | -9.4 | 0.143 |
| candidate_333 | + | A | 61 | 296.42 | 0.13 | NonCoding | -16.5 | 0.66 |
| candidate_334 | + | A | 71 | 484.81 | 0 | AntiSense | -24.7 | 0.116 |
| candidate_335 | + | A | 30 | 91.26 | 0 | AntiSense | -2.2 | 0.867 |
| candidate_336 | + | A | 36 | 428.41 | 0 | AntiSense | -6.7 | 0.748 |
| candidate_337 | + | A | 31 | 268.41 | 0.12 | NonCoding | -3.9 | 0.967 |
| candidate_338 | + | A | 77 | 318.86 | 0.12 | 5'/3'-UTR | -33.1 | 0.075 |
| candidate_339 | + | A | 107 | 751.08 | 0 | AntiSense | -27.01 | 0.545 |
| candidate_340 | + | A | 47 | 143.98 | 0 | AntiSense | -8.3 | 0.494 |
| candidate_341 | + | A | 64 | 517.42 | 0 | 5'/3'-UTR | -8.2 | 0.943 |
| candidate_342 | + | A | 30 | 126.32 | 0 | AntiSense | -7.4 | 0.107 |
| candidate_343 | + | A | 38 | 150.95 | 0.44 | NonCoding | -9.7 | 0.069 |
| candidate_344 | + | A | 31 | 117.31 | 0 | AntiSense | -6.6 | 0.515 |
| candidate_345 | + | A | 30 | 98.97 | 0 | AntiSense | -4.9 | 0.588 |
| candidate_346 | + | A | 36 | 83.03 | 0 | AntiSense | -12.8 | 0.154 |
| candidate_347 | + | A | 33 | 165.68 | 0 | AntiSense | -6.9 | 0.318 |
| candidate_348 | + | A | 44 | 240.22 | 0 | AntiSense | -7.4 | 0.204 |
| candidate_349 | + | A | 51 | 188.71 | 0.14 | NonCoding | -13.8 | 0.675 |
| candidate_350 | + | A | 69 | 244 | 0.12 | NonCoding | -19.5 | 0.644 |
| candidate_351 | + | A | 51 | 254.67 | 0.07 | NonCoding | -10.5 | 0.831 |
| candidate_352 | + | A | 31 | 119.84 | 0 | AntiSense | -6.4 | 0.363 |
| candidate_353 | + | A | 30 | 187.26 | 0.04 | 5'/3'-UTR | -3.7 | 0.957 |
| candidate_354 | + | A | 97 | 129.08 | 0 | 5'/3'-UTR | -40.8 | 0.159 |
| candidate_355 | + | A | 180 | 549.34 | 0 | AntiSense | -52.2 | 0.64 |
| candidate_356 | + | A | 39 | 241.6 | 0 | AntiSense | -8.5 | 0.493 |
| candidate_357 | + | A | 226 | 1642.32 | 0.02 | 5'/3'-UTR | -72.3 | 0.796 |
| candidate_358 | + | A | 111 | 472.37 | 0.06 | 5'/3'-UTR | -28.5 | 0.355 |
| candidate_359 | + | A | 60 | 310.72 | 0 | AntiSense | -15.4 | 0.747 |
| candidate_360 | + | A | 60 | 362.25 | 0 | AntiSense | -21.1 | 0.732 |
| candidate_361 | + | A | 33 | 187.32 | 0.14 | NonCoding | -3.4 | 0.894 |
| candidate_362 | + | A | 43 | 538.36 | 0 | AntiSense | -5.4 | 0.896 |
| candidate_363 | + | A | 116 | 304.61 | 0 | AntiSense | -36.73 | 0.599 |
| candidate_364 | + | A | 40 | 802.22 | 0 | AntiSense | -10.3 | 0.372 |
| candidate_365 | + | A | 30 | 127.9 | 0 | AntiSense | -8.2 | 0.228 |
| candidate_366 | + | A | 32 | 172.73 | 1.3 | NonCoding | -5.7 | 0.368 |
| candidate_367 | + | A | 44 | 104.18 | 0.08 | NonCoding | -11.1 | 0.79 |
| candidate_368 | + | A | 230 | 800.86 | 0 | 5'/3'-UTR | -78.6 | 0.693 |
| candidate_369 | + | A | 58 | 255.63 | 1.69 | 5'/3'-UTR | -11.8 | 0.759 |
| candidate_370 | + | A | 40 | 238.44 | 0 | AntiSense | -4.2 | 0.964 |
| candidate_371 | + | A | 34 | 1209.77 | 1.21 | NonCoding | -5.4 | 0.288 |
| candidate_372 | + | A | 64 | 1781.63 | 0.1 | NonCoding | -11.8 | 0.121 |
| candidate_373 | + | A | 30 | 303.19 | 0 | AntiSense | -1.6 | 0.986 |
| candidate_374 | + | A | 36 | 84.62 | 0 | AntiSense | -6.1 | 0.898 |
| candidate_375 | + | A | 83 | 277.55 | 0 | AntiSense | -19.53 | 0.664 |
| candidate_376 | + | A | 33 | 350.65 | 0 | AntiSense | -6 | 0.233 |
| candidate_377 | + | A | 106 | 426.31 | 0 | 5'/3'-UTR | -20.67 | 0.67 |
| candidate_378 | + | A | 38 | 710.82 | 0 | AntiSense | -10.2 | 0.228 |
| candidate_379 | + | A | 53 | 135.98 | 0 | AntiSense | -10.61 | 0.934 |
| candidate_380 | + | A | 41 | 72.33 | 0 | AntiSense | -7.1 | 0.591 |
| candidate_381 | + | A | 30 | 258.65 | 0 | AntiSense | 0 | 1 |
| candidate_382 | + | A | 41 | 243.24 | 0.1 | 5'/3'-UTR | -4.24 | 0.754 |
| candidate_383 | + | A | 31 | 147.78 | 0 | AntiSense | -10.4 | 0.323 |
| candidate_384 | + | A | 33 | 143.06 | 0 | AntiSense | -7.6 | 0.163 |
| candidate_385 | + | A | 30 | 77.87 | 0 | AntiSense | -8.7 | 0.036 |
| candidate_386 | + | A | 30 | 215.35 | 0.13 | NonCoding | -1.8 | 0.805 |
| candidate_387 | + | A | 45 | 120.13 | 0 | AntiSense | -7.1 | 0.986 |
| candidate_388 | + | A | 56 | 728.95 | 0 | AntiSense | -10.5 | 0.864 |
| candidate_389 | + | A | 118 | 402.28 | 0 | AntiSense | -41.9 | 0.137 |
| candidate_390 | + | A | 79 | 108.08 | 0 | AntiSense | -19.7 | 0.904 |
| candidate_391 | + | A | 59 | 114.97 | 0 | AntiSense | -11.5 | 0.794 |
| candidate_392 | + | A | 62 | 137.48 | 0 | AntiSense | -17.6 | 0.58 |
| candidate_393 | + | A | 91 | 180.53 | 0 | AntiSense | -34.9 | 0.137 |
| candidate_394 | + | A | 34 | 183.74 | 0 | AntiSense | -4.9 | 0.711 |
| candidate_395 | + | A | 54 | 263.6 | 0.14 | NonCoding | -14.6 | 0.573 |
| candidate_396 | + | A | 421 | 1109.4 | 0 | 5'/3'-UTR | -183 | 0.565 |
| candidate_397 | + | A | 37 | 109.47 | 0 | AntiSense | -10.8 | 0.289 |
| candidate_398 | + | A | 33 | 353.85 | 0 | AntiSense | -3.3 | 0.838 |
| candidate_399 | + | A | 66 | 271.69 | 0 | AntiSense | -27.8 | 0.355 |
| candidate_400 | + | A | 39 | 1559 | 0 | AntiSense | -6.6 | 0.078 |
| candidate_401 | + | A | 85 | 320.76 | 0 | AntiSense | -19.9 | 0.81 |
| candidate_402 | + | A | 127 | 492.16 | 0.14 | NonCoding | -32.4 | 0.662 |
| candidate_403 | + | A | 45 | 91.74 | 0.14 | NonCoding | -14.2 | 0.122 |
| candidate_404 | + | A | 38 | 124.31 | 0.13 | NonCoding | -3.2 | 0.575 |
| candidate_405 | + | A | 35 | 1619.22 | 0 | AntiSense | -21.8 | 0.013 |
| candidate_406 | + | A | 40 | 98.46 | 0 | AntiSense | -16.5 | 0.07 |
| candidate_407 | + | A | 164 | 146.76 | 0.02 | 5'/3'-UTR | -52.7 | 0.031 |
| candidate_408 | + | A | 56 | 139.74 | 0.09 | NonCoding | -11.5 | 0.48 |
| candidate_409 | + | A | 35 | 140.47 | 0 | AntiSense | -4.1 | 0.676 |
| candidate_410 | + | A | 51 | 147.19 | 0 | AntiSense | -18.7 | 0.136 |
| candidate_411 | + | A | 31 | 92.03 | 0.48 | NonCoding | -7.3 | 0.13 |
| candidate_412 | + | A | 54 | 134.11 | 2.72 | NonCoding | -4 | 0.953 |
| candidate_413 | + | A | 47 | 542.56 | 0 | AntiSense | -5.5 | 0.871 |
| candidate_414 | + | A | 31 | 137.78 | 0 | AntiSense | -9.8 | 0.277 |
| candidate_415 | + | A | 172 | 424.29 | 0 | AntiSense | -73.5 | 0.535 |
| candidate_416 | + | A | 36 | 194.22 | 0 | AntiSense | -1.49 | 0.963 |
| candidate_417 | + | A | 54 | 79.31 | 0 | AntiSense | -15.4 | 0.88 |
| candidate_418 | + | A | 58 | 297 | 0 | 5'/3'-UTR | -14.2 | 0.484 |
| candidate_419 | + | A | 54 | 221.29 | 0.3 | NonCoding | -14.2 | 0.715 |
| candidate_420 | + | A | 227 | 4836.48 | 0.01 | 5'/3'-UTR | -70 | 0.416 |
| candidate_421 | + | A | 31 | 85.06 | 0 | AntiSense | -2.81 | 0.975 |
| candidate_422 | + | A | 69 | 125.87 | 0 | AntiSense | -22.7 | 0.107 |
| candidate_423 | + | A | 47 | 161.46 | 0 | AntiSense | -9.6 | 0.669 |
| candidate_424 | + | A | 31 | 98.47 | 0 | AntiSense | -1.4 | 0.645 |
| candidate_425 | + | A | 33 | 158.74 | 0 | AntiSense | -7.4 | 0.612 |
| candidate_426 | + | A | 83 | 202.14 | 0 | AntiSense | -38.8 | 0.046 |
| candidate_427 | + | A | 30 | 351.58 | 0 | AntiSense | -13.8 | 0.009 |
| candidate_428 | + | A | 123 | 672.16 | 0 | AntiSense | -41 | 0.861 |
| candidate_429 | + | A | 39 | 160.95 | 0 | AntiSense | -11.8 | 0.203 |
| candidate_430 | + | A | 32 | 137.09 | 0 | 5'/3'-UTR | -3.9 | 0.588 |
| candidate_431 | + | A | 103 | 236.08 | 0 | AntiSense | -27.5 | 0.889 |
| candidate_432 | + | A | 149 | 384.28 | 0.14 | NonCoding | -46 | 0.719 |
| candidate_433 | + | A | 71 | 257.03 | 0 | AntiSense | -16 | 0.978 |
| candidate_434 | + | A | 40 | 137.56 | 0 | AntiSense | -4.9 | 0.82 |
| candidate_435 | + | A | 102 | 269.72 | 0.46 | NonCoding | -23.34 | 0.742 |
| candidate_436 | + | A | 41 | 632.67 | 0 | AntiSense | -3.69 | 0.857 |
| candidate_437 | + | A | 170 | 256.97 | 0 | AntiSense | -39.4 | 0.946 |
| candidate_438 | + | A | 91 | 226.78 | 0.01 | 5'/3'-UTR | -20.5 | 0.377 |
| candidate_439 | + | A | 46 | 159.79 | 0 | AntiSense | -9.6 | 0.872 |
| candidate_440 | + | A | 54 | 180.56 | 0 | AntiSense | -16.3 | 0.329 |
| candidate_441 | + | A | 50 | 76.75 | 0 | AntiSense | -12.6 | 0.502 |
| candidate_442 | + | A | 44 | 229.11 | 0.1 | NonCoding | -12.8 | 0.449 |
| candidate_443 | + | A | 117 | 167.19 | 0 | AntiSense | -29.8 | 0.8 |
| candidate_444 | + | A | 132 | 394.59 | 0.05 | 5'/3'-UTR | -31 | 0.157 |
| candidate_445 | + | A | 90 | 217.64 | 0.14 | NonCoding | -27.2 | 0.847 |
| candidate_446 | + | A | 31 | 247.22 | 0.47 | NonCoding | -3.9 | 0.639 |
| candidate_447 | + | A | 56 | 454.82 | 0.04 | 5'/3'-UTR | -6.5 | 0.691 |
| candidate_448 | + | A | 36 | 84.86 | 0 | AntiSense | -7.9 | 0.964 |
| candidate_449 | + | A | 30 | 111.29 | 0.09 | NonCoding | -7.1 | 0.791 |
| candidate_450 | + | A | 45 | 496.3 | 0.09 | NonCoding | -13.2 | 0.131 |
| candidate_451 | + | A | 86 | 95.84 | 2.26 | NonCoding | -31.1 | 0.357 |
| candidate_452 | + | A | 37 | 167.68 | 0 | AntiSense | -16.6 | 0.115 |
| candidate_453 | + | A | 53 | 231.83 | 0 | AntiSense | -11.2 | 0.902 |
| candidate_454 | + | A | 83 | 168.39 | 0 | AntiSense | -28.06 | 0.439 |
| candidate_455 | + | A | 30 | 132.42 | 2.61 | NonCoding | -7.4 | 0.223 |
| candidate_456 | + | A | 36 | 145.16 | 0 | AntiSense | -6.5 | 0.974 |
| candidate_457 | + | A | 50 | 545.88 | 0.23 | 5'/3'-UTR | -7.6 | 0.295 |
| candidate_458 | + | A | 36 | 269.41 | 0 | AntiSense | -9.8 | 0.22 |
| candidate_459 | + | A | 32 | 121.03 | 0 | AntiSense | -2 | 0.997 |
| candidate_460 | + | A | 32 | 359.21 | 0 | AntiSense | -4.7 | 0.655 |
| candidate_461 | + | A | 41 | 80.95 | 0 | AntiSense | -9.8 | 0.551 |
| candidate_462 | + | A | 55 | 525.45 | 0 | AntiSense | -23.6 | 0.049 |
| candidate_463 | + | A | 162 | 1067.06 | 0.08 | 5'/3'-UTR | -55.3 | 0.42 |
| candidate_464 | + | A | 47 | 1115.1 | 1.78 | NonCoding | -10.9 | 0.278 |
| candidate_465 | + | A | 57 | 386.52 | 1.67 | NonCoding | -13.5 | 0.922 |
| candidate_466 | + | A | 114 | 94.42 | 0 | AntiSense | -37.1 | 0.463 |
| candidate_467 | + | A | 92 | 235.46 | 0 | AntiSense | -33.8 | 0.134 |
| candidate_468 | + | A | 30 | 80.9 | 0.13 | NonCoding | -1.7 | 0.818 |
| candidate_469 | + | A | 107 | 153.54 | 0 | AntiSense | -29.6 | 0.946 |
| candidate_470 | + | A | 59 | 536.13 | 0 | AntiSense | -20.1 | 0.263 |
| candidate_471 | + | A | 36 | 137.51 | 0.1 | NonCoding | -8.9 | 0.562 |
| candidate_472 | + | A | 195 | 338.01 | 0 | AntiSense | -79.1 | 0.61 |
| candidate_473 | + | A | 35 | 228.06 | 0 | AntiSense | -8 | 0.477 |
| candidate_474 | + | A | 85 | 116.99 | 0 | AntiSense | -22.4 | 0.502 |
| candidate_475 | + | A | 35 | 265.64 | 0 | AntiSense | -11.4 | 0.092 |
| candidate_476 | + | A | 32 | 148.09 | 0 | AntiSense | -7.3 | 0.581 |
| candidate_477 | + | A | 37 | 123.95 | 0 | AntiSense | -11.3 | 0.237 |
| candidate_478 | + | A | 86 | 84.75 | 0.02 | 5'/3'-UTR | -21.07 | 0.052 |
| candidate_479 | + | A | 81 | 620.73 | 0 | AntiSense | -34.9 | 0.093 |
| candidate_480 | + | A | 31 | 116.22 | 0 | AntiSense | -4.4 | 0.912 |
| candidate_481 | + | A | 38 | 177.77 | 0.09 | NonCoding | -9.1 | 0.977 |
| candidate_482 | + | A | 62 | 274.35 | 0.13 | NonCoding | -11.8 | 0.16 |
| candidate_483 | + | A | 147 | 114.37 | 0.15 | NonCoding | -57.1 | 0.203 |
| candidate_484 | + | A | 58 | 370.75 | 0.1 | NonCoding | -5.5 | 0.877 |
| candidate_485 | + | A | 46 | 189.4 | 1.32 | NonCoding | -18.6 | 0.001 |
| candidate_486 | + | A | 36 | 142.51 | 0 | AntiSense | -14.2 | 0.173 |
| candidate_487 | + | A | 150 | 313.77 | 0.09 | NonCoding | -49.32 | 0.579 |
| candidate_488 | + | A | 278 | 557.25 | 0.2 | 5'/3'-UTR | -93.3 | 0.954 |
| candidate_489 | + | A | 53 | 149.78 | 0 | AntiSense | -15.21 | 0.411 |
| candidate_490 | + | A | 220 | 353.17 | 0.03 | 5'/3'-UTR | -81 | 0.513 |
| candidate_491 | + | A | 30 | 66.52 | 0 | AntiSense | -2.9 | 0.801 |
| candidate_492 | + | A | 108 | 94.38 | 0 | AntiSense | -31.7 | 0.701 |
| candidate_493 | + | A | 31 | 75.22 | 0 | AntiSense | -12.6 | 0.044 |
| candidate_494 | + | A | 60 | 136.41 | 0.38 | NonCoding | -8.7 | 0.712 |
| candidate_495 | + | A | 34 | 137.97 | 1.43 | NonCoding | -19.9 | 0.001 |
| candidate_496 | + | A | 136 | 228.28 | 2.05 | NonCoding | -33.2 | 0.596 |
| candidate_497 | + | A | 40 | 166.83 | 0.76 | NonCoding | -35.8 | 0.001 |
| candidate_498 | + | A | 299 | 625.47 | 1.04 | 5'/3'-UTR | -124.2 | 0.203 |
| candidate_499 | + | A | 62 | 235.43 | 0 | AntiSense | -12.5 | 0.758 |
| candidate_500 | + | A | 57 | 249.24 | 0.49 | 5'/3'-UTR | -16.2 | 0.431 |
| candidate_501 | + | A | 30 | 112.77 | 0 | AntiSense | -2.3 | 0.974 |
| candidate_502 | + | A | 32 | 125.91 | 0 | AntiSense | -7.2 | 0.435 |
| candidate_503 | + | A | 52 | 3063.09 | 2.45 | NonCoding | -9.7 | 0.431 |
| candidate_504 | + | A | 112 | 427.87 | 1.23 | NonCoding | -35 | 0.566 |
| candidate_505 | + | A | 77 | 190.49 | 4.86 | NonCoding | -17.3 | 0.344 |
| candidate_506 | + | A | 49 | 553 | 0.1 | NonCoding | -8.5 | 0.579 |
| candidate_507 | + | A | 48 | 134.88 | 0 | AntiSense | -10.4 | 0.84 |
| candidate_508 | + | A | 31 | 74.25 | 0 | AntiSense | -5.5 | 0.583 |
| candidate_509 | + | A | 117 | 187.53 | 0 | AntiSense | -27.44 | 0.859 |
| candidate_510 | + | A | 43 | 150.32 | 0 | AntiSense | -7.5 | 0.828 |
| candidate_511 | + | A | 38 | 209.87 | 0 | AntiSense | -10.7 | 0.108 |
| candidate_512 | + | A | 89 | 397.06 | 0 | AntiSense | -32.7 | 0.274 |
| candidate_513 | + | A | 72 | 105.21 | 0 | AntiSense | -25.6 | 0.29 |
| candidate_514 | + | A | 116 | 110.51 | 0 | AntiSense | -47.2 | 0.146 |
| candidate_515 | + | A | 42 | 100.91 | 0 | AntiSense | -17.9 | 0.035 |
| candidate_516 | + | A | 78 | 427.33 | 0 | AntiSense | -27.4 | 0.457 |
| candidate_517 | + | A | 43 | 121.64 | 0 | 5'/3'-UTR | -10.52 | 0.303 |
| candidate_518 | + | A | 30 | 92.74 | 0 | AntiSense | -4 | 0.614 |
| candidate_519 | + | A | 36 | 81.97 | 0 | AntiSense | -3.6 | 0.858 |
| candidate_520 | + | A | 239 | 481.05 | 0.02 | 5'/3'-UTR | -92.3 | 0.921 |
| candidate_521 | + | A | 124 | 234.66 | 0 | AntiSense | -47.8 | 0.841 |
| candidate_522 | + | A | 45 | 90.2 | 0 | AntiSense | -16.1 | 0.123 |
| candidate_523 | + | A | 41 | 447.1 | 0 | AntiSense | -15.7 | 0.026 |
| candidate_524 | + | A | 134 | 197.46 | 0.38 | 5'/3'-UTR | -44.5 | 0.245 |
| candidate_525 | + | A | 44 | 84.47 | 0 | AntiSense | -9.8 | 0.579 |
| candidate_526 | + | A | 42 | 104.16 | 0.1 | NonCoding | -14.4 | 0.246 |
| candidate_527 | + | A | 33 | 116 | 0.1 | NonCoding | -4 | 0.598 |
| candidate_528 | + | A | 91 | 336.51 | 0.3 | 5'/3'-UTR | -17.7 | 0.983 |
| candidate_529 | + | A | 35 | 160.06 | 0.14 | NonCoding | -7.7 | 0.228 |
| candidate_530 | + | A | 60 | 190.31 | 0 | AntiSense | -13.7 | 0.723 |
| candidate_531 | + | A | 58 | 296.46 | 0.14 | NonCoding | -16.8 | 0.872 |
| candidate_532 | + | A | 30 | 197.52 | 0 | AntiSense | -7 | 0.752 |
| candidate_533 | + | A | 35 | 284.42 | 1.36 | NonCoding | -3 | 0.791 |
| candidate_534 | + | A | 31 | 612.31 | 0 | AntiSense | -0.4 | 0.87 |
| candidate_535 | + | A | 54 | 377.2 | 0 | AntiSense | -10.1 | 0.721 |
| candidate_536 | + | A | 32 | 498.79 | 0 | AntiSense | -2.9 | 0.895 |
| candidate_537 | + | A | 30 | 155.03 | 0 | AntiSense | -8.1 | 0.296 |
| candidate_538 | + | A | 38 | 177.97 | 0.2 | 5'/3'-UTR | -3.2 | 0.938 |
| candidate_539 | + | A | 42 | 135.84 | 2.48 | NonCoding | -1.9 | 0.909 |
| candidate_540 | + | A | 129 | 1191.41 | 0.43 | 5'/3'-UTR | -27.9 | 0.862 |
| candidate_541 | + | A | 35 | 161.28 | 0 | AntiSense | -8.4 | 0.663 |
| candidate_542 | + | A | 40 | 100.54 | 0 | AntiSense | -16.7 | 0.01 |
| candidate_543 | + | A | 140 | 315.56 | 0.65 | 5'/3'-UTR | -41.3 | 0.386 |
| candidate_544 | + | A | 35 | 98.03 | 0 | AntiSense | -3 | 0.915 |
| candidate_545 | + | A | 85 | 103.07 | 0 | AntiSense | -29.6 | 0.093 |
| candidate_546 | + | A | 37 | 537.76 | 0 | AntiSense | -4.1 | 0.803 |
| candidate_547 | + | A | 85 | 233.8 | 0 | 5'/3'-UTR | -31.2 | 0.284 |
| candidate_548 | + | A | 136 | 385.09 | 0 | AntiSense | -41.4 | 0.944 |
| candidate_549 | + | A | 39 | 156.75 | 1.59 | NonCoding | -6.7 | 0.879 |
| candidate_550 | + | A | 51 | 123 | 0 | AntiSense | -11.9 | 0.697 |
| candidate_551 | + | A | 59 | 112.92 | 0 | AntiSense | -13 | 0.547 |
| candidate_552 | + | A | 138 | 242.78 | 0.07 | 5'/3'-UTR | -37.4 | 0.774 |
| candidate_553 | + | A | 41 | 168.24 | 0 | AntiSense | -13.5 | 0.027 |
| candidate_554 | + | A | 112 | 355.08 | 0.14 | NonCoding | -30.2 | 0.834 |
| candidate_555 | + | A | 30 | 137.42 | 0 | AntiSense | -6.5 | 0.281 |
| candidate_556 | + | A | 69 | 479.3 | 0.1 | 5'/3'-UTR | -44.4 | 0.001 |
| candidate_557 | + | A | 34 | 120.71 | 0 | 5'/3'-UTR | -5.8 | 0.682 |
| candidate_558 | + | A | 30 | 100.74 | 0 | AntiSense | -9.8 | 0.204 |
| candidate_559 | + | A | 124 | 853.55 | 0 | AntiSense | -40.9 | 0.645 |
| candidate_560 | + | A | 60 | 1691.13 | 6.09 | NonCoding | -17.9 | 0.125 |
| candidate_561 | + | A | 132 | 167.04 | 2.37 | NonCoding | -47.1 | 0.804 |
| candidate_562 | + | A | 77 | 322.86 | 0.44 | NonCoding | -35.8 | 0.025 |
| candidate_563 | + | A | 98 | 494.39 | 0 | AntiSense | -38.6 | 0.394 |
| candidate_564 | + | A | 45 | 184.91 | 0.06 | 5'/3'-UTR | -7 | 0.842 |
| candidate_565 | + | A | 60 | 450.33 | 0 | 5'/3'-UTR | -16.4 | 0.059 |
| candidate_566 | + | A | 87 | 178.78 | 0 | AntiSense | -33.3 | 0.249 |
| candidate_567 | + | A | 30 | 120.55 | 0 | AntiSense | -2 | 0.893 |
| candidate_568 | + | A | 33 | 491.26 | 0 | AntiSense | -7.3 | 0.829 |
| candidate_569 | + | A | 51 | 159.94 | 0.79 | NonCoding | -4.2 | 0.875 |
| candidate_570 | + | A | 37 | 250.29 | 2.12 | NonCoding | -13.4 | 0.038 |
| candidate_571 | + | A | 187 | 322.54 | 0.11 | NonCoding | -84.4 | 0.145 |
| candidate_572 | + | A | 43 | 231.41 | 0 | AntiSense | -5.1 | 0.364 |
| candidate_573 | + | A | 61 | 221.71 | 0 | AntiSense | -13.2 | 0.752 |
| candidate_574 | + | A | 37 | 105 | 0 | AntiSense | -6.1 | 0.637 |
| candidate_575 | + | A | 57 | 680.86 | 0 | AntiSense | -14.5 | 0.846 |
| candidate_576 | + | A | 39 | 441.53 | 0 | AntiSense | -1.7 | 0.917 |
| candidate_577 | + | A | 36 | 242.89 | 0 | AntiSense | -9.1 | 0.219 |
| candidate_578 | + | A | 38 | 172.56 | 0 | AntiSense | -9.22 | 0.647 |
| candidate_579 | + | A | 33 | 251.62 | 0.94 | NonCoding | -8.5 | 0.35 |
| candidate_580 | + | A | 127 | 325.01 | 0 | AntiSense | -37.6 | 0.993 |
| candidate_581 | + | A | 117 | 437.55 | 0 | AntiSense | -43 | 0.312 |
| candidate_582 | + | A | 55 | 139 | 0 | AntiSense | -9.9 | 0.933 |
| candidate_583 | + | A | 57 | 244.02 | 0 | AntiSense | -15.3 | 0.382 |
| candidate_584 | + | A | 30 | 72.77 | 0 | AntiSense | -4 | 0.772 |
| candidate_585 | + | A | 31 | 120.06 | 0.09 | 5'/3'-UTR | -3.1 | 0.314 |
| candidate_586 | + | A | 202 | 596.49 | 0 | AntiSense | -93.9 | 0.002 |
| candidate_587 | + | A | 77 | 116.65 | 0 | 5'/3'-UTR | -20.9 | 0.624 |
| candidate_588 | + | A | 80 | 133.35 | 0 | AntiSense | -18.1 | 0.107 |
| candidate_589 | + | A | 49 | 222.44 | 0.5 | NonCoding | -17.1 | 0.151 |
| candidate_590 | + | A | 94 | 131.06 | 0 | AntiSense | -28.5 | 0.089 |
| candidate_591 | + | A | 37 | 140 | 0 | AntiSense | -12.2 | 0.06 |
| candidate_592 | + | A | 116 | 1878.05 | 1.83 | NonCoding | -39.7 | 0.132 |
| candidate_593 | + | A | 88 | 337.8 | 0.35 | NonCoding | -20.4 | 0.762 |
| candidate_594 | + | A | 30 | 196.26 | 0.09 | NonCoding | -3.6 | 0.043 |
| candidate_595 | + | A | 81 | 602.74 | 0.25 | NonCoding | -39.4 | 0.001 |
| candidate_596 | + | A | 103 | 658.87 | 0.11 | 5'/3'-UTR | -21.1 | 0.935 |
| candidate_597 | + | A | 110 | 408.07 | 0.06 | 5'/3'-UTR | -33.1 | 0.707 |
| candidate_598 | + | A | 35 | 84.56 | 2 | NonCoding | -8 | 0.455 |
| candidate_599 | + | A | 42 | 173.56 | 0 | AntiSense | -5.6 | 0.952 |
| candidate_600 | + | A | 30 | 109.45 | 0 | 5'/3'-UTR | 0 | 1 |
| candidate_601 | + | A | 85 | 157.2 | 0.04 | 5'/3'-UTR | -26.2 | 0.405 |
| candidate_602 | + | B | 44 | 201.98 | 4.79 | NonCoding | -10.2 | 0.783 |
| candidate_603 | + | B | 30 | 129.52 | 0.95 | NonCoding | -5.5 | 0.786 |
| candidate_604 | + | B | 36 | 68.65 | 5.77 | NonCoding | -14.4 | 0.158 |
| candidate_605 | + | B | 33 | 77.38 | 2.85 | NonCoding | -11.5 | 0.863 |
| candidate_606 | + | B | 40 | 61.9 | 2.68 | NonCoding | -9 | 0.096 |
| candidate_607 | + | B | 44 | 104.04 | 0.95 | NonCoding | -7.3 | 0.76 |
| candidate_608 | + | B | 40 | 223.22 | 4.64 | NonCoding | -9.2 | 0.937 |
| candidate_609 | + | B | 31 | 76.69 | 2.4 | NonCoding | -4.9 | 0.815 |
| candidate_610 | + | B | 41 | 43.9 | 0.95 | NonCoding | -10.5 | 0.249 |
| candidate_611 | + | B | 49 | 310.6 | 2.5 | NonCoding | -30 | 0.005 |
| candidate_612 | + | B | 82 | 102.89 | 1.6 | NonCoding | -25.24 | 0.627 |
| candidate_613 | + | B | 48 | 58.12 | 1.58 | NonCoding | -24.2 | 0.008 |
| candidate_614 | + | B | 52 | 221.28 | 2.41 | NonCoding | -9.5 | 0.025 |
| candidate_615 | + | B | 35 | 30.22 | 5.3 | NonCoding | -10.1 | 0.457 |
| candidate_616 | + | B | 51 | 274.08 | 3.87 | NonCoding | -10.9 | 0.844 |
| candidate_617 | + | B | 31 | 47.16 | 0.95 | NonCoding | -6.9 | 0.594 |
| candidate_618 | + | B | 32 | 97.09 | 1.98 | NonCoding | -21.9 | 0.002 |
| candidate_619 | + | B | 40 | 99.17 | 2.64 | NonCoding | -10.2 | 0.417 |
| candidate_620 | + | B | 30 | 44.9 | 1.3 | NonCoding | -13.9 | 0.014 |
| candidate_621 | + | B | 37 | 86.5 | 2.91 | NonCoding | -13.9 | 0.294 |
| candidate_622 | + | B | 35 | 305.56 | 2.02 | NonCoding | -7.3 | 0.52 |
| candidate_623 | + | B | 44 | 98.11 | 4.17 | NonCoding | -8 | 0.801 |
| candidate_624 | + | B | 31 | 33.5 | 1.49 | NonCoding | -10.4 | 0.311 |
| candidate_625 | + | B | 39 | 124.45 | 1.77 | NonCoding | -6.1 | 0.565 |
| candidate_626 | + | B | 30 | 169.42 | 0.96 | NonCoding | -10.1 | 0.756 |
| candidate_627 | + | B | 30 | 208.35 | 1.91 | NonCoding | -4 | 0.508 |
| candidate_628 | + | B | 44 | 51.64 | 1.79 | NonCoding | -10.7 | 0.544 |
| candidate_629 | + | B | 51 | 240.13 | 1.69 | NonCoding | -33 | 0.001 |
| candidate_630 | + | B | 38 | 253.97 | 2.3 | NonCoding | -16.6 | 0.113 |
| candidate_631 | + | B | 54 | 28.78 | 1.36 | NonCoding | -18 | 0.306 |
| candidate_632 | + | B | 30 | 290.45 | 0.95 | NonCoding | -1.7 | 0.63 |
| candidate_633 | + | B | 31 | 54.63 | 1.28 | NonCoding | -5.8 | 0.667 |
| candidate_634 | + | B | 35 | 39.58 | 3.52 | NonCoding | -7 | 0.408 |
| candidate_635 | + | B | 32 | 64.42 | 2.2 | NonCoding | -8.6 | 0.337 |
| candidate_636 | + | B | 41 | 354.21 | 1.77 | NonCoding | -13.9 | 0.085 |
| candidate_637 | + | B | 35 | 58.36 | 2.21 | NonCoding | -10.5 | 0.373 |
| candidate_638 | + | B | 33 | 2738.85 | 1.19 | NonCoding | -17.5 | 0.011 |
| candidate_639 | + | B | 69 | 42.63 | 2.23 | NonCoding | -14.7 | 0.697 |
| candidate_640 | + | B | 39 | 69.3 | 0.95 | NonCoding | -8 | 0.2 |
| candidate_641 | + | B | 74 | 72.79 | 2.45 | NonCoding | -15.3 | 0.981 |
| candidate_642 | + | B | 58 | 944.49 | 0.96 | NonCoding | -8.4 | 0.585 |
| candidate_643 | + | B | 33 | 48.06 | 1.84 | NonCoding | -12 | 0.16 |
| candidate_644 | + | B | 32 | 63.48 | 1.8 | NonCoding | -5.5 | 0.666 |
| candidate_645 | + | B | 35 | 56.64 | 2.38 | NonCoding | -12 | 0.075 |
| candidate_646 | + | B | 46 | 52.55 | 0.95 | NonCoding | -12.7 | 0.193 |
| candidate_647 | + | B | 36 | 59 | 1.42 | NonCoding | -24.4 | 0.004 |
| candidate_648 | + | B | 38 | 77.21 | 3.31 | NonCoding | -5.8 | 0.959 |
| candidate_649 | + | B | 46 | 67.3 | 1.34 | NonCoding | -12.1 | 0.94 |
| candidate_650 | + | B | 67 | 67.5 | 1.37 | NonCoding | -18.1 | 0.258 |
| candidate_651 | + | B | 30 | 83.94 | 2.31 | NonCoding | -22 | 0.001 |
| candidate_652 | + | B | 35 | 36.61 | 1.01 | NonCoding | -12.1 | 0.013 |
| candidate_653 | + | B | 32 | 45.33 | 2.26 | NonCoding | 0 | 1 |
| candidate_654 | + | B | 48 | 60.16 | 2.55 | NonCoding | -13.4 | 0.429 |
| candidate_655 | + | B | 32 | 40.79 | 0.95 | NonCoding | -4.2 | 0.588 |
| candidate_656 | + | B | 33 | 69.91 | 1.58 | NonCoding | -6.4 | 0.071 |
| candidate_657 | + | B | 60 | 150.16 | 5.56 | NonCoding | -23.8 | 0.516 |
| candidate_658 | + | B | 78 | 145.33 | 3.75 | NonCoding | -23.8 | 0.96 |
| candidate_659 | + | B | 30 | 58.94 | 0.95 | NonCoding | -2.6 | 0.917 |
| candidate_660 | + | B | 37 | 259.29 | 1.91 | NonCoding | -12.2 | 0.047 |
| candidate_661 | + | B | 51 | 155.62 | 1.69 | NonCoding | -12.8 | 0.269 |
| candidate_662 | + | B | 30 | 42.06 | 2.41 | NonCoding | -7.1 | 0.136 |
| candidate_663 | + | C | 34 | 50.31 | 1.77 | NonCoding | -9.9 | 0.222 |
| candidate_664 | + | C | 52 | 5.7 | 3.55 | NonCoding | -12.6 | 0.194 |
| candidate_665 | + | C | 30 | 4.9 | 2.2 | NonCoding | -5.1 | 0.477 |
| candidate_666 | + | C | 41 | 15.48 | 5.32 | NonCoding | -14.7 | 0.37 |
| candidate_667 | + | C | 34 | 3.54 | 2.49 | NonCoding | -2.9 | 0.92 |
| candidate_668 | + | C | 33 | 15.18 | 1.77 | NonCoding | -13.8 | 0.004 |
| candidate_669 | + | C | 98 | 1.03 | 2.57 | NonCoding | -37.1 | 0.449 |
| candidate_670 | + | C | 51 | 2.4 | 1.77 | NonCoding | -14 | 0.298 |
| candidate_671 | + | C | 56 | 1.91 | 2.17 | NonCoding | -26.6 | 0.006 |
| candidate_672 | + | C | 49 | 0.48 | 3.89 | NonCoding | -16.2 | 0.129 |
| candidate_673 | + | C | 44 | 19.04 | 2.59 | NonCoding | -14.7 | 0.521 |
| candidate_674 | + | C | 87 | 0.9 | 3.36 | NonCoding | -34.7 | 0.342 |
| candidate_675 | + | C | 53 | 231.43 | 3.44 | NonCoding | -11.3 | 0.399 |
| candidate_676 | + | C | 42 | 24.53 | 1.77 | NonCoding | -28.3 | 0.001 |
| candidate_677 | + | C | 30 | 120.84 | 2.37 | NonCoding | -4.1 | 0.018 |
| candidate_678 | + | C | 30 | 21.13 | 3.12 | NonCoding | -10.1 | 0.002 |
| candidate_679 | + | C | 31 | 1.75 | 2.3 | NonCoding | -4.5 | 0.49 |
| candidate_680 | + | C | 56 | 5.98 | 4.33 | NonCoding | -13 | 0.17 |
| candidate_681 | + | C | 86 | 6.14 | 3.93 | NonCoding | -31.5 | 0.919 |
| candidate_682 | + | C | 92 | 1.77 | 5.48 | NonCoding | -39.9 | 0.083 |
| candidate_683 | + | C | 35 | 0.86 | 3.99 | NonCoding | -12.2 | 0.012 |
| candidate_684 | + | C | 32 | 2.24 | 1.77 | NonCoding | -5.9 | 0.73 |
| candidate_685 | + | C | 33 | 0 | 1.77 | NonCoding | -5.5 | 0.458 |
| candidate_686 | + | C | 30 | 7.84 | 1.8 | NonCoding | -4.12 | 0.76 |
| candidate_687 | + | C | 45 | 15.98 | 1.79 | NonCoding | -14.6 | 0.286 |
| candidate_688 | + | C | 39 | 12.1 | 1.77 | NonCoding | -15.29 | 0.179 |
| candidate_689 | + | C | 62 | 9.29 | 2.69 | NonCoding | -19 | 0.881 |
| candidate_690 | + | C | 69 | 0.26 | 2.65 | NonCoding | -24.9 | 0.571 |
| candidate_691 | + | C | 68 | 1.62 | 2.66 | NonCoding | -24.9 | 0.502 |
| candidate_692 | + | C | 89 | 32.59 | 4.79 | NonCoding | -20.9 | 0.762 |
| candidate_693 | + | C | 42 | 1.58 | 2.3 | NonCoding | -14.9 | 0.628 |
| candidate_694 | + | C | 46 | 1.38 | 2.25 | NonCoding | -16 | 0.712 |
| candidate_695 | + | C | 55 | 99.34 | 2.37 | NonCoding | -19.1 | 0.496 |
| candidate_696 | + | C | 42 | 24.51 | 3.04 | NonCoding | -17.1 | 0.073 |
| candidate_697 | + | C | 95 | 21.85 | 4.8 | NonCoding | -34.1 | 0.729 |
| candidate_698 | + | C | 41 | 1 | 2.75 | NonCoding | -21 | 0.044 |
| candidate_699 | + | C | 40 | 4.24 | 4.82 | NonCoding | -10.9 | 0.228 |
| candidate_700 | + | C | 40 | 2.8 | 1.77 | NonCoding | -15.2 | 0.078 |
| candidate_701 | + | C | 31 | 4.06 | 1.77 | NonCoding | -6.2 | 0.191 |
| candidate_702 | + | C | 37 | 10.39 | 2.53 | NonCoding | -7.6 | 0.649 |
| candidate_703 | + | C | 46 | 0.87 | 2.56 | NonCoding | -16.6 | 0.11 |
| candidate_704 | + | C | 152 | 35.97 | 3.26 | NonCoding | -72 | 0.018 |
| candidate_705 | + | C | 35 | 198.31 | 2.61 | NonCoding | -6.7 | 0.36 |
| candidate_706 | + | C | 46 | 10.98 | 1.77 | NonCoding | -14.4 | 0.505 |
| candidate_707 | + | C | 68 | 20.09 | 2.28 | NonCoding | -16.6 | 0.674 |
| candidate_708 | + | C | 68 | 4.2 | 2.26 | NonCoding | -24.3 | 0.583 |
| candidate_709 | + | C | 32 | 0.18 | 2.57 | NonCoding | -11.4 | 0.382 |
| candidate_710 | + | C | 96 | 18.98 | 4.8 | NonCoding | -39.3 | 0.035 |
| candidate_711 | + | C | 45 | 2.17 | 1.85 | NonCoding | -14.3 | 0.165 |
| candidate_712 | + | C | 63 | 11.52 | 3.85 | NonCoding | -6.5 | 0.941 |
| candidate_713 | + | C | 65 | 20.55 | 3.4 | NonCoding | -20 | 0.64 |
| candidate_714 | + | C | 38 | 0 | 2.19 | NonCoding | -15.2 | 0.209 |
| candidate_715 | + | C | 154 | 0.27 | 2.49 | NonCoding | -61.7 | 0.643 |
| candidate_716 | + | C | 44 | 21.8 | 3.19 | NonCoding | -19.8 | 0.063 |
| candidate_717 | + | C | 35 | 15.83 | 1.89 | NonCoding | -4.14 | 0.918 |
| candidate_718 | + | C | 30 | 467.1 | 2.04 | NonCoding | -6.6 | 0.752 |
| candidate_719 | + | C | 136 | 6.44 | 4.03 | NonCoding | -59.2 | 0.011 |
| candidate_720 | + | C | 58 | 34.15 | 2.76 | NonCoding | -55.8 | 0.001 |
| candidate_721 | + | C | 54 | 3.91 | 3.72 | NonCoding | -16 | 0.029 |
| candidate_722 | + | C | 30 | 2.9 | 2.27 | NonCoding | -7.3 | 0.032 |
| candidate_723 | + | C | 43 | 36.86 | 2.27 | NonCoding | -2.9 | 0.995 |
| candidate_724 | + | C | 45 | 4.43 | 2.57 | NonCoding | -13.8 | 0.537 |
| candidate_725 | + | C | 34 | 8.91 | 2.28 | NonCoding | -5.7 | 0.684 |
| candidate_726 | + | C | 40 | 2.17 | 3.38 | NonCoding | -9.4 | 0.428 |
| candidate_727 | + | C | 47 | 26.92 | 2.5 | NonCoding | -16.4 | 0.453 |
| candidate_728 | + | C | 34 | 4.4 | 1.77 | NonCoding | -7.3 | 0.096 |
| candidate_729 | + | C | 50 | 10.71 | 3.03 | NonCoding | -21.1 | 0.43 |
| candidate_730 | + | C | 80 | 30.4 | 2.19 | NonCoding | -24.6 | 0.644 |
| candidate_731 | + | C | 63 | 6.06 | 2.51 | NonCoding | -23.7 | 0.167 |
| candidate_732 | + | C | 47 | 1.5 | 3.53 | NonCoding | -10.42 | 0.137 |
| candidate_733 | + | C | 55 | 1.52 | 2.45 | NonCoding | -9.1 | 0.765 |
| candidate_734 | + | C | 31 | 10.78 | 2.57 | NonCoding | -0.6 | 0.974 |
| candidate_735 | + | C | 44 | 12.56 | 2.19 | NonCoding | -6.4 | 0.392 |
| candidate_736 | + | C | 44 | 7.09 | 1.77 | NonCoding | -8.1 | 0.289 |
| candidate_737 | + | C | 53 | 5.3 | 3.37 | NonCoding | -16.9 | 0.72 |
| candidate_738 | + | C | 38 | 1.31 | 2.2 | NonCoding | -16.7 | 0.354 |
| candidate_739 | + | C | 39 | 0 | 2.2 | NonCoding | -16.7 | 0.37 |
| candidate_740 | + | C | 39 | 0 | 2.2 | NonCoding | -16.7 | 0.383 |
| candidate_741 | + | C | 39 | 34.85 | 2.2 | NonCoding | -16.7 | 0.365 |
| candidate_742 | + | C | 42 | 8.42 | 2.2 | NonCoding | -20 | 0.03 |
| candidate_743 | + | C | 46 | 10.98 | 2.18 | NonCoding | -12.7 | 0.018 |
| candidate_744 | + | C | 39 | 18.55 | 2.85 | NonCoding | -6.9 | 0.724 |
| candidate_745 | + | C | 72 | 24.49 | 3.15 | NonCoding | -23 | 0.734 |
| candidate_746 | + | C | 35 | 4.81 | 2.54 | NonCoding | -9.9 | 0.23 |
| candidate_747 | + | C | 50 | 0.76 | 2.55 | NonCoding | -11.8 | 0.626 |
| candidate_748 | + | C | 39 | 25.85 | 2.02 | NonCoding | -17.4 | 0.016 |
| candidate_749 | + | C | 100 | 0 | 2.6 | NonCoding | -35.8 | 0.14 |
| candidate_750 | + | C | 42 | 27.72 | 2.2 | NonCoding | -11.6 | 0.726 |
| candidate_751 | + | C | 99 | 20.82 | 2.63 | NonCoding | -31.5 | 0.297 |
| candidate_752 | + | C | 60 | 6.62 | 2.34 | NonCoding | -25.1 | 0.32 |
| candidate_753 | + | C | 50 | 98.47 | 2.76 | NonCoding | -12.3 | 0.722 |
| candidate_754 | + | C | 66 | 8.75 | 2.7 | NonCoding | -22 | 0.672 |
| candidate_755 | + | C | 160 | 10.21 | 4.78 | NonCoding | -73.6 | 0.623 |
| candidate_756 | + | C | 46 | 1.79 | 2.94 | NonCoding | -12.5 | 0.662 |
| candidate_757 | + | C | 30 | 2.42 | 2.61 | NonCoding | -7.3 | 0.387 |
| candidate_758 | + | C | 39 | 1.3 | 2.17 | NonCoding | -8.2 | 0.594 |
| candidate_759 | + | C | 61 | 5.31 | 3.64 | NonCoding | -15.4 | 0.904 |
| candidate_760 | + | C | 38 | 10.44 | 2.59 | NonCoding | -17.7 | 0.059 |
| candidate_761 | + | C | 47 | 12.42 | 2.25 | NonCoding | -15.2 | 0.599 |
| candidate_762 | + | C | 35 | 10.5 | 4.14 | NonCoding | -8.9 | 0.262 |
| candidate_763 | + | C | 35 | 8.78 | 4.22 | NonCoding | -12.2 | 0.125 |
| candidate_764 | + | C | 42 | 0.53 | 2.56 | NonCoding | -12.2 | 0.369 |
| candidate_765 | + | C | 31 | 2 | 2.14 | NonCoding | -9.1 | 0.046 |
| candidate_766 | + | C | 93 | 14.05 | 2.9 | NonCoding | -40.8 | 0.017 |
| candidate_767 | + | C | 74 | 0.63 | 2.47 | NonCoding | -31.2 | 0.355 |
| candidate_768 | + | C | 48 | 0 | 2.43 | NonCoding | -20.3 | 0.209 |
| candidate_769 | + | C | 36 | 21.7 | 4.26 | NonCoding | -6.5 | 0.871 |
| candidate_770 | + | C | 30 | 7.23 | 1.79 | NonCoding | -7 | 0.589 |
| candidate_771 | + | C | 40 | 6.05 | 2.07 | NonCoding | -4.9 | 0.808 |
| candidate_772 | + | C | 35 | 6.19 | 1.79 | NonCoding | -5.9 | 0.264 |
| candidate_773 | + | C | 35 | 22.83 | 2.54 | NonCoding | -11 | 0.036 |
| candidate_774 | + | C | 36 | 2.24 | 2.61 | NonCoding | -7.7 | 0.549 |
| candidate_775 | + | C | 50 | 16.47 | 2.53 | NonCoding | -9.2 | 0.385 |
| candidate_776 | + | C | 52 | 65.77 | 2.11 | NonCoding | -15.9 | 0.033 |
| candidate_777 | + | C | 43 | 0 | 1.8 | NonCoding | -18.1 | 0.491 |
| candidate_778 | + | C | 102 | 82.82 | 3.33 | NonCoding | -30.9 | 0.125 |
| candidate_779 | + | C | 91 | 0.78 | 3.74 | NonCoding | -63.2 | 0.001 |
| candidate_780 | + | C | 50 | 6.75 | 3.95 | NonCoding | 0 | 1 |
| candidate_781 | + | C | 56 | 35.39 | 1.77 | NonCoding | -6.2 | 0.979 |
| candidate_782 | + | C | 62 | 13.84 | 2.1 | NonCoding | -14.7 | 0.992 |
| candidate_783 | + | C | 62 | 18.38 | 2.22 | NonCoding | -7.3 | 0.738 |
| candidate_784 | + | C | 44 | 5.47 | 4.77 | NonCoding | -7.8 | 0.597 |
| candidate_785 | + | C | 61 | 145.35 | 1.77 | NonCoding | -17.2 | 0.836 |
| candidate_786 | + | C | 40 | 11.02 | 2.88 | NonCoding | -15.8 | 0.163 |
| candidate_787 | + | C | 31 | 6.25 | 4.95 | NonCoding | -6.33 | 0.692 |
| candidate_788 | + | C | 71 | 3.38 | 5.54 | NonCoding | -7.6 | 0.652 |
| candidate_789 | + | C | 32 | 11.36 | 3.2 | NonCoding | -4.1 | 0.786 |
| candidate_790 | + | C | 311 | 0 | 2.52 | NonCoding | -90.14 | 0.393 |
| candidate_791 | + | C | 33 | 4.74 | 1.77 | NonCoding | -9.2 | 0.059 |
| candidate_792 | + | C | 59 | 1.23 | 2.54 | NonCoding | -9.5 | 0.825 |
| candidate_793 | + | C | 33 | 4.71 | 1.77 | NonCoding | -3.3 | 0.789 |
| candidate_794 | + | C | 64 | 0.37 | 3.34 | NonCoding | -29.9 | 0.046 |
| candidate_795 | + | C | 30 | 5.48 | 3.96 | NonCoding | -7.7 | 0.264 |
| candidate_796 | + | C | 259 | 9.71 | 3.37 | NonCoding | -98.9 | 0.275 |
| candidate_797 | + | C | 71 | 20.6 | 2.07 | NonCoding | -33.8 | 0.004 |
| candidate_798 | + | C | 34 | 8.4 | 3.04 | NonCoding | -14.2 | 0.04 |
| candidate_799 | + | C | 38 | 0.15 | 2.52 | NonCoding | -5.6 | 0.352 |
| candidate_800 | + | C | 39 | 0 | 2.49 | NonCoding | -14.2 | 0.352 |
| candidate_801 | + | C | 34 | 0.57 | 2.58 | NonCoding | -11.6 | 0.047 |
| candidate_802 | + | C | 79 | 0.51 | 4.68 | NonCoding | -23.5 | 0.084 |
| candidate_803 | + | C | 87 | 0 | 1.95 | NonCoding | -46.1 | 0.193 |
| candidate_804 | + | C | 93 | 0.41 | 2.54 | NonCoding | -38.6 | 0.465 |
| candidate_805 | + | C | 157 | 0 | 2.57 | NonCoding | -56.1 | 0.778 |
| candidate_806 | + | C | 97 | 20.77 | 3.07 | NonCoding | -18.6 | 0.368 |
| candidate_807 | + | C | 45 | 14.89 | 2.61 | NonCoding | -10.4 | 0.076 |
| candidate_808 | + | C | 39 | 50.9 | 1.79 | NonCoding | -10.9 | 0.237 |
| candidate_809 | + | C | 71 | 0.06 | 2.2 | NonCoding | -20.8 | 0.029 |
| candidate_810 | + | C | 62 | 0.22 | 2.84 | NonCoding | -22.1 | 0.088 |
| candidate_811 | + | C | 42 | 0.02 | 2.53 | NonCoding | -8.2 | 0.403 |
| candidate_812 | + | C | 34 | 0 | 2.36 | NonCoding | -14.4 | 0.189 |
| candidate_813 | + | C | 49 | 0 | 2.87 | NonCoding | -13.1 | 0.846 |
| candidate_814 | + | C | 37 | 0 | 2.08 | NonCoding | -16.7 | 0.06 |
| candidate_815 | + | C | 49 | 1.38 | 2.31 | NonCoding | -10.3 | 0.873 |
| candidate_816 | + | C | 208 | 2.76 | 3.09 | NonCoding | -83.4 | 0.329 |
| candidate_817 | + | C | 83 | 7.88 | 3.71 | NonCoding | -26.7 | 0.58 |
| candidate_818 | + | C | 41 | 5.38 | 2.21 | NonCoding | -11.3 | 0.648 |
| candidate_819 | + | C | 206 | 0 | 1.77 | NonCoding | -126.2 | 0.016 |
| candidate_820 | + | C | 152 | 0.21 | 2.49 | NonCoding | -54 | 0.043 |
| candidate_821 | + | C | 64 | 11.98 | 3.32 | NonCoding | -32.9 | 0.106 |
| candidate_822 | + | C | 41 | 4.79 | 3.26 | NonCoding | -14.1 | 0.511 |
| candidate_823 | + | C | 65 | 3.97 | 3.12 | NonCoding | -40.6 | 0.001 |
| candidate_824 | + | C | 72 | 14.59 | 4.44 | NonCoding | -29.2 | 0.368 |
| candidate_825 | + | C | 68 | 1.57 | 2.4 | NonCoding | -22.4 | 0.045 |
| candidate_826 | + | C | 213 | 0.13 | 2.38 | NonCoding | -144.15 | 0.001 |
| candidate_827 | + | C | 166 | 0 | 2.78 | NonCoding | -83.1 | 0.005 |
| candidate_828 | + | C | 56 | 0 | 3.11 | NonCoding | -25.8 | 0.315 |
| candidate_829 | + | C | 38 | 11.13 | 4.8 | NonCoding | -6.8 | 0.469 |
| candidate_830 | + | C | 40 | 5.66 | 2.27 | NonCoding | -12.4 | 0.283 |
| candidate_831 | + | C | 77 | 0 | 2.32 | NonCoding | -27.3 | 0.211 |
| candidate_832 | + | C | 39 | 0.53 | 1.8 | NonCoding | -22.3 | 0.091 |
| candidate_833 | + | C | 39 | 0.08 | 1.8 | NonCoding | -22.3 | 0.106 |
| candidate_834 | + | C | 39 | 0 | 1.8 | NonCoding | -22.3 | 0.117 |
| candidate_835 | + | C | 39 | 0 | 1.8 | NonCoding | -22.3 | 0.09 |
| candidate_836 | + | C | 39 | 1.15 | 1.8 | NonCoding | -22.3 | 0.096 |
| candidate_837 | + | C | 142 | 5.1 | 4.06 | NonCoding | -40.9 | 0.698 |
| candidate_838 | + | C | 45 | 0.15 | 1.77 | NonCoding | -23.1 | 0.018 |
| candidate_839 | + | C | 45 | 0 | 1.77 | NonCoding | -23.1 | 0.012 |
| candidate_840 | + | C | 69 | 8.27 | 1.77 | NonCoding | -24.7 | 0.061 |
| candidate_841 | + | C | 31 | 89.69 | 2.92 | NonCoding | -12.6 | 0.152 |
| candidate_842 | + | C | 66 | 130.54 | 2.58 | NonCoding | -23.3 | 0.066 |
| candidate_843 | + | C | 52 | 1 | 1.79 | NonCoding | -14.5 | 0.093 |
| candidate_844 | + | C | 60 | 29.46 | 2.55 | NonCoding | -15.3 | 0.256 |
| candidate_845 | + | C | 33 | 0.03 | 1.77 | NonCoding | -9 | 0.445 |
| candidate_846 | + | C | 58 | 0 | 3.19 | NonCoding | -27 | 0.02 |
| candidate_847 | + | C | 35 | 7.08 | 2.31 | NonCoding | -25.4 | 0.002 |
| candidate_848 | + | C | 44 | 4.2 | 2.3 | NonCoding | -21.8 | 0.202 |
| candidate_849 | + | C | 34 | 4.2 | 2.32 | NonCoding | -12.2 | 0.001 |
| candidate_850 | + | C | 46 | 0.87 | 2.34 | NonCoding | -17.71 | 0.258 |
| candidate_851 | + | C | 45 | 0.48 | 2.59 | NonCoding | -13.2 | 0.15 |
| candidate_852 | + | C | 136 | 6.52 | 4.62 | NonCoding | -67.5 | 0.286 |
| candidate_853 | + | C | 32 | 8.15 | 2.81 | NonCoding | -2.4 | 0.992 |
| candidate_854 | + | C | 32 | 1.73 | 1.78 | NonCoding | -11.4 | 0.427 |
| candidate_855 | + | C | 42 | 1.19 | 2.49 | NonCoding | -17.8 | 0.184 |
| candidate_856 | + | C | 41 | 0.31 | 2.17 | NonCoding | -17.1 | 0.212 |
| candidate_857 | + | C | 58 | 10.51 | 2.7 | NonCoding | -46.5 | 0.001 |
| candidate_858 | + | C | 60 | 13 | 3.23 | NonCoding | -38.6 | 0.001 |
| candidate_859 | + | C | 171 | 53.09 | 4.23 | NonCoding | -85.6 | 0.008 |
| candidate_860 | + | C | 98 | 9.35 | 2.55 | NonCoding | -29.9 | 0.583 |
| candidate_861 | + | C | 43 | 0 | 3.6 | NonCoding | -7.7 | 0.458 |
| candidate_862 | + | C | 30 | 5.06 | 3.93 | NonCoding | -1.4 | 0.644 |
| candidate_863 | + | C | 31 | 6.22 | 4.22 | NonCoding | -5.7 | 0.141 |
| candidate_864 | + | C | 82 | 31.34 | 4.5 | NonCoding | -17 | 0.362 |
| candidate_865 | + | C | 36 | 5 | 5.1 | NonCoding | -13.7 | 0.027 |
| candidate_866 | + | C | 33 | 2.47 | 3.68 | NonCoding | -11.9 | 0.06 |
| candidate_867 | + | C | 36 | 16.92 | 4.88 | NonCoding | -7.9 | 0.16 |
| candidate_868 | + | C | 92 | 9.53 | 3.09 | NonCoding | -28.7 | 0.026 |
| candidate_869 | + | C | 40 | 20.32 | 2.14 | NonCoding | -8.6 | 0.462 |
| candidate_870 | + | C | 52 | 3.34 | 3.04 | NonCoding | -21.5 | 0.025 |
| candidate_871 | + | C | 48 | 42.06 | 2.77 | NonCoding | -12.6 | 0.465 |
| candidate_872 | + | C | 39 | 4.23 | 2.4 | NonCoding | -6.5 | 0.504 |
| candidate_873 | + | C | 54 | 0 | 2.02 | NonCoding | -18.2 | 0.793 |
| candidate_874 | + | C | 55 | 28.45 | 1.79 | NonCoding | -12.9 | 0.881 |
| candidate_875 | + | C | 115 | 3.59 | 4.28 | NonCoding | -36.4 | 0.651 |
| candidate_876 | + | C | 34 | 3 | 3.04 | NonCoding | -1.7 | 0.892 |
| candidate_877 | + | C | 113 | 3.26 | 3.93 | NonCoding | -47.5 | 0.065 |
| candidate_878 | + | C | 48 | 11.45 | 2.47 | NonCoding | -8.8 | 0.473 |
| candidate_879 | + | C | 72 | 9.59 | 1.92 | NonCoding | -16.74 | 0.749 |
| candidate_880 | + | C | 48 | 3.84 | 3.24 | NonCoding | -17.3 | 0.754 |
| candidate_881 | + | C | 36 | 43.43 | 4.59 | NonCoding | -9.5 | 0.836 |
| candidate_882 | + | C | 45 | 0 | 1.8 | NonCoding | -18.2 | 0.151 |
| candidate_883 | + | C | 45 | 0 | 1.8 | NonCoding | -18.2 | 0.148 |
| candidate_884 | + | C | 54 | 0 | 2.48 | NonCoding | -18.2 | 0.35 |
| candidate_885 | + | C | 35 | 5.19 | 1.77 | NonCoding | -4.6 | 0.878 |
| candidate_886 | + | C | 41 | 0.62 | 2.44 | NonCoding | -9.6 | 0.896 |
| candidate_887 | + | C | 35 | 0.22 | 1.79 | NonCoding | -6.1 | 0.757 |
| candidate_888 | + | C | 32 | 23.03 | 2.33 | NonCoding | -8 | 0.241 |
| candidate_889 | + | C | 73 | 4.77 | 2.32 | NonCoding | -16 | 0.254 |
| candidate_890 | + | C | 31 | 0.78 | 4.33 | NonCoding | -0.2 | 0.535 |
| candidate_891 | + | C | 99 | 0.54 | 2.56 | NonCoding | -42.5 | 0.331 |
| candidate_892 | - | A | 41 | 190.43 | 1.25 | NonCoding | -2.6 | 0.768 |
| candidate_893 | - | A | 92 | 226.61 | 0 | AntiSense | -18.34 | 0.731 |
| candidate_894 | - | A | 78 | 351.43 | 0 | AntiSense | -9.17 | 0.905 |
| candidate_895 | - | A | 108 | 301.3 | 0 | AntiSense | -39.1 | 0.492 |
| candidate_896 | - | A | 49 | 206.1 | 0 | AntiSense | -13.1 | 0.509 |
| candidate_897 | - | A | 41 | 97.81 | 0 | AntiSense | -3 | 0.549 |
| candidate_898 | - | A | 54 | 289.67 | 0 | AntiSense | -14.7 | 0.83 |
| candidate_899 | - | A | 192 | 843.97 | 0 | AntiSense | -67 | 0.227 |
| candidate_900 | - | A | 105 | 325.66 | 0.1 | 5'/3'-UTR | -39.6 | 0.121 |
| candidate_901 | - | A | 37 | 116.42 | 0.14 | NonCoding | -13.1 | 0.463 |
| candidate_902 | - | A | 42 | 546.67 | 0 | AntiSense | -13.9 | 0.036 |
| candidate_903 | - | A | 61 | 537.05 | 0.64 | NonCoding | -6.4 | 0.837 |
| candidate_904 | - | A | 32 | 394.39 | 0.13 | NonCoding | -3 | 0.379 |
| candidate_905 | - | A | 75 | 549.45 | 2.84 | 5'/3'-UTR | -29.6 | 0.018 |
| candidate_906 | - | A | 51 | 287.77 | 0.08 | 5'/3'-UTR | -10.4 | 0.635 |
| candidate_907 | - | A | 66 | 420.69 | 0 | AntiSense | -16.9 | 0.929 |
| candidate_908 | - | A | 155 | 170.47 | 0 | AntiSense | -43.7 | 0.995 |
| candidate_909 | - | A | 44 | 140.24 | 0 | AntiSense | -7.5 | 0.677 |
| candidate_910 | - | A | 104 | 178.95 | 0 | AntiSense | -38.5 | 0.254 |
| candidate_911 | - | A | 160 | 164.42 | 0.05 | NonCoding | -60.3 | 0.411 |
| candidate_912 | - | A | 40 | 114.9 | 0 | AntiSense | -7.4 | 0.949 |
| candidate_913 | - | A | 46 | 427.66 | 0 | AntiSense | -5.7 | 0.459 |
| candidate_914 | - | A | 56 | 208.91 | 0 | AntiSense | -5.34 | 0.535 |
| candidate_915 | - | A | 47 | 119.33 | 0 | AntiSense | -13 | 0.732 |
| candidate_916 | - | A | 37 | 173.66 | 0 | AntiSense | -6.4 | 0.588 |
| candidate_917 | - | A | 51 | 177.56 | 0 | AntiSense | -11.2 | 0.839 |
| candidate_918 | - | A | 46 | 93.85 | 0 | AntiSense | -10 | 0.761 |
| candidate_919 | - | A | 116 | 141.34 | 0 | AntiSense | -40.5 | 0.747 |
| candidate_920 | - | A | 107 | 624.9 | 0 | AntiSense | -36.81 | 0.273 |
| candidate_921 | - | A | 32 | 74.24 | 0.13 | NonCoding | -5 | 0.759 |
| candidate_922 | - | A | 43 | 229.39 | 0 | AntiSense | -11.3 | 0.23 |
| candidate_923 | - | A | 32 | 160.73 | 0 | AntiSense | -6.9 | 0.546 |
| candidate_924 | - | A | 51 | 142.31 | 0 | AntiSense | -13.1 | 0.139 |
| candidate_925 | - | A | 46 | 1451.79 | 0.81 | NonCoding | -36.3 | 0.001 |
| candidate_926 | - | A | 31 | 274.91 | 0 | AntiSense | -8.7 | 0.284 |
| candidate_927 | - | A | 32 | 77.48 | 0 | AntiSense | -1.8 | 0.974 |
| candidate_928 | - | A | 86 | 798.33 | 0 | AntiSense | -19.4 | 0.322 |
| candidate_929 | - | A | 67 | 514.24 | 1.71 | NonCoding | -17.8 | 0.388 |
| candidate_930 | - | A | 117 | 360.82 | 0.04 | 5'/3'-UTR | -43.6 | 0.579 |
| candidate_931 | - | A | 32 | 100.21 | 0 | AntiSense | -1.2 | 0.95 |
| candidate_932 | - | A | 106 | 416.89 | 0 | AntiSense | -31.6 | 0.905 |
| candidate_933 | - | A | 44 | 148.16 | 0 | AntiSense | -4.5 | 0.894 |
| candidate_934 | - | A | 39 | 127.55 | 0 | AntiSense | -5.1 | 0.992 |
| candidate_935 | - | A | 93 | 568.31 | 0 | AntiSense | -31.3 | 0.298 |
| candidate_936 | - | A | 76 | 100.52 | 0 | AntiSense | -16.7 | 0.672 |
| candidate_937 | - | A | 31 | 112.91 | 0 | AntiSense | -6.8 | 0.177 |
| candidate_938 | - | A | 88 | 130.72 | 0 | AntiSense | -25.3 | 0.362 |
| candidate_939 | - | A | 30 | 98.9 | 0 | AntiSense | -3.6 | 0.784 |
| candidate_940 | - | A | 30 | 213.71 | 0 | AntiSense | -9.6 | 0.107 |
| candidate_941 | - | A | 182 | 95.14 | 0 | AntiSense | -80.9 | 0.14 |
| candidate_942 | - | A | 58 | 214.56 | 0.19 | NonCoding | -19.3 | 0.443 |
| candidate_943 | - | A | 107 | 343.54 | 0.41 | NonCoding | -32.9 | 0.65 |
| candidate_944 | - | A | 177 | 252.15 | 0.05 | 5'/3'-UTR | -52.33 | 0.66 |
| candidate_945 | - | A | 129 | 220.11 | 0 | AntiSense | -39.5 | 0.722 |
| candidate_946 | - | A | 166 | 758.77 | 0.06 | 5'/3'-UTR | -69.5 | 0.073 |
| candidate_947 | - | A | 32 | 91.52 | 0 | AntiSense | -2.5 | 0.798 |
| candidate_948 | - | A | 75 | 1532.64 | 0 | AntiSense | -9.7 | 0.989 |
| candidate_949 | - | A | 34 | 564.34 | 0 | AntiSense | -27.6 | 0.001 |
| candidate_950 | - | A | 73 | 197.19 | 0 | AntiSense | -22.8 | 0.551 |
| candidate_951 | - | A | 66 | 639.75 | 0.98 | NonCoding | -16.76 | 0.319 |
| candidate_952 | - | A | 41 | 156.48 | 0 | AntiSense | -6.3 | 0.961 |
| candidate_953 | - | A | 39 | 383.45 | 0 | AntiSense | -13.2 | 0.077 |
| candidate_954 | - | A | 34 | 268.8 | 1.59 | NonCoding | -7.7 | 0.241 |
| candidate_955 | - | A | 32 | 93.03 | 0 | AntiSense | -7.7 | 0.62 |
| candidate_956 | - | A | 54 | 97.18 | 0 | AntiSense | -13.8 | 0.582 |
| candidate_957 | - | A | 75 | 188.91 | 0.14 | NonCoding | -18.9 | 0.752 |
| candidate_958 | - | A | 31 | 113 | 0.53 | NonCoding | -2 | 0.438 |
| candidate_959 | - | A | 37 | 144.24 | 0 | AntiSense | -4 | 0.994 |
| candidate_960 | - | A | 31 | 131.78 | 0 | AntiSense | -2.5 | 0.955 |
| candidate_961 | - | A | 103 | 140.16 | 0 | AntiSense | -35.3 | 0.387 |
| candidate_962 | - | A | 32 | 1559.24 | 0.13 | NonCoding | -5.04 | 0.734 |
| candidate_963 | - | A | 38 | 187.44 | 0 | AntiSense | -6.6 | 0.876 |
| candidate_964 | - | A | 62 | 131.19 | 0.02 | NonCoding | -13.1 | 0.732 |
| candidate_965 | - | A | 113 | 274.1 | 0.07 | NonCoding | -39.4 | 0.751 |
| candidate_966 | - | A | 77 | 155.1 | 0 | AntiSense | -10.28 | 0.507 |
| candidate_967 | - | A | 377 | 555.98 | 0.17 | 5'/3'-UTR | -145.7 | 0.963 |
| candidate_968 | - | A | 37 | 123.37 | 0 | AntiSense | -8.4 | 0.478 |
| candidate_969 | - | A | 33 | 105.06 | 0 | AntiSense | -8.3 | 0.642 |
| candidate_970 | - | A | 121 | 155.58 | 0 | AntiSense | -41.4 | 0.685 |
| candidate_971 | - | A | 73 | 331.34 | 0 | AntiSense | -15.8 | 0.977 |
| candidate_972 | - | A | 36 | 127.08 | 0 | AntiSense | -7.3 | 0.515 |
| candidate_973 | - | A | 83 | 980.38 | 0 | AntiSense | -22.6 | 0.447 |
| candidate_974 | - | A | 136 | 320.76 | 0 | AntiSense | -36.9 | 0.829 |
| candidate_975 | - | A | 45 | 247.11 | 0 | AntiSense | -15.2 | 0.35 |
| candidate_976 | - | A | 66 | 84.66 | 0.25 | 5'/3'-UTR | -21 | 0.298 |
| candidate_977 | - | A | 42 | 189.19 | 0 | AntiSense | -6.2 | 0.585 |
| candidate_978 | - | A | 48 | 137.02 | 0 | AntiSense | -7.3 | 0.711 |
| candidate_979 | - | A | 59 | 149.02 | 0.05 | 5'/3'-UTR | -16.8 | 0.677 |
| candidate_980 | - | A | 43 | 340.3 | 0.07 | 5'/3'-UTR | -8.2 | 0.044 |
| candidate_981 | - | A | 47 | 111.69 | 0 | AntiSense | -11.3 | 0.154 |
| candidate_982 | - | A | 31 | 248.56 | 0 | AntiSense | -0.3 | 0.807 |
| candidate_983 | - | A | 64 | 1612.91 | 0 | AntiSense | -8.9 | 0.935 |
| candidate_984 | - | A | 46 | 507.91 | 0.14 | NonCoding | -9.2 | 0.242 |
| candidate_985 | - | A | 34 | 135.34 | 0.44 | NonCoding | -3.1 | 0.29 |
| candidate_986 | - | A | 44 | 501.6 | 0 | AntiSense | -10.7 | 0.522 |
| candidate_987 | - | A | 87 | 315.08 | 0 | AntiSense | -20 | 0.913 |
| candidate_988 | - | A | 35 | 63.72 | 0 | AntiSense | -30.6 | 0.001 |
| candidate_989 | - | A | 36 | 557.19 | 1.25 | NonCoding | -8.6 | 0.309 |
| candidate_990 | - | A | 38 | 255.77 | 0.09 | NonCoding | -4.2 | 0.898 |
| candidate_991 | - | A | 35 | 79 | 0 | AntiSense | -8.7 | 0.535 |
| candidate_992 | - | A | 33 | 103.35 | 0.23 | NonCoding | -4.5 | 0.443 |
| candidate_993 | - | A | 63 | 1379.97 | 0.07 | NonCoding | -9.8 | 0.964 |
| candidate_994 | - | A | 41 | 126.14 | 0.04 | 5'/3'-UTR | -7.1 | 0.632 |
| candidate_995 | - | A | 121 | 352.27 | 0.01 | 5'/3'-UTR | -36.8 | 0.312 |
| candidate_996 | - | A | 105 | 123.48 | 0.02 | 5'/3'-UTR | -27.6 | 0.557 |
| candidate_997 | - | A | 125 | 144.42 | 0 | AntiSense | -56.7 | 0.611 |
| candidate_998 | - | A | 77 | 144.46 | 0 | AntiSense | -14.14 | 0.919 |
| candidate_999 | - | A | 40 | 176.68 | 0 | AntiSense | -8.2 | 0.04 |
| candidate_1000 | - | A | 144 | 278.56 | 0 | AntiSense | -43.7 | 0.837 |
| candidate_1001 | - | A | 57 | 167.45 | 0 | AntiSense | -15.1 | 0.719 |
| candidate_1002 | - | A | 33 | 105.24 | 0 | AntiSense | -6.4 | 0.545 |
| candidate_1003 | - | A | 47 | 304.15 | 0 | AntiSense | -10.7 | 0.697 |
| candidate_1004 | - | A | 52 | 454.75 | 0 | AntiSense | -12.5 | 0.601 |
| candidate_1005 | - | A | 40 | 132.34 | 0.03 | 5'/3'-UTR | -6.7 | 0.918 |
| candidate_1006 | - | A | 32 | 82.76 | 0 | AntiSense | -1.8 | 0.596 |
| candidate_1007 | - | A | 42 | 227.98 | 0 | AntiSense | -5.3 | 0.759 |
| candidate_1008 | - | A | 148 | 234.07 | 0 | AntiSense | -64.6 | 0.802 |
| candidate_1009 | - | A | 30 | 439.48 | 0 | AntiSense | -5.4 | 0.75 |
| candidate_1010 | - | A | 45 | 164.11 | 0 | AntiSense | -8.7 | 0.615 |
| candidate_1011 | - | A | 137 | 252.55 | 0 | AntiSense | -56.5 | 0.471 |
| candidate_1012 | - | A | 156 | 239.03 | 0 | AntiSense | -59.1 | 0.563 |
| candidate_1013 | - | A | 32 | 581.36 | 0.05 | 5'/3'-UTR | -2.4 | 0.9 |
| candidate_1014 | - | A | 87 | 141.66 | 0.1 | 5'/3'-UTR | -25.5 | 0.047 |
| candidate_1015 | - | A | 107 | 391.75 | 0 | AntiSense | -35.4 | 0.789 |
| candidate_1016 | - | A | 198 | 1084.05 | 1.02 | 5'/3'-UTR | -76 | 0.849 |
| candidate_1017 | - | A | 126 | 172.72 | 0.01 | 5'/3'-UTR | -53.5 | 0.164 |
| candidate_1018 | - | A | 40 | 96.02 | 0 | AntiSense | -14.4 | 0.394 |
| candidate_1019 | - | A | 52 | 100.6 | 0 | AntiSense | -20.2 | 0.148 |
| candidate_1020 | - | A | 207 | 355.92 | 0.13 | NonCoding | -75.2 | 0.981 |
| candidate_1021 | - | A | 38 | 120.67 | 0 | AntiSense | -3.9 | 0.983 |
| candidate_1022 | - | A | 36 | 1016.14 | 0.09 | NonCoding | -10.3 | 0.229 |
| candidate_1023 | - | A | 32 | 126.36 | 0 | AntiSense | -1.9 | 0.874 |
| candidate_1024 | - | A | 33 | 1996.5 | 0.14 | NonCoding | -26.2 | 0.001 |
| candidate_1025 | - | A | 34 | 253.46 | 0.14 | NonCoding | 0 | 1 |
| candidate_1026 | - | A | 44 | 142.96 | 0 | AntiSense | -5.79 | 0.285 |
| candidate_1027 | - | A | 97 | 192.11 | 0.02 | 5'/3'-UTR | -29.6 | 0.96 |
| candidate_1028 | - | A | 161 | 567.88 | 0.04 | 5'/3'-UTR | -52.4 | 0.21 |
| candidate_1029 | - | A | 395 | 606.41 | 0 | 5'/3'-UTR | -146.8 | 0.61 |
| candidate_1030 | - | A | 33 | 185.88 | 0 | AntiSense | -7.1 | 0.505 |
| candidate_1031 | - | A | 76 | 270.55 | 0.05 | 5'/3'-UTR | -10.1 | 0.867 |
| candidate_1032 | - | A | 170 | 209.7 | 0 | AntiSense | -60.3 | 0.523 |
| candidate_1033 | - | A | 34 | 146.97 | 0.08 | NonCoding | -2.5 | 0.622 |
| candidate_1034 | - | A | 41 | 510.29 | 0.08 | NonCoding | -15.7 | 0.031 |
| candidate_1035 | - | A | 35 | 106.44 | 1.65 | NonCoding | -23.3 | 0.001 |
| candidate_1036 | - | A | 32 | 326.36 | 0.07 | 5'/3'-UTR | -5.6 | 0.724 |
| candidate_1037 | - | A | 91 | 152.14 | 0.01 | 5'/3'-UTR | -23.6 | 0.264 |
| candidate_1038 | - | A | 101 | 195.06 | 0 | AntiSense | -28.1 | 0.712 |
| candidate_1039 | - | A | 60 | 168.13 | 1.41 | 5'/3'-UTR | -22.7 | 0.078 |
| candidate_1040 | - | A | 34 | 139.63 | 0 | AntiSense | -6.7 | 0.789 |
| candidate_1041 | - | A | 32 | 156.36 | 0.57 | NonCoding | -7.4 | 0.37 |
| candidate_1042 | - | A | 146 | 179.44 | 0.3 | 5'/3'-UTR | -47.5 | 0.727 |
| candidate_1043 | - | A | 310 | 490.53 | 0.22 | 5'/3'-UTR | -106.2 | 0.482 |
| candidate_1044 | - | A | 33 | 110.38 | 0 | AntiSense | -4.9 | 0.623 |
| candidate_1045 | - | A | 31 | 112.19 | 0 | AntiSense | -11.8 | 0.015 |
| candidate_1046 | - | A | 89 | 172.56 | 0 | 5'/3'-UTR | -21.7 | 0.199 |
| candidate_1047 | - | A | 74 | 120.85 | 0 | 5'/3'-UTR | -26.5 | 0.597 |
| candidate_1048 | - | A | 82 | 124.99 | 0 | AntiSense | -20 | 0.89 |
| candidate_1049 | - | A | 80 | 618.64 | 0.28 | 5'/3'-UTR | -26.1 | 0.144 |
| candidate_1050 | - | A | 104 | 338.3 | 0 | AntiSense | -35.3 | 0.4 |
| candidate_1051 | - | A | 37 | 122.58 | 0.12 | 5'/3'-UTR | -0.6 | 0.999 |
| candidate_1052 | - | A | 246 | 310.77 | 0 | 5'/3'-UTR | -99.3 | 0.034 |
| candidate_1053 | - | A | 127 | 343.35 | 0 | 5'/3'-UTR | -44 | 0.482 |
| candidate_1054 | - | A | 233 | 1698.39 | 0 | 5'/3'-UTR | -87.01 | 0.3 |
| candidate_1055 | - | A | 75 | 114.38 | 0.9 | 5'/3'-UTR | -39.4 | 0.002 |
| candidate_1056 | - | A | 171 | 163.52 | 0 | AntiSense | -63.3 | 0.832 |
| candidate_1057 | - | A | 30 | 106.94 | 0 | AntiSense | -1.14 | 0.448 |
| candidate_1058 | - | A | 31 | 92.03 | 0.65 | NonCoding | -4.8 | 0.66 |
| candidate_1059 | - | A | 198 | 1195.92 | 0.2 | 5'/3'-UTR | -53.12 | 0.924 |
| candidate_1060 | - | A | 42 | 113.3 | 0 | AntiSense | -9.9 | 0.492 |
| candidate_1061 | - | A | 34 | 120.34 | 0 | AntiSense | -4.1 | 0.83 |
| candidate_1062 | - | A | 68 | 202.88 | 0 | AntiSense | -27.2 | 0.245 |
| candidate_1063 | - | A | 43 | 155.32 | 0.14 | NonCoding | -4 | 0.184 |
| candidate_1064 | - | A | 95 | 200.16 | 0 | AntiSense | -27 | 0.939 |
| candidate_1065 | - | A | 99 | 171.7 | 0 | AntiSense | -38.8 | 0.298 |
| candidate_1066 | - | A | 38 | 72.08 | 0 | AntiSense | -8.9 | 0.429 |
| candidate_1067 | - | A | 40 | 225.12 | 0 | 5'/3'-UTR | -8.9 | 0.071 |
| candidate_1068 | - | A | 43 | 246.23 | 0 | AntiSense | -10.1 | 0.186 |
| candidate_1069 | - | A | 84 | 297.59 | 0 | AntiSense | -27.6 | 0.739 |
| candidate_1070 | - | A | 68 | 165.41 | 0 | AntiSense | -23.9 | 0.05 |
| candidate_1071 | - | A | 55 | 396.54 | 0 | AntiSense | -14.6 | 0.439 |
| candidate_1072 | - | A | 78 | 158.42 | 0 | AntiSense | -28.1 | 0.549 |
| candidate_1073 | - | A | 94 | 220.97 | 0.09 | 5'/3'-UTR | -26.4 | 0.812 |
| candidate_1074 | - | A | 49 | 233.2 | 4.63 | NonCoding | -10.4 | 0.43 |
| candidate_1075 | - | A | 216 | 804.86 | 2.36 | NonCoding | -81.2 | 0.354 |
| candidate_1076 | - | A | 38 | 117.38 | 0 | AntiSense | -7 | 0.957 |
| candidate_1077 | - | A | 47 | 1384.17 | 0.53 | NonCoding | -4.6 | 0.666 |
| candidate_1078 | - | A | 35 | 197.08 | 0 | AntiSense | -11.7 | 0.252 |
| candidate_1079 | - | A | 40 | 96.05 | 0.14 | NonCoding | -7.1 | 0.398 |
| candidate_1080 | - | A | 283 | 668.53 | 0.07 | 5'/3'-UTR | -123.01 | 0.048 |
| candidate_1081 | - | A | 42 | 95.7 | 0 | AntiSense | -11.7 | 0.223 |
| candidate_1082 | - | A | 54 | 230.31 | 3.65 | NonCoding | -8.3 | 0.914 |
| candidate_1083 | - | A | 34 | 67.83 | 0.92 | NonCoding | -8.7 | 0.53 |
| candidate_1084 | - | A | 41 | 681.43 | 0.08 | 5'/3'-UTR | -9.5 | 0.748 |
| candidate_1085 | - | A | 182 | 522.15 | 0 | AntiSense | -59.1 | 0.732 |
| candidate_1086 | - | A | 109 | 460.75 | 0.14 | NonCoding | -40.8 | 0.27 |
| candidate_1087 | - | A | 55 | 135.73 | 0 | AntiSense | -17.3 | 0.463 |
| candidate_1088 | - | A | 30 | 134.61 | 0 | AntiSense | -6.5 | 0.452 |
| candidate_1089 | - | A | 70 | 104.08 | 0 | AntiSense | -17.4 | 0.246 |
| candidate_1090 | - | A | 38 | 312.21 | 0 | AntiSense | -5.2 | 0.777 |
| candidate_1091 | - | A | 52 | 314.02 | 0 | AntiSense | -8.8 | 0.766 |
| candidate_1092 | - | A | 44 | 377.29 | 0.04 | 5'/3'-UTR | -6.83 | 0.308 |
| candidate_1093 | - | A | 76 | 152.4 | 0.02 | 5'/3'-UTR | -17.6 | 0.288 |
| candidate_1094 | - | A | 65 | 165.17 | 0.02 | 5'/3'-UTR | -11.2 | 0.397 |
| candidate_1095 | - | A | 84 | 101.4 | 0 | AntiSense | -25.8 | 0.468 |
| candidate_1096 | - | A | 36 | 226.81 | 0.14 | NonCoding | -4.1 | 0.955 |
| candidate_1097 | - | A | 55 | 212.54 | 0 | AntiSense | -14.3 | 0.67 |
| candidate_1098 | - | A | 78 | 887.41 | 1.42 | NonCoding | -18 | 0.624 |
| candidate_1099 | - | A | 64 | 192.8 | 0.06 | 5'/3'-UTR | -6.8 | 0.995 |
| candidate_1100 | - | A | 43 | 92.2 | 0 | AntiSense | -8 | 0.497 |
| candidate_1101 | - | A | 97 | 181.59 | 0 | 5'/3'-UTR | -27.4 | 0.68 |
| candidate_1102 | - | A | 57 | 375.93 | 0 | AntiSense | -11.16 | 0.933 |
| candidate_1103 | - | A | 51 | 121.19 | 0 | AntiSense | -12.32 | 0.37 |
| candidate_1104 | - | A | 37 | 90.58 | 0 | AntiSense | -5.2 | 0.749 |
| candidate_1105 | - | A | 36 | 83.24 | 0.12 | 5'/3'-UTR | -5.2 | 0.308 |
| candidate_1106 | - | A | 32 | 170.79 | 0 | AntiSense | -13 | 0.024 |
| candidate_1107 | - | A | 33 | 265.97 | 0 | AntiSense | -5 | 0.834 |
| candidate_1108 | - | A | 71 | 223.4 | 0 | AntiSense | -15.9 | 0.716 |
| candidate_1109 | - | A | 43 | 130.18 | 0 | AntiSense | -12 | 0.342 |
| candidate_1110 | - | A | 33 | 181.35 | 0 | AntiSense | -9.8 | 0.669 |
| candidate_1111 | - | A | 101 | 240.5 | 0.03 | 5'/3'-UTR | -34.9 | 0.211 |
| candidate_1112 | - | A | 43 | 232.89 | 0 | AntiSense | -7.2 | 0.679 |
| candidate_1113 | - | A | 35 | 104.72 | 0 | AntiSense | -4.5 | 0.81 |
| candidate_1114 | - | A | 30 | 207.23 | 0.14 | NonCoding | -2.3 | 0.693 |
| candidate_1115 | - | A | 46 | 1042.45 | 2.22 | NonCoding | -7.1 | 0.951 |
| candidate_1116 | - | A | 76 | 794.65 | 0.01 | 5'/3'-UTR | -21.3 | 0.382 |
| candidate_1117 | - | A | 173 | 218.98 | 0 | AntiSense | -74.6 | 0.07 |
| candidate_1118 | - | A | 162 | 777.87 | 1.07 | NonCoding | -39.74 | 0.496 |
| candidate_1119 | - | A | 31 | 70.16 | 0.48 | NonCoding | -8.3 | 0.495 |
| candidate_1120 | - | A | 94 | 220.62 | 0 | AntiSense | -33.6 | 0.213 |
| candidate_1121 | - | A | 107 | 321.39 | 0 | AntiSense | -44.9 | 0.055 |
| candidate_1122 | - | A | 30 | 101.06 | 0.11 | NonCoding | -5.8 | 0.366 |
| candidate_1123 | - | A | 83 | 215.8 | 0.08 | NonCoding | -21.8 | 0.626 |
| candidate_1124 | - | A | 127 | 168.67 | 0 | AntiSense | -43.5 | 0.149 |
| candidate_1125 | - | A | 52 | 138.85 | 0.09 | 5'/3'-UTR | -9.5 | 0.862 |
| candidate_1126 | - | A | 95 | 526.44 | 1.15 | NonCoding | -35 | 0.02 |
| candidate_1127 | - | A | 38 | 255.9 | 0 | AntiSense | -13.8 | 0.094 |
| candidate_1128 | - | A | 35 | 149.67 | 0.14 | NonCoding | -8 | 0.246 |
| candidate_1129 | - | A | 229 | 349.37 | 0 | AntiSense | -78.7 | 0.275 |
| candidate_1130 | - | A | 51 | 269.79 | 0 | AntiSense | -12.9 | 0.6 |
| candidate_1131 | - | A | 50 | 78.02 | 0 | AntiSense | -16.9 | 0.087 |
| candidate_1132 | - | A | 76 | 189.78 | 0.12 | NonCoding | -25.5 | 0.765 |
| candidate_1133 | - | A | 37 | 172.74 | 3.29 | NonCoding | -10.7 | 0.712 |
| candidate_1134 | - | A | 43 | 288.52 | 2.3 | NonCoding | -10.2 | 0.213 |
| candidate_1135 | - | A | 42 | 312.81 | 0.1 | NonCoding | -9.5 | 0.731 |
| candidate_1136 | - | A | 32 | 73.21 | 0.91 | NonCoding | -6.8 | 0.503 |
| candidate_1137 | - | A | 62 | 1042.76 | 0.1 | NonCoding | -34.34 | 0.001 |
| candidate_1138 | - | A | 33 | 117.35 | 0.1 | NonCoding | -6.2 | 0.149 |
| candidate_1139 | - | A | 98 | 178.58 | 0.14 | NonCoding | -27.8 | 0.86 |
| candidate_1140 | - | A | 39 | 121.15 | 0.07 | 5'/3'-UTR | -14.3 | 0.008 |
| candidate_1141 | - | A | 92 | 228.33 | 0 | AntiSense | -41.6 | 0.354 |
| candidate_1142 | - | A | 272 | 633.01 | 0 | AntiSense | -113.2 | 0.685 |
| candidate_1143 | - | A | 78 | 403.96 | 0.14 | NonCoding | -14.91 | 0.331 |
| candidate_1144 | - | A | 40 | 254 | 2.9 | 5'/3'-UTR | -10.9 | 0.398 |
| candidate_1145 | - | A | 39 | 190.03 | 0.14 | NonCoding | -9.9 | 0.433 |
| candidate_1146 | - | A | 52 | 275.74 | 0 | AntiSense | -10.9 | 0.904 |
| candidate_1147 | - | A | 170 | 613.7 | 0 | AntiSense | -67.91 | 0.909 |
| candidate_1148 | - | A | 31 | 114.53 | 0 | AntiSense | -4.1 | 0.571 |
| candidate_1149 | - | A | 35 | 96.42 | 1.34 | NonCoding | -13.5 | 0.09 |
| candidate_1150 | - | A | 327 | 515.39 | 0.09 | 5'/3'-UTR | -111.7 | 0.965 |
| candidate_1151 | - | A | 57 | 5034.5 | 1.37 | NonCoding | -34.4 | 0.001 |
| candidate_1152 | - | A | 60 | 235.28 | 0 | AntiSense | -11.1 | 0.818 |
| candidate_1153 | - | A | 30 | 1368.77 | 0 | AntiSense | -2.3 | 0.682 |
| candidate_1154 | - | A | 238 | 559.48 | 0 | AntiSense | -106 | 0.141 |
| candidate_1155 | - | A | 86 | 147.26 | 0 | AntiSense | -19.5 | 0.916 |
| candidate_1156 | - | A | 37 | 205.68 | 0.14 | NonCoding | -1.8 | 0.902 |
| candidate_1157 | - | A | 51 | 226.92 | 0.14 | NonCoding | -16.1 | 0.319 |
| candidate_1158 | - | A | 58 | 393.61 | 0 | AntiSense | -16.6 | 0.224 |
| candidate_1159 | - | A | 33 | 77.24 | 0.14 | NonCoding | -8.3 | 0.343 |
| candidate_1160 | - | A | 93 | 206.48 | 0.1 | 5'/3'-UTR | -49.3 | 0.001 |
| candidate_1161 | - | A | 39 | 708.93 | 0 | AntiSense | -8.3 | 0.646 |
| candidate_1162 | - | A | 45 | 102.67 | 0 | AntiSense | -9 | 0.851 |
| candidate_1163 | - | A | 96 | 178.64 | 0 | AntiSense | -28.7 | 0.332 |
| candidate_1164 | - | A | 39 | 119.2 | 0 | AntiSense | -10.6 | 0.395 |
| candidate_1165 | - | A | 39 | 122.63 | 0 | AntiSense | -9.4 | 0.643 |
| candidate_1166 | - | A | 47 | 98.88 | 0 | AntiSense | -13.7 | 0.641 |
| candidate_1167 | - | A | 30 | 119.77 | 0 | AntiSense | -2.4 | 0.784 |
| candidate_1168 | - | A | 32 | 132.45 | 0 | AntiSense | -6.1 | 0.578 |
| candidate_1169 | - | A | 50 | 2013.59 | 1.18 | NonCoding | -11.1 | 0.408 |
| candidate_1170 | - | A | 52 | 261.66 | 0 | AntiSense | -10.5 | 0.91 |
| candidate_1171 | - | A | 186 | 361.66 | 0 | AntiSense | -74.2 | 0.738 |
| candidate_1172 | - | A | 31 | 124.81 | 0 | AntiSense | -9 | 0.206 |
| candidate_1173 | - | A | 129 | 259.63 | 0.01 | 5'/3'-UTR | -27.6 | 0.968 |
| candidate_1174 | - | A | 41 | 95.83 | 0 | AntiSense | 0 | 1 |
| candidate_1175 | - | A | 40 | 209.37 | 0 | AntiSense | -9.4 | 0.704 |
| candidate_1176 | - | A | 40 | 183.85 | 0 | AntiSense | -5.6 | 0.891 |
| candidate_1177 | - | A | 118 | 194.85 | 0.01 | 5'/3'-UTR | -32.1 | 0.388 |
| candidate_1178 | - | A | 30 | 99.87 | 0.51 | NonCoding | -4.2 | 0.318 |
| candidate_1179 | - | A | 52 | 168.51 | 0 | AntiSense | -11.8 | 0.752 |
| candidate_1180 | - | A | 98 | 634.85 | 0 | AntiSense | -26 | 0.858 |
| candidate_1181 | - | A | 52 | 150.72 | 0 | AntiSense | -11.9 | 0.734 |
| candidate_1182 | - | A | 256 | 492.96 | 0 | AntiSense | -103.02 | 0.675 |
| candidate_1183 | - | A | 72 | 3592.34 | 3.33 | NonCoding | -13.6 | 0.369 |
| candidate_1184 | - | A | 181 | 716.74 | 0.02 | 5'/3'-UTR | -40.3 | 0.837 |
| candidate_1186 | - | A | 235 | 186.6 | 0 | AntiSense | -349.9 | 0.565 |
| candidate_1187 | - | A | 425 | 274.01 | 1.09 | 5'/3'-UTR | -80.4 | 0.033 |
| candidate_1188 | - | A | 393 | 327.06 | 0 | AntiSense | -118.69 | 0.811 |
| candidate_1189 | - | A | 384 | 178.71 | 0 | AntiSense | -124.33 | 0.099 |
| candidate_1190 | - | A | 543 | 216.16 | 0 | AntiSense | -120.65 | 0.331 |
| candidate_1191 | - | A | 503 | 198.56 | 0 | AntiSense | -196.81 | 0.168 |
| candidate_1192 | - | A | 528 | 197.68 | 0 | AntiSense | -156.8 | 0.028 |
| candidate_1193 | - | A | 75 | 149.83 | 0 | AntiSense | -205.05 | 0.397 |
| candidate_1194 | - | A | 38 | 275.15 | 0 | 5'/3'-UTR | -16.06 | 0.785 |
| candidate_1195 | - | A | 74 | 439.51 | 0.14 | 5'/3'-UTR | -8.6 | 0.646 |
| candidate_1196 | - | A | 32 | 135.55 | 0 | AntiSense | -20 | 0.692 |
| candidate_1197 | - | A | 39 | 234.2 | 0.02 | 5'/3'-UTR | -4.8 | 0.059 |
| candidate_1198 | - | A | 160 | 430.51 | 0 | AntiSense | -14 | 0.698 |
| candidate_1199 | - | A | 34 | 207.34 | 1.65 | NonCoding | -57.5 | 0.001 |
| candidate_1200 | - | A | 30 | 126.1 | 0 | AntiSense | -26.5 | 0.501 |
| candidate_1201 | - | A | 37 | 128.55 | 0 | 5'/3'-UTR | -2 | 0.551 |
| candidate_1202 | - | A | 30 | 116.94 | 0 | AntiSense | -4 | 0.955 |
| candidate_1203 | - | A | 39 | 187.7 | 0.03 | 5'/3'-UTR | -5.1 | 0.603 |
| candidate_1204 | - | A | 107 | 204.93 | 0 | 5'/3'-UTR | -7.5 | 0.508 |
| candidate_1205 | - | A | 135 | 421.51 | 0.65 | NonCoding | -34.1 | 0.655 |
| candidate_1206 | - | A | 35 | 92.31 | 0 | AntiSense | -42.8 | 0.572 |
| candidate_1207 | - | A | 40 | 71.05 | 0 | AntiSense | -11.5 | 0.045 |
| candidate_1208 | - | A | 35 | 214.72 | 0 | AntiSense | -17.1 | 0.929 |
| candidate_1209 | - | A | 34 | 240.34 | 0 | AntiSense | -6.8 | 0.113 |
| candidate_1210 | - | A | 57 | 244.38 | 0 | AntiSense | -8.8 | 0.644 |
| candidate_1211 | - | A | 38 | 101.92 | 0 | AntiSense | -16 | 0.223 |
| candidate_1212 | - | A | 243 | 783.22 | 0.04 | 5'/3'-UTR | -10.2 | 0.091 |
| candidate_1213 | - | A | 46 | 197.85 | 0.09 | 5'/3'-UTR | -101.3 | 0.739 |
| candidate_1214 | - | A | 33 | 160.15 | 1.62 | NonCoding | -8.3 | 0.001 |
| candidate_1215 | - | A | 163 | 345.8 | 0.14 | NonCoding | -25.9 | 0.472 |
| candidate_1216 | - | A | 64 | 128.29 | 0 | 5'/3'-UTR | -30.2 | 0.765 |
| candidate_1217 | - | A | 45 | 376.8 | 0 | AntiSense | -17.3 | 0.474 |
| candidate_1218 | - | A | 38 | 112.79 | 0 | AntiSense | -10.4 | 0.643 |
| candidate_1219 | - | A | 78 | 176.14 | 0 | AntiSense | -7.5 | 0.916 |
| candidate_1220 | - | A | 44 | 83.42 | 0 | AntiSense | -15.1 | 0.401 |
| candidate_1221 | - | A | 30 | 146.39 | 0 | AntiSense | -11.3 | 0.148 |
| candidate_1222 | - | A | 73 | 250.68 | 0.06 | 5'/3'-UTR | -4.1 | 0.973 |
| candidate_1223 | - | A | 39 | 105.03 | 0 | AntiSense | -18.9 | 0.45 |
| candidate_1224 | - | A | 57 | 212.95 | 0 | 5'/3'-UTR | -14 | 0.117 |
| candidate_1225 | - | A | 59 | 2419.83 | 0.09 | 5'/3'-UTR | -15.8 | 0.236 |
| candidate_1226 | - | A | 52 | 153.92 | 0 | AntiSense | -14.2 | 0.444 |
| candidate_1227 | - | A | 38 | 113.44 | 0 | AntiSense | -11.4 | 0.349 |
| candidate_1228 | - | A | 31 | 91.13 | 0 | AntiSense | -6.5 | 0.007 |
| candidate_1229 | - | A | 38 | 125.67 | 0 | AntiSense | -10.8 | 0.601 |
| candidate_1230 | - | A | 52 | 641.77 | 0 | AntiSense | -7.5 | 0.1 |
| candidate_1231 | - | A | 53 | 220.2 | 0 | AntiSense | -17 | 0.644 |
| candidate_1232 | - | A | 34 | 96.69 | 0 | AntiSense | -7 | 0.791 |
| candidate_1233 | - | A | 143 | 441.82 | 0 | AntiSense | -7.1 | 0.963 |
| candidate_1234 | - | A | 119 | 203.63 | 0.27 | 5'/3'-UTR | -44.3 | 0.507 |
| candidate_1235 | - | A | 36 | 148.57 | 0 | AntiSense | -36.4 | 0.491 |
| candidate_1236 | - | A | 32 | 106.33 | 0 | AntiSense | -9.3 | 0.151 |
| candidate_1237 | - | A | 219 | 177.06 | 0 | AntiSense | -8.2 | 0.328 |
| candidate_1238 | - | A | 110 | 257.2 | 0 | AntiSense | -71.1 | 0.361 |
| candidate_1239 | - | A | 31 | 306.66 | 0 | 5'/3'-UTR | -37.2 | 0.602 |
| candidate_1240 | - | A | 128 | 494.61 | 0 | AntiSense | -5.5 | 0.878 |
| candidate_1241 | - | A | 34 | 136.4 | 0 | AntiSense | -43.8 | 0.654 |
| candidate_1242 | - | A | 32 | 365.97 | 0.14 | NonCoding | -9.9 | 0.063 |
| candidate_1243 | - | A | 46 | 116.55 | 0.11 | NonCoding | -9.3 | 0.054 |
| candidate_1244 | - | A | 150 | 533.28 | 0.13 | NonCoding | -16.9 | 0.764 |
| candidate_1245 | - | A | 149 | 137.84 | 0 | AntiSense | -54.7 | 0.879 |
| candidate_1246 | - | A | 97 | 176.3 | 0 | AntiSense | -57.7 | 0.847 |
| candidate_1247 | - | A | 33 | 344.47 | 0 | AntiSense | -39.1 | 0.404 |
| candidate_1248 | - | A | 53 | 210.02 | 0 | AntiSense | -8 | 0.23 |
| candidate_1249 | - | A | 31 | 107.19 | 0 | AntiSense | -10 | 0.001 |
| candidate_1250 | - | A | 45 | 182.89 | 0 | AntiSense | -25 | 0.663 |
| candidate_1251 | - | A | 30 | 580.03 | 0.12 | NonCoding | -14.5 | 0.632 |
| candidate_1252 | - | A | 41 | 217.52 | 0.28 | NonCoding | -5.8 | 0.551 |
| candidate_1253 | - | A | 158 | 224.04 | 0 | AntiSense | -7.5 | 0.099 |
| candidate_1254 | - | A | 36 | 190.03 | 1.38 | NonCoding | -56.1 | 0.001 |
| candidate_1255 | - | A | 178 | 310.91 | 0 | AntiSense | -25 | 0.516 |
| candidate_1256 | - | A | 72 | 1603.66 | 1.82 | 5'/3'-UTR | -75 | 0.094 |
| candidate_1257 | - | A | 30 | 106.9 | 0 | AntiSense | -25.3 | 0.661 |
| candidate_1258 | - | A | 49 | 397.64 | 0 | AntiSense | -4.3 | 0.615 |
| candidate_1259 | - | A | 44 | 158.76 | 0 | AntiSense | -10.5 | 0.255 |
| candidate_1260 | - | A | 97 | 477.37 | 0 | AntiSense | -11.4 | 0.672 |
| candidate_1261 | - | A | 64 | 223.72 | 0 | AntiSense | -28.2 | 0.915 |
| candidate_1262 | - | A | 41 | 116.83 | 0.08 | 5'/3'-UTR | -13.5 | 0.256 |
| candidate_1263 | - | A | 40 | 230.02 | 0 | AntiSense | -12.2 | 0.531 |
| candidate_1264 | - | A | 37 | 175.71 | 0.59 | 5'/3'-UTR | -10.7 | 0.829 |
| candidate_1265 | - | A | 30 | 77.97 | 0 | AntiSense | -5.2 | 0.25 |
| candidate_1266 | - | A | 57 | 127.62 | 0 | AntiSense | -5.7 | 0.198 |
| candidate_1267 | - | A | 31 | 138.66 | 0 | AntiSense | -17.5 | 0.868 |
| candidate_1268 | - | A | 49 | 231.12 | 0 | 5'/3'-UTR | -4.2 | 0.865 |
| candidate_1269 | - | A | 151 | 570.48 | 0.14 | NonCoding | -8.6 | 0.005 |
| candidate_1270 | - | A | 30 | 146.84 | 0 | AntiSense | -63.8 | 0.408 |
| candidate_1271 | - | A | 43 | 259.75 | 0 | AntiSense | -6.1 | 0.901 |
| candidate_1272 | - | A | 50 | 300.22 | 0 | AntiSense | -11.1 | 0.76 |
| candidate_1273 | - | A | 41 | 245.69 | 0 | AntiSense | -9.6 | 0.222 |
| candidate_1274 | - | A | 60 | 2126.07 | 0.1 | NonCoding | -10.36 | 0.136 |
| candidate_1275 | - | A | 30 | 84.97 | 0.14 | NonCoding | -21.8 | 0.666 |
| candidate_1276 | - | A | 144 | 154.97 | 0 | 5'/3'-UTR | -5.2 | 0.479 |
| candidate_1277 | - | A | 33 | 77.09 | 0 | AntiSense | -49.15 | 0.374 |
| candidate_1278 | - | A | 40 | 164.39 | 0 | AntiSense | -6.3 | 0.193 |
| candidate_1279 | - | A | 41 | 604.62 | 0.14 | 5'/3'-UTR | -10.7 | 0.859 |
| candidate_1280 | - | A | 38 | 131.44 | 0.14 | NonCoding | -2.5 | 0.759 |
| candidate_1281 | - | A | 160 | 394.37 | 0 | AntiSense | -0.2 | 0.812 |
| candidate_1282 | - | A | 31 | 199.56 | 0 | AntiSense | -49.9 | 0.711 |
| candidate_1283 | - | A | 149 | 176.5 | 0 | AntiSense | -3.2 | 0.852 |
| candidate_1284 | - | A | 32 | 442.76 | 0 | AntiSense | -52.7 | 0.709 |
| candidate_1285 | - | A | 86 | 202.45 | 0 | 5'/3'-UTR | -6.1 | 0.133 |
| candidate_1286 | - | A | 106 | 483.28 | 0.09 | 5'/3'-UTR | -28 | 0.439 |
| candidate_1287 | - | A | 41 | 96.57 | 0 | AntiSense | -24.7 | 0.327 |
| candidate_1288 | - | A | 34 | 661.2 | 0.14 | NonCoding | -8.4 | 0.398 |
| candidate_1289 | - | A | 57 | 1622.26 | 0.55 | 5'/3'-UTR | -3.8 | 0.412 |
| candidate_1290 | - | A | 90 | 963.56 | 1.11 | 5'/3'-UTR | -16.1 | 0.03 |
| candidate_1291 | - | A | 56 | 235.81 | 0 | AntiSense | -35.5 | 0.597 |
| candidate_1292 | - | A | 74 | 207.28 | 0 | AntiSense | -15.9 | 0.89 |
| candidate_1293 | - | A | 83 | 124.06 | 0 | AntiSense | -11.11 | 0.697 |
| candidate_1294 | - | A | 36 | 96 | 0 | AntiSense | -20.9 | 0.692 |
| candidate_1295 | - | A | 38 | 179.87 | 0 | AntiSense | -7.8 | 0.552 |
| candidate_1296 | - | A | 56 | 274.05 | 0 | AntiSense | -4.3 | 0.45 |
| candidate_1297 | - | A | 43 | 397.36 | 0.02 | 5'/3'-UTR | -16.1 | 0.828 |
| candidate_1298 | - | A | 47 | 111.96 | 0 | AntiSense | -6.5 | 0.233 |
| candidate_1299 | - | A | 38 | 102.82 | 0.14 | NonCoding | -13.1 | 0.549 |
| candidate_1300 | - | A | 56 | 180.86 | 0.08 | 5'/3'-UTR | -8.9 | 0.044 |
| candidate_1301 | - | A | 82 | 184.47 | 0.14 | NonCoding | -20.3 | 0.865 |
| candidate_1302 | - | A | 30 | 682.35 | 0.14 | NonCoding | -28 | 0.684 |
| candidate_1303 | - | A | 218 | 306.62 | 0.14 | NonCoding | -5 | 0.981 |
| candidate_1304 | - | A | 116 | 285.05 | 0 | AntiSense | -64.4 | 0.577 |
| candidate_1305 | - | A | 54 | 154.44 | 0 | AntiSense | -34.34 | 0.963 |
| candidate_1306 | - | A | 162 | 563.93 | 0.25 | NonCoding | -11.5 | 0.761 |
| candidate_1307 | - | A | 39 | 193.73 | 0 | AntiSense | -54.3 | 0.958 |
| candidate_1308 | - | A | 30 | 159.58 | 0 | AntiSense | -6.9 | 0.056 |
| candidate_1309 | - | A | 87 | 123.39 | 0 | AntiSense | -9 | 0.095 |
| candidate_1310 | - | A | 251 | 421.83 | 0 | 5'/3'-UTR | -38.8 | 0.673 |
| candidate_1311 | - | A | 97 | 126.64 | 0 | AntiSense | -100.4 | 0.714 |
| candidate_1312 | - | A | 65 | 350.32 | 0.14 | NonCoding | -34.4 | 0.619 |
| candidate_1313 | - | A | 39 | 1094.8 | 0.31 | NonCoding | -24 | 0.999 |
| candidate_1314 | - | A | 47 | 1070.38 | 0.5 | NonCoding | -0.27 | 0.209 |
| candidate_1315 | - | A | 72 | 2470.6 | 0.08 | 5'/3'-UTR | -17.9 | 0.803 |
| candidate_1316 | - | A | 36 | 555.38 | 0 | AntiSense | -10.9 | 0.286 |
| candidate_1317 | - | A | 53 | 364.41 | 0 | AntiSense | -13.2 | 0.48 |
| candidate_1318 | - | A | 45 | 119.78 | 0 | AntiSense | -17.1 | 0.029 |
| candidate_1319 | - | A | 30 | 83.58 | 0.14 | NonCoding | -16.64 | 0.485 |
| candidate_1320 | - | A | 32 | 626.97 | 0.14 | NonCoding | -2.2 | 0.181 |
| candidate_1321 | - | A | 34 | 82.23 | 0 | AntiSense | -12.9 | 0.02 |
| candidate_1322 | - | A | 76 | 846.65 | 0 | AntiSense | -14.5 | 0.942 |
| candidate_1323 | - | A | 32 | 76.42 | 0 | AntiSense | -27 | 0.621 |
| candidate_1324 | - | A | 77 | 509.36 | 0.01 | 5'/3'-UTR | -6.6 | 0.061 |
| candidate_1325 | - | A | 46 | 82.13 | 0 | AntiSense | -24.8 | 0.921 |
| candidate_1326 | - | A | 34 | 201.8 | 0.13 | NonCoding | -10.4 | 0.354 |
| candidate_1327 | - | A | 32 | 152.88 | 0 | AntiSense | -5.9 | 0.918 |
| candidate_1328 | - | A | 56 | 102.65 | 0 | AntiSense | -3.3 | 0.906 |
| candidate_1329 | - | A | 42 | 295.28 | 0.11 | NonCoding | -11.3 | 0.243 |
| candidate_1330 | - | A | 66 | 185.34 | 0.11 | NonCoding | -9.7 | 0.688 |
| candidate_1331 | - | A | 34 | 103.74 | 0.06 | NonCoding | -15.3 | 0.984 |
| candidate_1332 | - | A | 31 | 144.59 | 0 | AntiSense | -0.1 | 0.911 |
| candidate_1333 | - | A | 45 | 388.39 | 0.14 | NonCoding | -3 | 0.899 |
| candidate_1334 | - | A | 34 | 64.77 | 0.14 | NonCoding | -7.9 | 0.64 |
| candidate_1335 | - | A | 38 | 137.95 | 0 | AntiSense | -3.2 | 0.592 |
| candidate_1336 | - | A | 102 | 185.46 | 0 | AntiSense | -10.2 | 0.286 |
| candidate_1337 | - | A | 168 | 239.49 | 0 | AntiSense | -37.4 | 0.153 |
| candidate_1338 | - | A | 52 | 169.43 | 0 | AntiSense | -61 | 0.884 |
| candidate_1339 | - | A | 35 | 118.72 | 0 | AntiSense | -11.9 | 0.53 |
| candidate_1340 | - | A | 80 | 171.3 | 0.06 | 5'/3'-UTR | -4.3 | 0.976 |
| candidate_1341 | - | A | 64 | 395.25 | 0.12 | NonCoding | -19.6 | 0.001 |
| candidate_1342 | - | A | 30 | 307.32 | 0 | AntiSense | -31.8 | 0.261 |
| candidate_1343 | - | A | 35 | 107.56 | 0 | AntiSense | -6.5 | 0.624 |
| candidate_1344 | - | A | 80 | 361.28 | 0.25 | NonCoding | -7.6 | 0.583 |
| candidate_1345 | - | A | 30 | 324.13 | 0 | AntiSense | -35.2 | 0.45 |
| candidate_1346 | - | A | 41 | 171.5 | 0 | AntiSense | -3.3 | 0.081 |
| candidate_1347 | - | A | 30 | 237.42 | 0 | AntiSense | -12 | 0.556 |
| candidate_1348 | - | A | 136 | 342.89 | 0 | AntiSense | -5.2 | 0.651 |
| candidate_1349 | - | A | 168 | 346.46 | 0.06 | 5'/3'-UTR | -51.4 | 0.273 |
| candidate_1350 | - | A | 136 | 394.47 | 0.13 | NonCoding | -70.7 | 0.329 |
| candidate_1351 | - | A | 36 | 87.22 | 0 | AntiSense | -48.9 | 0.327 |
| candidate_1352 | - | A | 35 | 127.97 | 0 | AntiSense | -13.7 | 0.83 |
| candidate_1353 | - | A | 216 | 483.17 | 0 | AntiSense | -5 | 0.659 |
| candidate_1354 | - | A | 178 | 221.32 | 0 | AntiSense | -64 | 0.313 |
| candidate_1355 | - | A | 57 | 179.62 | 0 | 5'/3'-UTR | -73.6 | 0.564 |
| candidate_1356 | - | A | 33 | 413.06 | 0.02 | 5'/3'-UTR | -12.6 | 0.103 |
| candidate_1357 | - | A | 44 | 599.58 | 0.08 | NonCoding | -12.7 | 0.779 |
| candidate_1358 | - | A | 108 | 409.39 | 3.21 | NonCoding | -13.1 | 0.087 |
| candidate_1359 | - | A | 31 | 292.44 | 3.85 | NonCoding | -32.2 | 0.274 |
| candidate_1360 | - | A | 45 | 231.91 | 1.73 | NonCoding | -8.9 | 0.35 |
| candidate_1361 | - | A | 35 | 286.53 | 3.69 | NonCoding | -11.5 | 0.245 |
| candidate_1362 | - | A | 36 | 125.62 | 0 | AntiSense | -8.3 | 0.145 |
| candidate_1363 | - | A | 34 | 201.09 | 0 | AntiSense | -11.5 | 0.641 |
| candidate_1364 | - | A | 32 | 147 | 0 | AntiSense | -7.2 | 0.904 |
| candidate_1365 | - | A | 51 | 106.56 | 1.28 | NonCoding | -5.4 | 0.238 |
| candidate_1366 | - | A | 43 | 527.02 | 0.14 | NonCoding | -16.53 | 0.49 |
| candidate_1367 | - | A | 32 | 110.76 | 0 | AntiSense | -16.2 | 0.641 |
| candidate_1368 | - | A | 95 | 113.6 | 1.35 | NonCoding | -7.9 | 0.112 |
| candidate_1369 | - | A | 35 | 139.58 | 0.22 | 5'/3'-UTR | -20.5 | 0.541 |
| candidate_1370 | - | A | 39 | 124.23 | 0 | AntiSense | -4.3 | 0.738 |
| candidate_1371 | - | A | 148 | 207.8 | 0 | AntiSense | -2.5 | 0.692 |
| candidate_1372 | - | A | 33 | 69.62 | 0 | AntiSense | -50.4 | 0.759 |
| candidate_1373 | - | A | 57 | 192.16 | 0 | AntiSense | -7.4 | 0.484 |
| candidate_1374 | - | A | 56 | 98.86 | 0 | AntiSense | -12.94 | 0.453 |
| candidate_1375 | - | A | 279 | 198.13 | 0 | AntiSense | -14.2 | 0.256 |
| candidate_1376 | - | A | 31 | 101.28 | 0 | AntiSense | -119 | 0.856 |
| candidate_1377 | - | A | 216 | 413.89 | 0 | AntiSense | -3.6 | 0.983 |
| candidate_1378 | - | A | 38 | 131.95 | 0 | AntiSense | -67.3 | 0.777 |
| candidate_1379 | - | A | 62 | 226.11 | 0.64 | NonCoding | -9.8 | 0.182 |
| candidate_1380 | - | A | 35 | 1827.5 | 1.18 | NonCoding | -21.5 | 0.022 |
| candidate_1381 | - | A | 33 | 190.79 | 0 | AntiSense | -17.8 | 0.125 |
| candidate_1382 | - | A | 56 | 178.56 | 0 | AntiSense | -9.5 | 0.247 |
| candidate_1383 | - | A | 45 | 71.78 | 0 | AntiSense | -18.3 | 0.285 |
| candidate_1384 | - | A | 97 | 130.95 | 0 | 5'/3'-UTR | -9.1 | 0.916 |
| candidate_1385 | - | A | 37 | 128.05 | 0.14 | NonCoding | -19.1 | 0.758 |
| candidate_1386 | - | A | 51 | 233.13 | 0 | AntiSense | -5.3 | 0.864 |
| candidate_1387 | - | A | 81 | 337.52 | 0.14 | NonCoding | -12.7 | 0.06 |
| candidate_1388 | - | A | 66 | 122.37 | 0 | AntiSense | -32.3 | 0.559 |
| candidate_1389 | - | A | 84 | 149.11 | 0 | AntiSense | -20 | 0.522 |
| candidate_1390 | - | A | 44 | 217.78 | 0.05 | 5'/3'-UTR | -24.8 | 0.408 |
| candidate_1391 | - | A | 31 | 146 | 0 | AntiSense | -10.4 | 0.661 |
| candidate_1392 | - | A | 222 | 442.52 | 0 | AntiSense | -4.4 | 0.175 |
| candidate_1393 | - | A | 30 | 1754.58 | 2.82 | NonCoding | -109.6 | 0.146 |
| candidate_1394 | - | A | 61 | 230.92 | 0 | AntiSense | -5.8 | 0.751 |
| candidate_1395 | - | A | 56 | 100.84 | 0.09 | NonCoding | -13.4 | 0.573 |
| candidate_1396 | - | A | 43 | 88.52 | 0 | AntiSense | -14.6 | 0.129 |
| candidate_1397 | - | A | 286 | 684.42 | 0.24 | 5'/3'-UTR | -15.1 | 0.04 |
| candidate_1398 | - | A | 32 | 181.09 | 0 | AntiSense | -130.02 | 0.245 |
| candidate_1399 | - | A | 35 | 207.36 | 0 | AntiSense | -7.6 | 0.364 |
| candidate_1400 | - | A | 57 | 85.81 | 1.87 | NonCoding | -10.4 | 0.241 |
| candidate_1401 | - | A | 243 | 447.73 | 0 | 5'/3'-UTR | -12.5 | 0.888 |
| candidate_1402 | - | A | 37 | 185.68 | 0 | AntiSense | -84.7 | 0.289 |
| candidate_1403 | - | A | 40 | 182.49 | 0 | AntiSense | -10 | 0.977 |
| candidate_1404 | - | A | 68 | 723.67 | 0 | 5'/3'-UTR | -4.4 | 0.912 |
| candidate_1405 | - | A | 241 | 903.29 | 0 | AntiSense | -11.8 | 0.985 |
| candidate_1406 | - | A | 51 | 232.83 | 0.13 | NonCoding | -78.11 | 0.702 |
| candidate_1407 | - | A | 66 | 133.4 | 0 | AntiSense | -10.2 | 0.938 |
| candidate_1408 | - | A | 37 | 116.34 | 0.1 | NonCoding | -18.4 | 0.098 |
| candidate_1409 | - | A | 32 | 333.39 | 0.04 | 5'/3'-UTR | -8.4 | 0.1 |
| candidate_1410 | - | A | 61 | 237.76 | 0.12 | NonCoding | -8.8 | 0.207 |
| candidate_1411 | - | A | 174 | 444.45 | 0.07 | 5'/3'-UTR | -23.5 | 0.001 |
| candidate_1412 | - | A | 37 | 275.42 | 0.14 | NonCoding | -94.6 | 0.977 |
| candidate_1413 | - | A | 38 | 820.79 | 0.07 | NonCoding | -5.3 | 0.208 |
| candidate_1414 | - | A | 45 | 132.17 | 0.64 | NonCoding | -10.2 | 0.03 |
| candidate_1415 | - | A | 54 | 579.98 | 0 | AntiSense | -19.8 | 0.139 |
| candidate_1416 | - | A | 102 | 216.3 | 0.06 | 5'/3'-UTR | -15.4 | 0.857 |
| candidate_1417 | - | A | 44 | 282.93 | 0 | AntiSense | -28.7 | 0.914 |
| candidate_1418 | - | A | 41 | 101.31 | 0.13 | NonCoding | -10.4 | 0.428 |
| candidate_1419 | - | A | 37 | 442.21 | 0.11 | NonCoding | -3.1 | 0.66 |
| candidate_1420 | - | A | 30 | 215.68 | 0 | AntiSense | -2.4 | 0.371 |
| candidate_1421 | - | A | 37 | 264.84 | 0 | AntiSense | -6.2 | 0.657 |
| candidate_1422 | - | A | 38 | 91.67 | 0.1 | 5'/3'-UTR | -10.1 | 0.986 |
| candidate_1423 | - | A | 63 | 209.25 | 0 | 5'/3'-UTR | -3.9 | 0.159 |
| candidate_1424 | - | A | 35 | 161.75 | 0 | AntiSense | -25.6 | 0.337 |
| candidate_1425 | - | A | 31 | 110.94 | 0.03 | 5'/3'-UTR | -9.43 | 0.588 |
| candidate_1426 | - | A | 43 | 192.68 | 0 | AntiSense | -7.3 | 0.41 |
| candidate_1427 | - | A | 47 | 24201.42 | 0.1 | NonCoding | -9 | 0.667 |
| candidate_1428 | - | A | 83 | 595.07 | 0.13 | NonCoding | -4.89 | 0.281 |
| candidate_1429 | - | A | 31 | 80.25 | 0 | AntiSense | -21.5 | 0.601 |
| candidate_1430 | - | A | 35 | 620.28 | 0.46 | NonCoding | -4.6 | 0.001 |
| candidate_1431 | - | A | 152 | 189.93 | 2.02 | NonCoding | -25.1 | 0.609 |
| candidate_1432 | - | A | 42 | 823.3 | 2.28 | NonCoding | -50.3 | 0.477 |
| candidate_1433 | - | A | 133 | 686.03 | 0.58 | 5'/3'-UTR | -12.4 | 0.277 |
| candidate_1434 | - | A | 91 | 917.61 | 0.1 | NonCoding | -44.2 | 0.799 |
| candidate_1435 | - | A | 37 | 161.55 | 0.92 | NonCoding | -24.7 | 0.817 |
| candidate_1436 | - | A | 37 | 168.13 | 0 | 5'/3'-UTR | -4 | 0.16 |
| candidate_1437 | - | A | 35 | 126.42 | 0 | AntiSense | -2.8 | 0.158 |
| candidate_1438 | - | A | 160 | 587.54 | 0 | AntiSense | -8.6 | 0.543 |
| candidate_1439 | - | A | 31 | 266.66 | 0.13 | NonCoding | -56.4 | 0.989 |
| candidate_1440 | - | A | 30 | 147.55 | 0.14 | NonCoding | -2.1 | 0.991 |
| candidate_1441 | - | A | 36 | 560.22 | 0.16 | NonCoding | -0.8 | 0.821 |
| candidate_1442 | - | A | 40 | 168.59 | 0 | AntiSense | -1.9 | 0.969 |
| candidate_1443 | - | A | 41 | 190.64 | 0 | AntiSense | -7 | 0.22 |
| candidate_1444 | - | A | 34 | 178.94 | 1.54 | NonCoding | -12.3 | 0.042 |
| candidate_1445 | - | A | 39 | 180.68 | 0.14 | NonCoding | -12 | 0.903 |
| candidate_1446 | - | A | 36 | 256.08 | 0 | AntiSense | -2.9 | 0.742 |
| candidate_1447 | - | A | 310 | 1162.86 | 0 | 5'/3'-UTR | -7.6 | 0.267 |
| candidate_1448 | - | A | 59 | 345.45 | 0.13 | NonCoding | -130.62 | 0.398 |
| candidate_1449 | - | A | 37 | 718.11 | 0.13 | NonCoding | -15.9 | 0.572 |
| candidate_1450 | - | A | 94 | 432.62 | 0.14 | NonCoding | -6 | 0.041 |
| candidate_1451 | - | A | 30 | 77.45 | 0 | AntiSense | -33.8 | 0.92 |
| candidate_1452 | - | A | 32 | 313.3 | 0.12 | NonCoding | -3.7 | 0.068 |
| candidate_1453 | - | A | 35 | 136.14 | 0.14 | NonCoding | -7.6 | 0.735 |
| candidate_1454 | - | A | 33 | 287.21 | 0 | AntiSense | -3.03 | 0.039 |
| candidate_1455 | - | A | 31 | 119.66 | 0.07 | NonCoding | -10.2 | 0.243 |
| candidate_1456 | - | A | 235 | 260.5 | 0 | 5'/3'-UTR | -8.9 | 0.068 |
| candidate_1457 | - | A | 173 | 185.71 | 0 | AntiSense | -102.3 | 0.19 |
| candidate_1458 | - | A | 156 | 586.03 | 0 | AntiSense | -73.8 | 0.883 |
| candidate_1459 | - | A | 107 | 733.62 | 0 | AntiSense | -52.2 | 0.945 |
| candidate_1460 | - | A | 161 | 463.51 | 0 | AntiSense | -27.1 | 0.009 |
| candidate_1461 | - | A | 49 | 173.86 | 0 | AntiSense | -89.03 | 0.474 |
| candidate_1462 | - | A | 80 | 296.85 | 0 | AntiSense | -13.7 | 0.469 |
| candidate_1463 | - | A | 38 | 103.31 | 0 | AntiSense | -34.1 | 0.26 |
| candidate_1464 | - | A | 33 | 161.21 | 0.46 | NonCoding | -10.6 | 0.639 |
| candidate_1465 | - | A | 53 | 92.15 | 0.24 | NonCoding | -4.1 | 0.142 |
| candidate_1466 | - | A | 41 | 140.02 | 0.22 | NonCoding | -21.2 | 0.399 |
| candidate_1467 | - | A | 38 | 1930.21 | 0.14 | NonCoding | -18.7 | 0.001 |
| candidate_1468 | - | A | 37 | 138.29 | 0.1 | 5'/3'-UTR | -26 | 0.953 |
| candidate_1469 | - | A | 52 | 150.58 | 0 | AntiSense | -1.22 | 0.906 |
| candidate_1470 | - | A | 72 | 388.52 | 0.14 | NonCoding | -14.9 | 0.513 |
| candidate_1471 | - | A | 131 | 217.46 | 0 | AntiSense | -22.7 | 0.445 |
| candidate_1472 | - | A | 80 | 133.98 | 0 | AntiSense | -37.92 | 0.934 |
| candidate_1473 | - | A | 49 | 221.92 | 0 | AntiSense | -23.2 | 0.793 |
| candidate_1474 | - | A | 39 | 137.35 | 0 | AntiSense | -9.9 | 0.739 |
| candidate_1475 | - | A | 135 | 722.3 | 0 | AntiSense | -6.6 | 0.818 |
| candidate_1476 | - | A | 62 | 170.08 | 0 | 5'/3'-UTR | -40.6 | 0.763 |
| candidate_1477 | - | A | 54 | 277.13 | 0 | AntiSense | -10.01 | 0.818 |
| candidate_1478 | - | A | 34 | 161.63 | 0 | AntiSense | -10.6 | 0.972 |
| candidate_1479 | - | A | 33 | 151.41 | 0 | AntiSense | -1.2 | 0.731 |
| candidate_1480 | - | A | 30 | 123.23 | 0 | AntiSense | -7.2 | 0.249 |
| candidate_1481 | - | A | 151 | 99.09 | 0 | AntiSense | -6.1 | 0.349 |
| candidate_1482 | - | A | 53 | 114.28 | 0.14 | NonCoding | -52.5 | 0.541 |
| candidate_1483 | - | A | 161 | 245.38 | 0.36 | NonCoding | -16.3 | 0.522 |
| candidate_1484 | - | A | 45 | 638.87 | 0 | AntiSense | -60.6 | 0.488 |
| candidate_1485 | - | A | 35 | 129.78 | 0 | AntiSense | -12.3 | 0.914 |
| candidate_1486 | - | A | 39 | 200.95 | 0 | AntiSense | -2.7 | 0.064 |
| candidate_1487 | - | A | 39 | 149.23 | 0 | AntiSense | -19.6 | 0.429 |
| candidate_1488 | - | A | 39 | 76.38 | 0 | AntiSense | -10.4 | 0.263 |
| candidate_1489 | - | A | 41 | 152.69 | 0.1 | NonCoding | -14.4 | 0.911 |
| candidate_1490 | - | A | 150 | 137.07 | 0 | AntiSense | -1.9 | 0.804 |
| candidate_1491 | - | A | 33 | 172.12 | 0.14 | NonCoding | -42.5 | 0.664 |
| candidate_1492 | - | A | 30 | 179.26 | 0 | AntiSense | -2.4 | 0.671 |
| candidate_1493 | - | A | 79 | 280.63 | 0 | AntiSense | -3.6 | 0.013 |
| candidate_1494 | - | A | 103 | 310.52 | 0.14 | NonCoding | -30.11 | 0.786 |
| candidate_1495 | - | A | 152 | 918.11 | 0 | 5'/3'-UTR | -37.9 | 0.879 |
| candidate_1496 | - | A | 30 | 263.23 | 0.47 | NonCoding | -47.1 | 0.979 |
| candidate_1497 | - | A | 63 | 335.7 | 0.12 | 5'/3'-UTR | -3.5 | 0.001 |
| candidate_1498 | - | A | 46 | 770.34 | 0.88 | NonCoding | -38.2 | 0.41 |
| candidate_1499 | - | A | 40 | 244.54 | 0 | AntiSense | -7.4 | 0.33 |
| candidate_1500 | - | A | 50 | 119.1 | 0.05 | 5'/3'-UTR | -7.4 | 0.996 |
| candidate_1501 | - | A | 189 | 141.84 | 0.1 | NonCoding | -3.8 | 0.091 |
| candidate_1502 | - | A | 130 | 214.75 | 2.04 | NonCoding | -81.3 | 0.001 |
| candidate_1503 | - | A | 50 | 916.86 | 0 | AntiSense | -63.6 | 0.975 |
| candidate_1504 | - | A | 31 | 156.41 | 2.61 | NonCoding | -8.4 | 0.54 |
| candidate_1505 | - | A | 43 | 515.32 | 0.26 | NonCoding | -7.1 | 0.81 |
| candidate_1506 | - | A | 42 | 89.65 | 0.1 | NonCoding | -9.1 | 0.372 |
| candidate_1507 | - | A | 346 | 1504.99 | 1.22 | NonCoding | -10.1 | 0.594 |
| candidate_1508 | - | A | 33 | 68.26 | 0 | AntiSense | -122.2 | 0.466 |
| candidate_1509 | - | A | 32 | 127.91 | 0 | AntiSense | -2.4 | 0.457 |
| candidate_1510 | - | A | 35 | 1455.61 | 0 | 5'/3'-UTR | -6.4 | 0.971 |
| candidate_1511 | - | A | 43 | 1359.07 | 0 | AntiSense | -4.2 | 0.203 |
| candidate_1512 | - | A | 43 | 399.41 | 0.04 | 5'/3'-UTR | -10.8 | 0.76 |
| candidate_1513 | - | A | 56 | 104.88 | 0 | AntiSense | -6.2 | 0.093 |
| candidate_1514 | - | A | 170 | 610.01 | 0 | AntiSense | -20 | 0.851 |
| candidate_1515 | - | A | 72 | 143.82 | 0.14 | NonCoding | -61.5 | 0.62 |
| candidate_1516 | - | A | 42 | 322.72 | 0.13 | NonCoding | -15.5 | 0.115 |
| candidate_1517 | - | A | 148 | 609.52 | 0 | AntiSense | -13.5 | 0.047 |
| candidate_1518 | - | A | 32 | 116.03 | 0.14 | NonCoding | -75.31 | 0.747 |
| candidate_1519 | - | A | 39 | 758.63 | 0.09 | NonCoding | -5.6 | 0.503 |
| candidate_1520 | - | A | 160 | 226.66 | 0.05 | 5'/3'-UTR | -13.5 | 0.109 |
| candidate_1521 | - | A | 30 | 387.68 | 0 | AntiSense | -61.5 | 0.675 |
| candidate_1522 | - | A | 39 | 114.93 | 0.08 | NonCoding | -3 | 0.438 |
| candidate_1523 | - | A | 116 | 114.21 | 0.15 | NonCoding | -7.8 | 0.725 |
| candidate_1524 | - | A | 74 | 385 | 0.12 | NonCoding | -43.2 | 0.174 |
| candidate_1525 | - | A | 103 | 147.17 | 0 | AntiSense | -26 | 0.553 |
| candidate_1526 | - | A | 39 | 68.3 | 0.07 | 5'/3'-UTR | -35.3 | 0.393 |
| candidate_1527 | - | A | 61 | 180.48 | 0 | AntiSense | -9.1 | 0.367 |
| candidate_1528 | - | A | 34 | 174.71 | 0.93 | NonCoding | -21.3 | 0.951 |
| candidate_1529 | - | A | 42 | 137.21 | 0 | AntiSense | -9 | 0.331 |
| candidate_1530 | - | A | 36 | 265.73 | 0 | AntiSense | -12.4 | 0.114 |
| candidate_1531 | - | A | 150 | 306.53 | 0.09 | NonCoding | -11.3 | 0.649 |
| candidate_1532 | - | A | 225 | 341.81 | 0.16 | NonCoding | -47.2 | 1 |
| candidate_1533 | - | A | 35 | 272.47 | 0.26 | NonCoding | -74.3 | 0.001 |
| candidate_1534 | - | A | 33 | 96.41 | 0.1 | NonCoding | -26.5 | 0.001 |
| candidate_1535 | - | A | 49 | 484.76 | 0.14 | NonCoding | -18.1 | 0.668 |
| candidate_1536 | - | A | 32 | 69.42 | 0.35 | NonCoding | -10 | 0.931 |
| candidate_1537 | - | A | 34 | 144.46 | 1.34 | NonCoding | -3.5 | 0.001 |
| candidate_1538 | - | A | 141 | 274.38 | 2.02 | NonCoding | -20.2 | 0.87 |
| candidate_1539 | - | A | 214 | 1338.87 | 1.08 | NonCoding | -26 | 0.007 |
| candidate_1540 | - | A | 64 | 371.26 | 0 | AntiSense | -95.79 | 0.773 |
| candidate_1541 | - | A | 115 | 268.24 | 0 | AntiSense | -13.92 | 0.72 |
| candidate_1542 | - | A | 201 | 772.71 | 1.57 | NonCoding | -39.1 | 0.906 |
| candidate_1543 | - | A | 67 | 193.06 | 3.43 | NonCoding | -66.8 | 0.048 |
| candidate_1544 | - | A | 37 | 85.13 | 0 | AntiSense | -18.5 | 0.079 |
| candidate_1545 | - | A | 90 | 117.76 | 0.05 | 5'/3'-UTR | -15.1 | 0.226 |
| candidate_1546 | - | A | 103 | 187.17 | 0 | AntiSense | -23.8 | 0.652 |
| candidate_1547 | - | A | 36 | 420.54 | 0 | AntiSense | -38 | 0.743 |
| candidate_1548 | - | A | 84 | 350.07 | 0 | AntiSense | -6.5 | 0.771 |
| candidate_1549 | - | A | 34 | 198.77 | 0 | AntiSense | -25.3 | 0.86 |
| candidate_1550 | - | A | 92 | 457.56 | 0 | AntiSense | -4.34 | 0.252 |
| candidate_1551 | - | A | 39 | 110.43 | 0 | AntiSense | -29.7 | 0.299 |
| candidate_1552 | - | A | 40 | 155.73 | 0 | AntiSense | -11.7 | 0.28 |
| candidate_1553 | - | A | 55 | 209.21 | 0 | AntiSense | -8.9 | 0.538 |
| candidate_1554 | - | A | 31 | 119.75 | 0 | AntiSense | -12.4 | 0.012 |
| candidate_1555 | - | A | 86 | 188.51 | 0 | AntiSense | -15.9 | 0.069 |
| candidate_1556 | - | A | 86 | 812.53 | 0.1 | NonCoding | -37.4 | 0.001 |
| candidate_1557 | - | A | 64 | 153.34 | 0 | AntiSense | -34.5 | 0.627 |
| candidate_1558 | - | A | 38 | 239.28 | 0 | AntiSense | -26.6 | 0.298 |
| candidate_1559 | - | A | 117 | 202.11 | 0 | AntiSense | -9 | 0.286 |
| candidate_1560 | - | A | 36 | 160.14 | 0 | AntiSense | -39.5 | 0.712 |
| candidate_1561 | - | A | 39 | 126.28 | 0.14 | NonCoding | -5.7 | 0.316 |
| candidate_1562 | - | A | 35 | 90.08 | 0 | AntiSense | -11.5 | 0.327 |
| candidate_1563 | - | A | 258 | 236.97 | 0 | AntiSense | -7.84 | 0.97 |
| candidate_1564 | - | A | 34 | 126.26 | 0 | AntiSense | -90.1 | 0.825 |
| candidate_1565 | - | A | 170 | 173.51 | 3.09 | NonCoding | -8.1 | 0.241 |
| candidate_1566 | - | A | 210 | 246.2 | 0 | AntiSense | -76.7 | 0.722 |
| candidate_1567 | - | A | 95 | 570.26 | 0 | AntiSense | -69.4 | 0.523 |
| candidate_1568 | - | A | 32 | 2256.39 | 0.13 | NonCoding | -25.9 | 0.339 |
| candidate_1569 | - | A | 33 | 541.5 | 0.65 | NonCoding | -5.8 | 0.963 |
| candidate_1570 | - | A | 82 | 372.96 | 0.58 | NonCoding | -1.4 | 0.396 |
| candidate_1571 | - | A | 51 | 123.19 | 0 | 5'/3'-UTR | -25.7 | 0.714 |
| candidate_1572 | - | A | 32 | 75.97 | 0 | AntiSense | -10.6 | 0.518 |
| candidate_1573 | - | A | 40 | 300.51 | 0.1 | NonCoding | -9.3 | 0.241 |
| candidate_1574 | - | A | 128 | 597.53 | 0.05 | 5'/3'-UTR | -10.5 | 0.993 |
| candidate_1575 | - | A | 43 | 136.05 | 0 | AntiSense | -36.1 | 0.576 |
| candidate_1576 | - | A | 70 | 1169.83 | 0.63 | NonCoding | -11.4 | 0.64 |
| candidate_1577 | - | A | 45 | 79.87 | 0 | AntiSense | -17.3 | 0.649 |
| candidate_1578 | - | A | 53 | 167.2 | 0 | AntiSense | -14.3 | 0.961 |
| candidate_1579 | - | A | 33 | 81.44 | 0 | AntiSense | -11.2 | 0.726 |
| candidate_1580 | - | A | 30 | 257.61 | 0 | AntiSense | -7.1 | 0.657 |
| candidate_1581 | - | A | 50 | 104.73 | 0.1 | NonCoding | -1.5 | 0.707 |
| candidate_1582 | - | A | 31 | 89.16 | 0.14 | NonCoding | -7.91 | 0.708 |
| candidate_1583 | - | A | 38 | 193.21 | 0.14 | NonCoding | -6.7 | 0.846 |
| candidate_1584 | - | A | 35 | 176.69 | 0 | AntiSense | -7.7 | 0.426 |
| candidate_1585 | - | A | 38 | 132.36 | 0 | AntiSense | -9.5 | 0.119 |
| candidate_1586 | - | A | 58 | 142.2 | 0 | AntiSense | -14.1 | 0.416 |
| candidate_1587 | - | A | 212 | 189.65 | 0 | AntiSense | -8.3 | 0.438 |
| candidate_1588 | - | A | 43 | 261.57 | 1.14 | 5'/3'-UTR | -88.8 | 0.295 |
| candidate_1589 | - | A | 43 | 198.18 | 0 | AntiSense | -14.5 | 0.85 |
| candidate_1590 | - | A | 31 | 153.16 | 1.73 | NonCoding | -8.6 | 0.015 |
| candidate_1591 | - | A | 51 | 247 | 0 | AntiSense | -10.6 | 0.262 |
| candidate_1592 | - | A | 73 | 849.07 | 0.04 | 5'/3'-UTR | -10.8 | 0.973 |
| candidate_1593 | - | A | 31 | 141.59 | 0 | 5'/3'-UTR | -23.1 | 0.814 |
| candidate_1594 | - | A | 46 | 235.17 | 1.9 | NonCoding | -5.8 | 0.938 |
| candidate_1595 | - | A | 40 | 499.54 | 1.66 | NonCoding | -7.7 | 0.849 |
| candidate_1596 | - | A | 70 | 233.76 | 0.14 | NonCoding | -11.8 | 0.313 |
| candidate_1597 | - | A | 148 | 348.06 | 0.01 | 5'/3'-UTR | -20.2 | 0.69 |
| candidate_1598 | - | A | 167 | 349.07 | 0.1 | 5'/3'-UTR | -53 | 0.01 |
| candidate_1599 | - | A | 35 | 477.06 | 0.11 | NonCoding | -72 | 0.983 |
| candidate_1600 | - | A | 203 | 612.16 | 0 | AntiSense | -2.7 | 0.042 |
| candidate_1601 | - | A | 63 | 461.03 | 6.04 | NonCoding | -85.4 | 0.01 |
| candidate_1602 | - | A | 30 | 358.55 | 0 | AntiSense | -28.9 | 0.981 |
| candidate_1603 | - | A | 93 | 2468.26 | 4.13 | NonCoding | -2.5 | 0.001 |
| candidate_1604 | - | A | 73 | 215.82 | 1.85 | NonCoding | -55.8 | 0.211 |
| candidate_1605 | - | A | 39 | 207.2 | 0.74 | NonCoding | -32.4 | 0.981 |
| candidate_1606 | - | A | 95 | 373.44 | 0 | AntiSense | -5.4 | 0.116 |
| candidate_1607 | - | A | 31 | 232.69 | 0 | AntiSense | -38.8 | 0.975 |
| candidate_1608 | - | A | 38 | 307.23 | 0 | AntiSense | -3.2 | 0.447 |
| candidate_1609 | - | A | 43 | 118.86 | 0 | AntiSense | -10.9 | 0.326 |
| candidate_1610 | - | A | 42 | 161.91 | 0 | AntiSense | -14.3 | 0.02 |
| candidate_1611 | - | A | 224 | 789.31 | 0 | AntiSense | -21.3 | 0.524 |
| candidate_1612 | - | A | 38 | 82.64 | 1.78 | NonCoding | -84.1 | 0.876 |
| candidate_1613 | - | A | 33 | 112.03 | 0 | AntiSense | -4.3 | 0.874 |
| candidate_1614 | - | A | 52 | 610.23 | 0 | AntiSense | -8.5 | 0.771 |
| candidate_1615 | - | A | 32 | 227.94 | 0 | AntiSense | -7.4 | 0.718 |
| candidate_1616 | - | A | 36 | 126.35 | 0 | AntiSense | -7.2 | 0.508 |
| candidate_1617 | - | A | 41 | 530.38 | 2.17 | NonCoding | -7.2 | 0.154 |
| candidate_1618 | - | A | 47 | 244.33 | 0 | AntiSense | -6.9 | 0.992 |
| candidate_1619 | - | A | 54 | 422.29 | 0 | AntiSense | -2.8 | 0.467 |
| candidate_1620 | - | A | 62 | 143.83 | 0 | AntiSense | -16.5 | 0.778 |
| candidate_1621 | - | A | 88 | 957.27 | 3.68 | NonCoding | -13.2 | 0.042 |
| candidate_1622 | - | A | 69 | 148.34 | 0 | AntiSense | -45.4 | 0.544 |
| candidate_1623 | - | A | 264 | 428.49 | 0.08 | 5'/3'-UTR | -19.5 | 0.14 |
| candidate_1624 | - | A | 36 | 196.95 | 0 | AntiSense | -120.3 | 0.926 |
| candidate_1625 | - | A | 43 | 214.82 | 0 | AntiSense | -2.8 | 0.19 |
| candidate_1626 | - | A | 38 | 83.95 | 0 | AntiSense | -13.6 | 0.58 |
| candidate_1627 | - | A | 146 | 79.37 | 0.18 | 5'/3'-UTR | -9.3 | 0.583 |
| candidate_1628 | - | A | 315 | 641.23 | 0 | AntiSense | -48.7 | 0.104 |
| candidate_1629 | - | A | 36 | 120.97 | 0.14 | NonCoding | -140.1 | 0.342 |
| candidate_1630 | - | A | 31 | 86.16 | 0 | AntiSense | -5.3 | 0.751 |
| candidate_1631 | - | A | 43 | 169.43 | 0.1 | NonCoding | -4.5 | 0.115 |
| candidate_1632 | - | A | 74 | 187.73 | 0 | AntiSense | -12.9 | 0.01 |
| candidate_1633 | - | A | 39 | 92.75 | 0 | AntiSense | -28.6 | 0.573 |
| candidate_1634 | - | A | 52 | 169.89 | 0 | AntiSense | -8.6 | 0.473 |
| candidate_1635 | - | A | 35 | 110.06 | 0.78 | NonCoding | -13.2 | 0.109 |
| candidate_1636 | - | A | 30 | 202.39 | 0.13 | NonCoding | -8.6 | 0.749 |
| candidate_1637 | - | A | 68 | 373.61 | 0.88 | NonCoding | -4 | 0.43 |
| candidate_1638 | - | A | 149 | 107.57 | 0 | AntiSense | -20.8 | 0.29 |
| candidate_1639 | - | A | 181 | 147.25 | 0 | AntiSense | -51.9 | 0.049 |
| candidate_1640 | - | A | 47 | 298.08 | 0.2 | NonCoding | -93.1 | 0.91 |
| candidate_1641 | - | A | 196 | 165.31 | 0 | AntiSense | -5 | 0.87 |
| candidate_1642 | - | A | 104 | 800.81 | 0 | AntiSense | -71.7 | 0.732 |
| candidate_1643 | - | A | 37 | 136.32 | 0 | AntiSense | -31 | 0.864 |
| candidate_1644 | - | A | 47 | 576.38 | 0.45 | NonCoding | -7.1 | 0.518 |
| candidate_1645 | - | A | 32 | 79.39 | 0 | AntiSense | -10.3 | 0.871 |
| candidate_1646 | - | A | 145 | 268.47 | 0 | AntiSense | -4.3 | 0.679 |
| candidate_1647 | - | A | 41 | 458.71 | 0.14 | NonCoding | -42.64 | 0.816 |
| candidate_1648 | - | A | 37 | 113.11 | 0 | AntiSense | -9.2 | 0.963 |
| candidate_1649 | - | A | 115 | 720.99 | 1.84 | NonCoding | -7.9 | 0.321 |
| candidate_1650 | - | A | 39 | 98.53 | 0.14 | NonCoding | -37 | 0.514 |
| candidate_1651 | - | A | 217 | 713.83 | 0.14 | 5'/3'-UTR | -6.5 | 0.051 |
| candidate_1652 | - | A | 35 | 216.03 | 0 | AntiSense | -72.8 | 0.692 |
| candidate_1653 | - | A | 33 | 137.74 | 0 | AntiSense | -7.3 | 0.658 |
| candidate_1654 | - | A | 47 | 202.15 | 0 | AntiSense | -6.6 | 0.027 |
| candidate_1655 | - | A | 46 | 417.91 | 0.21 | NonCoding | -17.5 | 0.008 |
| candidate_1656 | - | A | 32 | 192.39 | 0 | AntiSense | -20.9 | 0.135 |
| candidate_1657 | - | A | 90 | 250.95 | 0 | AntiSense | -11.2 | 0.309 |
| candidate_1658 | - | A | 30 | 100.97 | 0 | AntiSense | -30.7 | 0.801 |
| candidate_1659 | - | A | 96 | 86.84 | 0 | AntiSense | -4 | 0.435 |
| candidate_1660 | - | A | 32 | 153.12 | 0 | AntiSense | -28 | 0.257 |
| candidate_1661 | - | A | 40 | 139.2 | 0 | AntiSense | -8.1 | 0.172 |
| candidate_1662 | - | A | 37 | 77.55 | 0 | AntiSense | -10.1 | 0.11 |
| candidate_1663 | - | A | 65 | 426.05 | 0.12 | NonCoding | -13.3 | 0.895 |
| candidate_1664 | - | A | 33 | 479.62 | 0.13 | NonCoding | -10.8 | 0.066 |
| candidate_1665 | - | A | 32 | 112.12 | 0 | AntiSense | -8.9 | 0.712 |
| candidate_1666 | - | A | 124 | 251.33 | 0 | 5'/3'-UTR | -4.4 | 0.299 |
| candidate_1667 | - | A | 49 | 111.62 | 0 | AntiSense | -43.4 | 0.155 |
| candidate_1668 | - | A | 69 | 122.04 | 0.1 | NonCoding | -17.7 | 0.694 |
| candidate_1669 | - | A | 38 | 142.97 | 0.04 | 5'/3'-UTR | -17.8 | 0.32 |
| candidate_1670 | - | B | 30 | 64.77 | 0.95 | NonCoding | -7.6 | 0.838 |
| candidate_1671 | - | B | 41 | 107.95 | 1.31 | NonCoding | -4 | 0.881 |
| candidate_1672 | - | B | 36 | 87.78 | 0.95 | NonCoding | -8.9 | 0.632 |
| candidate_1673 | - | B | 33 | 135.65 | 0.96 | NonCoding | -10.4 | 0.3 |
| candidate_1674 | - | B | 45 | 118.48 | 0.96 | NonCoding | -11.6 | 0.806 |
| candidate_1675 | - | B | 31 | 141.38 | 1.93 | NonCoding | -7 | 0.775 |
| candidate_1676 | - | B | 56 | 388.51 | 1.97 | NonCoding | -3.3 | 0.18 |
| candidate_1677 | - | B | 39 | 90.23 | 0.95 | NonCoding | -21.4 | 0.133 |
| candidate_1678 | - | B | 32 | 45.91 | 4.75 | NonCoding | -15.7 | 0.411 |
| candidate_1679 | - | B | 40 | 48.1 | 0.95 | NonCoding | -3.1 | 0.768 |
| candidate_1680 | - | B | 50 | 48.06 | 2.1 | NonCoding | -5.5 | 0.004 |
| candidate_1681 | - | B | 39 | 150.95 | 5.37 | NonCoding | -30 | 0.407 |
| candidate_1682 | - | B | 75 | 65.61 | 2.34 | NonCoding | -11.3 | 0.092 |
| candidate_1683 | - | B | 38 | 46.15 | 1.5 | NonCoding | -21.7 | 0.612 |
| candidate_1684 | - | B | 37 | 34.82 | 2.77 | NonCoding | -7 | 0.001 |
| candidate_1685 | - | B | 33 | 42.79 | 1.98 | NonCoding | -28.6 | 0.002 |
| candidate_1686 | - | B | 38 | 104.54 | 1.52 | NonCoding | -22.1 | 0.001 |
| candidate_1687 | - | B | 61 | 265.32 | 1.36 | NonCoding | -21.6 | 0.429 |
| candidate_1688 | - | B | 44 | 61.42 | 1.98 | NonCoding | -15.1 | 0.002 |
| candidate_1689 | - | B | 64 | 188.03 | 2.69 | NonCoding | -24.9 | 0.011 |
| candidate_1690 | - | B | 34 | 45.06 | 2.42 | NonCoding | -29.2 | 0.12 |
| candidate_1691 | - | B | 38 | 100.26 | 2.92 | NonCoding | -11.1 | 0.512 |
| candidate_1692 | - | B | 44 | 996.27 | 1.77 | NonCoding | -12.9 | 0.199 |
| candidate_1693 | - | B | 37 | 330.16 | 0.96 | NonCoding | -11.1 | 0.957 |
| candidate_1694 | - | B | 31 | 28.81 | 0.96 | NonCoding | -7.4 | 0.648 |
| candidate_1695 | - | B | 78 | 46.65 | 2.11 | NonCoding | -9.1 | 0.446 |
| candidate_1696 | - | B | 31 | 831.16 | 2.8 | NonCoding | -22.8 | 0.102 |
| candidate_1697 | - | B | 33 | 112.35 | 1.31 | NonCoding | -9 | 0.918 |
| candidate_1698 | - | B | 31 | 56.13 | 3.08 | NonCoding | -5.5 | 0.11 |
| candidate_1699 | - | B | 30 | 39.48 | 4.83 | NonCoding | -8.6 | 0.541 |
| candidate_1700 | - | B | 37 | 45.03 | 1.62 | NonCoding | -7.2 | 0.001 |
| candidate_1701 | - | B | 33 | 37.82 | 2.17 | NonCoding | -34.7 | 0.187 |
| candidate_1702 | - | B | 32 | 65.52 | 0.95 | NonCoding | -7.1 | 0.78 |
| candidate_1703 | - | B | 30 | 4989.29 | 4.16 | NonCoding | -5 | 0.685 |
| candidate_1704 | - | B | 30 | 113.65 | 1.87 | NonCoding | -3.9 | 0.284 |
| candidate_1705 | - | B | 30 | 1927.52 | 4.92 | NonCoding | -8.3 | 0.508 |
| candidate_1706 | - | B | 35 | 55.28 | 1.55 | NonCoding | -10.3 | 0.887 |
| candidate_1707 | - | B | 30 | 118.94 | 0.95 | NonCoding | -3.4 | 0.773 |
| candidate_1708 | - | B | 34 | 52.91 | 2.61 | NonCoding | -3.8 | 0.516 |
| candidate_1709 | - | B | 31 | 1167.13 | 1.04 | NonCoding | -4.2 | 0.593 |
| candidate_1710 | - | B | 36 | 54.3 | 3.29 | NonCoding | -6.5 | 0.437 |
| candidate_1711 | - | B | 40 | 46.63 | 1.42 | NonCoding | -6.7 | 0.002 |
| candidate_1712 | - | B | 36 | 106.81 | 3.38 | NonCoding | -24.1 | 0.179 |
| candidate_1713 | - | B | 30 | 96.97 | 4.95 | NonCoding | -16.2 | 0.442 |
| candidate_1714 | - | B | 40 | 32.24 | 3.97 | NonCoding | -5.6 | 0.638 |
| candidate_1715 | - | B | 61 | 30.16 | 2.53 | NonCoding | -9 | 0.192 |
| candidate_1716 | - | B | 33 | 86.47 | 1.36 | NonCoding | -17 | 0.603 |
| candidate_1717 | - | B | 30 | 65.35 | 0.96 | NonCoding | -6.6 | 0.001 |
| candidate_1718 | - | B | 31 | 50.19 | 5.35 | NonCoding | -27 | 0.828 |
| candidate_1719 | - | B | 33 | 187.56 | 0.95 | NonCoding | -5.8 | 0.638 |
| candidate_1720 | - | B | 56 | 43.12 | 0.95 | NonCoding | -3.47 | 0.396 |
| candidate_1721 | - | B | 47 | 55.52 | 2.27 | NonCoding | -15.3 | 0.855 |
| candidate_1722 | - | B | 42 | 63.81 | 1.64 | NonCoding | -7.9 | 0.382 |
| candidate_1723 | - | C | 52 | 16.91 | 3.55 | NonCoding | -6.1 | 0.181 |
| candidate_1724 | - | C | 30 | 11.16 | 2.2 | NonCoding | -10 | 0.304 |
| candidate_1725 | - | C | 41 | 307.64 | 5.32 | NonCoding | -5.1 | 0.247 |
| candidate_1726 | - | C | 34 | 11.23 | 2.49 | NonCoding | -15.7 | 0.903 |
| candidate_1727 | - | C | 33 | 5.94 | 1.77 | NonCoding | -3.2 | 0.683 |
| candidate_1728 | - | C | 98 | 0.49 | 2.57 | NonCoding | -3.9 | 0.185 |
| candidate_1729 | - | C | 51 | 8.1 | 1.77 | NonCoding | -36.99 | 0.933 |
| candidate_1730 | - | C | 56 | 0.74 | 2.17 | NonCoding | -10 | 0.002 |
| candidate_1731 | - | C | 49 | 2.9 | 3.89 | NonCoding | -26.5 | 0.463 |
| candidate_1732 | - | C | 44 | 11.89 | 2.59 | NonCoding | -14.5 | 0.461 |
| candidate_1733 | - | C | 87 | 2.84 | 3.36 | NonCoding | -15.7 | 0.639 |
| candidate_1734 | - | C | 32 | 19.39 | 4.02 | NonCoding | -39.1 | 0.421 |
| candidate_1735 | - | C | 42 | 29.65 | 1.77 | NonCoding | -3.8 | 0.001 |
| candidate_1736 | - | C | 30 | 135.9 | 2.37 | NonCoding | -23.5 | 0.215 |
| candidate_1737 | - | C | 30 | 4.77 | 3.12 | NonCoding | -4.6 | 0.003 |
| candidate_1738 | - | C | 31 | 1.22 | 2.3 | NonCoding | -9.9 | 0.508 |
| candidate_1739 | - | C | 86 | 2.17 | 3.93 | NonCoding | -6 | 0.847 |
| candidate_1740 | - | C | 92 | 4.1 | 5.48 | NonCoding | -28.2 | 0.244 |
| candidate_1741 | - | C | 77 | 32.09 | 4.88 | NonCoding | -42.2 | 0.011 |
| candidate_1742 | - | C | 98 | 18.09 | 2.54 | NonCoding | -25.6 | 0.792 |
| candidate_1743 | - | C | 32 | 0 | 1.77 | NonCoding | -38.9 | 0.679 |
| candidate_1744 | - | C | 33 | 0 | 1.77 | NonCoding | -2.9 | 0.522 |
| candidate_1745 | - | C | 30 | 9.19 | 1.8 | NonCoding | -3.9 | 0.082 |
| candidate_1746 | - | C | 45 | 20.61 | 1.79 | NonCoding | -9.5 | 0.486 |
| candidate_1747 | - | C | 39 | 12.63 | 1.77 | NonCoding | -12.1 | 0.633 |
| candidate_1748 | - | C | 62 | 5.3 | 2.69 | NonCoding | -12 | 0.555 |
| candidate_1749 | - | C | 69 | 0.29 | 2.65 | NonCoding | -16.8 | 0.418 |
| candidate_1750 | - | C | 68 | 0.32 | 2.66 | NonCoding | -20.6 | 0.417 |
| candidate_1751 | - | C | 131 | 20.64 | 4.73 | NonCoding | -20.6 | 0.377 |
| candidate_1752 | - | C | 42 | 9.05 | 2.3 | NonCoding | -42.1 | 0.154 |
| candidate_1753 | - | C | 46 | 3 | 2.25 | NonCoding | -18.7 | 0.098 |
| candidate_1754 | - | C | 34 | 19.23 | 1.77 | NonCoding | -21.2 | 0.161 |
| candidate_1755 | - | C | 55 | 122.29 | 2.37 | NonCoding | -9 | 0.285 |
| candidate_1756 | - | C | 51 | 6.27 | 2.19 | NonCoding | -16.3 | 0.206 |
| candidate_1757 | - | C | 42 | 8.7 | 3.04 | NonCoding | -16 | 0.358 |
| candidate_1758 | - | C | 95 | 10.95 | 4.8 | NonCoding | -17.01 | 0.733 |
| candidate_1759 | - | C | 41 | 1.05 | 2.75 | NonCoding | -37.5 | 0.311 |
| candidate_1760 | - | C | 36 | 1.78 | 4.67 | NonCoding | -17 | 0.138 |
| candidate_1761 | - | C | 59 | 23.67 | 1.77 | NonCoding | -7.1 | 0.435 |
| candidate_1762 | - | C | 51 | 36.75 | 2.48 | NonCoding | -16.52 | 0.192 |
| candidate_1763 | - | C | 31 | 3.72 | 1.77 | NonCoding | -10.9 | 0.792 |
| candidate_1764 | - | C | 37 | 7.05 | 2.53 | NonCoding | -2.7 | 0.086 |
| candidate_1765 | - | C | 46 | 0 | 2.55 | NonCoding | -11.5 | 0.574 |
| candidate_1766 | - | C | 197 | 16.78 | 3.63 | NonCoding | -11.8 | 0.015 |
| candidate_1767 | - | C | 35 | 93.67 | 2.61 | NonCoding | -92.4 | 0.911 |
| candidate_1768 | - | C | 46 | 2.15 | 1.77 | NonCoding | -2.9 | 0.08 |
| candidate_1769 | - | C | 68 | 32.01 | 2.28 | NonCoding | -17.8 | 0.111 |
| candidate_1770 | - | C | 68 | 2.06 | 2.26 | NonCoding | -16.2 | 0.84 |
| candidate_1771 | - | C | 32 | 0 | 2.57 | NonCoding | -16.2 | 0.368 |
| candidate_1772 | - | C | 44 | 6.42 | 1.85 | NonCoding | -9.2 | 0.378 |
| candidate_1773 | - | C | 63 | 496 | 3.85 | NonCoding | -12.7 | 0.97 |
| candidate_1774 | - | C | 66 | 5.75 | 3.38 | NonCoding | -5.8 | 0.28 |
| candidate_1775 | - | C | 38 | 0 | 2.19 | NonCoding | -22.6 | 0.003 |
| candidate_1776 | - | C | 154 | 0.35 | 2.49 | NonCoding | -17.5 | 0.934 |
| candidate_1777 | - | C | 44 | 19.62 | 3.19 | NonCoding | -58 | 0.059 |
| candidate_1778 | - | C | 34 | 4.34 | 1.88 | NonCoding | -20.5 | 0.076 |
| candidate_1779 | - | C | 46 | 490.3 | 2.18 | NonCoding | -11 | 0.461 |
| candidate_1780 | - | C | 136 | 28.37 | 4.03 | NonCoding | -16 | 0.011 |
| candidate_1781 | - | C | 58 | 6.44 | 2.76 | NonCoding | -65.9 | 0.001 |
| candidate_1782 | - | C | 54 | 2.13 | 3.72 | NonCoding | -54.7 | 0.014 |
| candidate_1783 | - | C | 30 | 10.16 | 2.27 | NonCoding | -10 | 0.106 |
| candidate_1784 | - | C | 43 | 22.02 | 2.27 | NonCoding | -3.8 | 0.966 |
| candidate_1785 | - | C | 46 | 0.49 | 2.57 | NonCoding | -8.5 | 0.282 |
| candidate_1786 | - | C | 34 | 0.63 | 2.28 | NonCoding | -11.1 | 0.557 |
| candidate_1787 | - | C | 40 | 37.12 | 3.38 | NonCoding | -6.7 | 0.001 |
| candidate_1788 | - | C | 47 | 3.38 | 2.5 | NonCoding | -21.3 | 0.608 |
| candidate_1789 | - | C | 34 | 4.4 | 1.77 | NonCoding | -12.5 | 0.48 |
| candidate_1790 | - | C | 50 | 27.1 | 3.41 | NonCoding | -7.2 | 0.337 |
| candidate_1791 | - | C | 32 | 21.03 | 1.88 | NonCoding | -22.6 | 0.725 |
| candidate_1792 | - | C | 31 | 33.5 | 2.45 | NonCoding | -7.7 | 0.043 |
| candidate_1793 | - | C | 47 | 20.83 | 3.53 | NonCoding | -13.3 | 0.316 |
| candidate_1794 | - | C | 55 | 0.73 | 2.45 | NonCoding | -6.7 | 0.754 |
| candidate_1795 | - | C | 44 | 22.29 | 2.19 | NonCoding | -4 | 0.519 |
| candidate_1796 | - | C | 44 | 25.78 | 1.77 | NonCoding | -1.05 | 0.374 |
| candidate_1797 | - | C | 53 | 3.13 | 3.37 | NonCoding | -10.1 | 0.167 |
| candidate_1798 | - | C | 37 | 0.63 | 2.2 | NonCoding | -19.9 | 0.587 |
| candidate_1799 | - | C | 39 | 0 | 2.2 | NonCoding | -9.2 | 0.61 |
| candidate_1800 | - | C | 39 | 0 | 2.2 | NonCoding | -9.2 | 0.581 |
| candidate_1801 | - | C | 39 | 1.3 | 2.2 | NonCoding | -9.2 | 0.587 |
| candidate_1802 | - | C | 44 | 3.04 | 2.2 | NonCoding | -9.2 | 0.232 |
| candidate_1803 | - | C | 116 | 7.12 | 1.95 | NonCoding | -13.6 | 0.32 |
| candidate_1804 | - | C | 39 | 5.4 | 2.85 | NonCoding | -32.8 | 0.948 |
| candidate_1805 | - | C | 46 | 32.23 | 1.77 | NonCoding | -1.5 | 0.001 |
| candidate_1806 | - | C | 33 | 14.62 | 3.07 | NonCoding | -30.3 | 0.785 |
| candidate_1807 | - | C | 72 | 4.58 | 3.15 | NonCoding | -11.7 | 0.952 |
| candidate_1808 | - | C | 36 | 1.86 | 2.54 | NonCoding | -20.1 | 0.103 |
| candidate_1809 | - | C | 50 | 1.33 | 2.56 | NonCoding | -12.2 | 0.159 |
| candidate_1810 | - | C | 39 | 5.7 | 2.02 | NonCoding | -18.1 | 0.707 |
| candidate_1811 | - | C | 99 | 0 | 2.6 | NonCoding | -8.4 | 0.13 |
| candidate_1812 | - | C | 42 | 51.51 | 2.2 | NonCoding | -40.3 | 0.287 |
| candidate_1813 | - | C | 99 | 14.1 | 2.63 | NonCoding | -14 | 0.015 |
| candidate_1814 | - | C | 60 | 7.69 | 2.34 | NonCoding | -29.2 | 0.561 |
| candidate_1815 | - | C | 140 | 29.03 | 2.82 | NonCoding | -20.6 | 0.025 |
| candidate_1816 | - | C | 51 | 288.48 | 1.77 | NonCoding | -62.7 | 0.179 |
| candidate_1817 | - | C | 160 | 3.19 | 4.78 | NonCoding | -17.11 | 0.987 |
| candidate_1818 | - | C | 47 | 4.73 | 2.92 | NonCoding | -61.8 | 0.723 |
| candidate_1819 | - | C | 30 | 4.52 | 2.61 | NonCoding | -13.9 | 0.444 |
| candidate_1820 | - | C | 39 | 2.35 | 2.17 | NonCoding | -5 | 0.83 |
| candidate_1821 | - | C | 61 | 17.85 | 3.64 | NonCoding | -4.9 | 0.533 |
| candidate_1822 | - | C | 38 | 3.1 | 2.59 | NonCoding | -8.82 | 0.016 |
| candidate_1823 | - | C | 47 | 8.79 | 2.25 | NonCoding | -16.1 | 0.715 |
| candidate_1824 | - | C | 35 | 9.14 | 4.14 | NonCoding | -11.2 | 0.104 |
| candidate_1825 | - | C | 35 | 4.14 | 4.22 | NonCoding | -8.44 | 0.164 |
| candidate_1826 | - | C | 42 | 0.12 | 2.56 | NonCoding | -5 | 0.137 |
| candidate_1827 | - | C | 31 | 21.47 | 2.14 | NonCoding | -12.9 | 0.021 |
| candidate_1828 | - | C | 94 | 2.91 | 2.9 | NonCoding | -9.3 | 0.02 |
| candidate_1829 | - | C | 74 | 0.32 | 2.47 | NonCoding | -42.2 | 0.636 |
| candidate_1830 | - | C | 49 | 0 | 2.41 | NonCoding | -25.6 | 0.297 |
| candidate_1831 | - | C | 30 | 10.26 | 1.79 | NonCoding | -17.1 | 0.684 |
| candidate_1832 | - | C | 40 | 66.71 | 2.07 | NonCoding | -7.6 | 0.625 |
| candidate_1833 | - | C | 48 | 19.92 | 4.54 | NonCoding | -7.5 | 0.57 |
| candidate_1834 | - | C | 34 | 33.83 | 1.79 | NonCoding | -17.7 | 0.736 |
| candidate_1835 | - | C | 35 | 113.25 | 2.54 | NonCoding | -6.5 | 0.482 |
| candidate_1836 | - | C | 37 | 1.03 | 2.61 | NonCoding | -4.4 | 0.447 |
| candidate_1837 | - | C | 50 | 18.57 | 2.53 | NonCoding | -8.2 | 0.689 |
| candidate_1838 | - | C | 43 | 0 | 1.8 | NonCoding | -6.4 | 0.902 |
| candidate_1839 | - | C | 102 | 104.05 | 3.33 | NonCoding | -9.9 | 0.139 |
| candidate_1840 | - | C | 50 | 7.02 | 3.95 | NonCoding | -31.7 | 0.41 |
| candidate_1841 | - | C | 62 | 9.46 | 2.1 | NonCoding | -3.8 | 0.895 |
| candidate_1842 | - | C | 42 | 8 | 4.85 | NonCoding | -18.7 | 0.801 |
| candidate_1843 | - | C | 61 | 40.39 | 1.77 | NonCoding | -7.7 | 0.962 |
| candidate_1844 | - | C | 72 | 4.58 | 5.5 | NonCoding | -13.4 | 0.358 |
| candidate_1845 | - | C | 36 | 12.89 | 3.28 | NonCoding | -15.5 | 0.934 |
| candidate_1846 | - | C | 310 | 0.04 | 2.52 | NonCoding | -1 | 0.001 |
| candidate_1847 | - | C | 33 | 5.53 | 1.77 | NonCoding | -131 | 0.015 |
| candidate_1848 | - | C | 59 | 0.03 | 2.54 | NonCoding | -10.8 | 0.759 |
| candidate_1849 | - | C | 33 | 3.71 | 1.77 | NonCoding | -15.56 | 0.931 |
| candidate_1850 | - | C | 64 | 0.18 | 3.34 | NonCoding | -2.3 | 0.637 |
| candidate_1851 | - | C | 30 | 19.29 | 3.96 | NonCoding | -25.4 | 0.862 |
| candidate_1852 | - | C | 170 | 14.82 | 3.76 | NonCoding | -3.5 | 0.066 |
| candidate_1853 | - | C | 52 | 34.53 | 2.56 | NonCoding | -76.3 | 0.026 |
| candidate_1854 | - | C | 57 | 18.34 | 2.14 | NonCoding | -22 | 0.118 |
| candidate_1855 | - | C | 34 | 10.2 | 3.04 | NonCoding | -27.7 | 0.132 |
| candidate_1856 | - | C | 38 | 0.1 | 2.52 | NonCoding | -14.8 | 0.358 |
| candidate_1857 | - | C | 39 | 0.45 | 2.49 | NonCoding | -10.5 | 0.068 |
| candidate_1858 | - | C | 34 | 1.37 | 2.58 | NonCoding | -22.7 | 0.803 |
| candidate_1859 | - | C | 79 | 2.29 | 4.68 | NonCoding | -2.7 | 0.404 |
| candidate_1860 | - | C | 79 | 15.1 | 2.15 | NonCoding | -15.2 | 0.861 |
| candidate_1861 | - | C | 87 | 0 | 1.95 | NonCoding | -20.4 | 0.078 |
| candidate_1862 | - | C | 94 | 0.16 | 2.54 | NonCoding | -46.61 | 0.138 |
| candidate_1863 | - | C | 156 | 0.11 | 2.57 | NonCoding | -37.7 | 0.451 |
| candidate_1864 | - | C | 97 | 59.27 | 3.07 | NonCoding | -64.7 | 0.396 |
| candidate_1865 | - | C | 45 | 1.61 | 2.61 | NonCoding | -26.4 | 0.462 |
| candidate_1866 | - | C | 40 | 17.24 | 1.79 | NonCoding | -8.8 | 0.004 |
| candidate_1867 | - | C | 71 | 0.56 | 2.2 | NonCoding | -16.8 | 0.205 |
| candidate_1868 | - | C | 62 | 7.68 | 2.84 | NonCoding | -21.9 | 0.033 |
| candidate_1869 | - | C | 42 | 6.63 | 2.53 | NonCoding | -20.4 | 0.439 |
| candidate_1870 | - | C | 34 | 0 | 2.36 | NonCoding | -10.1 | 0.573 |
| candidate_1871 | - | C | 50 | 0.8 | 2.86 | NonCoding | -11.5 | 0.173 |
| candidate_1872 | - | C | 37 | 0 | 2.08 | NonCoding | -12.2 | 0.112 |
| candidate_1873 | - | C | 48 | 6.18 | 2.31 | NonCoding | -13.8 | 0.801 |
| candidate_1874 | - | C | 208 | 7.56 | 3.09 | NonCoding | -8.2 | 0.024 |
| candidate_1875 | - | C | 83 | 19.96 | 3.71 | NonCoding | -100 | 0.35 |
| candidate_1876 | - | C | 41 | 50.12 | 2.21 | NonCoding | -24.3 | 0.758 |
| candidate_1877 | - | C | 206 | 0 | 1.77 | NonCoding | -9.8 | 0.017 |
| candidate_1878 | - | C | 151 | 1.1 | 2.5 | NonCoding | -119.6 | 0.031 |
| candidate_1879 | - | C | 64 | 19.6 | 3.32 | NonCoding | -65.9 | 0.059 |
| candidate_1880 | - | C | 41 | 9.43 | 3.26 | NonCoding | -30.4 | 0.039 |
| candidate_1881 | - | C | 65 | 1.53 | 3.12 | NonCoding | -20.6 | 0.001 |
| candidate_1882 | - | C | 72 | 26 | 4.44 | NonCoding | -43.7 | 0.028 |
| candidate_1883 | - | C | 68 | 5 | 2.4 | NonCoding | -33.3 | 0.104 |
| candidate_1884 | - | C | 213 | 0 | 2.38 | NonCoding | -20.7 | 0.003 |
| candidate_1885 | - | C | 166 | 0 | 2.78 | NonCoding | -138.14 | 0.035 |
| candidate_1886 | - | C | 56 | 0 | 3.11 | NonCoding | -81.2 | 0.299 |
| candidate_1887 | - | C | 38 | 24.59 | 4.8 | NonCoding | -25.3 | 0.065 |
| candidate_1888 | - | C | 39 | 3.18 | 2.28 | NonCoding | -7.8 | 0.803 |
| candidate_1889 | - | C | 77 | 0.23 | 2.32 | NonCoding | -10.6 | 0.781 |
| candidate_1890 | - | C | 39 | 0 | 1.8 | NonCoding | -26.9 | 0.382 |
| candidate_1891 | - | C | 39 | 0 | 1.8 | NonCoding | -18 | 0.405 |
| candidate_1892 | - | C | 39 | 0 | 1.8 | NonCoding | -18 | 0.381 |
| candidate_1893 | - | C | 39 | 0.53 | 1.8 | NonCoding | -18 | 0.387 |
| candidate_1894 | - | C | 39 | 2.03 | 1.8 | NonCoding | -18 | 0.425 |
| candidate_1895 | - | C | 106 | 4.26 | 3.95 | NonCoding | -18 | 0.747 |
| candidate_1896 | - | C | 45 | 0 | 1.77 | NonCoding | -32.8 | 0.012 |
| candidate_1897 | - | C | 45 | 0 | 1.77 | NonCoding | -24.6 | 0.007 |
| candidate_1898 | - | C | 69 | 6.36 | 1.77 | NonCoding | -24.6 | 0.016 |
| candidate_1899 | - | C | 31 | 292.53 | 2.92 | NonCoding | -26.3 | 0.122 |
| candidate_1900 | - | C | 65 | 35.62 | 2.58 | NonCoding | -11.7 | 0.045 |
| candidate_1901 | - | C | 52 | 0.51 | 1.79 | NonCoding | -23 | 0.319 |
| candidate_1902 | - | C | 33 | 4 | 1.77 | NonCoding | -20.3 | 0.045 |
| candidate_1903 | - | C | 58 | 0.14 | 3.19 | NonCoding | -14.9 | 0.001 |
| candidate_1904 | - | C | 35 | 16.17 | 2.31 | NonCoding | -41.6 | 0.001 |
| candidate_1905 | - | C | 45 | 2.52 | 2.3 | NonCoding | -22.8 | 0.075 |
| candidate_1906 | - | C | 34 | 70.83 | 2.32 | NonCoding | -26.5 | 0.001 |
| candidate_1907 | - | C | 47 | 4.81 | 2.34 | NonCoding | -14.7 | 0.206 |
| candidate_1908 | - | C | 44 | 0.49 | 2.59 | NonCoding | -20.5 | 0.699 |
| candidate_1909 | - | C | 136 | 5.46 | 4.62 | NonCoding | -10.9 | 0.251 |
| candidate_1910 | - | C | 32 | 2.45 | 2.81 | NonCoding | -66.2 | 0.767 |
| candidate_1911 | - | C | 32 | 2.58 | 1.78 | NonCoding | -6.2 | 0.275 |
| candidate_1912 | - | C | 42 | 1.02 | 2.49 | NonCoding | -11.3 | 0.032 |
| candidate_1913 | - | C | 41 | 3.1 | 2.17 | NonCoding | -17.7 | 0.058 |
| candidate_1914 | - | C | 58 | 17.02 | 2.7 | NonCoding | -16.3 | 0.001 |
| candidate_1915 | - | C | 60 | 60.75 | 3.23 | NonCoding | -45 | 0.001 |
| candidate_1916 | - | C | 43 | 8.43 | 5.2 | NonCoding | -40.5 | 0.012 |
| candidate_1917 | - | C | 136 | 25.53 | 2.46 | NonCoding | -17 | 0.019 |
| candidate_1918 | - | C | 43 | 3.02 | 3.6 | NonCoding | -67.3 | 0.081 |
| candidate_1919 | - | C | 31 | 24.31 | 4.22 | NonCoding | -10.4 | 0.413 |
| candidate_1920 | - | C | 73 | 31.96 | 4.59 | NonCoding | -3.7 | 0.178 |
| candidate_1921 | - | C | 35 | 0.92 | 5.11 | NonCoding | -17.9 | 0.016 |
| candidate_1922 | - | C | 33 | 2.32 | 3.68 | NonCoding | -10.2 | 0.579 |
| candidate_1923 | - | C | 36 | 1.08 | 4.88 | NonCoding | -11.2 | 0.031 |
| candidate_1924 | - | C | 92 | 3.27 | 3.09 | NonCoding | -11 | 0.002 |
| candidate_1925 | - | C | 40 | 9.68 | 2.14 | NonCoding | -26.6 | 0.261 |
| candidate_1926 | - | C | 76 | 544.38 | 2.78 | NonCoding | -9.3 | 0.035 |
| candidate_1927 | - | C | 48 | 22.14 | 2.77 | NonCoding | -31.7 | 0.153 |
| candidate_1928 | - | C | 44 | 29.62 | 2.43 | NonCoding | -16.6 | 0.621 |
| candidate_1929 | - | C | 39 | 18.48 | 2.4 | NonCoding | -6 | 0.936 |
| candidate_1930 | - | C | 53 | 0.04 | 2.03 | NonCoding | -5.2 | 0.674 |
| candidate_1931 | - | C | 55 | 9.93 | 1.79 | NonCoding | -17.2 | 0.986 |
| candidate_1932 | - | C | 114 | 9.57 | 4.26 | NonCoding | -13.8 | 0.363 |
| candidate_1933 | - | C | 32 | 2.88 | 2.97 | NonCoding | -40.93 | 0.819 |
| candidate_1934 | - | C | 35 | 57.06 | 3.04 | NonCoding | -3.2 | 0.443 |
| candidate_1935 | - | C | 67 | 21.9 | 3.06 | NonCoding | -6.2 | 0.366 |
| candidate_1936 | - | C | 48 | 13.04 | 2.47 | NonCoding | -15.6 | 0.259 |
| candidate_1937 | - | C | 48 | 0.92 | 3.24 | NonCoding | -10.9 | 0.752 |
| candidate_1938 | - | C | 98 | 88.57 | 5.18 | NonCoding | -14.8 | 0.292 |
| candidate_1939 | - | C | 45 | 0 | 1.8 | NonCoding | -40.2 | 0.013 |
| candidate_1940 | - | C | 45 | 0 | 1.8 | NonCoding | -28.9 | 0.021 |
| candidate_1941 | - | C | 54 | 0.11 | 2.48 | NonCoding | -28.9 | 0.012 |
| candidate_1942 | - | C | 35 | 6.36 | 1.77 | NonCoding | -31.7 | 0.58 |
| candidate_1943 | - | C | 41 | 0.9 | 2.44 | NonCoding | -7.9 | 0.597 |
| candidate_1944 | - | C | 35 | 0.5 | 1.79 | NonCoding | -11 | 0.707 |
| candidate_1945 | - | C | 73 | 36.66 | 2.32 | NonCoding | -8.8 | 0.002 |
| candidate_1946 | - | C | 31 | 4.03 | 4.33 | NonCoding | -29.6 | 0.706 |
| candidate_1947 | - | C | 42 | 122.02 | 1.85 | NonCoding | -2.2 | 0.224 |
| candidate_1948 | - | C | 50 | 12.49 | 2.66 | NonCoding | -8.7 | 0.347 |
| candidate_1949 | - | C | 98 | 0.11 | 2.56 | NonCoding | -10.2 | 0.377 |
