# Supplementary material for: A Genome-Wide Identification Analysis of Small Regulatory RNAs in Mycobacterium tuberculosis by RNA-Seq and Conservation Analysis
Source: PLoS One. 2012 Mar 28;7(3):e32723. doi: 10.1371/journal.pone.0032723 (PMC3314655; doi:10.1371/journal.pone.0032723)
Supplement: Table S3 — Comparison with Livny J, et al., 2007 [12] putative sRNA encoding loci. (DOC) [file pone.0032723.s004.doc]

| **Putative sRNAs encoding loci identified in Livny J., et al. [12]** | | | | **Candidate identified by our method** | | | | | | |
| --- | --- | --- | --- | --- | --- | --- | --- | --- | --- | --- |
| **sRNA** | **Start** | **End** | **strand** | **Id** | **Type** | **Start** | **End** | **strand** | **meanExpr** | **meanCons** |
| Candidate_68 | 293495 | 293710 | + | candidate_29 | A | 293604 | 293662 | + | 616.32 | 1.91 |
| Candidate_68 | 293495 | 293710 | + | candidate_676 | C | 293668 | 293710 | + | 24.53 | 1.77 |
| Candidate_12 | 565395 | 565796 | + | candidate_687 | C | 565686 | 565731 | + | 15.98 | 1.79 |
| Candidate_14 | 767328 | 767683 | + | candidate_98 | A | 767320 | 767382 | + | 165.11 | 1.69 |
| Candidate_11 | 786040 | 786148 | + | candidate_103 | A | 786036 | 786069 | + | 157.35 | 0.14 |
| Candidate_99 | 786085 | 786148 | + | candidate_103 | A | 786036 | 786069 | + | 157.35 | 0.14 |
| Candidate_101 | 806182 | 806334 | + | candidate_1055 | A | 806143 | 806218 | - | 114.38 | 0.90 |
| Candidate_15 | 905105 | 905221 | - | candidate_1074 | A | 905114 | 905163 | - | 233.2 | 4.63 |
| Candidate_27 | 1058067 | 1058131 | - | candidate_1780 | C | 1057971 | 1058107 | - | 28.37 | 4.03 |
| Candidate_29 | 1127885 | 1127997 | + | candidate_619 | B | 1127911 | 1127951 | + | 99.17 | 2.64 |
| Candidate_29 | 1127885 | 1127997 | + | candidate_140 | A | 1127953 | 1128073 | + | 761.75 | 0.51 |
| Candidate_33 | 1271915 | 1272270 | - | candidate_1792 | C | 1271968 | 1271999 | - | 33.5 | 2.45 |
| Candidate_35 | 1313343 | 1313724 | - | candidate_1151 | A | 1313343 | 1313400 | - | 5034.5 | 1.37 |
| Candidate_39 | 1471579 | 1471794 | + | candidate_190 | A | 1471657 | 1471737 | + | 23731.14 | 3.03 |
| Candidate_39 | 1471579 | 1471794 | + | candidate_191 | A | 1471783 | 1471846 | + | 574.02 | 0.14 |
| Candidate_40 | 1473394 | 1473651 | - | candidate_1696 | B | 1473471 | 1473502 | - | 831.16 | 2.80 |
| Candidate_40 | 1473394 | 1473651 | - | candidate_1187 | A | 1473503 | 1473928 | - | 274.01 | 1.09 |
| Candidate_2 | 1735473 | 1735808 | + | candidate_753 | C | 1735491 | 1735541 | + | 98.47 | 2.76 |
| Candidate_2 | 1735473 | 1735808 | + | candidate_634 | B | 1735542 | 1735577 | + | 39.58 | 3.52 |
| Candidate_2 | 1735473 | 1735808 | + | candidate_754 | C | 1735578 | 1735644 | + | 8.75 | 2.70 |
| Candidate_2 | 1735473 | 1735808 | + | candidate_635 | B | 1735645 | 1735677 | + | 64.42 | 2.20 |
| Candidate_2 | 1735473 | 1735808 | + | candidate_224 | A | 1735693 | 1735747 | + | 107.47 | 0.25 |
| Candidate_2 | 1735473 | 1735808 | + | candidate_636 | B | 1735777 | 1735818 | + | 354.21 | 1.77 |
| Candidate_48 | 1852037 | 1852185 | + | candidate_760 | C | 1852139 | 1852177 | + | 10.44 | 2.59 |
| Candidate_55 | 2307069 | 2307292 | - | candidate_1701 | B | 2307105 | 2307138 | - | 37.82 | 2.17 |
| Candidate_59 | 2409529 | 2409695 | + | candidate_778 | C | 2409573 | 2409675 | + | 82.82 | 3.33 |
| Candidate_4 | 2849407 | 2849612 | + | candidate_801 | C | 2849542 | 2849576 | + | 0.57 | 2.58 |
| Candidate_70 | 2995891 | 2996104 | - | candidate_1862 | C | 2996009 | 2996103 | - | 0.16 | 2.54 |
| Candidate_71 | 3023434 | 3023524 | + | candidate_647 | B | 3023454 | 3023490 | + | 59 | 1.42 |
| Candidate_73 | 3217645 | 3217713 | + | candidate_824 | C | 3217666 | 3217738 | + | 14.59 | 4.44 |
| Candidate_77 | 3386816 | 3387055 | + | candidate_840 | C | 3386925 | 3386994 | + | 8.27 | 1.77 |
| Candidate_79 | 3551168 | 3551230 | - | candidate_1903 | C | 3551161 | 3551219 | - | 0.14 | 3.19 |
| Candidate_80 | 3650085 | 3650233 | - | candidate_1545 | A | 3650186 | 3650276 | - | 117.76 | 0.05 |
| Candidate_24 | 4100668 | 4100980 | + | candidate_561 | A | 4100684 | 4100816 | + | 167.04 | 2.37 |
| Candidate_24 | 4100668 | 4100980 | + | candidate_654 | B | 4100817 | 4100865 | + | 60.16 | 2.55 |
| Candidate_24 | 4100668 | 4100980 | + | candidate_562 | A | 4100900 | 4100977 | + | 322.86 | 0.44 |
| Candidate_91 | 4156788 | 4156856 | - | candidate_1939 | C | 4156803 | 4156848 | - | 0 | 1.80 |
| Candidate_25 | 4352898 | 4352973 | + | candidate_595 | A | 4352898 | 4352979 | + | 602.74 | 0.25 |
