# Supplementary material for: A Genome-Wide Identification Analysis of Small Regulatory RNAs in Mycobacterium tuberculosis by RNA-Seq and Conservation Analysis
Source: PLoS One. 2012 Mar 28;7(3):e32723. doi: 10.1371/journal.pone.0032723 (PMC3314655; doi:10.1371/journal.pone.0032723)
Supplement: Table S4 — BLASTN output of comparison between candidates sequences and Rfam V.10 database. (DOC) [file pone.0032723.s005.doc]

| candidate_184 | RF00177;SSU_rRNA_5;AF339595.1/32-484 | 100 | 19 | 0 | 0 | 14 | 32 | 105 | 123 | 0.01 | 38.2 |
| --- | --- | --- | --- | --- | --- | --- | --- | --- | --- | --- | --- |
| candidate_407 | RF00005;tRNA;ACEB01000020.1/106278-106349 | 100 | 25 | 0 | 0 | 57 | 81 | 48 | 72 | 9.00E-006 | 50.1 |
| candidate_557 | RF00005;tRNA;ABJB010844889.1/97-201 | 100 | 19 | 0 | 0 | 8 | 26 | 44 | 62 | 0.01 | 38.2 |
| candidate_634 | RF00380;ykoK;AASN01000027.1/80343-80511 | 100 | 36 | 0 | 0 | 1 | 36 | 34 | 69 | 4.00E-013 | 71.9 |
| candidate_634 | RF00380;ykoK;CP000854.1/361669-361839 | 100 | 33 | 0 | 0 | 4 | 36 | 37 | 69 | 2.00E-011 | 65.9 |
| candidate_634 | RF00380;ykoK;ABIN01000121.1/2753-2927 | 97.14 | 35 | 1 | 0 | 1 | 35 | 34 | 68 | 3.00E-010 | 61.9 |
| candidate_634 | RF00380;ykoK;ACBV01000002.1/67696-67865 | 96.97 | 33 | 1 | 0 | 4 | 36 | 37 | 69 | 5.00E-009 | 58.0 |
| candidate_634 | RF00380;ykoK;ABVA01000002.1/216092-216271 | 100 | 25 | 0 | 0 | 12 | 36 | 47 | 71 | 1.00E-006 | 50.1 |
| candidate_634 | RF00380;ykoK;CP000854.1/3273427-3273602 | 93.94 | 33 | 2 | 0 | 4 | 36 | 37 | 69 | 1.00E-006 | 50.1 |
| candidate_635 | RF00380;ykoK;ABIN01000121.1/2753-2927 | 100 | 32 | 0 | 0 | 1 | 32 | 142 | 173 | 7.00E-011 | 63.9 |
| candidate_635 | RF00380;ykoK;ACBV01000002.1/67696-67865 | 96.97 | 33 | 1 | 0 | 1 | 33 | 137 | 169 | 5.00E-009 | 58.0 |
| candidate_635 | RF00380;ykoK;CP000854.1/3273427-3273602 | 96.77 | 31 | 1 | 0 | 1 | 31 | 143 | 173 | 7.00E-008 | 54.0 |
| candidate_635 | RF00380;ykoK;CU458896.1/2766928-2767096 | 96.43 | 28 | 1 | 0 | 5 | 32 | 140 | 167 | 4.00E-006 | 48.1 |
| candidate_635 | RF00380;ykoK;CU458896.1/4831017-4831187 | 96.15 | 26 | 1 | 0 | 5 | 30 | 142 | 167 | 7.00E-005 | 44.1 |
| candidate_635 | RF00380;ykoK;ABTI01000017.1/77864-78032 | 96.15 | 26 | 1 | 0 | 5 | 30 | 140 | 165 | 7.00E-005 | 44.1 |
| candidate_635 | RF00380;ykoK;CP000854.1/361669-361839 | 92.86 | 28 | 2 | 0 | 5 | 32 | 142 | 169 | 0 | 40.1 |
| candidate_661 | RF00080;yybP-ykoY;ABGM02000021.1/374186-374293 | 100 | 52 | 0 | 0 | 1 | 52 | 5 | 56 | 2.00E-022 | 103 |
| candidate_661 | RF00080;yybP-ykoY;ACBV01000005.1/9558-9662 | 94 | 50 | 3 | 0 | 1 | 50 | 5 | 54 | 4.00E-014 | 75.8 |
| candidate_661 | RF00080;yybP-ykoY;CP000325.1/5567393-5567499 | 93.48 | 46 | 3 | 0 | 1 | 46 | 5 | 50 | 9.00E-012 | 67.9 |
| candidate_661 | RF00080;yybP-ykoY;CP000518.1/5381878-5381981 | 90.7 | 43 | 4 | 0 | 3 | 45 | 7 | 49 | 1.00E-007 | 54.0 |
| candidate_661 | RF00080;yybP-ykoY;AP006618.1/107400-107521 | 87.8 | 41 | 5 | 0 | 3 | 43 | 7 | 47 | 5.00E-004 | 42.1 |
| candidate_661 | RF00080;yybP-ykoY;ABYC01000041.1/23151-23282 | 100 | 20 | 0 | 0 | 3 | 22 | 7 | 26 | 0 | 40.1 |
| candidate_661 | RF00080;yybP-ykoY;ABYA01000017.1/19138-19256 | 100 | 20 | 0 | 0 | 4 | 23 | 8 | 27 | 0 | 40.1 |
| candidate_661 | RF00080;yybP-ykoY;CP000481.1/2229255-2229359 | 100 | 20 | 0 | 0 | 3 | 22 | 7 | 26 | 0 | 40.1 |
| candidate_661 | RF00080;yybP-ykoY;ABVA01000001.1/154040-154179 | 86.36 | 44 | 6 | 0 | 5 | 48 | 9 | 52 | 0 | 40.1 |
| candidate_661 | RF00080;yybP-ykoY;AAMN01000001.1/930627-930731 | 87.18 | 39 | 5 | 0 | 5 | 43 | 9 | 47 | 0.01 | 38.2 |
| candidate_681 | RF00059;TPP;CP000656.1/181563-181649 | 94.12 | 85 | 5 | 0 | 1 | 85 | 2 | 86 | 6.00E-030 | 129 |
| candidate_681 | RF00059;TPP;CP000480.1/878044-878154 | 93.02 | 86 | 5 | 1 | 1 | 85 | 10 | 95 | 9.00E-026 | 115 |
| candidate_681 | RF00059;TPP;ABIN01000033.1/44565-44683 | 100 | 48 | 0 | 0 | 39 | 86 | 57 | 104 | 8.00E-020 | 95.6 |
| candidate_681 | RF00059;TPP;CP000325.1/3132109-3132239 | 93.75 | 48 | 3 | 0 | 38 | 85 | 68 | 115 | 1.00E-012 | 71.9 |
| candidate_681 | RF00059;TPP;ACBV01000011.1/47244-47411 | 93.62 | 47 | 3 | 0 | 39 | 85 | 106 | 152 | 5.00E-012 | 69.9 |
| candidate_681 | RF00059;TPP;CP000518.1/611439-611556 | 93.33 | 45 | 3 | 0 | 42 | 86 | 59 | 103 | 7.00E-011 | 65.9 |
| candidate_681 | RF00059;TPP;FM211192.1/382684-382822 | 97.14 | 35 | 1 | 0 | 53 | 87 | 90 | 124 | 1.00E-009 | 61.9 |
| candidate_681 | RF00059;TPP;ABUZ01000090.1/7665-7778 | 100 | 30 | 0 | 0 | 56 | 85 | 69 | 98 | 5.00E-009 | 60.0 |
| candidate_681 | RF00059;TPP;ABUI01000056.1/4772-4883 | 100 | 30 | 0 | 0 | 56 | 85 | 67 | 96 | 5.00E-009 | 60.0 |
| candidate_681 | RF00059;TPP;AE015451.1/3613932-3614046 | 100 | 29 | 0 | 0 | 56 | 84 | 70 | 98 | 2.00E-008 | 58.0 |
| candidate_681 | RF00059;TPP;AAMO01000007.1/212536-212652 | 100 | 29 | 0 | 0 | 56 | 84 | 71 | 99 | 2.00E-008 | 58.0 |
| candidate_681 | RF00059;TPP;CP000613.1/3179378-3179480 | 96.88 | 32 | 1 | 0 | 56 | 87 | 58 | 89 | 7.00E-008 | 56.0 |
| candidate_681 | RF00059;TPP;CU458896.1/4286819-4286934 | 90.91 | 44 | 4 | 0 | 42 | 85 | 57 | 100 | 7.00E-008 | 56.0 |
| candidate_681 | RF00059;TPP;AM942444.1/505025-505143 | 96.88 | 32 | 1 | 0 | 48 | 79 | 64 | 95 | 7.00E-008 | 56.0 |
| candidate_681 | RF00059;TPP;AACY020182334.1/1203-1309 | 96.77 | 31 | 1 | 0 | 56 | 86 | 61 | 91 | 3.00E-007 | 54.0 |
| candidate_681 | RF00059;TPP;CP001340.1/2260162-2260267 | 96.77 | 31 | 1 | 0 | 56 | 86 | 61 | 91 | 3.00E-007 | 54.0 |
| candidate_681 | RF00059;TPP;ACEX01000579.1/4826-4939 | 96.77 | 31 | 1 | 0 | 56 | 86 | 69 | 99 | 3.00E-007 | 54.0 |
| candidate_681 | RF00059;TPP;ABYC01000141.1/10523-10634 | 96.77 | 31 | 1 | 0 | 56 | 86 | 65 | 95 | 3.00E-007 | 54.0 |
| candidate_681 | RF00059;TPP;ABYA01000416.1/19428-19539 | 96.77 | 31 | 1 | 0 | 56 | 86 | 67 | 97 | 3.00E-007 | 54.0 |
| candidate_681 | RF00059;TPP;AACY022573432.1/258-340 | 96.67 | 30 | 1 | 0 | 56 | 85 | 54 | 83 | 1.00E-006 | 52.0 |
| candidate_681 | RF00059;TPP;AACY023342574.1/1295-1400 | 96.67 | 30 | 1 | 0 | 56 | 85 | 61 | 90 | 1.00E-006 | 52.0 |
| candidate_681 | RF00059;TPP;AP008955.1/944363-944479 | 96.67 | 30 | 1 | 0 | 55 | 84 | 68 | 97 | 1.00E-006 | 52.0 |
| candidate_681 | RF00059;TPP;BAAW01000620.1/1767-1890 | 96.55 | 29 | 1 | 0 | 56 | 84 | 78 | 106 | 4.00E-006 | 50.1 |
| candidate_681 | RF00059;TPP;AACY023286694.1/565-662 | 96.55 | 29 | 1 | 0 | 56 | 84 | 52 | 80 | 4.00E-006 | 50.1 |
| candidate_681 | RF00059;TPP;AACY023445401.1/64-170 | 96.55 | 29 | 1 | 0 | 56 | 84 | 60 | 88 | 4.00E-006 | 50.1 |
| candidate_681 | RF00059;TPP;AAFY01006381.1/487-584 | 96.55 | 29 | 1 | 0 | 56 | 84 | 53 | 81 | 4.00E-006 | 50.1 |
| candidate_681 | RF00059;TPP;EU016624.1/15749-15877 | 96.55 | 29 | 1 | 0 | 56 | 84 | 83 | 111 | 4.00E-006 | 50.1 |
| candidate_681 | RF00059;TPP;AAQX01000956.1/26594-26717 | 96.55 | 29 | 1 | 0 | 56 | 84 | 78 | 106 | 4.00E-006 | 50.1 |
| candidate_681 | RF00059;TPP;ABTR01000007.1/37535-37641 | 96.55 | 29 | 1 | 0 | 56 | 84 | 62 | 90 | 4.00E-006 | 50.1 |
| candidate_681 | RF00059;TPP;CP001127.1/126644-126743 | 96.55 | 29 | 1 | 0 | 56 | 84 | 54 | 82 | 4.00E-006 | 50.1 |
| candidate_681 | RF00059;TPP;ABWL01000008.1/104697-104794 | 96.55 | 29 | 1 | 0 | 56 | 84 | 52 | 80 | 4.00E-006 | 50.1 |
| candidate_681 | RF00059;TPP;ACDJ01000024.1/183456-183678 | 96.55 | 29 | 1 | 0 | 56 | 84 | 177 | 205 | 4.00E-006 | 50.1 |
| candidate_681 | RF00059;TPP;ACKY01000057.1/29544-29637 | 96.55 | 29 | 1 | 0 | 56 | 84 | 49 | 77 | 4.00E-006 | 50.1 |
| candidate_681 | RF00059;TPP;CP000230.1/2319857-2319989 | 96.55 | 29 | 1 | 0 | 56 | 84 | 87 | 115 | 4.00E-006 | 50.1 |
| candidate_681 | RF00059;TPP;CP001189.1/1550144-1550252 | 96.55 | 29 | 1 | 0 | 56 | 84 | 63 | 91 | 4.00E-006 | 50.1 |
| candidate_681 | RF00059;TPP;ACCU01000015.1/491844-491950 | 96.55 | 29 | 1 | 0 | 56 | 84 | 62 | 90 | 4.00E-006 | 50.1 |
| candidate_681 | RF00059;TPP;AP009384.1/2941928-2942063 | 96.55 | 29 | 1 | 0 | 56 | 84 | 90 | 118 | 4.00E-006 | 50.1 |
| candidate_681 | RF00059;TPP;AP009384.1/2089229-2089334 | 96.55 | 29 | 1 | 0 | 56 | 84 | 60 | 88 | 4.00E-006 | 50.1 |
| candidate_681 | RF00059;TPP;CP000774.1/2328672-2328784 | 96.55 | 29 | 1 | 0 | 56 | 84 | 68 | 96 | 4.00E-006 | 50.1 |
| candidate_681 | RF00059;TPP;CP000301.1/2359100-2359237 | 96.55 | 29 | 1 | 0 | 56 | 84 | 92 | 120 | 4.00E-006 | 50.1 |
| candidate_681 | RF00059;TPP;CP000463.1/2282845-2282986 | 96.55 | 29 | 1 | 0 | 56 | 84 | 96 | 124 | 4.00E-006 | 50.1 |
| candidate_681 | RF00059;TPP;BX572604.1/199848-199985 | 96.55 | 29 | 1 | 0 | 56 | 84 | 93 | 121 | 4.00E-006 | 50.1 |
| candidate_681 | RF00059;TPP;CP000250.1/2225558-2225706 | 96.55 | 29 | 1 | 0 | 56 | 84 | 104 | 132 | 4.00E-006 | 50.1 |
| candidate_681 | RF00059;TPP;CP000283.1/3812110-3812250 | 96.55 | 29 | 1 | 0 | 56 | 84 | 96 | 124 | 4.00E-006 | 50.1 |
| candidate_681 | RF00059;TPP;CP001196.1/2988271-2988380 | 96.55 | 29 | 1 | 0 | 56 | 84 | 65 | 93 | 4.00E-006 | 50.1 |
| candidate_681 | RF00059;TPP;CP000319.1/3185661-3185795 | 96.55 | 29 | 1 | 0 | 56 | 84 | 90 | 118 | 4.00E-006 | 50.1 |
| candidate_681 | RF00059;TPP;CP000115.1/2687699-2687833 | 96.55 | 29 | 1 | 0 | 56 | 84 | 90 | 118 | 4.00E-006 | 50.1 |
| candidate_681 | RF00059;TPP;BA000040.2/7330682-7330809 | 96.55 | 29 | 1 | 0 | 56 | 84 | 83 | 111 | 4.00E-006 | 50.1 |
| candidate_681 | RF00059;TPP;CP000494.1/6469821-6469931 | 96.55 | 29 | 1 | 0 | 56 | 84 | 65 | 93 | 4.00E-006 | 50.1 |
| candidate_681 | RF00059;TPP;CU234118.1/5912069-5912178 | 96.55 | 29 | 1 | 0 | 56 | 84 | 65 | 93 | 4.00E-006 | 50.1 |
| candidate_681 | RF00059;TPP;AATP01000003.1/96792-96900 | 96.55 | 29 | 1 | 0 | 56 | 84 | 63 | 91 | 4.00E-006 | 50.1 |
| candidate_681 | RF00059;TPP;CP000747.1/3325605-3325712 | 96.55 | 29 | 1 | 0 | 56 | 84 | 63 | 91 | 4.00E-006 | 50.1 |
| candidate_681 | RF00059;TPP;ABRU01000011.1/593-701 | 96.55 | 29 | 1 | 0 | 56 | 84 | 63 | 91 | 4.00E-006 | 50.1 |
| candidate_681 | RF00059;TPP;CP000560.1/1522149-1522254 | 96.55 | 29 | 1 | 0 | 56 | 84 | 61 | 89 | 4.00E-006 | 50.1 |
| candidate_681 | RF00059;TPP;ABUH01000009.1/55076-55187 | 96.55 | 29 | 1 | 0 | 56 | 84 | 66 | 94 | 4.00E-006 | 50.1 |
| candidate_681 | RF00059;TPP;AAYI02000004.1/1724296-1724403 | 91.89 | 37 | 3 | 0 | 48 | 84 | 55 | 91 | 4.00E-006 | 50.1 |
| candidate_681 | RF00059;TPP;ACKQ01000008.1/25396-25503 | 93.75 | 32 | 2 | 0 | 53 | 84 | 60 | 91 | 2.00E-005 | 48.1 |
| candidate_681 | RF00059;TPP;ABZW01000001.1/533523-533636 | 100 | 24 | 0 | 0 | 62 | 85 | 75 | 98 | 2.00E-005 | 48.1 |
| candidate_681 | RF00059;TPP;ABVA01000001.1/801145-801244 | 90 | 40 | 4 | 0 | 43 | 82 | 58 | 97 | 2.00E-005 | 48.1 |
| candidate_681 | RF00059;TPP;ABMV01102734.1/1-85 | 93.55 | 31 | 2 | 0 | 54 | 84 | 55 | 85 | 7.00E-005 | 46.1 |
| candidate_681 | RF00059;TPP;AAFX01125019.1/603-670 | 100 | 23 | 0 | 0 | 56 | 78 | 40 | 62 | 7.00E-005 | 46.1 |
| candidate_681 | RF00059;TPP;AM406670.1/904016-904117 | 93.55 | 31 | 2 | 0 | 54 | 84 | 55 | 85 | 7.00E-005 | 46.1 |
| candidate_681 | RF00059;TPP;ABCR01000001.1/235576-235709 | 100 | 23 | 0 | 0 | 56 | 78 | 89 | 111 | 7.00E-005 | 46.1 |
| candidate_681 | RF00059;TPP;CP000679.1/255822-255947 | 93.55 | 31 | 2 | 0 | 56 | 86 | 81 | 111 | 7.00E-005 | 46.1 |
| candidate_681 | RF00059;TPP;AAWV02000002.1/347861-347972 | 100 | 23 | 0 | 0 | 56 | 78 | 67 | 89 | 7.00E-005 | 46.1 |
| candidate_681 | RF00059;TPP;CT971583.1/206168-206279 | 100 | 23 | 0 | 0 | 56 | 78 | 66 | 88 | 7.00E-005 | 46.1 |
| candidate_681 | RF00059;TPP;ACEV01000086.1/11073-11184 | 96.3 | 27 | 1 | 0 | 60 | 86 | 71 | 97 | 7.00E-005 | 46.1 |
| candidate_681 | RF00059;TPP;ACEU01000123.1/6943-7053 | 93.55 | 31 | 2 | 0 | 56 | 86 | 66 | 96 | 7.00E-005 | 46.1 |
| candidate_681 | RF00059;TPP;ABYB01000080.1/970-1083 | 93.55 | 31 | 2 | 0 | 56 | 86 | 68 | 98 | 7.00E-005 | 46.1 |
| candidate_681 | RF00059;TPP;AM420293.1/1093457-1093545 | 93.55 | 31 | 2 | 0 | 56 | 86 | 58 | 88 | 7.00E-005 | 46.1 |
| candidate_681 | RF00059;TPP;CP000481.1/1089799-1089911 | 93.55 | 31 | 2 | 0 | 56 | 86 | 67 | 97 | 7.00E-005 | 46.1 |
| candidate_681 | RF00059;TPP;AP008957.1/1615848-1615994 | 93.55 | 31 | 2 | 0 | 48 | 78 | 94 | 124 | 7.00E-005 | 46.1 |
| candidate_681 | RF00059;TPP;CP000431.1/2301323-2301435 | 93.55 | 31 | 2 | 0 | 48 | 78 | 60 | 90 | 7.00E-005 | 46.1 |
| candidate_681 | RF00059;TPP;AACY023844374.1/1530-1607 | 93.33 | 30 | 2 | 0 | 56 | 85 | 46 | 75 | 3.00E-004 | 44.1 |
| candidate_681 | RF00059;TPP;AAFX01036045.1/1-108 | 93.33 | 30 | 2 | 0 | 56 | 85 | 63 | 92 | 3.00E-004 | 44.1 |
| candidate_681 | RF00059;TPP;AAFX01035049.1/856-958 | 93.33 | 30 | 2 | 0 | 56 | 85 | 57 | 86 | 3.00E-004 | 44.1 |
| candidate_681 | RF00059;TPP;ABHC01000014.1/42663-42760 | 93.33 | 30 | 2 | 0 | 56 | 85 | 53 | 82 | 3.00E-004 | 44.1 |
| candidate_681 | RF00059;TPP;ABHC01000002.1/261118-261228 | 93.33 | 30 | 2 | 0 | 56 | 85 | 65 | 94 | 3.00E-004 | 44.1 |
| candidate_681 | RF00059;TPP;AAUA01000001.1/1364485-1364592 | 93.33 | 30 | 2 | 0 | 55 | 84 | 62 | 91 | 3.00E-004 | 44.1 |
| candidate_681 | RF00059;TPP;ABCM01000004.1/355196-355337 | 93.33 | 30 | 2 | 0 | 55 | 84 | 96 | 125 | 3.00E-004 | 44.1 |
| candidate_681 | RF00059;TPP;AJ561198.1/81601-81713 | 93.33 | 30 | 2 | 0 | 56 | 85 | 68 | 97 | 3.00E-004 | 44.1 |
| candidate_681 | RF00059;TPP;CP000088.1/1223540-1223649 | 93.33 | 30 | 2 | 0 | 56 | 85 | 65 | 94 | 3.00E-004 | 44.1 |
| candidate_681 | RF00059;TPP;ACEV01000087.1/566658-566765 | 93.33 | 30 | 2 | 0 | 56 | 85 | 63 | 92 | 3.00E-004 | 44.1 |
| candidate_681 | RF00059;TPP;ACEX01000411.1/30142-30313 | 93.33 | 30 | 2 | 0 | 56 | 85 | 127 | 156 | 3.00E-004 | 44.1 |
| candidate_681 | RF00059;TPP;AM420293.1/559560-559669 | 93.33 | 30 | 2 | 0 | 56 | 85 | 65 | 94 | 3.00E-004 | 44.1 |
| candidate_681 | RF00059;TPP;ABTA01000007.1/126035-126146 | 93.33 | 30 | 2 | 0 | 56 | 85 | 67 | 96 | 3.00E-004 | 44.1 |
| candidate_681 | RF00059;TPP;ABUH01000025.1/64366-64450 | 93.33 | 30 | 2 | 0 | 56 | 85 | 56 | 85 | 3.00E-004 | 44.1 |
| candidate_681 | RF00059;TPP;BAAW01000260.1/2791-2920 | 93.1 | 29 | 2 | 0 | 56 | 84 | 85 | 113 | 0 | 42.1 |
| candidate_681 | RF00059;TPP;BAAW01010333.1/375-537 | 93.1 | 29 | 2 | 0 | 56 | 84 | 117 | 145 | 0 | 42.1 |
| candidate_681 | RF00059;TPP;BABA01007393.1/1019-1134 | 93.1 | 29 | 2 | 0 | 56 | 84 | 70 | 98 | 0 | 42.1 |
| candidate_681 | RF00059;TPP;AACY020556771.1/1618-1726 | 93.1 | 29 | 2 | 0 | 56 | 84 | 64 | 92 | 0 | 42.1 |
| candidate_681 | RF00059;TPP;AACY022601453.1/232-332 | 93.1 | 29 | 2 | 0 | 56 | 84 | 55 | 83 | 0 | 42.1 |
| candidate_681 | RF00059;TPP;AACY020535509.1/2057-2154 | 93.1 | 29 | 2 | 0 | 56 | 84 | 53 | 81 | 0 | 42.1 |
| candidate_681 | RF00059;TPP;AACY020367462.1/3697-3796 | 93.1 | 29 | 2 | 0 | 56 | 84 | 55 | 83 | 0 | 42.1 |
| candidate_681 | RF00059;TPP;AACY023858922.1/681-781 | 93.1 | 29 | 2 | 0 | 56 | 84 | 55 | 83 | 0 | 42.1 |
| candidate_681 | RF00059;TPP;AACY020152799.1/970-1070 | 93.1 | 29 | 2 | 0 | 56 | 84 | 55 | 83 | 0 | 42.1 |
| candidate_681 | RF00059;TPP;AACY022263442.1/362-461 | 93.1 | 29 | 2 | 0 | 56 | 84 | 54 | 82 | 0 | 42.1 |
| candidate_681 | RF00059;TPP;AACY022576335.1/424-537 | 93.1 | 29 | 2 | 0 | 56 | 84 | 69 | 97 | 0 | 42.1 |
| candidate_681 | RF00059;TPP;ABON01003074.1/11-120 | 93.1 | 29 | 2 | 0 | 56 | 84 | 63 | 91 | 0 | 42.1 |
| candidate_681 | RF00059;TPP;AASZ01001815.1/3595-3692 | 93.1 | 29 | 2 | 0 | 56 | 84 | 53 | 81 | 0 | 42.1 |
| candidate_681 | RF00059;TPP;AAFZ01028830.1/464-561 | 93.1 | 29 | 2 | 0 | 56 | 84 | 53 | 81 | 0 | 42.1 |
| candidate_681 | RF00059;TPP;AASZ01000538.1/31436-31543 | 93.1 | 29 | 2 | 0 | 56 | 84 | 63 | 91 | 0 | 42.1 |
| candidate_681 | RF00059;TPP;AAFY01023680.1/441-538 | 93.1 | 29 | 2 | 0 | 56 | 84 | 53 | 81 | 0 | 42.1 |
| candidate_681 | RF00059;TPP;CP000975.1/59171-59283 | 93.1 | 29 | 2 | 0 | 56 | 84 | 68 | 96 | 0 | 42.1 |
| candidate_681 | RF00059;TPP;ACJX01000177.1/15723-15833 | 93.1 | 29 | 2 | 0 | 56 | 84 | 65 | 93 | 0 | 42.1 |
| candidate_681 | RF00059;TPP;CP000647.1/79059-79278 | 93.1 | 29 | 2 | 0 | 56 | 84 | 174 | 202 | 0 | 42.1 |
| candidate_681 | RF00059;TPP;ABKX01000006.1/141255-141355 | 93.1 | 29 | 2 | 0 | 56 | 84 | 55 | 83 | 0 | 42.1 |
| candidate_681 | RF00059;TPP;AAJU02000044.1/13148-13247 | 93.1 | 29 | 2 | 0 | 56 | 84 | 54 | 82 | 0 | 42.1 |
| candidate_681 | RF00059;TPP;ABWM01000011.1/155370-155470 | 93.1 | 29 | 2 | 0 | 56 | 84 | 55 | 83 | 0 | 42.1 |
| candidate_681 | RF00059;TPP;CP000783.1/3198294-3198395 | 93.1 | 29 | 2 | 0 | 56 | 84 | 56 | 84 | 0 | 42.1 |
| candidate_681 | RF00059;TPP;CP000822.1/3078977-3079098 | 93.1 | 29 | 2 | 0 | 56 | 84 | 76 | 104 | 0 | 42.1 |
| candidate_681 | RF00059;TPP;AAXY01000001.1/116185-116293 | 93.1 | 29 | 2 | 0 | 56 | 84 | 64 | 92 | 0 | 42.1 |
| candidate_681 | RF00059;TPP;ABST01000043.1/3103-3215 | 93.1 | 29 | 2 | 0 | 56 | 84 | 67 | 95 | 0 | 42.1 |
| candidate_681 | RF00059;TPP;CP001219.1/864231-864324 | 93.1 | 29 | 2 | 0 | 56 | 84 | 49 | 77 | 0 | 42.1 |
| candidate_681 | RF00059;TPP;CP000148.1/3319593-3319698 | 93.1 | 29 | 2 | 0 | 56 | 84 | 61 | 89 | 0 | 42.1 |
| candidate_681 | RF00059;TPP;AP007255.1/253983-254095 | 93.1 | 29 | 2 | 0 | 56 | 84 | 68 | 96 | 0 | 42.1 |
| candidate_681 | RF00059;TPP;ABXL01000049.1/160359-160466 | 93.1 | 29 | 2 | 0 | 56 | 84 | 63 | 91 | 0 | 42.1 |
| candidate_681 | RF00059;TPP;CP000489.1/34800-34903 | 93.1 | 29 | 2 | 0 | 56 | 84 | 59 | 87 | 0 | 42.1 |
| candidate_681 | RF00059;TPP;CP000781.1/3914689-3914822 | 93.1 | 29 | 2 | 0 | 56 | 84 | 88 | 116 | 0 | 42.1 |
| candidate_681 | RF00059;TPP;CP001393.1/421807-421939 | 93.1 | 29 | 2 | 0 | 56 | 84 | 90 | 118 | 0 | 42.1 |
| candidate_681 | RF00059;TPP;AP008226.1/1687829-1687922 | 93.1 | 29 | 2 | 0 | 56 | 84 | 48 | 76 | 0 | 42.1 |
| candidate_681 | RF00059;TPP;ABTN01000014.1/36536-36633 | 93.1 | 29 | 2 | 0 | 56 | 84 | 53 | 81 | 0 | 42.1 |
| candidate_681 | RF00059;TPP;AANO01000004.1/218735-218862 | 93.1 | 29 | 2 | 0 | 56 | 84 | 83 | 111 | 0 | 42.1 |
| candidate_681 | RF00059;TPP;BX569689.1/134905-135013 | 93.1 | 29 | 2 | 0 | 56 | 84 | 64 | 92 | 0 | 42.1 |
| candidate_681 | RF00059;TPP;ABSE01000016.1/67537-67654 | 93.1 | 29 | 2 | 0 | 56 | 84 | 73 | 101 | 0 | 42.1 |
| candidate_681 | RF00059;TPP;ACHB01000090.1/32343-32402 | 93.1 | 29 | 2 | 0 | 56 | 84 | 15 | 43 | 0 | 42.1 |
| candidate_681 | RF00059;TPP;ACHA01000112.1/46763-46850 | 93.1 | 29 | 2 | 0 | 56 | 84 | 43 | 71 | 0 | 42.1 |
| candidate_681 | RF00059;TPP;AAXU01000002.1/192275-192371 | 93.1 | 29 | 2 | 0 | 56 | 84 | 52 | 80 | 0 | 42.1 |
| candidate_681 | RF00059;TPP;ABZY01000050.1/14436-14532 | 93.1 | 29 | 2 | 0 | 56 | 84 | 52 | 80 | 0 | 42.1 |
| candidate_681 | RF00059;TPP;ABTA01000008.1/13667-13783 | 90.91 | 33 | 3 | 0 | 53 | 85 | 67 | 99 | 0 | 42.1 |
| candidate_681 | RF00059;TPP;CP000910.1/1024259-1024382 | 93.1 | 29 | 2 | 0 | 56 | 84 | 78 | 106 | 0 | 42.1 |
| candidate_681 | RF00059;TPP;CP001620.1/2002546-2002654 | 100 | 21 | 0 | 0 | 62 | 82 | 70 | 90 | 0 | 42.1 |
| candidate_681 | RF00059;TPP;CP001601.1/413204-413383 | 96 | 25 | 1 | 0 | 56 | 80 | 132 | 156 | 0 | 42.1 |
| candidate_681 | RF00059;TPP;AAFZ01019953.1/69-180 | 95.83 | 24 | 1 | 0 | 56 | 79 | 66 | 89 | 0 | 40.1 |
| candidate_681 | RF00059;TPP;AE017283.1/962710-962823 | 95.83 | 24 | 1 | 0 | 62 | 85 | 75 | 98 | 0 | 40.1 |
| candidate_681 | RF00059;TPP;ABTV01000008.1/20705-20800 | 90.62 | 32 | 3 | 0 | 56 | 87 | 55 | 86 | 0 | 40.1 |
| candidate_681 | RF00059;TPP;BX927151.1/81491-81600 | 95.83 | 24 | 1 | 0 | 56 | 79 | 64 | 87 | 0 | 40.1 |
| candidate_753 | RF00380;ykoK;AASN01000027.1/80343-80511 | 100 | 33 | 0 | 0 | 19 | 51 | 1 | 33 | 4.00E-011 | 65.9 |
| candidate_753 | RF00380;ykoK;ACBV01000002.1/67696-67865 | 100 | 33 | 0 | 0 | 19 | 51 | 1 | 33 | 4.00E-011 | 65.9 |
| candidate_754 | RF00380;ykoK;AASN01000027.1/80343-80511 | 98.51 | 67 | 1 | 0 | 1 | 67 | 70 | 136 | 7.00E-029 | 125 |
| candidate_754 | RF00380;ykoK;ACBV01000002.1/67696-67865 | 97.01 | 67 | 2 | 0 | 1 | 67 | 70 | 136 | 2.00E-026 | 117 |
| candidate_754 | RF00380;ykoK;CP000854.1/3273427-3273602 | 98.15 | 54 | 1 | 0 | 1 | 54 | 70 | 123 | 4.00E-021 | 99.6 |
| candidate_754 | RF00380;ykoK;CP000854.1/361669-361839 | 96.08 | 51 | 2 | 0 | 1 | 51 | 70 | 120 | 6.00E-017 | 85.7 |
| candidate_754 | RF00380;ykoK;ABIN01000121.1/2753-2927 | 94.34 | 53 | 3 | 0 | 1 | 53 | 70 | 122 | 9.00E-016 | 81.8 |
| candidate_754 | RF00380;ykoK;AE016822.1/2178596-2178766 | 89.13 | 46 | 5 | 0 | 4 | 49 | 74 | 119 | 8.00E-007 | 52.0 |
| candidate_754 | RF00380;ykoK;CU458896.1/4831017-4831187 | 87.76 | 49 | 6 | 0 | 1 | 49 | 71 | 119 | 3.00E-006 | 50.1 |
| candidate_754 | RF00380;ykoK;ABUA01000006.1/49399-49573 | 90.91 | 33 | 3 | 0 | 17 | 49 | 87 | 119 | 8.00E-004 | 42.1 |
| candidate_754 | RF00380;ykoK;ABVA01000005.1/109821-109991 | 85.71 | 49 | 7 | 0 | 1 | 49 | 71 | 119 | 8.00E-004 | 42.1 |
| candidate_754 | RF00380;ykoK;ACNO01000010.1/64597-64770 | 86.36 | 44 | 6 | 0 | 6 | 49 | 76 | 119 | 0 | 40.1 |
| candidate_755 | RF00230;T-box;CP000448.1/2470265-2470519 | 95.83 | 24 | 1 | 0 | 107 | 130 | 210 | 233 | 0.01 | 40.1 |
| candidate_755 | RF00230;T-box;ABUS01000002.1/276730-276890 | 90.62 | 32 | 3 | 0 | 52 | 83 | 61 | 92 | 0.01 | 40.1 |
| candidate_766 | RF00380;ykoK;CP000854.1/3273427-3273602 | 95.65 | 92 | 4 | 0 | 3 | 94 | 1 | 92 | 2.00E-036 | 151 |
| candidate_766 | RF00380;ykoK;ABIN01000121.1/2753-2927 | 95.6 | 91 | 4 | 0 | 3 | 93 | 1 | 91 | 7.00E-036 | 149 |
| candidate_766 | RF00380;ykoK;CP000854.1/361669-361839 | 91.3 | 92 | 8 | 0 | 3 | 94 | 1 | 92 | 6.00E-027 | 119 |
| candidate_766 | RF00380;ykoK;AASN01000027.1/80343-80511 | 90.11 | 91 | 9 | 0 | 4 | 94 | 2 | 92 | 6.00E-024 | 109 |
| candidate_766 | RF00380;ykoK;ACBV01000002.1/67696-67865 | 89.01 | 91 | 10 | 0 | 4 | 94 | 2 | 92 | 1.00E-021 | 101 |
| candidate_766 | RF00380;ykoK;ABVA01000005.1/109821-109991 | 85.23 | 88 | 10 | 2 | 8 | 93 | 6 | 92 | 2.00E-008 | 58.0 |
| candidate_766 | RF00380;ykoK;AL583922.1/170554-170727 | 89.8 | 49 | 5 | 0 | 9 | 57 | 7 | 55 | 2.00E-008 | 58.0 |
| candidate_766 | RF00380;ykoK;CP000480.1/5735476-5735649 | 92.5 | 40 | 3 | 0 | 55 | 94 | 54 | 93 | 8.00E-008 | 56.0 |
| candidate_766 | RF00380;ykoK;FM211192.1/3196861-3197030 | 90.7 | 43 | 4 | 0 | 3 | 45 | 1 | 43 | 3.00E-007 | 54.0 |
| candidate_766 | RF00380;ykoK;ABVA01000002.1/216092-216271 | 87.04 | 54 | 7 | 0 | 30 | 83 | 30 | 83 | 1.00E-006 | 52.0 |
| candidate_766 | RF00380;ykoK;CU458896.1/4831017-4831187 | 83.7 | 92 | 12 | 2 | 5 | 94 | 3 | 93 | 5.00E-006 | 50.1 |
| candidate_766 | RF00380;ykoK;CU458896.1/2766928-2767096 | 83.52 | 91 | 12 | 2 | 5 | 93 | 3 | 92 | 2.00E-005 | 48.1 |
| candidate_766 | RF00380;ykoK;AAGD02008568.1/493-663 | 87.5 | 40 | 5 | 0 | 55 | 94 | 54 | 93 | 0.01 | 40.1 |
| candidate_852 | RF00634;SAM-IV;CP000325.1/1553311-1553430 | 95 | 120 | 6 | 0 | 2 | 121 | 1 | 120 | 3.00E-048 | 190 |
| candidate_852 | RF00634;SAM-IV;CP000480.1/1748264-1748393 | 98.86 | 88 | 1 | 0 | 2 | 89 | 1 | 88 | 5.00E-041 | 167 |
| candidate_852 | RF00634;SAM-IV;U00022.1/34579-34697 | 93.1 | 116 | 7 | 1 | 2 | 117 | 1 | 115 | 1.00E-038 | 159 |
| candidate_852 | RF00634;SAM-IV;CU458896.1/3741892-3742006 | 95.65 | 92 | 4 | 0 | 2 | 93 | 1 | 92 | 3.00E-036 | 151 |
| candidate_852 | RF00634;SAM-IV;AM420293.1/6539066-6539181 | 98.73 | 79 | 1 | 0 | 2 | 80 | 1 | 79 | 1.00E-035 | 149 |
| candidate_852 | RF00634;SAM-IV;AP008957.1/2193162-2193300 | 96.43 | 84 | 3 | 0 | 2 | 85 | 1 | 84 | 7.00E-034 | 143 |
| candidate_852 | RF00634;SAM-IV;CP000511.1/1643626-1643745 | 94.57 | 92 | 5 | 0 | 2 | 93 | 1 | 92 | 7.00E-034 | 143 |
| candidate_852 | RF00634;SAM-IV;AAMN01000002.1/91115-91229 | 95.12 | 82 | 4 | 0 | 2 | 83 | 1 | 82 | 2.00E-030 | 131 |
| candidate_852 | RF00634;SAM-IV;AP006618.1/1024437-1024547 | 94.94 | 79 | 4 | 0 | 2 | 80 | 1 | 79 | 2.00E-028 | 125 |
| candidate_852 | RF00634;SAM-IV;ACEV01000063.1/16996-17110 | 93.9 | 82 | 5 | 0 | 2 | 83 | 1 | 82 | 6.00E-028 | 123 |
| candidate_852 | RF00634;SAM-IV;CP001341.1/1524978-1525093 | 92.94 | 85 | 6 | 0 | 2 | 86 | 1 | 85 | 2.00E-027 | 121 |
| candidate_852 | RF00634;SAM-IV;AACY020524451.1/3655-3761 | 92.68 | 82 | 6 | 0 | 2 | 83 | 1 | 82 | 1.00E-025 | 115 |
| candidate_852 | RF00634;SAM-IV;ABVC01000005.1/190421-190536 | 92.68 | 82 | 6 | 0 | 2 | 83 | 1 | 82 | 1.00E-025 | 115 |
| candidate_852 | RF00634;SAM-IV;CP000820.1/5910498-5910611 | 95.12 | 82 | 2 | 2 | 2 | 83 | 1 | 80 | 1.00E-025 | 115 |
| candidate_852 | RF00634;SAM-IV;ABTV01000002.1/25962-26079 | 91.76 | 85 | 7 | 0 | 2 | 86 | 1 | 85 | 6.00E-025 | 113 |
| candidate_852 | RF00634;SAM-IV;ABUI01000015.1/132940-133054 | 94.44 | 72 | 4 | 0 | 13 | 84 | 12 | 83 | 2.00E-024 | 111 |
| candidate_852 | RF00634;SAM-IV;ABVA01000004.1/99894-100014 | 92.86 | 84 | 5 | 1 | 2 | 85 | 1 | 83 | 2.00E-024 | 111 |
| candidate_852 | RF00634;SAM-IV;ABUH01000002.1/213285-213399 | 98.31 | 59 | 1 | 0 | 22 | 80 | 21 | 79 | 9.00E-024 | 109 |
| candidate_852 | RF00634;SAM-IV;ABUH01000009.1/76831-76946 | 93.24 | 74 | 5 | 0 | 2 | 75 | 1 | 74 | 4.00E-023 | 107 |
| candidate_852 | RF00634;SAM-IV;CP000431.1/6724302-6724422 | 91.67 | 84 | 6 | 1 | 2 | 85 | 1 | 83 | 6.00E-022 | 103 |
| candidate_852 | RF00634;SAM-IV;CP000454.1/1477703-1477817 | 91.14 | 79 | 7 | 0 | 2 | 80 | 1 | 79 | 2.00E-021 | 101 |
| candidate_852 | RF00634;SAM-IV;CP000750.2/1351757-1351870 | 92.41 | 79 | 5 | 1 | 2 | 80 | 1 | 78 | 2.00E-021 | 101 |
| candidate_852 | RF00634;SAM-IV;AACY023819247.1/1-103 | 98.15 | 54 | 1 | 0 | 13 | 66 | 12 | 65 | 9.00E-021 | 99.6 |
| candidate_852 | RF00634;SAM-IV;ABUC01000021.1/23885-23998 | 91.46 | 82 | 6 | 1 | 2 | 83 | 1 | 81 | 9.00E-021 | 99.6 |
| candidate_852 | RF00634;SAM-IV;ABUA01000011.1/21354-21471 | 90.24 | 82 | 8 | 0 | 2 | 83 | 1 | 82 | 9.00E-021 | 99.6 |
| candidate_852 | RF00634;SAM-IV;ACES01000171.1/19283-19402 | 91.57 | 83 | 3 | 2 | 2 | 80 | 1 | 83 | 3.00E-020 | 97.6 |
| candidate_852 | RF00634;SAM-IV;CP000454.1/2980407-2980520 | 89.02 | 82 | 9 | 0 | 2 | 83 | 1 | 82 | 2.00E-018 | 91.7 |
| candidate_852 | RF00634;SAM-IV;ABLL01000167.1/263401-263515 | 100 | 45 | 0 | 0 | 2 | 46 | 1 | 45 | 8.00E-018 | 89.7 |
| candidate_852 | RF00634;SAM-IV;ABLL01000167.1/263401-263515 | 100 | 23 | 0 | 0 | 99 | 121 | 93 | 115 | 1.00E-004 | 46.1 |
| candidate_852 | RF00634;SAM-IV;ABTJ01000057.1/3503-3617 | 91.25 | 80 | 5 | 2 | 2 | 80 | 1 | 79 | 3.00E-017 | 87.7 |
| candidate_852 | RF00634;SAM-IV;CP000750.2/1117347-1117457 | 89.87 | 79 | 6 | 1 | 2 | 80 | 1 | 77 | 3.00E-017 | 87.7 |
| candidate_852 | RF00634;SAM-IV;CP000431.1/4627893-4628007 | 90.79 | 76 | 6 | 1 | 13 | 88 | 12 | 86 | 3.00E-017 | 87.7 |
| candidate_852 | RF00634;SAM-IV;BABF01015715.1/309-421 | 89.87 | 79 | 7 | 1 | 2 | 80 | 1 | 78 | 1.00E-016 | 85.7 |
| candidate_852 | RF00634;SAM-IV;AACY020299785.1/1743-1846 | 92.54 | 67 | 4 | 1 | 14 | 79 | 13 | 79 | 1.00E-016 | 85.7 |
| candidate_852 | RF00634;SAM-IV;ABTA01000006.1/22003-22116 | 89.16 | 83 | 8 | 1 | 2 | 84 | 1 | 82 | 1.00E-016 | 85.7 |
| candidate_852 | RF00634;SAM-IV;CP000850.1/357435-357554 | 89.29 | 84 | 4 | 2 | 2 | 80 | 1 | 84 | 1.00E-016 | 85.7 |
| candidate_852 | RF00634;SAM-IV;CP000910.1/1175203-1175316 | 88.61 | 79 | 9 | 0 | 2 | 80 | 1 | 79 | 1.00E-016 | 85.7 |
| candidate_852 | RF00634;SAM-IV;AP009152.1/1881654-1881772 | 91.04 | 67 | 6 | 0 | 14 | 80 | 13 | 79 | 1.00E-016 | 85.7 |
| candidate_852 | RF00634;SAM-IV;CP001341.1/1526602-1526716 | 93.22 | 59 | 4 | 0 | 22 | 80 | 20 | 78 | 1.00E-016 | 85.7 |
| candidate_852 | RF00634;SAM-IV;CP000474.1/1618337-1618453 | 94.92 | 59 | 2 | 1 | 22 | 80 | 21 | 78 | 1.00E-016 | 85.7 |
| candidate_852 | RF00634;SAM-IV;AAMN01000002.1/104082-104193 | 90.54 | 74 | 5 | 1 | 2 | 75 | 1 | 72 | 1.00E-016 | 85.7 |
| candidate_852 | RF00634;SAM-IV;ABVA01000002.1/599201-599312 | 89.87 | 79 | 7 | 1 | 2 | 80 | 1 | 78 | 1.00E-016 | 85.7 |
| candidate_852 | RF00634;SAM-IV;AAAP01003574.1/1814-1929 | 87.8 | 82 | 10 | 0 | 2 | 83 | 1 | 82 | 5.00E-016 | 83.8 |
| candidate_852 | RF00634;SAM-IV;ACEW01000235.1/7286-7402 | 91.94 | 62 | 5 | 0 | 23 | 84 | 23 | 84 | 5.00E-016 | 83.8 |
| candidate_852 | RF00634;SAM-IV;ABLQ01000005.1/68080-68195 | 91.8 | 61 | 5 | 0 | 23 | 83 | 22 | 82 | 2.00E-015 | 81.8 |
| candidate_852 | RF00634;SAM-IV;AACY020733997.1/91-203 | 90.28 | 72 | 6 | 1 | 13 | 84 | 12 | 82 | 8.00E-015 | 79.8 |
| candidate_852 | RF00634;SAM-IV;AM746676.1/1674959-1675073 | 88.75 | 80 | 8 | 1 | 2 | 80 | 1 | 80 | 8.00E-015 | 79.8 |
| candidate_852 | RF00634;SAM-IV;ABUU01000008.1/48812-48927 | 88.75 | 80 | 8 | 1 | 2 | 80 | 1 | 80 | 8.00E-015 | 79.8 |
| candidate_852 | RF00634;SAM-IV;AACY021939217.1/127-229 | 94.12 | 51 | 3 | 0 | 21 | 71 | 20 | 70 | 3.00E-014 | 77.8 |
| candidate_852 | RF00634;SAM-IV;AACY023319371.1/240-353 | 92.06 | 63 | 4 | 1 | 22 | 84 | 21 | 82 | 3.00E-014 | 77.8 |
| candidate_852 | RF00634;SAM-IV;AP006618.1/396893-397006 | 88.61 | 79 | 8 | 1 | 2 | 80 | 1 | 78 | 3.00E-014 | 77.8 |
| candidate_852 | RF00634;SAM-IV;AP006618.1/5665528-5665640 | 88.61 | 79 | 8 | 1 | 2 | 80 | 1 | 78 | 3.00E-014 | 77.8 |
| candidate_852 | RF00634;SAM-IV;ABTV01000005.1/152086-152199 | 88.61 | 79 | 8 | 1 | 2 | 80 | 1 | 78 | 3.00E-014 | 77.8 |
| candidate_852 | RF00634;SAM-IV;CP000910.1/1370717-1370844 | 93.1 | 58 | 3 | 1 | 23 | 80 | 37 | 93 | 1.00E-013 | 75.8 |
| candidate_852 | RF00634;SAM-IV;AACY020172363.1/59-171 | 90.77 | 65 | 5 | 1 | 21 | 85 | 21 | 84 | 5.00E-013 | 73.8 |
| candidate_852 | RF00634;SAM-IV;CT573213.2/4831346-4831458 | 89.04 | 73 | 7 | 1 | 3 | 75 | 2 | 73 | 5.00E-013 | 73.8 |
| candidate_852 | RF00634;SAM-IV;AACY020857079.1/737-849 | 90.62 | 64 | 5 | 1 | 21 | 84 | 19 | 81 | 2.00E-012 | 71.9 |
| candidate_852 | RF00634;SAM-IV;AACY020485607.1/210-315 | 90.48 | 63 | 5 | 1 | 22 | 84 | 21 | 82 | 8.00E-012 | 69.9 |
| candidate_852 | RF00634;SAM-IV;AACY020470541.1/4478-4591 | 90.48 | 63 | 5 | 1 | 22 | 84 | 21 | 82 | 8.00E-012 | 69.9 |
| candidate_852 | RF00634;SAM-IV;AACY023320770.1/896-1008 | 88.73 | 71 | 7 | 1 | 13 | 83 | 12 | 81 | 8.00E-012 | 69.9 |
| candidate_852 | RF00634;SAM-IV;ABJJ01000202.1/1763-1872 | 86.08 | 79 | 11 | 0 | 2 | 80 | 1 | 79 | 8.00E-012 | 69.9 |
| candidate_852 | RF00634;SAM-IV;AAMN01000002.1/4575-4689 | 91.53 | 59 | 4 | 1 | 22 | 80 | 21 | 78 | 8.00E-012 | 69.9 |
| candidate_852 | RF00634;SAM-IV;ABTU01000022.1/12836-12948 | 87.34 | 79 | 9 | 1 | 2 | 80 | 1 | 78 | 8.00E-012 | 69.9 |
| candidate_852 | RF00634;SAM-IV;ACNO01000010.1/27883-27997 | 91.53 | 59 | 4 | 1 | 22 | 80 | 21 | 78 | 8.00E-012 | 69.9 |
| candidate_852 | RF00634;SAM-IV;AACY022337359.1/42-155 | 90.32 | 62 | 5 | 1 | 21 | 82 | 20 | 80 | 3.00E-011 | 67.9 |
| candidate_852 | RF00634;SAM-IV;CT573213.2/4609200-4609314 | 86.3 | 73 | 10 | 0 | 7 | 79 | 6 | 78 | 1.00E-010 | 65.9 |
| candidate_852 | RF00634;SAM-IV;AACY020059440.1/385-514 | 90.62 | 64 | 4 | 2 | 21 | 83 | 40 | 102 | 5.00E-010 | 63.9 |
| candidate_852 | RF00634;SAM-IV;AACY023429840.1/1546-1651 | 90 | 60 | 5 | 1 | 21 | 80 | 20 | 78 | 5.00E-010 | 63.9 |
| candidate_852 | RF00634;SAM-IV;AACY020767873.1/507-620 | 89.06 | 64 | 6 | 1 | 21 | 84 | 20 | 82 | 5.00E-010 | 63.9 |
| candidate_852 | RF00634;SAM-IV;AACY020168337.1/2556-2688 | 86.57 | 67 | 9 | 0 | 21 | 87 | 20 | 86 | 2.00E-009 | 61.9 |
| candidate_852 | RF00634;SAM-IV;AACY020174818.1/746-859 | 88.89 | 63 | 6 | 1 | 22 | 84 | 21 | 82 | 2.00E-009 | 61.9 |
| candidate_852 | RF00634;SAM-IV;CP000750.2/3280049-3280159 | 86.08 | 79 | 10 | 1 | 2 | 80 | 1 | 78 | 2.00E-009 | 61.9 |
| candidate_852 | RF00634;SAM-IV;CU458896.1/513238-513350 | 86.08 | 79 | 10 | 1 | 2 | 80 | 1 | 78 | 2.00E-009 | 61.9 |
| candidate_852 | RF00634;SAM-IV;ACEU01000123.1/41999-42116 | 87.93 | 58 | 7 | 0 | 23 | 80 | 24 | 81 | 8.00E-009 | 60.0 |
| candidate_852 | RF00634;SAM-IV;ABJJ01000068.1/34841-34954 | 88.71 | 62 | 6 | 1 | 14 | 75 | 13 | 73 | 8.00E-009 | 60.0 |
| candidate_852 | RF00634;SAM-IV;AACY023345252.1/107-222 | 93.33 | 45 | 2 | 1 | 39 | 83 | 38 | 81 | 3.00E-008 | 58.0 |
| candidate_852 | RF00634;SAM-IV;AACY023288661.1/869-983 | 88.52 | 61 | 6 | 1 | 22 | 82 | 21 | 80 | 3.00E-008 | 58.0 |
| candidate_852 | RF00634;SAM-IV;ABUU01000141.1/4790-4902 | 89.8 | 49 | 5 | 0 | 21 | 69 | 20 | 68 | 3.00E-008 | 58.0 |
| candidate_852 | RF00634;SAM-IV;AACY020471271.1/2597-2710 | 87.5 | 64 | 7 | 1 | 19 | 82 | 18 | 80 | 1.00E-007 | 56.0 |
| candidate_852 | RF00634;SAM-IV;AACY023299278.1/1484-1598 | 87.5 | 64 | 7 | 1 | 22 | 85 | 21 | 83 | 1.00E-007 | 56.0 |
| candidate_852 | RF00634;SAM-IV;AACY023427498.1/645-749 | 87.3 | 63 | 7 | 1 | 22 | 84 | 21 | 82 | 5.00E-007 | 54.0 |
| candidate_852 | RF00634;SAM-IV;BA000030.3/3688669-3688777 | 96.77 | 31 | 1 | 0 | 13 | 43 | 12 | 42 | 5.00E-007 | 54.0 |
| candidate_852 | RF00634;SAM-IV;ACEY01000237.1/15548-15664 | 89.36 | 47 | 5 | 0 | 38 | 84 | 38 | 84 | 5.00E-007 | 54.0 |
| candidate_852 | RF00634;SAM-IV;ABUH01000009.1/72095-72206 | 84.81 | 79 | 11 | 1 | 2 | 80 | 1 | 78 | 5.00E-007 | 54.0 |
| candidate_852 | RF00634;SAM-IV;AACY023352981.1/678-809 | 91.3 | 46 | 3 | 1 | 21 | 66 | 41 | 85 | 2.00E-006 | 52.0 |
| candidate_852 | RF00634;SAM-IV;AACY024007064.1/392-505 | 92.86 | 42 | 2 | 1 | 39 | 80 | 38 | 78 | 2.00E-006 | 52.0 |
| candidate_852 | RF00634;SAM-IV;AACY023291574.1/1181-1294 | 91.3 | 46 | 3 | 1 | 39 | 84 | 38 | 82 | 2.00E-006 | 52.0 |
| candidate_852 | RF00634;SAM-IV;AACY020053614.1/406-510 | 88.89 | 54 | 5 | 1 | 22 | 75 | 21 | 73 | 2.00E-006 | 52.0 |
| candidate_852 | RF00634;SAM-IV;ABUS01000007.1/234919-235036 | 83.33 | 84 | 13 | 1 | 2 | 85 | 1 | 83 | 3.00E-005 | 48.1 |
| candidate_852 | RF00634;SAM-IV;AACY023329096.1/1119-1223 | 85.71 | 63 | 8 | 1 | 22 | 84 | 21 | 82 | 1.00E-004 | 46.1 |
| candidate_852 | RF00634;SAM-IV;ACEZ01000090.1/123461-123577 | 88.37 | 43 | 5 | 0 | 38 | 80 | 38 | 80 | 1.00E-004 | 46.1 |
| candidate_852 | RF00634;SAM-IV;CP000509.1/3644126-3644238 | 87.27 | 55 | 6 | 1 | 21 | 75 | 20 | 73 | 1.00E-004 | 46.1 |
| candidate_852 | RF00634;SAM-IV;ABVC01000011.1/30199-30311 | 96.15 | 26 | 1 | 0 | 41 | 66 | 40 | 65 | 5.00E-004 | 44.1 |
| candidate_852 | RF00634;SAM-IV;CP000750.2/44279-44389 | 84.48 | 58 | 9 | 0 | 23 | 80 | 22 | 79 | 5.00E-004 | 44.1 |
| candidate_852 | RF00634;SAM-IV;ABVC01000005.1/164092-164203 | 83.58 | 67 | 9 | 1 | 17 | 83 | 16 | 80 | 0.01 | 40.1 |
| candidate_852 | RF00634;SAM-IV;CP000474.1/4015360-4015471 | 86.36 | 44 | 6 | 0 | 6 | 49 | 5 | 48 | 0.01 | 40.1 |
| candidate_852 | RF00634;SAM-IV;ABUH01000009.1/61168-61277 | 88.89 | 36 | 4 | 0 | 14 | 49 | 13 | 48 | 0.01 | 40.1 |
| candidate_1082 | RF00379;ydaO-yuaA;ACHP01000024.1/35645-35867 | 100 | 53 | 0 | 0 | 1 | 53 | 171 | 223 | 5.00E-023 | 105 |
| candidate_1082 | RF00379;ydaO-yuaA;ABGN01000022.1/10987-11148 | 100 | 53 | 0 | 0 | 1 | 53 | 110 | 162 | 5.00E-023 | 105 |
| candidate_1082 | RF00379;ydaO-yuaA;ABGL01000010.1/286738-286960 | 100 | 53 | 0 | 0 | 1 | 53 | 171 | 223 | 5.00E-023 | 105 |
| candidate_1082 | RF00379;ydaO-yuaA;CP000325.1/290980-291193 | 98 | 50 | 1 | 0 | 4 | 53 | 165 | 214 | 7.00E-019 | 91.7 |
| candidate_1082 | RF00379;ydaO-yuaA;ACBV01000079.1/26483-26704 | 95.83 | 48 | 2 | 0 | 5 | 52 | 174 | 221 | 3.00E-015 | 79.8 |
| candidate_1082 | RF00379;ydaO-yuaA;ABIN01000324.1/17520-17728 | 97.06 | 34 | 1 | 0 | 4 | 37 | 160 | 193 | 2.00E-009 | 60.0 |
| candidate_1082 | RF00379;ydaO-yuaA;ACFI01000032.1/188781-188993 | 97.06 | 34 | 1 | 0 | 4 | 37 | 164 | 197 | 2.00E-009 | 60.0 |
| candidate_1082 | RF00379;ydaO-yuaA;AAFX01127366.1/160-341 | 96.77 | 31 | 1 | 0 | 9 | 39 | 139 | 169 | 2.00E-007 | 54.0 |
| candidate_1082 | RF00379;ydaO-yuaA;CP000750.2/2709708-2709896 | 96.77 | 31 | 1 | 0 | 8 | 38 | 145 | 175 | 2.00E-007 | 54.0 |
| candidate_1082 | RF00379;ydaO-yuaA;ACBV01000022.1/53288-53511 | 88.24 | 51 | 6 | 0 | 1 | 51 | 172 | 222 | 2.00E-007 | 54.0 |
| candidate_1082 | RF00379;ydaO-yuaA;ACEX01000466.1/13865-14032 | 96.67 | 30 | 1 | 0 | 9 | 38 | 125 | 154 | 6.00E-007 | 52.0 |
| candidate_1082 | RF00379;ydaO-yuaA;ABJI01000007.1/2071-2260 | 94.12 | 34 | 2 | 0 | 4 | 37 | 144 | 177 | 6.00E-007 | 52.0 |
| candidate_1082 | RF00379;ydaO-yuaA;BA000030.3/4374567-4374791 | 96.67 | 30 | 1 | 0 | 9 | 38 | 182 | 211 | 6.00E-007 | 52.0 |
| candidate_1082 | RF00379;ydaO-yuaA;ABJH01000406.1/8870-9003 | 96.67 | 30 | 1 | 0 | 9 | 38 | 91 | 120 | 6.00E-007 | 52.0 |
| candidate_1082 | RF00379;ydaO-yuaA;ABYA01000336.1/5322-5536 | 96.67 | 30 | 1 | 0 | 9 | 38 | 172 | 201 | 6.00E-007 | 52.0 |
| candidate_1082 | RF00379;ydaO-yuaA;AP009493.1/5189174-5189397 | 96.67 | 30 | 1 | 0 | 9 | 38 | 181 | 210 | 6.00E-007 | 52.0 |
| candidate_1082 | RF00379;ydaO-yuaA;ABJI01000479.1/8601-8738 | 96.67 | 30 | 1 | 0 | 9 | 38 | 95 | 124 | 6.00E-007 | 52.0 |
| candidate_1082 | RF00379;ydaO-yuaA;ABYC01000317.1/7348-7580 | 96.67 | 30 | 1 | 0 | 9 | 38 | 190 | 219 | 6.00E-007 | 52.0 |
| candidate_1082 | RF00379;ydaO-yuaA;ABJF01000219.1/8197-8364 | 96.67 | 30 | 1 | 0 | 9 | 38 | 125 | 154 | 6.00E-007 | 52.0 |
| candidate_1082 | RF00379;ydaO-yuaA;ABYX01000124.1/98158-98377 | 96.67 | 30 | 1 | 0 | 9 | 38 | 177 | 206 | 6.00E-007 | 52.0 |
| candidate_1082 | RF00379;ydaO-yuaA;ABTJ01000112.1/8922-9149 | 96.67 | 30 | 1 | 0 | 8 | 37 | 185 | 214 | 6.00E-007 | 52.0 |
| candidate_1082 | RF00379;ydaO-yuaA;CP000480.1/5785361-5785569 | 96.67 | 30 | 1 | 0 | 8 | 37 | 165 | 194 | 6.00E-007 | 52.0 |
| candidate_1082 | RF00379;ydaO-yuaA;ABVC01000015.1/4553-4729 | 96.55 | 29 | 1 | 0 | 9 | 37 | 134 | 162 | 2.00E-006 | 50.1 |
| candidate_1082 | RF00379;ydaO-yuaA;AP008957.1/5078926-5079167 | 96.55 | 29 | 1 | 0 | 9 | 37 | 198 | 226 | 2.00E-006 | 50.1 |
| candidate_1082 | RF00379;ydaO-yuaA;AP011115.1/5435990-5436203 | 96.55 | 29 | 1 | 0 | 9 | 37 | 170 | 198 | 2.00E-006 | 50.1 |
| candidate_1082 | RF00379;ydaO-yuaA;FM211192.1/2554476-2554661 | 87.76 | 49 | 6 | 0 | 4 | 52 | 137 | 185 | 2.00E-006 | 50.1 |
| candidate_1082 | RF00379;ydaO-yuaA;ABYA01000076.1/1968-2163 | 96.43 | 28 | 1 | 0 | 10 | 37 | 156 | 183 | 9.00E-006 | 48.1 |
| candidate_1082 | RF00379;ydaO-yuaA;CP000580.1/5106747-5106951 | 100 | 24 | 0 | 0 | 8 | 31 | 161 | 184 | 9.00E-006 | 48.1 |
| candidate_1082 | RF00379;ydaO-yuaA;CP000511.1/5402727-5402940 | 96.43 | 28 | 1 | 0 | 4 | 31 | 166 | 193 | 9.00E-006 | 48.1 |
| candidate_1082 | RF00379;ydaO-yuaA;CP000656.1/1774549-1774764 | 96.43 | 28 | 1 | 0 | 4 | 31 | 168 | 195 | 9.00E-006 | 48.1 |
| candidate_1082 | RF00379;ydaO-yuaA;CP000568.1/1397223-1397353 | 93.55 | 31 | 2 | 0 | 8 | 38 | 88 | 118 | 4.00E-005 | 46.1 |
| candidate_1082 | RF00379;ydaO-yuaA;AL009126.3/3188193-3188341 | 91.43 | 35 | 3 | 0 | 16 | 50 | 112 | 146 | 4.00E-005 | 46.1 |
| candidate_1082 | RF00379;ydaO-yuaA;ABTU01000003.1/299712-299839 | 100 | 23 | 0 | 0 | 9 | 31 | 85 | 107 | 4.00E-005 | 46.1 |
| candidate_1082 | RF00379;ydaO-yuaA;CT573213.2/7087632-7087807 | 93.55 | 31 | 2 | 0 | 9 | 39 | 133 | 163 | 4.00E-005 | 46.1 |
| candidate_1082 | RF00379;ydaO-yuaA;CP000249.1/5067434-5067609 | 93.55 | 31 | 2 | 0 | 9 | 39 | 133 | 163 | 4.00E-005 | 46.1 |
| candidate_1082 | RF00379;ydaO-yuaA;CP000820.1/262864-263110 | 93.55 | 31 | 2 | 0 | 9 | 39 | 206 | 236 | 4.00E-005 | 46.1 |
| candidate_1082 | RF00379;ydaO-yuaA;ABTV01000007.1/58977-59189 | 96.3 | 27 | 1 | 0 | 11 | 37 | 171 | 197 | 4.00E-005 | 46.1 |
| candidate_1082 | RF00379;ydaO-yuaA;ABSN01011425.1/311-437 | 91.18 | 34 | 3 | 0 | 6 | 39 | 81 | 114 | 1.00E-004 | 44.1 |
| candidate_1082 | RF00379;ydaO-yuaA;CP001098.1/1255255-1255400 | 96.15 | 26 | 1 | 0 | 13 | 38 | 107 | 132 | 1.00E-004 | 44.1 |
| candidate_1082 | RF00379;ydaO-yuaA;CP001615.1/1227986-1228128 | 93.33 | 30 | 2 | 0 | 9 | 38 | 100 | 129 | 1.00E-004 | 44.1 |
| candidate_1082 | RF00379;ydaO-yuaA;ABJI01000377.1/5684-5873 | 91.18 | 34 | 3 | 0 | 4 | 37 | 144 | 177 | 1.00E-004 | 44.1 |
| candidate_1082 | RF00379;ydaO-yuaA;ACEZ01000120.1/58290-58456 | 93.33 | 30 | 2 | 0 | 9 | 38 | 124 | 153 | 1.00E-004 | 44.1 |
| candidate_1082 | RF00379;ydaO-yuaA;ABYA01000247.1/5523-5685 | 93.33 | 30 | 2 | 0 | 9 | 38 | 122 | 151 | 1.00E-004 | 44.1 |
| candidate_1082 | RF00379;ydaO-yuaA;BA000030.3/5911718-5911883 | 91.18 | 34 | 3 | 0 | 4 | 37 | 118 | 151 | 1.00E-004 | 44.1 |
| candidate_1082 | RF00379;ydaO-yuaA;ABJJ01000211.1/4704-4916 | 93.33 | 30 | 2 | 0 | 9 | 38 | 170 | 199 | 1.00E-004 | 44.1 |
| candidate_1082 | RF00379;ydaO-yuaA;AL939115.1/44621-44787 | 93.33 | 30 | 2 | 0 | 9 | 38 | 124 | 153 | 1.00E-004 | 44.1 |
| candidate_1082 | RF00379;ydaO-yuaA;ABYC01000459.1/1010-1223 | 91.18 | 34 | 3 | 0 | 4 | 37 | 166 | 199 | 1.00E-004 | 44.1 |
| candidate_1082 | RF00379;ydaO-yuaA;AL939120.1/174617-174820 | 93.33 | 30 | 2 | 0 | 9 | 38 | 162 | 191 | 1.00E-004 | 44.1 |
| candidate_1082 | RF00379;ydaO-yuaA;ABJG01000069.1/16832-17009 | 93.33 | 30 | 2 | 0 | 9 | 38 | 135 | 164 | 1.00E-004 | 44.1 |
| candidate_1082 | RF00379;ydaO-yuaA;ACFA01000382.1/6406-6606 | 93.33 | 30 | 2 | 0 | 9 | 38 | 160 | 189 | 1.00E-004 | 44.1 |
| candidate_1082 | RF00379;ydaO-yuaA;ACFA01000534.1/5156-5325 | 93.33 | 30 | 2 | 0 | 9 | 38 | 127 | 156 | 1.00E-004 | 44.1 |
| candidate_1082 | RF00379;ydaO-yuaA;ACEZ01000201.1/99378-99538 | 91.18 | 34 | 3 | 0 | 4 | 37 | 115 | 148 | 1.00E-004 | 44.1 |
| candidate_1082 | RF00379;ydaO-yuaA;ACEW01000296.1/19576-19748 | 93.33 | 30 | 2 | 0 | 9 | 38 | 130 | 159 | 1.00E-004 | 44.1 |
| candidate_1082 | RF00379;ydaO-yuaA;AP009493.1/1131616-1131776 | 91.18 | 34 | 3 | 0 | 4 | 37 | 113 | 146 | 1.00E-004 | 44.1 |
| candidate_1082 | RF00379;ydaO-yuaA;ABVC01000015.1/3390-3572 | 93.33 | 30 | 2 | 0 | 8 | 37 | 140 | 169 | 1.00E-004 | 44.1 |
| candidate_1082 | RF00379;ydaO-yuaA;CP000751.1/39835-40034 | 93.33 | 30 | 2 | 0 | 9 | 38 | 157 | 186 | 1.00E-004 | 44.1 |
| candidate_1082 | RF00379;ydaO-yuaA;ABUH01000044.1/5222-5422 | 93.33 | 30 | 2 | 0 | 9 | 38 | 158 | 187 | 1.00E-004 | 44.1 |
| candidate_1082 | RF00379;ydaO-yuaA;CP000820.1/2985789-2986002 | 93.33 | 30 | 2 | 0 | 9 | 38 | 171 | 200 | 1.00E-004 | 44.1 |
| candidate_1082 | RF00379;ydaO-yuaA;CP000820.1/7938173-7938379 | 93.33 | 30 | 2 | 0 | 9 | 38 | 165 | 194 | 1.00E-004 | 44.1 |
| candidate_1082 | RF00379;ydaO-yuaA;CP000249.1/3540383-3540581 | 93.33 | 30 | 2 | 0 | 9 | 38 | 157 | 186 | 1.00E-004 | 44.1 |
| candidate_1082 | RF00379;ydaO-yuaA;CT573213.2/5337532-5337721 | 93.33 | 30 | 2 | 0 | 9 | 38 | 148 | 177 | 1.00E-004 | 44.1 |
| candidate_1082 | RF00379;ydaO-yuaA;CP000820.1/6974725-6974922 | 93.33 | 30 | 2 | 0 | 9 | 38 | 156 | 185 | 1.00E-004 | 44.1 |
| candidate_1082 | RF00379;ydaO-yuaA;ACIH01000204.1/18885-19097 | 96 | 25 | 1 | 0 | 9 | 33 | 170 | 194 | 6.00E-004 | 42.1 |
| candidate_1082 | RF00379;ydaO-yuaA;ACEZ01000153.1/15367-15545 | 93.1 | 29 | 2 | 0 | 9 | 37 | 136 | 164 | 6.00E-004 | 42.1 |
| candidate_1082 | RF00379;ydaO-yuaA;ABJJ01000079.1/20755-20959 | 93.1 | 29 | 2 | 0 | 9 | 37 | 162 | 190 | 6.00E-004 | 42.1 |
| candidate_1082 | RF00379;ydaO-yuaA;CP000474.1/4242499-4242644 | 93.1 | 29 | 2 | 0 | 10 | 38 | 105 | 133 | 6.00E-004 | 42.1 |
| candidate_1082 | RF00379;ydaO-yuaA;CP001341.1/2323594-2323799 | 100 | 21 | 0 | 0 | 4 | 24 | 158 | 178 | 6.00E-004 | 42.1 |
| candidate_1082 | RF00379;ydaO-yuaA;CU458896.1/867721-867926 | 100 | 21 | 0 | 0 | 11 | 31 | 163 | 183 | 6.00E-004 | 42.1 |
| candidate_1082 | RF00379;ydaO-yuaA;ACFG01000004.1/273300-273475 | 96 | 25 | 1 | 0 | 12 | 36 | 137 | 161 | 6.00E-004 | 42.1 |
| candidate_1082 | RF00379;ydaO-yuaA;BA000030.3/8541474-8541695 | 92.86 | 28 | 2 | 0 | 10 | 37 | 180 | 207 | 0 | 40.1 |
| candidate_1082 | RF00379;ydaO-yuaA;ACFA01000105.1/4052-4252 | 92.86 | 28 | 2 | 0 | 10 | 37 | 159 | 186 | 0 | 40.1 |
| candidate_1082 | RF00379;ydaO-yuaA;ACEW01000500.1/24190-24392 | 92.86 | 28 | 2 | 0 | 10 | 37 | 163 | 190 | 0 | 40.1 |
| candidate_1082 | RF00379;ydaO-yuaA;ABUA01000074.1/6888-7075 | 92.86 | 28 | 2 | 0 | 4 | 31 | 140 | 167 | 0 | 40.1 |
| candidate_1082 | RF00379;ydaO-yuaA;CP000923.1/2119053-2119184 | 90.32 | 31 | 3 | 0 | 8 | 38 | 89 | 119 | 0.01 | 38.2 |
| candidate_1082 | RF00379;ydaO-yuaA;AAWL01000023.1/24489-24636 | 90.32 | 31 | 3 | 0 | 8 | 38 | 104 | 134 | 0.01 | 38.2 |
| candidate_1082 | RF00379;ydaO-yuaA;CP000612.1/3033561-3033714 | 90.32 | 31 | 3 | 0 | 6 | 36 | 109 | 139 | 0.01 | 38.2 |
| candidate_1082 | RF00379;ydaO-yuaA;ACNM01000019.1/34732-34872 | 95.65 | 23 | 1 | 0 | 9 | 31 | 98 | 120 | 0.01 | 38.2 |
| candidate_1082 | RF00379;ydaO-yuaA;BA000043.1/224415-224554 | 95.65 | 23 | 1 | 0 | 9 | 31 | 97 | 119 | 0.01 | 38.2 |
| candidate_1082 | RF00379;ydaO-yuaA;ACED01000001.1/34840-34979 | 95.65 | 23 | 1 | 0 | 9 | 31 | 97 | 119 | 0.01 | 38.2 |
| candidate_1082 | RF00379;ydaO-yuaA;ACCS01000001.1/187371-187512 | 90.32 | 31 | 3 | 0 | 8 | 38 | 98 | 128 | 0.01 | 38.2 |
| candidate_1082 | RF00379;ydaO-yuaA;CP000509.1/1318708-1318855 | 90.32 | 31 | 3 | 0 | 9 | 39 | 107 | 137 | 0.01 | 38.2 |
| candidate_1082 | RF00379;ydaO-yuaA;CP000454.1/861421-861614 | 92.59 | 27 | 2 | 0 | 10 | 36 | 153 | 179 | 0.01 | 38.2 |
| candidate_1082 | RF00379;ydaO-yuaA;CP000475.1/172193-172398 | 92.59 | 27 | 2 | 0 | 10 | 36 | 165 | 191 | 0.01 | 38.2 |
| candidate_1082 | RF00379;ydaO-yuaA;CP000820.1/8014703-8014880 | 90.32 | 31 | 3 | 0 | 9 | 39 | 135 | 165 | 0.01 | 38.2 |
| candidate_1083 | RF00379;ydaO-yuaA;ACHP01000024.1/35645-35867 | 100 | 35 | 0 | 0 | 1 | 35 | 9 | 43 | 1.00E-012 | 69.9 |
| candidate_1083 | RF00379;ydaO-yuaA;ABGN01000022.1/10987-11148 | 100 | 35 | 0 | 0 | 1 | 35 | 9 | 43 | 1.00E-012 | 69.9 |
| candidate_1083 | RF00379;ydaO-yuaA;ABGL01000010.1/286738-286960 | 100 | 35 | 0 | 0 | 1 | 35 | 9 | 43 | 1.00E-012 | 69.9 |
| candidate_1133 | RF00174;Cobalamin;ABIN01000323.1/12685-12982 | 94.74 | 38 | 2 | 0 | 1 | 38 | 253 | 290 | 1.00E-009 | 60.0 |
| candidate_1133 | RF00174;Cobalamin;AE016958.1/2995864-2996105 | 92.11 | 38 | 3 | 0 | 1 | 38 | 197 | 234 | 4.00E-007 | 52.0 |
| candidate_1133 | RF00174;Cobalamin;CP000854.1/3553203-3553413 | 96.67 | 30 | 1 | 0 | 1 | 30 | 166 | 195 | 4.00E-007 | 52.0 |
| candidate_1133 | RF00174;Cobalamin;CP000717.1/1265267-1265417 | 100 | 25 | 0 | 0 | 1 | 25 | 127 | 151 | 1.00E-006 | 50.1 |
| candidate_1133 | RF00174;Cobalamin;CP001472.1/2470423-2470618 | 100 | 25 | 0 | 0 | 5 | 29 | 155 | 179 | 1.00E-006 | 50.1 |
| candidate_1133 | RF00174;Cobalamin;Z94723.1/22998-23246 | 96.43 | 28 | 1 | 0 | 1 | 28 | 204 | 231 | 6.00E-006 | 48.1 |
| candidate_1133 | RF00174;Cobalamin;ACES01000182.1/23171-23389 | 100 | 22 | 0 | 0 | 10 | 31 | 183 | 204 | 9.00E-005 | 44.1 |
| candidate_1133 | RF00174;Cobalamin;ACEU01000643.1/1615-1835 | 100 | 21 | 0 | 0 | 10 | 30 | 185 | 205 | 3.00E-004 | 42.1 |
| candidate_1133 | RF00174;Cobalamin;ACBV01000034.1/6489-6629 | 96 | 25 | 1 | 0 | 1 | 25 | 117 | 141 | 3.00E-004 | 42.1 |
| candidate_1133 | RF00174;Cobalamin;CP000325.1/141018-141169 | 96 | 25 | 1 | 0 | 1 | 25 | 128 | 152 | 3.00E-004 | 42.1 |
| candidate_1133 | RF00174;Cobalamin;ACEV01000048.1/217132-217351 | 100 | 20 | 0 | 0 | 10 | 29 | 184 | 203 | 0 | 40.1 |
| candidate_1133 | RF00174;Cobalamin;AM420293.1/5303054-5303317 | 100 | 20 | 0 | 0 | 10 | 29 | 228 | 247 | 0 | 40.1 |
| candidate_1133 | RF00174;Cobalamin;ABUM01000010.1/57901-58137 | 100 | 20 | 0 | 0 | 10 | 29 | 201 | 220 | 0 | 40.1 |
| candidate_1133 | RF00174;Cobalamin;AY033236.1/353-563 | 100 | 20 | 0 | 0 | 12 | 31 | 177 | 196 | 0 | 40.1 |
| candidate_1133 | RF00174;Cobalamin;ABUD01000001.1/219828-220105 | 100 | 20 | 0 | 0 | 12 | 31 | 244 | 263 | 0 | 40.1 |
| candidate_1133 | RF00174;Cobalamin;AATY01000057.1/1784-1987 | 100 | 19 | 0 | 0 | 13 | 31 | 171 | 189 | 0.01 | 38.2 |
| candidate_1133 | RF00174;Cobalamin;CP000020.2/2736076-2736294 | 100 | 19 | 0 | 0 | 13 | 31 | 186 | 204 | 0.01 | 38.2 |
| candidate_1133 | RF00174;Cobalamin;CP001139.1/2743570-2743795 | 100 | 19 | 0 | 0 | 13 | 31 | 193 | 211 | 0.01 | 38.2 |
| candidate_1133 | RF00174;Cobalamin;CP000155.1/7046067-7046289 | 100 | 19 | 0 | 0 | 11 | 29 | 188 | 206 | 0.01 | 38.2 |
| candidate_1133 | RF00174;Cobalamin;CP000514.1/4279828-4280063 | 100 | 19 | 0 | 0 | 12 | 30 | 202 | 220 | 0.01 | 38.2 |
| candidate_1133 | RF00174;Cobalamin;ABCP01000006.1/101233-101427 | 100 | 19 | 0 | 0 | 12 | 30 | 161 | 179 | 0.01 | 38.2 |
| candidate_1133 | RF00174;Cobalamin;ABCS01000025.1/79534-79736 | 100 | 19 | 0 | 0 | 10 | 28 | 167 | 185 | 0.01 | 38.2 |
| candidate_1133 | RF00174;Cobalamin;CP000113.1/8100515-8100727 | 95.65 | 23 | 1 | 0 | 6 | 28 | 173 | 195 | 0.01 | 38.2 |
| candidate_1133 | RF00174;Cobalamin;AAMD01000084.1/10237-10455 | 95.65 | 23 | 1 | 0 | 6 | 28 | 179 | 201 | 0.01 | 38.2 |
| candidate_1133 | RF00174;Cobalamin;AP006840.1/361720-361924 | 95.65 | 23 | 1 | 0 | 6 | 28 | 165 | 187 | 0.01 | 38.2 |
| candidate_1133 | RF00174;Cobalamin;AF263012.1/9015-9229 | 100 | 19 | 0 | 0 | 10 | 28 | 179 | 197 | 0.01 | 38.2 |
| candidate_1133 | RF00174;Cobalamin;AL939107.1/141879-142126 | 100 | 19 | 0 | 0 | 11 | 29 | 213 | 231 | 0.01 | 38.2 |
| candidate_1133 | RF00174;Cobalamin;ABYB01000334.1/10936-11151 | 100 | 19 | 0 | 0 | 10 | 28 | 180 | 198 | 0.01 | 38.2 |
| candidate_1133 | RF00174;Cobalamin;ACLF01000696.1/1086-1319 | 100 | 19 | 0 | 0 | 12 | 30 | 200 | 218 | 0.01 | 38.2 |
| candidate_1133 | RF00174;Cobalamin;ABUS01000002.1/716306-716507 | 100 | 19 | 0 | 0 | 10 | 28 | 166 | 184 | 0.01 | 38.2 |
| candidate_1133 | RF00174;Cobalamin;CP000481.1/1939207-1939426 | 100 | 19 | 0 | 0 | 13 | 31 | 187 | 205 | 0.01 | 38.2 |
| candidate_1133 | RF00174;Cobalamin;AP008957.1/5154507-5154732 | 100 | 19 | 0 | 0 | 10 | 28 | 190 | 208 | 0.01 | 38.2 |
| candidate_1133 | RF00174;Cobalamin;CU458896.1/552467-552689 | 100 | 19 | 0 | 0 | 11 | 29 | 188 | 206 | 0.01 | 38.2 |
| candidate_1134 | RF00174;Cobalamin;CP000717.1/1265267-1265417 | 100 | 44 | 0 | 0 | 1 | 44 | 50 | 93 | 8.00E-018 | 87.7 |
| candidate_1184 | RF00177;SSU_rRNA_5;DQ011851.1/4-387 | 100 | 21 | 0 | 0 | 120 | 140 | 48 | 68 | 0 | 42.1 |
| candidate_1184 | RF00177;SSU_rRNA_5;EU771075.1/22-494 | 100 | 21 | 0 | 0 | 121 | 141 | 238 | 258 | 0 | 42.1 |
| candidate_1184 | RF00177;SSU_rRNA_5;FM179912.1/2-489 | 96 | 25 | 1 | 0 | 116 | 140 | 1 | 25 | 0 | 42.1 |
| candidate_1184 | RF00177;SSU_rRNA_5;DQ303436.1/22-498 | 100 | 20 | 0 | 0 | 121 | 140 | 242 | 261 | 0.01 | 40.1 |
| candidate_1184 | RF00177;SSU_rRNA_5;DQ278905.1/1-440 | 100 | 20 | 0 | 0 | 121 | 140 | 230 | 249 | 0.01 | 40.1 |
| candidate_1184 | RF00177;SSU_rRNA_5;AM182485.1/17-428 | 100 | 20 | 0 | 0 | 121 | 140 | 202 | 221 | 0.01 | 40.1 |
| candidate_1184 | RF00177;SSU_rRNA_5;DQ924832.1/12-439 | 100 | 20 | 0 | 0 | 121 | 140 | 23 | 42 | 0.01 | 40.1 |
| candidate_1184 | RF00177;SSU_rRNA_5;DQ924832.1/12-439 | 100 | 20 | 0 | 0 | 121 | 140 | 57 | 76 | 0.01 | 40.1 |
| candidate_1184 | RF00177;SSU_rRNA_5;DQ924832.1/12-439 | 100 | 20 | 0 | 0 | 121 | 140 | 131 | 150 | 0.01 | 40.1 |
| candidate_1184 | RF00177;SSU_rRNA_5;AY211112.1/5-358 | 100 | 20 | 0 | 0 | 121 | 140 | 37 | 56 | 0.01 | 40.1 |
| candidate_1184 | RF00177;SSU_rRNA_5;AY211112.1/5-358 | 100 | 20 | 0 | 0 | 121 | 140 | 74 | 93 | 0.01 | 40.1 |
| candidate_1186 | RF00177;SSU_rRNA_5;AJ578493.1/1-369 | 97.87 | 94 | 2 | 0 | 63 | 156 | 276 | 369 | 5.00E-042 | 170 |
| candidate_1186 | RF00177;SSU_rRNA_5;AJ578491.1/8-381 | 100 | 84 | 0 | 0 | 102 | 185 | 291 | 374 | 8.00E-041 | 167 |
| candidate_1186 | RF00177;SSU_rRNA_5;DQ682956.1/47-498 | 88.3 | 94 | 8 | 1 | 76 | 169 | 15 | 105 | 3.00E-019 | 95.6 |
| candidate_1186 | RF00177;SSU_rRNA_5;AJ578492.1/1-405 | 87.69 | 65 | 5 | 1 | 38 | 99 | 341 | 405 | 3.00E-009 | 61.9 |
| candidate_1186 | RF00177;SSU_rRNA_5;AJ578490.1/1-433 | 94.59 | 37 | 2 | 0 | 63 | 99 | 397 | 433 | 5.00E-008 | 58.0 |
| candidate_1186 | RF00177;SSU_rRNA_5;AY211112.1/5-358 | 100 | 23 | 0 | 0 | 1 | 23 | 13 | 35 | 2.00E-004 | 46.1 |
| candidate_1238 | RF00013;6S;CP000614.1/2817923-2818104 | 100 | 20 | 0 | 0 | 33 | 52 | 40 | 59 | 0.01 | 40.1 |
| candidate_1238 | RF00013;6S;ABBE01000293.1/11364-11545 | 100 | 20 | 0 | 0 | 33 | 52 | 40 | 59 | 0.01 | 40.1 |
| candidate_1238 | RF00013;6S;CP001052.1/1115043-1115224 | 100 | 20 | 0 | 0 | 33 | 52 | 40 | 59 | 0.01 | 40.1 |
| candidate_1393 | RF00010;RNaseP_bact_a;ABIN01000066.1/3901-4302 | 100 | 26 | 0 | 0 | 1 | 26 | 377 | 402 | 2.00E-007 | 52.0 |
| candidate_1393 | RF00010;RNaseP_bact_a;AE016958.1/2182516-2182909 | 100 | 26 | 0 | 0 | 1 | 26 | 369 | 394 | 2.00E-007 | 52.0 |
| candidate_1393 | RF00010;RNaseP_bact_a;CP000325.1/1422068-1422493 | 100 | 26 | 0 | 0 | 1 | 26 | 401 | 426 | 2.00E-007 | 52.0 |
| candidate_1393 | RF00010;RNaseP_bact_a;CU458896.1/1909668-1910072 | 100 | 23 | 0 | 0 | 1 | 23 | 380 | 402 | 2.00E-005 | 46.1 |
| candidate_1393 | RF00010;RNaseP_bact_a;CP000480.1/4392645-4393032 | 100 | 23 | 0 | 0 | 1 | 23 | 363 | 385 | 2.00E-005 | 46.1 |
| candidate_1393 | RF00010;RNaseP_bact_a;AM420293.1/1744463-1744869 | 96.15 | 26 | 1 | 0 | 1 | 26 | 382 | 407 | 6.00E-005 | 44.1 |
| candidate_1393 | RF00010;RNaseP_bact_a;AP006618.1/1794140-1794557 | 96.15 | 26 | 1 | 0 | 1 | 26 | 393 | 418 | 6.00E-005 | 44.1 |
| candidate_1393 | RF00010;RNaseP_bact_a;ACBV01000004.1/123669-124085 | 100 | 22 | 0 | 0 | 1 | 22 | 392 | 413 | 6.00E-005 | 44.1 |
| candidate_1393 | RF00010;RNaseP_bact_a;CP000384.1/3536871-3537274 | 96.15 | 26 | 1 | 0 | 1 | 26 | 379 | 404 | 6.00E-005 | 44.1 |
| candidate_1393 | RF00010;RNaseP_bact_a;CP000820.1/6067977-6068392 | 95.83 | 24 | 1 | 0 | 3 | 26 | 393 | 416 | 0 | 40.1 |
| candidate_1393 | RF00010;RNaseP_bact_a;AACY023084164.1/291-639 | 100 | 19 | 0 | 0 | 3 | 21 | 326 | 344 | 0 | 38.2 |
| candidate_1393 | RF00010;RNaseP_bact_a;AAGA01006953.1/690-1052 | 100 | 19 | 0 | 0 | 3 | 21 | 340 | 358 | 0 | 38.2 |
| candidate_1393 | RF00010;RNaseP_bact_a;AAFZ01021408.1/342-696 | 100 | 19 | 0 | 0 | 3 | 21 | 332 | 350 | 0 | 38.2 |
| candidate_1393 | RF00010;RNaseP_bact_a;CP000431.1/1253273-1253711 | 95.65 | 23 | 1 | 0 | 1 | 23 | 414 | 436 | 0 | 38.2 |
| candidate_1393 | RF00373;RNaseP_arch;AACY023084164.1/296-638 | 100 | 19 | 0 | 0 | 3 | 21 | 325 | 343 | 0 | 38.2 |
| candidate_1603 | RF01066;6C;CU458896.1/433380-433455 | 96 | 50 | 2 | 0 | 19 | 68 | 2 | 51 | 3.00E-016 | 83.8 |
| candidate_1603 | RF01066;6C;CP000854.1/6232183-6232258 | 94.74 | 76 | 4 | 0 | 18 | 93 | 1 | 76 | 2.00E-014 | 77.8 |
| candidate_1603 | RF01066;6C;CP000480.1/6241132-6241207 | 90.2 | 51 | 5 | 0 | 18 | 68 | 1 | 51 | 1.00E-009 | 61.9 |
| candidate_1603 | RF01066;6C;ACEV01000088.1/209957-210031 | 100 | 23 | 0 | 0 | 24 | 46 | 7 | 29 | 7.00E-005 | 46.1 |
| candidate_1603 | RF01066;6C;AP011115.1/4577196-4577271 | 91.3 | 46 | 2 | 2 | 24 | 68 | 7 | 51 | 3.00E-004 | 44.1 |
| candidate_1603 | RF01066;6C;ABTV01000003.1/155059-155134 | 86.67 | 45 | 6 | 0 | 24 | 68 | 7 | 51 | 0 | 42.1 |
| candidate_1621 | RF00169;SRP_bact;ABGL01000088.1/303994-304099 | 100 | 89 | 0 | 0 | 1 | 89 | 6 | 94 | 3.00E-044 | 176 |
| candidate_1621 | RF00169;SRP_bact;AE016958.1/347342-347444 | 98.88 | 89 | 1 | 0 | 1 | 89 | 6 | 94 | 7.00E-042 | 168 |
| candidate_1621 | RF00169;SRP_bact;ACBV01000050.1/7788-7891 | 98.88 | 89 | 1 | 0 | 1 | 89 | 7 | 95 | 7.00E-042 | 168 |
| candidate_1621 | RF00169;SRP_bact;CU458896.1/306561-306663 | 97.75 | 89 | 2 | 0 | 1 | 89 | 6 | 94 | 2.00E-039 | 161 |
| candidate_1621 | RF00169;SRP_bact;CP000854.1/6331088-6331187 | 97.73 | 88 | 2 | 0 | 1 | 88 | 6 | 93 | 7.00E-039 | 159 |
| candidate_1621 | RF00169;SRP_bact;AL023596.1/21928-22027 | 96.63 | 89 | 3 | 0 | 1 | 89 | 6 | 94 | 4.00E-037 | 153 |
| candidate_1621 | RF00169;SRP_bact;CP000480.1/6352822-6352925 | 96.63 | 89 | 3 | 0 | 1 | 89 | 7 | 95 | 4.00E-037 | 153 |
| candidate_1621 | RF00169;SRP_bact;CP000518.1/5240093-5240194 | 96.63 | 89 | 3 | 0 | 1 | 89 | 8 | 96 | 4.00E-037 | 153 |
| candidate_1621 | RF00169;SRP_bact;CP000580.1/5529817-5529919 | 96.63 | 89 | 3 | 0 | 1 | 89 | 6 | 94 | 4.00E-037 | 153 |
| candidate_1621 | RF00169;SRP_bact;CP000656.1/1332663-1332758 | 96.63 | 89 | 3 | 0 | 1 | 89 | 4 | 92 | 4.00E-037 | 153 |
| candidate_1621 | RF00169;SRP_bact;ABTV01000003.1/58907-59008 | 95.29 | 85 | 4 | 0 | 5 | 89 | 10 | 94 | 2.00E-032 | 137 |
| candidate_1621 | RF00169;SRP_bact;ABVA01000001.1/516826-516927 | 93.26 | 89 | 6 | 0 | 1 | 89 | 6 | 94 | 6.00E-030 | 129 |
| candidate_1621 | RF00169;SRP_bact;AP006618.1/282139-282237 | 94.12 | 85 | 5 | 0 | 5 | 89 | 9 | 93 | 6.00E-030 | 129 |
| candidate_1621 | RF00169;SRP_bact;AP008957.1/400430-400532 | 93.9 | 82 | 5 | 0 | 5 | 86 | 10 | 91 | 4.00E-028 | 123 |
| candidate_1621 | RF00169;SRP_bact;CR931997.1/2352931-2353032 | 92.86 | 84 | 6 | 0 | 6 | 89 | 11 | 94 | 6.00E-027 | 119 |
| candidate_1621 | RF00169;SRP_bact;AM420293.1/285506-285605 | 91.01 | 89 | 8 | 0 | 1 | 89 | 6 | 94 | 4.00E-025 | 113 |
| candidate_1621 | RF00169;SRP_bact;CP001620.1/194226-194327 | 90.48 | 84 | 8 | 0 | 6 | 89 | 11 | 94 | 3.00E-022 | 103 |
| candidate_1621 | RF00169;SRP_bact;AP009044.1/340385-340486 | 89.41 | 85 | 9 | 0 | 5 | 89 | 10 | 94 | 2.00E-020 | 97.6 |
| candidate_1621 | RF00169;SRP_bact;ACGE01000132.1/19452-19553 | 89.02 | 82 | 9 | 0 | 6 | 87 | 11 | 92 | 1.00E-018 | 91.7 |
| candidate_1621 | RF00169;SRP_bact;AM942444.1/2244482-2244581 | 88.89 | 81 | 9 | 0 | 6 | 86 | 11 | 91 | 5.00E-018 | 89.7 |
| candidate_1621 | RF00169;SRP_bact;ABZU01000024.1/11645-11748 | 90.91 | 66 | 6 | 0 | 24 | 89 | 30 | 95 | 3.00E-016 | 83.8 |
| candidate_1621 | RF00169;SRP_bact;ACHF01000104.1/294-397 | 87.06 | 85 | 11 | 0 | 5 | 89 | 11 | 95 | 1.00E-015 | 81.8 |
| candidate_1621 | RF00169;SRP_bact;AAGP01000014.1/28727-28828 | 88.75 | 80 | 8 | 1 | 4 | 83 | 10 | 88 | 5.00E-015 | 79.8 |
| candidate_1621 | RF00169;SRP_bact;CP000820.1/7984971-7985064 | 91.67 | 60 | 5 | 0 | 24 | 83 | 26 | 85 | 5.00E-015 | 79.8 |
| candidate_1621 | RF00169;SRP_bact;ACEB01000003.1/4143-4247 | 89.39 | 66 | 7 | 0 | 24 | 89 | 29 | 94 | 8.00E-014 | 75.8 |
| candidate_1621 | RF00169;SRP_bact;CP000249.1/312017-312118 | 90 | 60 | 6 | 0 | 24 | 83 | 30 | 89 | 1.00E-012 | 71.9 |
| candidate_1621 | RF00169;SRP_bact;CT573213.2/674087-674190 | 90 | 60 | 6 | 0 | 24 | 83 | 31 | 90 | 1.00E-012 | 71.9 |
| candidate_1621 | RF00169;SRP_bact;ACEV01000088.1/328365-328468 | 86.67 | 75 | 10 | 0 | 1 | 75 | 7 | 81 | 5.00E-012 | 69.9 |
| candidate_1621 | RF00169;SRP_bact;ABTU01000019.1/128719-128811 | 88.89 | 63 | 7 | 0 | 21 | 83 | 22 | 84 | 5.00E-012 | 69.9 |
| candidate_1621 | RF00169;SRP_bact;ACLF01000389.1/629-727 | 90.74 | 54 | 5 | 0 | 27 | 80 | 32 | 85 | 2.00E-011 | 67.9 |
| candidate_1621 | RF00169;SRP_bact;ACLJ01000058.1/6662-6763 | 87.14 | 70 | 9 | 0 | 18 | 87 | 23 | 92 | 2.00E-011 | 67.9 |
| candidate_1621 | RF00169;SRP_bact;AACY022920660.1/288-381 | 90.57 | 53 | 5 | 0 | 31 | 83 | 33 | 85 | 8.00E-011 | 65.9 |
| candidate_1621 | RF00169;SRP_bact;ABUM01000018.1/88711-88812 | 86.42 | 81 | 10 | 1 | 9 | 89 | 14 | 93 | 8.00E-011 | 65.9 |
| candidate_1621 | RF00169;SRP_bact;AP009152.1/317215-317312 | 89.47 | 57 | 6 | 0 | 27 | 83 | 33 | 89 | 8.00E-011 | 65.9 |
| candidate_1621 | RF00169;SRP_bact;BX248354.1/219353-219454 | 87.69 | 65 | 8 | 0 | 25 | 89 | 30 | 94 | 8.00E-011 | 65.9 |
| candidate_1621 | RF00169;SRP_bact;AACY023337942.1/742-836 | 100 | 32 | 0 | 0 | 26 | 57 | 29 | 60 | 3.00E-010 | 63.9 |
| candidate_1621 | RF00169;SRP_bact;AACY021237078.1/632-727 | 100 | 32 | 0 | 0 | 26 | 57 | 29 | 60 | 3.00E-010 | 63.9 |
| candidate_1621 | RF00169;SRP_bact;AACY020697676.1/171-264 | 100 | 32 | 0 | 0 | 26 | 57 | 28 | 59 | 3.00E-010 | 63.9 |
| candidate_1621 | RF00169;SRP_bact;ABUI01000017.1/126668-126771 | 90.38 | 52 | 5 | 0 | 32 | 83 | 37 | 88 | 3.00E-010 | 63.9 |
| candidate_1621 | RF00169;SRP_bact;ACEW01000332.1/25149-25246 | 90.38 | 52 | 5 | 0 | 27 | 78 | 30 | 81 | 3.00E-010 | 63.9 |
| candidate_1621 | RF00169;SRP_bact;AACY020302892.1/6158-6261 | 90 | 50 | 5 | 0 | 31 | 80 | 38 | 87 | 5.00E-009 | 60.0 |
| candidate_1621 | RF00169;SRP_bact;ABUU01000182.1/9279-9383 | 87.14 | 70 | 8 | 1 | 7 | 76 | 14 | 82 | 5.00E-009 | 60.0 |
| candidate_1621 | RF00169;SRP_bact;CP000509.1/339856-339944 | 88.89 | 54 | 6 | 0 | 30 | 83 | 29 | 82 | 5.00E-009 | 60.0 |
| candidate_1621 | RF00169;SRP_bact;ABUS01000007.1/74669-74767 | 86.49 | 74 | 9 | 1 | 7 | 80 | 12 | 84 | 5.00E-009 | 60.0 |
| candidate_1621 | RF00169;SRP_bact;ABYS02000013.1/133293-133394 | 88.68 | 53 | 6 | 0 | 26 | 78 | 33 | 85 | 2.00E-008 | 58.0 |
| candidate_1621 | RF00169;SRP_bact;ABUZ01000004.1/445297-445402 | 86.15 | 65 | 9 | 0 | 19 | 83 | 25 | 89 | 2.00E-008 | 58.0 |
| candidate_1621 | RF00169;SRP_bact;ABJG01000092.1/10000-10101 | 96.97 | 33 | 1 | 0 | 25 | 57 | 30 | 62 | 2.00E-008 | 58.0 |
| candidate_1621 | RF00169;SRP_bact;ABYB01000171.1/1258-1349 | 96.97 | 33 | 1 | 0 | 25 | 57 | 26 | 58 | 2.00E-008 | 58.0 |
| candidate_1621 | RF00169;SRP_bact;AL939118.1/230078-230174 | 96.97 | 33 | 1 | 0 | 25 | 57 | 27 | 59 | 2.00E-008 | 58.0 |
| candidate_1621 | RF00169;SRP_bact;ABUC01000014.1/199031-199124 | 87.72 | 57 | 7 | 0 | 27 | 83 | 29 | 85 | 2.00E-008 | 58.0 |
| candidate_1621 | RF00169;SRP_bact;CP000481.1/2292428-2292521 | 88.68 | 53 | 6 | 0 | 31 | 83 | 33 | 85 | 2.00E-008 | 58.0 |
| candidate_1621 | RF00169;SRP_bact;CP000850.1/277831-277920 | 88.46 | 52 | 6 | 0 | 32 | 83 | 32 | 83 | 7.00E-008 | 56.0 |
| candidate_1621 | RF00169;SRP_bact;ABVC01000007.1/68519-68612 | 96.88 | 32 | 1 | 0 | 26 | 57 | 28 | 59 | 7.00E-008 | 56.0 |
| candidate_1621 | RF00169;SRP_bact;ABUH01000002.1/60930-61031 | 88.14 | 59 | 6 | 1 | 31 | 89 | 36 | 93 | 3.00E-007 | 54.0 |
| candidate_1621 | RF00169;SRP_bact;ABTA01000005.1/4846-4945 | 86.21 | 58 | 8 | 0 | 32 | 89 | 37 | 94 | 1.00E-006 | 52.0 |
| candidate_1621 | RF00169;SRP_bact;AACY021288407.1/181-279 | 93.94 | 33 | 2 | 0 | 25 | 57 | 29 | 61 | 4.00E-006 | 50.1 |
| candidate_1621 | RF00169;SRP_bact;AACY023313026.1/474-572 | 93.94 | 33 | 2 | 0 | 25 | 57 | 29 | 61 | 4.00E-006 | 50.1 |
| candidate_1621 | RF00169;SRP_bact;AACY023243827.1/857-959 | 93.94 | 33 | 2 | 0 | 25 | 57 | 30 | 62 | 4.00E-006 | 50.1 |
| candidate_1621 | RF00169;SRP_bact;AACY020475285.1/1472-1568 | 93.94 | 33 | 2 | 0 | 25 | 57 | 29 | 61 | 4.00E-006 | 50.1 |
| candidate_1621 | RF00169;SRP_bact;AACY023316480.1/1116-1210 | 93.94 | 33 | 2 | 0 | 25 | 57 | 28 | 60 | 4.00E-006 | 50.1 |
| candidate_1621 | RF00169;SRP_bact;BA000030.3/5095051-5095153 | 93.94 | 33 | 2 | 0 | 25 | 57 | 30 | 62 | 4.00E-006 | 50.1 |
| candidate_1621 | RF00169;SRP_bact;CP000474.1/687147-687246 | 86.79 | 53 | 7 | 0 | 25 | 77 | 30 | 82 | 4.00E-006 | 50.1 |
| candidate_1621 | RF00169;SRP_bact;AE016822.1/334706-334801 | 85.96 | 57 | 8 | 0 | 27 | 83 | 30 | 86 | 4.00E-006 | 50.1 |
| candidate_1621 | RF00169;SRP_bact;CP000750.2/1826519-1826614 | 93.94 | 33 | 2 | 0 | 25 | 57 | 28 | 60 | 4.00E-006 | 50.1 |
| candidate_1621 | RF00169;SRP_bact;AACY023439272.1/682-783 | 93.75 | 32 | 2 | 0 | 26 | 57 | 31 | 62 | 2.00E-005 | 48.1 |
| candidate_1621 | RF00169;SRP_bact;AACY020777139.1/709-809 | 93.75 | 32 | 2 | 0 | 26 | 57 | 30 | 61 | 2.00E-005 | 48.1 |
| candidate_1621 | RF00169;SRP_bact;AACY021919266.1/153-248 | 93.75 | 32 | 2 | 0 | 26 | 57 | 29 | 60 | 2.00E-005 | 48.1 |
| candidate_1621 | RF00169;SRP_bact;AACY020487187.1/651-749 | 93.75 | 32 | 2 | 0 | 26 | 57 | 30 | 61 | 2.00E-005 | 48.1 |
| candidate_1621 | RF00169;SRP_bact;AACY020102136.1/387-488 | 96.43 | 28 | 1 | 0 | 30 | 57 | 34 | 61 | 2.00E-005 | 48.1 |
| candidate_1621 | RF00169;SRP_bact;AACY020165682.1/1390-1450 | 93.75 | 32 | 2 | 0 | 26 | 57 | 13 | 44 | 2.00E-005 | 48.1 |
| candidate_1621 | RF00169;SRP_bact;ABSP01034286.1/459-554 | 100 | 24 | 0 | 0 | 32 | 55 | 34 | 57 | 2.00E-005 | 48.1 |
| candidate_1621 | RF00169;SRP_bact;AACY020621520.1/182-288 | 93.75 | 32 | 2 | 0 | 26 | 57 | 32 | 63 | 2.00E-005 | 48.1 |
| candidate_1621 | RF00169;SRP_bact;CP000088.1/57389-57486 | 86.54 | 52 | 7 | 0 | 32 | 83 | 35 | 86 | 2.00E-005 | 48.1 |
| candidate_1621 | RF00169;SRP_bact;ABUD01000002.1/178064-178157 | 93.75 | 32 | 2 | 0 | 26 | 57 | 28 | 59 | 2.00E-005 | 48.1 |
| candidate_1621 | RF00169;SRP_bact;ABTF01000004.1/21746-21844 | 93.75 | 32 | 2 | 0 | 25 | 56 | 30 | 61 | 2.00E-005 | 48.1 |
| candidate_1621 | RF00169;SRP_bact;ABTJ01000169.1/148-241 | 93.75 | 32 | 2 | 0 | 25 | 56 | 27 | 58 | 2.00E-005 | 48.1 |
| candidate_1621 | RF00169;SRP_bact;ABTI01000001.1/579950-580049 | 93.75 | 32 | 2 | 0 | 27 | 58 | 32 | 63 | 2.00E-005 | 48.1 |
| candidate_1621 | RF00169;SRP_bact;ABMI01012063.1/74-120 | 96.3 | 27 | 1 | 0 | 31 | 57 | 11 | 37 | 7.00E-005 | 46.1 |
| candidate_1621 | RF00169;SRP_bact;AACY020058466.1/193-287 | 96.3 | 27 | 1 | 0 | 30 | 56 | 32 | 58 | 7.00E-005 | 46.1 |
| candidate_1621 | RF00169;SRP_bact;AACY023445801.1/1229-1329 | 93.55 | 31 | 2 | 0 | 27 | 57 | 32 | 62 | 7.00E-005 | 46.1 |
| candidate_1621 | RF00169;SRP_bact;ACCG01000036.1/11994-12097 | 93.55 | 31 | 2 | 0 | 27 | 57 | 33 | 63 | 7.00E-005 | 46.1 |
| candidate_1621 | RF00169;SRP_bact;ABXY01000023.1/230851-230954 | 93.55 | 31 | 2 | 0 | 27 | 57 | 32 | 62 | 7.00E-005 | 46.1 |
| candidate_1621 | RF00169;SRP_bact;AAXD02000018.1/304180-304277 | 93.55 | 31 | 2 | 0 | 27 | 57 | 31 | 61 | 7.00E-005 | 46.1 |
| candidate_1621 | RF00169;SRP_bact;CP000605.1/1473849-1473940 | 93.55 | 31 | 2 | 0 | 27 | 57 | 28 | 58 | 7.00E-005 | 46.1 |
| candidate_1621 | RF00169;SRP_bact;ACES01000181.1/1347-1443 | 86.27 | 51 | 7 | 0 | 32 | 82 | 36 | 86 | 7.00E-005 | 46.1 |
| candidate_1621 | RF00169;SRP_bact;ABUA01000027.1/12445-12546 | 96.3 | 27 | 1 | 0 | 30 | 56 | 35 | 61 | 7.00E-005 | 46.1 |
| candidate_1621 | RF00169;SRP_bact;AAMN01000001.1/951066-951161 | 93.55 | 31 | 2 | 0 | 26 | 56 | 29 | 59 | 7.00E-005 | 46.1 |
| candidate_1621 | RF00169;SRP_bact;BABE01019455.1/792-890 | 93.33 | 30 | 2 | 0 | 28 | 57 | 33 | 62 | 3.00E-004 | 44.1 |
| candidate_1621 | RF00169;SRP_bact;AB205012.1/18642-18740 | 85.19 | 54 | 8 | 0 | 25 | 78 | 29 | 82 | 3.00E-004 | 44.1 |
| candidate_1621 | RF00169;SRP_bact;AAOB01000010.1/2849-2948 | 85.19 | 54 | 8 | 0 | 27 | 80 | 32 | 85 | 3.00E-004 | 44.1 |
| candidate_1621 | RF00169;SRP_bact;AACY020309506.1/1457-1553 | 90.91 | 33 | 3 | 0 | 25 | 57 | 28 | 60 | 0 | 42.1 |
| candidate_1621 | RF00169;SRP_bact;ABPQ01010894.1/167-268 | 90.91 | 33 | 3 | 0 | 25 | 57 | 30 | 62 | 0 | 42.1 |
| candidate_1621 | RF00169;SRP_bact;AACY022573530.1/363-464 | 90.91 | 33 | 3 | 0 | 25 | 57 | 30 | 62 | 0 | 42.1 |
| candidate_1621 | RF00169;SRP_bact;AACY022003726.1/669-766 | 90.91 | 33 | 3 | 0 | 25 | 57 | 28 | 60 | 0 | 42.1 |
| candidate_1621 | RF00169;SRP_bact;ABCM01000002.1/364118-364220 | 90.91 | 33 | 3 | 0 | 25 | 57 | 30 | 62 | 0 | 42.1 |
| candidate_1621 | RF00169;SRP_bact;ACGF01000005.1/3899-3996 | 84.91 | 53 | 8 | 0 | 31 | 83 | 34 | 86 | 0 | 42.1 |
| candidate_1621 | RF00169;SRP_bact;ABQP01000019.1/86764-86866 | 86.67 | 45 | 6 | 0 | 31 | 75 | 37 | 81 | 0 | 42.1 |
| candidate_1621 | RF00169;SRP_bact;BABD01033951.1/147-248 | 90.62 | 32 | 3 | 0 | 26 | 57 | 31 | 62 | 0 | 40.1 |
| candidate_1621 | RF00169;SRP_bact;BABD01019804.1/513-615 | 90.62 | 32 | 3 | 0 | 26 | 57 | 31 | 62 | 0 | 40.1 |
| candidate_1621 | RF00169;SRP_bact;AACY021514576.1/763-864 | 90.62 | 32 | 3 | 0 | 26 | 57 | 31 | 62 | 0 | 40.1 |
| candidate_1621 | RF00169;SRP_bact;AAFX01005108.1/1068-1168 | 90.62 | 32 | 3 | 0 | 26 | 57 | 31 | 62 | 0 | 40.1 |
| candidate_1621 | RF00169;SRP_bact;ABVV01000005.1/45701-45802 | 92.86 | 28 | 2 | 0 | 30 | 57 | 34 | 61 | 0 | 40.1 |
| candidate_1621 | RF00169;SRP_bact;AP010656.1/1061855-1061957 | 92.86 | 28 | 2 | 0 | 30 | 57 | 35 | 62 | 0 | 40.1 |
| candidate_1621 | RF00169;SRP_bact;ACKW01000052.1/150019-150113 | 95.83 | 24 | 1 | 0 | 32 | 55 | 34 | 57 | 0 | 40.1 |
| candidate_1621 | RF00169;SRP_bact;ACFG01000004.1/292411-292513 | 95.83 | 24 | 1 | 0 | 32 | 55 | 38 | 61 | 0 | 40.1 |
| candidate_1672 | RF00504;Glycine;ACHP01000005.1/397457-397569 | 100 | 37 | 0 | 0 | 1 | 37 | 30 | 66 | 9.00E-014 | 73.8 |
| candidate_1689 | RF00174;Cobalamin;Z94723.1/22998-23246 | 97.92 | 48 | 1 | 0 | 18 | 65 | 73 | 120 | 1.00E-017 | 87.7 |
| candidate_1689 | RF00174;Cobalamin;CP000717.1/1265267-1265417 | 100 | 43 | 0 | 0 | 23 | 65 | 1 | 43 | 5.00E-017 | 85.7 |
| candidate_1689 | RF00174;Cobalamin;CU458896.1/4710696-4710910 | 97.67 | 43 | 1 | 0 | 23 | 65 | 44 | 86 | 1.00E-014 | 77.8 |
| candidate_1689 | RF00174;Cobalamin;AP011115.1/3271397-3271539 | 97.56 | 41 | 1 | 0 | 23 | 63 | 1 | 41 | 2.00E-013 | 73.8 |
| candidate_1689 | RF00174;Cobalamin;ABIN01000323.1/12685-12982 | 92.45 | 53 | 4 | 0 | 13 | 65 | 72 | 124 | 2.00E-013 | 73.8 |
| candidate_1689 | RF00174;Cobalamin;CP000480.1/6689978-6690152 | 95.56 | 45 | 2 | 0 | 21 | 65 | 28 | 72 | 2.00E-013 | 73.8 |
| candidate_1689 | RF00174;Cobalamin;CP000854.1/3553203-3553413 | 95.45 | 44 | 2 | 0 | 22 | 65 | 42 | 85 | 8.00E-013 | 71.9 |
| candidate_1689 | RF00174;Cobalamin;AP008957.1/5154507-5154732 | 95.35 | 43 | 2 | 0 | 22 | 64 | 61 | 103 | 3.00E-012 | 69.9 |
| candidate_1689 | RF00174;Cobalamin;AE016958.1/2995864-2996105 | 95.35 | 43 | 2 | 0 | 23 | 65 | 33 | 75 | 3.00E-012 | 69.9 |
| candidate_1689 | RF00174;Cobalamin;AL939107.1/141879-142126 | 95.12 | 41 | 2 | 0 | 23 | 63 | 62 | 102 | 5.00E-011 | 65.9 |
| candidate_1689 | RF00174;Cobalamin;CP000325.1/141018-141169 | 93.02 | 43 | 3 | 0 | 23 | 65 | 3 | 45 | 8.00E-010 | 61.9 |
| candidate_1689 | RF00174;Cobalamin;ACEU01000643.1/1615-1835 | 91.3 | 46 | 4 | 0 | 18 | 63 | 47 | 92 | 3.00E-009 | 60.0 |
| candidate_1689 | RF00174;Cobalamin;ABUM01000010.1/57901-58137 | 91.3 | 46 | 4 | 0 | 18 | 63 | 44 | 89 | 3.00E-009 | 60.0 |
| candidate_1689 | RF00174;Cobalamin;ABUX01000003.1/480396-480590 | 92.68 | 41 | 3 | 0 | 23 | 63 | 45 | 85 | 1.00E-008 | 58.0 |
| candidate_1689 | RF00174;Cobalamin;CP001339.1/653458-653682 | 92.68 | 41 | 3 | 0 | 25 | 65 | 52 | 92 | 1.00E-008 | 58.0 |
| candidate_1689 | RF00174;Cobalamin;ABUU01000087.1/24235-24436 | 92.68 | 41 | 3 | 0 | 23 | 63 | 43 | 83 | 1.00E-008 | 58.0 |
| candidate_1689 | RF00174;Cobalamin;ABJH01000030.1/15760-15986 | 92.68 | 41 | 3 | 0 | 23 | 63 | 52 | 92 | 1.00E-008 | 58.0 |
| candidate_1689 | RF00174;Cobalamin;ACEV01000048.1/217132-217351 | 92.68 | 41 | 3 | 0 | 23 | 63 | 52 | 92 | 1.00E-008 | 58.0 |
| candidate_1689 | RF00174;Cobalamin;AL939107.1/133900-134052 | 92.68 | 41 | 3 | 0 | 23 | 63 | 2 | 42 | 1.00E-008 | 58.0 |
| candidate_1689 | RF00174;Cobalamin;ABYB01000334.1/10936-11151 | 92.68 | 41 | 3 | 0 | 23 | 63 | 51 | 91 | 1.00E-008 | 58.0 |
| candidate_1689 | RF00174;Cobalamin;ABYC01000415.1/35239-35388 | 92.68 | 41 | 3 | 0 | 23 | 63 | 3 | 43 | 1.00E-008 | 58.0 |
| candidate_1689 | RF00174;Cobalamin;AM420293.1/5303054-5303317 | 92.68 | 41 | 3 | 0 | 23 | 63 | 71 | 111 | 1.00E-008 | 58.0 |
| candidate_1689 | RF00174;Cobalamin;ABTA01000016.1/25834-25981 | 92.68 | 41 | 3 | 0 | 23 | 63 | 2 | 42 | 1.00E-008 | 58.0 |
| candidate_1689 | RF00174;Cobalamin;ABUS01000005.1/442853-443000 | 92.68 | 41 | 3 | 0 | 23 | 63 | 2 | 42 | 1.00E-008 | 58.0 |
| candidate_1689 | RF00174;Cobalamin;ABTI01000053.1/53531-53703 | 92.68 | 41 | 3 | 0 | 23 | 63 | 2 | 42 | 1.00E-008 | 58.0 |
| candidate_1689 | RF00174;Cobalamin;CP000113.1/8100515-8100727 | 92.31 | 39 | 3 | 0 | 25 | 63 | 47 | 85 | 2.00E-007 | 54.0 |
| candidate_1689 | RF00174;Cobalamin;ABUI01000035.1/27356-27519 | 90.7 | 43 | 4 | 0 | 23 | 65 | 2 | 44 | 2.00E-007 | 54.0 |
| candidate_1689 | RF00174;Cobalamin;AM420293.1/1414388-1414592 | 92.31 | 39 | 3 | 0 | 25 | 63 | 44 | 82 | 2.00E-007 | 54.0 |
| candidate_1689 | RF00174;Cobalamin;ABUM01000004.1/65519-65703 | 92.31 | 39 | 3 | 0 | 25 | 63 | 44 | 82 | 2.00E-007 | 54.0 |
| candidate_1689 | RF00174;Cobalamin;ABUD01000001.1/219828-220105 | 90.7 | 43 | 4 | 0 | 23 | 65 | 90 | 132 | 2.00E-007 | 54.0 |
| candidate_1689 | RF00174;Cobalamin;CP000820.1/4201058-4201213 | 90.7 | 43 | 4 | 0 | 23 | 65 | 2 | 44 | 2.00E-007 | 54.0 |
| candidate_1689 | RF00174;Cobalamin;CP000481.1/1942077-1942287 | 92.31 | 39 | 3 | 0 | 25 | 63 | 46 | 84 | 2.00E-007 | 54.0 |
| candidate_1689 | RF00174;Cobalamin;CP000481.1/1939207-1939426 | 92.31 | 39 | 3 | 0 | 25 | 63 | 51 | 89 | 2.00E-007 | 54.0 |
| candidate_1689 | RF00174;Cobalamin;ACBV01000034.1/6489-6629 | 90.7 | 43 | 4 | 0 | 23 | 65 | 1 | 43 | 2.00E-007 | 54.0 |
| candidate_1689 | RF00174;Cobalamin;AATD01004072.1/2-200 | 90.48 | 42 | 4 | 0 | 23 | 64 | 47 | 88 | 8.00E-007 | 52.0 |
| candidate_1689 | RF00174;Cobalamin;AF263012.1/9015-9229 | 89.13 | 46 | 5 | 0 | 18 | 63 | 46 | 91 | 8.00E-007 | 52.0 |
| candidate_1689 | RF00174;Cobalamin;ACEX01000415.1/6970-7130 | 90.24 | 41 | 4 | 0 | 23 | 63 | 2 | 42 | 3.00E-006 | 50.1 |
| candidate_1689 | RF00174;Cobalamin;ABTA01000016.1/114032-114247 | 90.24 | 41 | 4 | 0 | 23 | 63 | 56 | 96 | 3.00E-006 | 50.1 |
| candidate_1689 | RF00174;Cobalamin;CP000850.1/5530984-5531130 | 90.24 | 41 | 4 | 0 | 23 | 63 | 2 | 42 | 3.00E-006 | 50.1 |
| candidate_1689 | RF00174;Cobalamin;ACES01000182.1/23171-23389 | 90.24 | 41 | 4 | 0 | 23 | 63 | 53 | 93 | 3.00E-006 | 50.1 |
| candidate_1689 | RF00174;Cobalamin;ABUS01000002.1/716306-716507 | 90.24 | 41 | 4 | 0 | 23 | 63 | 47 | 87 | 3.00E-006 | 50.1 |
| candidate_1689 | RF00174;Cobalamin;CP001620.1/2022576-2022885 | 90.24 | 41 | 4 | 0 | 25 | 65 | 70 | 110 | 3.00E-006 | 50.1 |
| candidate_1689 | RF00174;Cobalamin;AM746676.1/5390207-5390415 | 90 | 40 | 4 | 0 | 25 | 64 | 49 | 88 | 1.00E-005 | 48.1 |
| candidate_1689 | RF00174;Cobalamin;AM746676.1/5397715-5397910 | 92.5 | 40 | 2 | 1 | 25 | 63 | 37 | 76 | 1.00E-005 | 48.1 |
| candidate_1689 | RF00174;Cobalamin;ABCS01000001.1/110179-110390 | 88.64 | 44 | 5 | 0 | 21 | 64 | 41 | 84 | 1.00E-005 | 48.1 |
| candidate_1689 | RF00174;Cobalamin;AAMD01000003.1/110935-111144 | 90 | 40 | 4 | 0 | 25 | 64 | 46 | 85 | 1.00E-005 | 48.1 |
| candidate_1689 | RF00174;Cobalamin;AAMD01000003.1/140765-140975 | 90 | 40 | 4 | 0 | 25 | 64 | 47 | 86 | 1.00E-005 | 48.1 |
| candidate_1689 | RF00174;Cobalamin;ABTV01000002.1/95589-95758 | 88.64 | 44 | 5 | 0 | 22 | 65 | 20 | 63 | 1.00E-005 | 48.1 |
| candidate_1689 | RF00174;Cobalamin;CP000471.1/3914877-3915128 | 89.74 | 39 | 4 | 0 | 25 | 63 | 46 | 84 | 5.00E-005 | 46.1 |
| candidate_1689 | RF00174;Cobalamin;AAMD01000084.1/10237-10455 | 89.74 | 39 | 4 | 0 | 25 | 63 | 47 | 85 | 5.00E-005 | 46.1 |
| candidate_1689 | RF00174;Cobalamin;ABUU01000080.1/35613-35824 | 89.74 | 39 | 4 | 0 | 25 | 63 | 54 | 92 | 5.00E-005 | 46.1 |
| candidate_1689 | RF00174;Cobalamin;ABUH01000001.1/106204-106444 | 89.74 | 39 | 4 | 0 | 25 | 63 | 51 | 89 | 5.00E-005 | 46.1 |
| candidate_1689 | RF00174;Cobalamin;AM746676.1/1313939-1314117 | 88.1 | 42 | 5 | 0 | 23 | 64 | 30 | 71 | 2.00E-004 | 44.1 |
| candidate_1689 | RF00174;Cobalamin;ACBZ01000030.1/4583-4768 | 96 | 25 | 1 | 0 | 25 | 49 | 43 | 67 | 7.00E-004 | 42.1 |
| candidate_1689 | RF00174;Cobalamin;ACEV01000034.1/29479-29700 | 87.8 | 41 | 5 | 0 | 25 | 65 | 56 | 96 | 7.00E-004 | 42.1 |
| candidate_1689 | RF00174;Cobalamin;ABZW01000001.1/822392-822644 | 87.8 | 41 | 5 | 0 | 23 | 63 | 56 | 96 | 7.00E-004 | 42.1 |
| candidate_1689 | RF00174;Cobalamin;AE017283.1/470738-470914 | 89.19 | 37 | 4 | 0 | 27 | 63 | 42 | 78 | 7.00E-004 | 42.1 |
| candidate_1689 | RF00174;Cobalamin;CP000509.1/3078874-3079071 | 87.8 | 41 | 5 | 0 | 25 | 65 | 42 | 82 | 7.00E-004 | 42.1 |
| candidate_1689 | RF00174;Cobalamin;AAFX01019365.1/576-785 | 87.5 | 40 | 5 | 0 | 25 | 64 | 49 | 88 | 0 | 40.1 |
| candidate_1689 | RF00174;Cobalamin;CP000514.1/4279828-4280063 | 90.62 | 32 | 3 | 0 | 34 | 65 | 89 | 120 | 0 | 40.1 |
| candidate_1689 | RF00174;Cobalamin;ABTQ01000112.1/2056-2251 | 90.62 | 32 | 3 | 0 | 23 | 54 | 43 | 74 | 0 | 40.1 |
| candidate_1689 | RF00174;Cobalamin;ABJF01000206.1/7961-8038 | 90.62 | 32 | 3 | 0 | 34 | 65 | 9 | 40 | 0 | 40.1 |
| candidate_1705 | RF00010;RNaseP_bact_a;CP000325.1/1422068-1422493 | 100 | 23 | 0 | 0 | 9 | 31 | 2 | 24 | 2.00E-005 | 46.1 |
| candidate_1705 | RF00010;RNaseP_bact_a;L78818.1/7603-8020 | 100 | 23 | 0 | 0 | 9 | 31 | 2 | 24 | 2.00E-005 | 46.1 |
| candidate_1705 | RF00010;RNaseP_bact_a;ACLF01000194.1/3743-4158 | 95.65 | 23 | 1 | 0 | 8 | 30 | 1 | 23 | 0 | 38.2 |
| candidate_1705 | RF00010;RNaseP_bact_a;ACNO01000025.1/35619-36033 | 95.65 | 23 | 1 | 0 | 9 | 31 | 2 | 24 | 0 | 38.2 |
| candidate_1705 | RF00010;RNaseP_bact_a;AE016958.1/2182516-2182909 | 95.65 | 23 | 1 | 0 | 9 | 31 | 2 | 24 | 0 | 38.2 |
| candidate_1705 | RF00010;RNaseP_bact_a;ACBV01000004.1/123669-124085 | 95.65 | 23 | 1 | 0 | 9 | 31 | 2 | 24 | 0 | 38.2 |
| candidate_1740 | RF00059;TPP;AM408590.1/541031-541141 | 100 | 93 | 0 | 0 | 1 | 93 | 6 | 98 | 1.00E-046 | 184 |
| candidate_1740 | RF00059;TPP;CP000479.1/4856110-4856221 | 98.85 | 87 | 1 | 0 | 4 | 90 | 10 | 96 | 1.00E-040 | 165 |
| candidate_1740 | RF00059;TPP;ACBV01000011.1/62495-62605 | 96.63 | 89 | 3 | 0 | 2 | 90 | 7 | 95 | 4.00E-037 | 153 |
| candidate_1740 | RF00059;TPP;CP000518.1/5599557-5599666 | 95.6 | 91 | 3 | 1 | 1 | 91 | 6 | 95 | 2.00E-033 | 141 |
| candidate_1740 | RF00059;TPP;FM211192.1/377401-377511 | 94.38 | 89 | 5 | 0 | 2 | 90 | 7 | 95 | 3.00E-032 | 137 |
| candidate_1740 | RF00059;TPP;CP000480.1/6773883-6773993 | 93.41 | 91 | 6 | 0 | 1 | 91 | 6 | 96 | 4.00E-031 | 133 |
| candidate_1740 | RF00059;TPP;CP000854.1/884185-884296 | 92.05 | 88 | 7 | 0 | 3 | 90 | 8 | 95 | 6.00E-027 | 119 |
| candidate_1740 | RF00059;TPP;CP000656.1/165375-165481 | 93.18 | 88 | 5 | 1 | 3 | 90 | 4 | 90 | 6.00E-027 | 119 |
| candidate_1740 | RF00059;TPP;CP000580.1/610429-610537 | 92.31 | 91 | 5 | 2 | 2 | 92 | 7 | 95 | 6.00E-024 | 109 |
| candidate_1740 | RF00059;TPP;CP000656.1/1018078-1018190 | 91.76 | 85 | 6 | 1 | 7 | 90 | 12 | 96 | 9.00E-023 | 105 |
| candidate_1740 | RF00059;TPP;CP000511.1/767963-768068 | 90.8 | 87 | 7 | 1 | 4 | 90 | 5 | 90 | 1.00E-021 | 101 |
| candidate_1740 | RF00059;TPP;CP000480.1/912954-913063 | 90.7 | 86 | 7 | 1 | 5 | 90 | 10 | 94 | 6.00E-021 | 99.6 |
| candidate_1740 | RF00059;TPP;CU458896.1/4261024-4261129 | 95.31 | 64 | 2 | 1 | 27 | 90 | 31 | 93 | 9.00E-020 | 95.6 |
| candidate_1740 | RF00059;TPP;AP006618.1/5617005-5617129 | 97.87 | 47 | 1 | 0 | 44 | 90 | 64 | 110 | 9.00E-017 | 85.7 |
| candidate_1740 | RF00059;TPP;CP000656.1/776321-776432 | 92.54 | 67 | 4 | 1 | 25 | 90 | 30 | 96 | 9.00E-017 | 85.7 |
| candidate_1740 | RF00059;TPP;AP008957.1/1624922-1625029 | 92.19 | 64 | 4 | 1 | 27 | 90 | 31 | 93 | 5.00E-015 | 79.8 |
| candidate_1740 | RF00059;TPP;CP000431.1/2292277-2292384 | 93.75 | 64 | 2 | 2 | 27 | 90 | 31 | 92 | 5.00E-015 | 79.8 |
| candidate_1740 | RF00059;TPP;ABUM01000010.1/118286-118415 | 95.65 | 46 | 2 | 0 | 46 | 91 | 72 | 117 | 8.00E-014 | 75.8 |
| candidate_1740 | RF00059;TPP;CP000249.1/455994-456101 | 90.77 | 65 | 5 | 1 | 27 | 91 | 30 | 93 | 3.00E-013 | 73.8 |
| candidate_1740 | RF00059;TPP;ABVA01000001.1/814009-814120 | 95.35 | 43 | 2 | 0 | 48 | 90 | 54 | 96 | 5.00E-012 | 69.9 |
| candidate_1740 | RF00059;TPP;ACEV01000087.1/557663-557795 | 95.12 | 41 | 2 | 0 | 51 | 91 | 78 | 118 | 8.00E-011 | 65.9 |
| candidate_1740 | RF00059;TPP;ABTA01000007.1/113267-113395 | 91.84 | 49 | 4 | 0 | 42 | 90 | 63 | 111 | 8.00E-011 | 65.9 |
| candidate_1740 | RF00059;TPP;ABUZ01000065.1/11224-11337 | 100 | 32 | 0 | 0 | 60 | 91 | 66 | 97 | 3.00E-010 | 63.9 |
| candidate_1740 | RF00059;TPP;ABTI01000014.1/173316-173424 | 90.62 | 64 | 4 | 2 | 27 | 90 | 32 | 93 | 3.00E-010 | 63.9 |
| candidate_1740 | RF00059;TPP;ABUC01000003.1/510042-510169 | 100 | 31 | 0 | 0 | 60 | 90 | 81 | 111 | 1.00E-009 | 61.9 |
| candidate_1740 | RF00059;TPP;ABTU01000010.1/90740-90852 | 100 | 31 | 0 | 0 | 60 | 90 | 67 | 97 | 1.00E-009 | 61.9 |
| candidate_1740 | RF00059;TPP;AM420293.1/566638-566761 | 90 | 50 | 5 | 0 | 42 | 91 | 61 | 110 | 5.00E-009 | 60.0 |
| candidate_1740 | RF00059;TPP;CT573213.2/873916-874022 | 92 | 50 | 3 | 1 | 43 | 92 | 45 | 93 | 5.00E-009 | 60.0 |
| candidate_1740 | RF00059;TPP;ABUU01000186.1/160-300 | 91.11 | 45 | 4 | 0 | 46 | 90 | 80 | 124 | 2.00E-008 | 58.0 |
| candidate_1740 | RF00059;TPP;ABTV01000003.1/299954-300067 | 96.77 | 31 | 1 | 0 | 60 | 90 | 68 | 98 | 3.00E-007 | 54.0 |
| candidate_1740 | RF00059;TPP;ACEX01000403.1/5097-5211 | 100 | 25 | 0 | 0 | 67 | 91 | 78 | 102 | 5.00E-006 | 50.1 |
| candidate_1740 | RF00059;TPP;ABJG01000322.1/6634-6782 | 100 | 25 | 0 | 0 | 67 | 91 | 110 | 134 | 5.00E-006 | 50.1 |
| candidate_1740 | RF00059;TPP;ABJH01000318.1/27235-27393 | 100 | 25 | 0 | 0 | 67 | 91 | 120 | 144 | 5.00E-006 | 50.1 |
| candidate_1740 | RF00059;TPP;ACEX01000579.1/4826-4939 | 92.68 | 41 | 2 | 1 | 51 | 90 | 58 | 98 | 5.00E-006 | 50.1 |
| candidate_1740 | RF00059;TPP;ABYC01000262.1/93324-93481 | 100 | 25 | 0 | 0 | 67 | 91 | 119 | 143 | 5.00E-006 | 50.1 |
| candidate_1740 | RF00059;TPP;ABYB01000182.1/24362-24497 | 100 | 25 | 0 | 0 | 67 | 91 | 97 | 121 | 5.00E-006 | 50.1 |
| candidate_1740 | RF00059;TPP;BA000030.3/5233679-5233835 | 100 | 25 | 0 | 0 | 67 | 91 | 118 | 142 | 5.00E-006 | 50.1 |
| candidate_1740 | RF00059;TPP;ABYA01000416.1/19428-19539 | 92.68 | 41 | 2 | 1 | 51 | 90 | 56 | 96 | 5.00E-006 | 50.1 |
| candidate_1740 | RF00059;TPP;ABJJ01000418.1/19164-19353 | 100 | 25 | 0 | 0 | 67 | 91 | 151 | 175 | 5.00E-006 | 50.1 |
| candidate_1740 | RF00059;TPP;AP009493.1/4274187-4274320 | 100 | 25 | 0 | 0 | 67 | 91 | 95 | 119 | 5.00E-006 | 50.1 |
| candidate_1740 | RF00059;TPP;CP000968.1/872492-872607 | 96.97 | 33 | 0 | 1 | 59 | 90 | 67 | 99 | 5.00E-006 | 50.1 |
| candidate_1740 | RF00059;TPP;AAFX01073425.1/376-466 | 96.88 | 32 | 0 | 1 | 60 | 90 | 44 | 75 | 2.00E-005 | 48.1 |
| candidate_1740 | RF00059;TPP;AAFX01130813.1/324-431 | 96.88 | 32 | 0 | 1 | 60 | 90 | 61 | 92 | 2.00E-005 | 48.1 |
| candidate_1740 | RF00059;TPP;CP000812.1/1355659-1355767 | 96.88 | 32 | 0 | 1 | 60 | 90 | 62 | 93 | 2.00E-005 | 48.1 |
| candidate_1740 | RF00059;TPP;ACCJ01000005.1/23010-23116 | 90 | 40 | 4 | 0 | 50 | 89 | 51 | 90 | 2.00E-005 | 48.1 |
| candidate_1740 | RF00059;TPP;ABJF01000217.1/23202-23348 | 100 | 24 | 0 | 0 | 67 | 90 | 108 | 131 | 2.00E-005 | 48.1 |
| candidate_1740 | RF00059;TPP;ACEW01000329.1/68110-68254 | 100 | 24 | 0 | 0 | 67 | 90 | 106 | 129 | 2.00E-005 | 48.1 |
| candidate_1740 | RF00059;TPP;CP000820.1/6805029-6805127 | 93.75 | 32 | 2 | 0 | 60 | 91 | 68 | 99 | 2.00E-005 | 48.1 |
| candidate_1740 | RF00059;TPP;CT573213.2/1668077-1668201 | 93.75 | 32 | 2 | 0 | 60 | 91 | 77 | 108 | 2.00E-005 | 48.1 |
| candidate_1740 | RF00059;TPP;BAAY01028811.1/785-886 | 96.77 | 31 | 0 | 1 | 59 | 88 | 54 | 84 | 7.00E-005 | 46.1 |
| candidate_1740 | RF00059;TPP;BAAV01006269.1/966-1080 | 96.77 | 31 | 0 | 1 | 60 | 89 | 66 | 96 | 7.00E-005 | 46.1 |
| candidate_1740 | RF00059;TPP;ABUW01000001.1/62674-62762 | 100 | 23 | 0 | 0 | 68 | 90 | 63 | 85 | 7.00E-005 | 46.1 |
| candidate_1740 | RF00059;TPP;CP000089.1/4217781-4217877 | 93.55 | 31 | 2 | 0 | 59 | 89 | 50 | 80 | 7.00E-005 | 46.1 |
| candidate_1740 | RF00059;TPP;EF681139.1/8926-9018 | 96.77 | 31 | 0 | 1 | 60 | 89 | 45 | 75 | 7.00E-005 | 46.1 |
| candidate_1740 | RF00059;TPP;ABTS01000001.1/395745-395839 | 100 | 23 | 0 | 0 | 68 | 90 | 58 | 80 | 7.00E-005 | 46.1 |
| candidate_1740 | RF00059;TPP;CAAN02269759.1/6-66 | 92.86 | 42 | 1 | 2 | 50 | 90 | 5 | 45 | 3.00E-004 | 44.1 |
| candidate_1740 | RF00059;TPP;BAAY01021929.1/159-270 | 100 | 22 | 0 | 0 | 69 | 90 | 75 | 96 | 3.00E-004 | 44.1 |
| candidate_1740 | RF00059;TPP;AACY020302796.1/1073-1192 | 100 | 22 | 0 | 0 | 68 | 89 | 82 | 103 | 3.00E-004 | 44.1 |
| candidate_1740 | RF00059;TPP;ABMI01026253.1/2-78 | 92.86 | 42 | 1 | 2 | 50 | 90 | 37 | 77 | 3.00E-004 | 44.1 |
| candidate_1740 | RF00059;TPP;AACY023802730.1/527-603 | 92.86 | 42 | 1 | 2 | 50 | 90 | 37 | 77 | 3.00E-004 | 44.1 |
| candidate_1740 | RF00059;TPP;AAQK01008810.1/1691-1800 | 96.67 | 30 | 0 | 1 | 59 | 87 | 66 | 95 | 3.00E-004 | 44.1 |
| candidate_1740 | RF00059;TPP;CP000148.1/3292802-3292904 | 100 | 22 | 0 | 0 | 68 | 89 | 65 | 86 | 3.00E-004 | 44.1 |
| candidate_1740 | RF00059;TPP;ACIQ01000064.1/3481-3583 | 100 | 22 | 0 | 0 | 68 | 89 | 65 | 86 | 3.00E-004 | 44.1 |
| candidate_1740 | RF00059;TPP;AP006840.1/3180549-3180655 | 100 | 22 | 0 | 0 | 68 | 89 | 69 | 90 | 3.00E-004 | 44.1 |
| candidate_1740 | RF00059;TPP;AAWV02000002.1/457796-457896 | 93.33 | 30 | 2 | 0 | 60 | 89 | 55 | 84 | 3.00E-004 | 44.1 |
| candidate_1740 | RF00059;TPP;ABXJ01000068.1/43553-43614 | 100 | 22 | 0 | 0 | 68 | 89 | 24 | 45 | 3.00E-004 | 44.1 |
| candidate_1740 | RF00059;TPP;AM420293.1/1093457-1093545 | 90.48 | 42 | 3 | 1 | 50 | 90 | 46 | 87 | 3.00E-004 | 44.1 |
| candidate_1740 | RF00059;TPP;BABE01014637.1/9-89 | 100 | 21 | 0 | 0 | 69 | 89 | 44 | 64 | 0 | 42.1 |
| candidate_1740 | RF00059;TPP;AACY020298801.1/2858-2957 | 96 | 25 | 1 | 0 | 67 | 91 | 61 | 85 | 0 | 42.1 |
| candidate_1740 | RF00059;TPP;CP000771.1/1540195-1540307 | 90.24 | 41 | 3 | 1 | 50 | 89 | 55 | 95 | 0 | 42.1 |
| candidate_1740 | RF00059;TPP;ABED02000025.1/15684-15784 | 96 | 25 | 1 | 0 | 68 | 92 | 63 | 87 | 0 | 42.1 |
| candidate_1740 | RF00059;TPP;CP001251.1/1671125-1671244 | 93.94 | 33 | 1 | 1 | 60 | 91 | 72 | 104 | 0 | 42.1 |
| candidate_1740 | RF00059;TPP;AAXG02000004.1/132768-132885 | 96.55 | 29 | 0 | 1 | 60 | 87 | 70 | 98 | 0 | 42.1 |
| candidate_1740 | RF00059;TPP;ABYB01000080.1/970-1083 | 90.24 | 41 | 3 | 1 | 51 | 90 | 57 | 97 | 0 | 42.1 |
| candidate_1740 | RF00059;TPP;ACES01000163.1/7274-7381 | 93.94 | 33 | 1 | 1 | 59 | 90 | 60 | 92 | 0 | 42.1 |
| candidate_1740 | RF00059;TPP;CP000475.1/286143-286252 | 96 | 25 | 1 | 0 | 59 | 83 | 63 | 87 | 0 | 42.1 |
| candidate_1740 | RF00059;TPP;CP001341.1/2496265-2496372 | 96 | 25 | 1 | 0 | 59 | 83 | 61 | 85 | 0 | 42.1 |
| candidate_1740 | RF00059;TPP;BABB01009298.1/130-248 | 100 | 20 | 0 | 0 | 68 | 87 | 81 | 100 | 0.01 | 40.1 |
| candidate_1740 | RF00059;TPP;BAAU01007906.1/1091-1205 | 100 | 20 | 0 | 0 | 68 | 87 | 77 | 96 | 0.01 | 40.1 |
| candidate_1740 | RF00059;TPP;ABSN01022598.1/551-650 | 100 | 20 | 0 | 0 | 68 | 87 | 62 | 81 | 0.01 | 40.1 |
| candidate_1740 | RF00059;TPP;AACY023342574.1/1295-1400 | 90 | 40 | 3 | 1 | 52 | 90 | 51 | 90 | 0.01 | 40.1 |
| candidate_1740 | RF00059;TPP;ABSP01013859.1/563-654 | 93.75 | 32 | 1 | 1 | 59 | 89 | 44 | 75 | 0.01 | 40.1 |
| candidate_1740 | RF00059;TPP;AAXY01000001.1/116185-116293 | 93.75 | 32 | 1 | 1 | 59 | 89 | 61 | 92 | 0.01 | 40.1 |
| candidate_1740 | RF00059;TPP;CP000252.1/2063731-2063843 | 95.83 | 24 | 1 | 0 | 68 | 91 | 76 | 99 | 0.01 | 40.1 |
| candidate_1740 | RF00059;TPP;AE017180.1/622575-622727 | 93.75 | 32 | 1 | 1 | 59 | 89 | 105 | 136 | 0.01 | 40.1 |
| candidate_1740 | RF00059;TPP;CP001087.1/2561228-2561330 | 100 | 20 | 0 | 0 | 68 | 87 | 65 | 84 | 0.01 | 40.1 |
| candidate_1740 | RF00059;TPP;AAMB02000001.1/726566-726671 | 100 | 20 | 0 | 0 | 68 | 87 | 68 | 87 | 0.01 | 40.1 |
| candidate_1740 | RF00059;TPP;ABCL01000005.1/82967-83069 | 93.75 | 32 | 1 | 1 | 59 | 89 | 55 | 86 | 0.01 | 40.1 |
| candidate_1740 | RF00059;TPP;CP000489.1/34800-34903 | 93.75 | 32 | 1 | 1 | 59 | 89 | 56 | 87 | 0.01 | 40.1 |
| candidate_1740 | RF00059;TPP;CP000679.1/255822-255947 | 93.75 | 32 | 1 | 1 | 60 | 90 | 79 | 110 | 0.01 | 40.1 |
| candidate_1740 | RF00059;TPP;CP001146.1/1487736-1487855 | 93.75 | 32 | 1 | 1 | 60 | 90 | 72 | 103 | 0.01 | 40.1 |
| candidate_1740 | RF00059;TPP;CP000686.1/1267620-1267721 | 100 | 20 | 0 | 0 | 68 | 87 | 63 | 82 | 0.01 | 40.1 |
| candidate_1740 | RF00059;TPP;CP000804.1/1827092-1827192 | 100 | 20 | 0 | 0 | 68 | 87 | 63 | 82 | 0.01 | 40.1 |
| candidate_1740 | RF00059;TPP;ABZY01000050.1/14436-14532 | 93.75 | 32 | 1 | 1 | 59 | 89 | 49 | 80 | 0.01 | 40.1 |
| candidate_1740 | RF00059;TPP;AE015928.1/2977734-2977830 | 100 | 20 | 0 | 0 | 67 | 86 | 58 | 77 | 0.01 | 40.1 |
| candidate_1740 | RF00059;TPP;CP000386.1/2112145-2112255 | 93.75 | 32 | 1 | 1 | 60 | 90 | 64 | 95 | 0.01 | 40.1 |
| candidate_1740 | RF00059;TPP;CP000386.1/2803868-2803972 | 93.75 | 32 | 1 | 1 | 60 | 90 | 58 | 89 | 0.01 | 40.1 |
| candidate_1740 | RF00059;TPP;CP000474.1/2690645-2690751 | 95.83 | 24 | 1 | 0 | 60 | 83 | 61 | 84 | 0.01 | 40.1 |
| candidate_1740 | RF00059;TPP;CP000559.1/257484-257594 | 95.83 | 24 | 1 | 0 | 68 | 91 | 75 | 98 | 0.01 | 40.1 |
| candidate_1777 | RF00379;ydaO-yuaA;ACHP01000024.1/35645-35867 | 100 | 45 | 0 | 0 | 1 | 45 | 49 | 93 | 2.00E-018 | 89.7 |
| candidate_1777 | RF00379;ydaO-yuaA;ABGN01000022.1/10987-11148 | 100 | 45 | 0 | 0 | 1 | 45 | 49 | 93 | 2.00E-018 | 89.7 |
| candidate_1777 | RF00379;ydaO-yuaA;ABGL01000010.1/286738-286960 | 100 | 45 | 0 | 0 | 1 | 45 | 49 | 93 | 2.00E-018 | 89.7 |
| candidate_1777 | RF00379;ydaO-yuaA;CP000325.1/290980-291193 | 97.73 | 44 | 1 | 0 | 1 | 44 | 50 | 93 | 2.00E-015 | 79.8 |
| candidate_1777 | RF00379;ydaO-yuaA;ACBV01000079.1/26483-26704 | 97.73 | 44 | 1 | 0 | 1 | 44 | 49 | 92 | 2.00E-015 | 79.8 |
| candidate_1777 | RF00379;ydaO-yuaA;ACBV01000022.1/53288-53511 | 97.73 | 44 | 1 | 0 | 1 | 44 | 49 | 92 | 2.00E-015 | 79.8 |
| candidate_1777 | RF00379;ydaO-yuaA;CP000580.1/5106747-5106951 | 100 | 22 | 0 | 0 | 1 | 22 | 53 | 74 | 1.00E-004 | 44.1 |
| candidate_1777 | RF00379;ydaO-yuaA;CP000511.1/5402727-5402940 | 100 | 22 | 0 | 0 | 1 | 22 | 57 | 78 | 1.00E-004 | 44.1 |
| candidate_1777 | RF00379;ydaO-yuaA;ABIN01000324.1/17520-17728 | 90.24 | 41 | 3 | 1 | 1 | 41 | 50 | 89 | 4.00E-004 | 42.1 |
| candidate_1777 | RF00379;ydaO-yuaA;ACFI01000032.1/188781-188993 | 90.24 | 41 | 3 | 1 | 1 | 41 | 52 | 91 | 4.00E-004 | 42.1 |
| candidate_1777 | RF00379;ydaO-yuaA;CP000480.1/5785361-5785569 | 90.91 | 33 | 3 | 0 | 1 | 33 | 54 | 86 | 4.00E-004 | 42.1 |
| candidate_1777 | RF00379;ydaO-yuaA;CP000656.1/1774549-1774764 | 100 | 21 | 0 | 0 | 1 | 21 | 58 | 78 | 4.00E-004 | 42.1 |
| candidate_1791 | RF00174;Cobalamin;CP000717.1/1265267-1265417 | 100 | 33 | 0 | 0 | 1 | 33 | 94 | 126 | 2.00E-011 | 65.9 |
| candidate_1791 | RF00174;Cobalamin;AM420293.1/5303054-5303317 | 96.3 | 27 | 1 | 0 | 1 | 27 | 175 | 201 | 2.00E-005 | 46.1 |
| candidate_1791 | RF00174;Cobalamin;ABTV01000002.1/95589-95758 | 100 | 23 | 0 | 0 | 1 | 23 | 106 | 128 | 2.00E-005 | 46.1 |
| candidate_1791 | RF00174;Cobalamin;ACBV01000034.1/6489-6629 | 96.15 | 26 | 1 | 0 | 1 | 26 | 83 | 108 | 7.00E-005 | 44.1 |
| candidate_1791 | RF00174;Cobalamin;ABUU01000080.1/35613-35824 | 100 | 21 | 0 | 0 | 1 | 21 | 140 | 160 | 3.00E-004 | 42.1 |
| candidate_1791 | RF00174;Cobalamin;ABGO01000157.1/12361-12571 | 100 | 20 | 0 | 0 | 1 | 20 | 133 | 152 | 0 | 40.1 |
| candidate_1791 | RF00174;Cobalamin;ABUU01000087.1/24235-24436 | 100 | 20 | 0 | 0 | 1 | 20 | 130 | 149 | 0 | 40.1 |
| candidate_1791 | RF00174;Cobalamin;ABJH01000030.1/15760-15986 | 100 | 20 | 0 | 0 | 1 | 20 | 138 | 157 | 0 | 40.1 |
| candidate_1791 | RF00174;Cobalamin;ACEV01000048.1/217132-217351 | 100 | 20 | 0 | 0 | 1 | 20 | 138 | 157 | 0 | 40.1 |
| candidate_1791 | RF00174;Cobalamin;AL939107.1/133900-134052 | 100 | 20 | 0 | 0 | 1 | 20 | 92 | 111 | 0 | 40.1 |
| candidate_1791 | RF00174;Cobalamin;ACEU01000643.1/1615-1835 | 100 | 20 | 0 | 0 | 1 | 20 | 140 | 159 | 0 | 40.1 |
| candidate_1791 | RF00174;Cobalamin;ACEX01000415.1/6970-7130 | 100 | 20 | 0 | 0 | 1 | 20 | 91 | 110 | 0 | 40.1 |
| candidate_1791 | RF00174;Cobalamin;ABYC01000415.1/35239-35388 | 100 | 20 | 0 | 0 | 1 | 20 | 92 | 111 | 0 | 40.1 |
| candidate_1791 | RF00174;Cobalamin;ABTA01000016.1/25834-25981 | 100 | 20 | 0 | 0 | 1 | 20 | 90 | 109 | 0 | 40.1 |
| candidate_1791 | RF00174;Cobalamin;CP000850.1/5530984-5531130 | 100 | 20 | 0 | 0 | 1 | 20 | 88 | 107 | 0 | 40.1 |
| candidate_1791 | RF00174;Cobalamin;ACES01000182.1/23171-23389 | 100 | 20 | 0 | 0 | 1 | 20 | 139 | 158 | 0 | 40.1 |
| candidate_1791 | RF00174;Cobalamin;ABUD01000001.1/219828-220105 | 100 | 20 | 0 | 0 | 1 | 20 | 178 | 197 | 0 | 40.1 |
| candidate_1791 | RF00174;Cobalamin;ABUS01000002.1/716306-716507 | 100 | 20 | 0 | 0 | 1 | 20 | 130 | 149 | 0 | 40.1 |
| candidate_1791 | RF00174;Cobalamin;CP000820.1/4201058-4201213 | 100 | 20 | 0 | 0 | 1 | 20 | 90 | 109 | 0 | 40.1 |
| candidate_1791 | RF00174;Cobalamin;CP000481.1/1939207-1939426 | 100 | 20 | 0 | 0 | 1 | 20 | 131 | 150 | 0 | 40.1 |
| candidate_1791 | RF00174;Cobalamin;AP008957.1/5154507-5154732 | 100 | 20 | 0 | 0 | 1 | 20 | 145 | 164 | 0 | 40.1 |
| candidate_1791 | RF00174;Cobalamin;CP000480.1/6689978-6690152 | 100 | 20 | 0 | 0 | 1 | 20 | 117 | 136 | 0 | 40.1 |
| candidate_1791 | RF00174;Cobalamin;ABTA01000016.1/114032-114247 | 95.65 | 23 | 1 | 0 | 1 | 23 | 144 | 166 | 0 | 38.2 |
