# Supplementary material for: A Genome-Wide Identification Analysis of Small Regulatory RNAs in Mycobacterium tuberculosis by RNA-Seq and Conservation Analysis
Source: PLoS One. 2012 Mar 28;7(3):e32723. doi: 10.1371/journal.pone.0032723 (PMC3314655; doi:10.1371/journal.pone.0032723)
Supplement: Table S5 — Infernal output of comparison between secondary structure candidates sequences and secondary structure of ncRNA in Rfam V.10 database. (DOC) [file pone.0032723.s006.doc]

| SAM-IV | candidate_852 | 2 | 121 | 1 | 116 | 100.95 | 1.16E-022 | 72 |
| --- | --- | --- | --- | --- | --- | --- | --- | --- |
| SAM-IV | candidate_1909 | 134 | 15 | 1 | 116 | 100.95 | 1.16E-022 | 72 |
| tRNA | candidate_1433 | 73 | 3 | 1 | 71 | 71.67 | 1.03E-017 | 62 |
| tRNA | candidate_1028 | 147 | 74 | 1 | 71 | 70.39 | 2.37E-017 | 57 |
| 6C | candidate_877 | 77 | 2 | 1 | 76 | 73.39 | 1.35E-014 | 72 |
| 6C | candidate_1603 | 18 | 93 | 1 | 76 | 73.39 | 1.35E-014 | 72 |
| Bacteria_small_SRP | candidate_1621 | 1 | 86 | 5 | 90 | 50.97 | 2.33E-012 | 71 |
| tRNA | candidate_1116 | 70 | 1 | 2 | 69 | 50.42 | 1.08E-011 | 60 |
| TPP | candidate_682 | 88 | 1 | 9 | 90 | 42.61 | 2.80E-010 | 70 |
| TPP | candidate_1740 | 4 | 91 | 9 | 90 | 42.61 | 2.80E-010 | 70 |
| tRNA | candidate_900 | 85 | 3 | 1 | 71 | 44.23 | 6.10E-010 | 67 |
| tRNA-Sec | candidate_1433 | 73 | 4 | 1 | 91 | 41.67 | 8.08E-010 | 63 |
| Bacteria_small_SRP | candidate_658 | 68 | 1 | 14 | 79 | 42.23 | 8.65E-010 | 66 |
| Actino-pnp | candidate_816 | 83 | 13 | 1 | 61 | 42.89 | 1.58E-009 | 70 |
| Actino-pnp | candidate_1874 | 125 | 195 | 1 | 61 | 42.89 | 1.58E-009 | 70 |
| TPP | candidate_681 | 1 | 85 | 10 | 89 | 39.5 | 2.19E-009 | 69 |
| TPP | candidate_1739 | 85 | 1 | 10 | 89 | 39.5 | 2.19E-009 | 69 |
| Bacteria_large_SRP | candidate_1621 | 5 | 84 | 120 | 199 | 26.77 | 2.74E-005 | 70 |
| tmRNA | candidate_1116 | 24 | 7 | 334 | 351 | 14.91 | 6.27E-005 | 61 |
| SSU_rRNA_bacteria | candidate_1186 | 169 | 141 | 1319 | 1347 | 17.96 | 9.04E-005 | 52 |
| tRNA-Sec | candidate_1028 | 147 | 75 | 1 | 91 | 24.33 | 1.05E-004 | 58 |
| Bacteria_large_SRP | candidate_658 | 59 | 10 | 133 | 182 | 23.95 | 1.46E-004 | 62 |
| tmRNA | candidate_1433 | 26 | 12 | 336 | 350 | 13.84 | 1.59E-004 | 67 |
| rliE | candidate_1254 | 37 | 2 | 145 | 183 | 20.5 | 1.93E-004 | 44 |
| Metazoa_SRP | candidate_497 | 41 | 6 | 130 | 165 | 18.4 | 2.22E-004 | 69 |
| tmRNA | candidate_1356 | 30 | 14 | 335 | 351 | 13.24 | 2.71E-004 | 71 |
| CsrC | candidate_497 | 41 | 6 | 209 | 244 | 16.3 | 3.02E-004 | 69 |
| rliE | candidate_1254 | 1 | 37 | 144 | 183 | 19.76 | 3.10E-004 | 46 |
| CsrC | candidate_497 | 6 | 41 | 209 | 244 | 16.18 | 3.32E-004 | 69 |
| lsy-6 | candidate_98 | 63 | 7 | 1 | 74 | 23.77 | 5.31E-004 | 61 |
| RsaJ | candidate_234 | 1 | 34 | 254 | 287 | 17.37 | 6.24E-004 | 47 |
| RsaJ | candidate_1254 | 36 | 3 | 254 | 287 | 17.37 | 6.24E-004 | 47 |
| .ykoK | candidate_766 | 46 | 82 | 44 | 80 | 20.98 | 8.22E-004 | 59 |
| ykoK | candidate_1828 | 48 | 12 | 44 | 80 | 20.98 | 8.22E-004 | 59 |
| P17 | candidate_988 | 35 | 4 | 251 | 282 | 13.95 | 9.86E-004 | 75 |
| RsaJ | candidate_234 | 36 | 1 | 252 | 287 | 16.43 | 1.20E-003 | 47 |
| RsaJ | candidate_1254 | 1 | 36 | 252 | 287 | 16.43 | 1.20E-003 | 47 |
| rliE | candidate_234 | 1 | 34 | 146 | 182 | 17.5 | 1.34E-003 | 47 |
| Thr_leader | candidate_1151 | 58 | 19 | 79 | 117 | 21.01 | 1.84E-003 | 58 |
| RsaJ | candidate_1430 | 36 | 1 | 252 | 287 | 15.83 | 1.84E-003 | 50 |
| CsrC | candidate_103 | 34 | 1 | 210 | 243 | 13.96 | 1.88E-003 | 59 |
| .CsrC | candidate_103 | 1 | 34 | 210 | 243 | 13.95 | 1.89E-003 | 59 |
| Cobalamin | candidate_1689 | 31 | 58 | 51 | 79 | 16.89 | 1.97E-003 | 64 |
| RsaJ | candidate_949 | 34 | 1 | 254 | 287 | 15.68 | 2.05E-003 | 50 |
| tmRNA | candidate_1028 | 99 | 82 | 334 | 351 | 10.85 | 2.20E-003 | 78 |
| RsaJ | candidate_1430 | 1 | 34 | 254 | 287 | 15.47 | 2.37E-003 | 50 |
| His_leader | candidate_1533 | 1 | 33 | 88 | 120 | 19.88 | 2.46E-003 | 58 |
| rliE | candidate_234 | 34 | 1 | 146 | 182 | 16.48 | 2.60E-003 | 47 |
| Metazoa_SRP | candidate_497 | 6 | 41 | 130 | 165 | 14.85 | 2.65E-003 | 69 |
| P17 | candidate_1430 | 35 | 3 | 249 | 282 | 12.4 | 3.41E-003 | 52 |
| rliE | candidate_949 | 34 | 1 | 146 | 182 | 15.98 | 3.60E-003 | 50 |
| Metazoa_SRP | candidate_658 | 56 | 15 | 177 | 218 | 14.37 | 3.71E-003 | 64 |
| Metazoa_SRP | candidate_1621 | 22 | 63 | 177 | 218 | 14.37 | 3.71E-003 | 64 |
| .CopA | candidate_1497 | 8 | 60 | 33 | 87 | 22.18 | 3.77E-003 | 53 |
| P15 | candidate_949 | 33 | 5 | 91 | 119 | 16.31 | 4.15E-003 | 52 |
| rliE | candidate_949 | 1 | 34 | 146 | 182 | 15.73 | 4.23E-003 | 50 |
| OLE | candidate_1254 | 2 | 37 | 317 | 352 | 13.25 | 4.76E-003 | 44 |
| isrG | candidate_823 | 5 | 64 | 137 | 197 | 13.42 | 5.03E-003 | 67 |
| isrG | candidate_1881 | 60 | 1 | 137 | 197 | 13.42 | 5.03E-003 | 67 |
| isrG | candidate_720 | 1 | 58 | 123 | 211 | 13.42 | 5.04E-003 | 69 |
| lsy-6 | candidate_720 | 54 | 4 | 1 | 74 | 20.25 | 5.26E-003 | 69 |
| lsy-6 | candidate_1781 | 4 | 54 | 1 | 74 | 20.25 | 5.26E-003 | 69 |
| sbcD | candidate_497 | 41 | 6 | 112 | 152 | 14.55 | 5.46E-003 | 69 |
| isrG | candidate_720 | 58 | 1 | 123 | 211 | 13.26 | 5.65E-003 | 69 |
| OLE | candidate_1430 | 1 | 34 | 318 | 351 | 12.89 | 6.19E-003 | 50 |
| rliE | candidate_1430 | 34 | 1 | 146 | 182 | 15.15 | 6.19E-003 | 50 |
| isrG | candidate_1160 | 26 | 75 | 117 | 217 | 13.08 | 6.40E-003 | 60 |
| NrrF | candidate_196 | 36 | 2 | 121 | 160 | 17.33 | 6.57E-003 | 54 |
| rli40 | candidate_1151 | 57 | 20 | 227 | 263 | 13.32 | 6.59E-003 | 55 |
| P17 | candidate_1430 | 1 | 32 | 250 | 282 | 11.56 | 6.66E-003 | 53 |
| Thr_leader | candidate_1151 | 17 | 58 | 77 | 117 | 18.75 | 6.68E-003 | 57 |
| tRNA-Sec | candidate_900 | 85 | 4 | 1 | 91 | 18.21 | 6.68E-003 | 68 |
| isrG | candidate_1781 | 56 | 1 | 124 | 210 | 12.93 | 7.10E-003 | 68 |
| rli41 | candidate_1411 | 45 | 82 | 450 | 487 | 13.54 | 7.14E-003 | 74 |
| RsaJ | candidate_949 | 1 | 34 | 254 | 287 | 13.89 | 7.18E-003 | 50 |
| OLE | candidate_1430 | 34 | 1 | 318 | 351 | 12.64 | 7.42E-003 | 50 |
| His_leader | candidate_1533 | 33 | 1 | 88 | 120 | 17.9 | 7.69E-003 | 58 |
| isrG | candidate_1781 | 1 | 56 | 124 | 210 | 12.77 | 7.95E-003 | 68 |
| sraL | candidate_207 | 2 | 33 | 110 | 141 | 15.49 | 8.32E-003 | 59 |
| sraL | candidate_1214 | 33 | 2 | 110 | 141 | 15.49 | 8.32E-003 | 59 |
| P4 | candidate_720 | 54 | 5 | 139 | 189 | 13.81 | 8.33E-003 | 68 |
| P4 | candidate_1781 | 4 | 53 | 139 | 189 | 13.81 | 8.33E-003 | 68 |
| lsy-6 | candidate_857 | 53 | 9 | 1 | 74 | 19.49 | 8.63E-003 | 69 |
| lsy-6 | candidate_1914 | 5 | 49 | 1 | 74 | 19.49 | 8.63E-003 | 69 |
| CopA | candidate_1497 | 60 | 5 | 32 | 89 | 20.58 | 9.16E-003 | 52 |
| RsaJ | candidate_196 | 36 | 1 | 252 | 287 | 13.52 | 9.32E-003 | 56 |
| NrrF | candidate_1199 | 2 | 34 | 123 | 160 | 16.7 | 9.55E-003 | 55 |
| CsrC | candidate_1151 | 19 | 58 | 210 | 243 | 11.85 | 9.79E-003 | 58 |
