# Supplementary material for: A Genome-Wide Identification Analysis of Small Regulatory RNAs in Mycobacterium tuberculosis by RNA-Seq and Conservation Analysis
Source: PLoS One. 2012 Mar 28;7(3):e32723. doi: 10.1371/journal.pone.0032723 (PMC3314655; doi:10.1371/journal.pone.0032723)
Supplement: Text S1 — Conservation map construction. (DOC) [file pone.0032723.s007.doc]

**Conservation map construction**

The *sequence conservation value* (*C_i_*) of a specific genomic position corresponds to the weighted count of different genomes containing that position with at least one alignment satisfying quality criteria.

$$C_{i}=\sum w_{j}*I_{ij}$$

where

- *C_i_* corresponds to the *conservation value* of the i-th base on the genome and *i* takes all values included in genomic regions within T_IGRcoord database.
- *I_ij_* is an indicator function that takes value equal to 1 if the *j-th* genome contains *C_i_* within at least one hit considering the BLASTN output fulfilling the quality requirements.
- *w_j_* is the weight assigned to each genome in the comparison set corresponding to its evolutionary distance to target genome. The distance is calculated accordingly with the method proposed in the recent literature [28]*.*
