# Supplementary material for: A Genome-Wide Identification Analysis of Small Regulatory RNAs in Mycobacterium tuberculosis by RNA-Seq and Conservation Analysis
Source: PLoS One. 2012 Mar 28;7(3):e32723. doi: 10.1371/journal.pone.0032723 (PMC3314655; doi:10.1371/journal.pone.0032723)
Supplement: Text S2 — Thresholds definition. (DOC) [file pone.0032723.s008.doc]

**Thresholds definition**

A first consideration for threshold definition is that each observation within a given base i is the result of two sources of random values: background noise [29]$X_{bk,i}$and sRNA expression $X_{\text{exp},i}$. We assume that these two sources of randomness are independent.$X_{bk,i}$can be reasonably thought as a random variable assuming positive values and with density distribution $f_{bk}$ not depending on the considered base. Moreover, $X_{\text{exp},i}$is assumed to be a non negative random variable, that takes positive values in coding regions only and assumes distribution $f_{\text{exp},i}$which depends on its expression level. In formula: $X_{i}=X_{bk,i}+X_{\text{exp},i}$.

The observed values $X_{n}$, are random samples drawn from the distribution of $X_{i}$ given by the convolution$f_{x_{i}}\left(z\right)=\int f_{bk}\left(z-x\right)+f_{\text{exp},i}\left(x\right)dx$. The non-negativity of $X_{\text{exp},i}$implies that $X_{bk}$ stochastically dominates each $X_{i}$ and therefore dominates$X$,$X$being the mixture of all $X_{i}$ we actually observe; i.e $F_{bk}\left(x\right)\geq F_{X}\left(x\right)$ or, in other words, $F_{bk,\alpha}\leq F_{X,\alpha}$where is the $\alpha-th$ percentile of the distribution. In this sense$F_{X,\alpha}$represents a more conservative threshold than the corresponding$F_{bk,\alpha}$. As estimate of$F_{X}$we considered its Empirical Distribution Function (EDF) defined as: $\hat{F}_{X,n}\left(x\right)=\frac{1}{n}\sum I\left(x_{n}\leq x\right)$. By the Glivenko-Cantelli theorem we have that $sup_{x}=\left(F_{x,n}\left(x\right)-F_{x}\left(x\right)\right)\to1$with probability 1.

We chose as threshold $F_{x,n,0.95}$and $F_{x,n,0.90}$, respectively the 95-th and the 90-th percentile of the EDF of $X$. In other words, ExprT1 and ConsT1 thresholds correspond to 95th percentile of reads abundance and conservation distributions respectively, while ExprT2 and ConsT2 thresholds correspond to the 90th percentile. This procedure tests the null hypothesis of the presence of a pure background signal with respect to an alternative hypothesis of a mixture of background and sRNA signal, set to a significance level equal to α=0.05 and α=0.10. Setting the threshold on a combination of background noise and sRNA candidates signal represents a conservative approach since it shifts the discriminating cut-off to a larger value and hence to a higher percentiles producing an effective level < α. From the comparative genomics step we consider the percentiles of the conservation distributions as ad hoc cut-offs to identify the more likely promising regions.
